# Computationally Driven Discovery of a BCR-ABL1 Kinase Inhibitor with Activity in Multidrug-Resistant Chronic Myeloid Leukemia

**DOI:** 10.1021/acs.jmedchem.4c01852

**Published:** 2024-09-23

**Authors:** Jarvis Hill, R. Houston Givhan, Bin Yi, Robert M. Jones, Eugene F. Douglass, Yaguang Xi, Henry F. Schaefer, David Crich

**Affiliations:** † Department of Pharmaceutical and Biomedical Sciences, University of Georgia, 250 West Green Street, Athens, Georgia 30602, United States; ‡ Department of Chemistry, University of Georgia, 302 East Campus Road, Athens, Georgia 30602, United States; § Center for Computational Quantum Chemistry, University of Georgia, 1004 Cedar Street, Athens, Georgia 30602, United States; ∥ Independent consultant, P.O. Box 568, Oakley, Utah 84055-0568, United States; ⊥ Complex Carbohydrate Research Center, University of Georgia, 315 Riverbend Road, Athens, Georgia 30602, United States

## Abstract

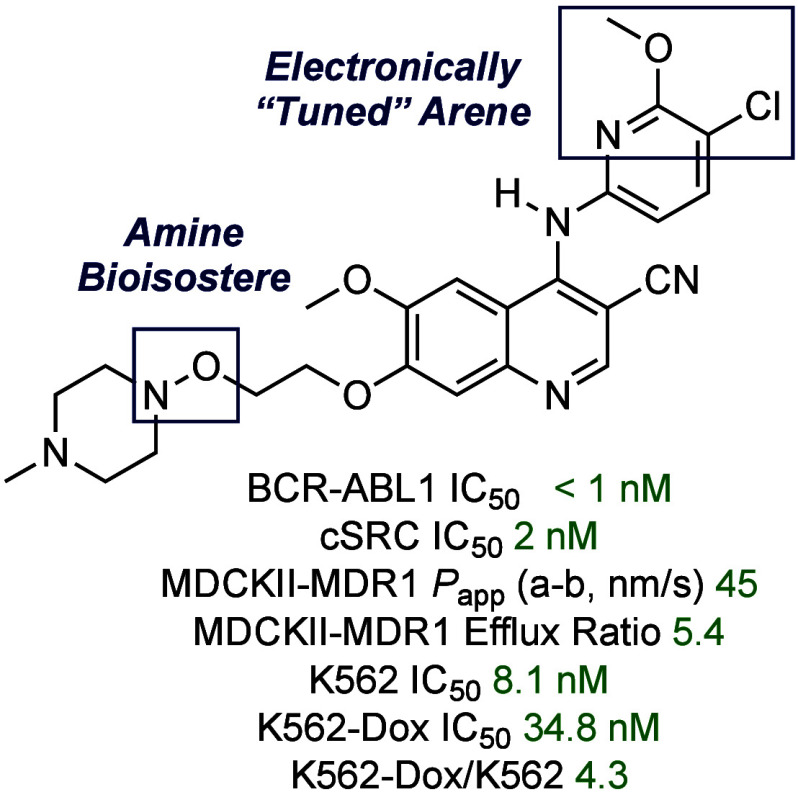

The permeability glycoprotein, encoded by the *ABCB1* gene, is widely implicated in multidrug resistance (MDR), as it has been shown to reduce the intracellular concentration of most small molecule therapeutics, including the majority of the breakpoint cluster region Abelson proto-oncogene 1 (BCR-ABL1) kinase inhibitors used in the treatment of Philadelphia chromosome positive (Ph+) leukemias. With this in mind, we describe an integrated theoretical and experimental approach to shed light on substituent effects in the pendant anilino moiety of 4-anilinoquinazolines and 4-anilinoquinoline-3-carbonitrile-based kinase inhibitors and their influence on P-gp-mediated efflux. This analysis culminated in the identification of a hydroxylamine-bearing, dual cSRC/BCR-ABL1 kinase inhibitor **16a** that exhibits a marked reduction in P-gp-mediated efflux ratio and potent activity in a Ph+ patient-derived cell line (K562) and an MDR-Ph+ patient-derived cell line (K562/Dox) overexpressing P-gp. Overall, we demonstrate that the P-gp-mediated efflux ratio can be minimized by computationally driven optimization of the molecular dipole and/or cp*K*_a_ without recourse to intramolecular hydrogen bonds.

## Introduction

**Figure 1:**
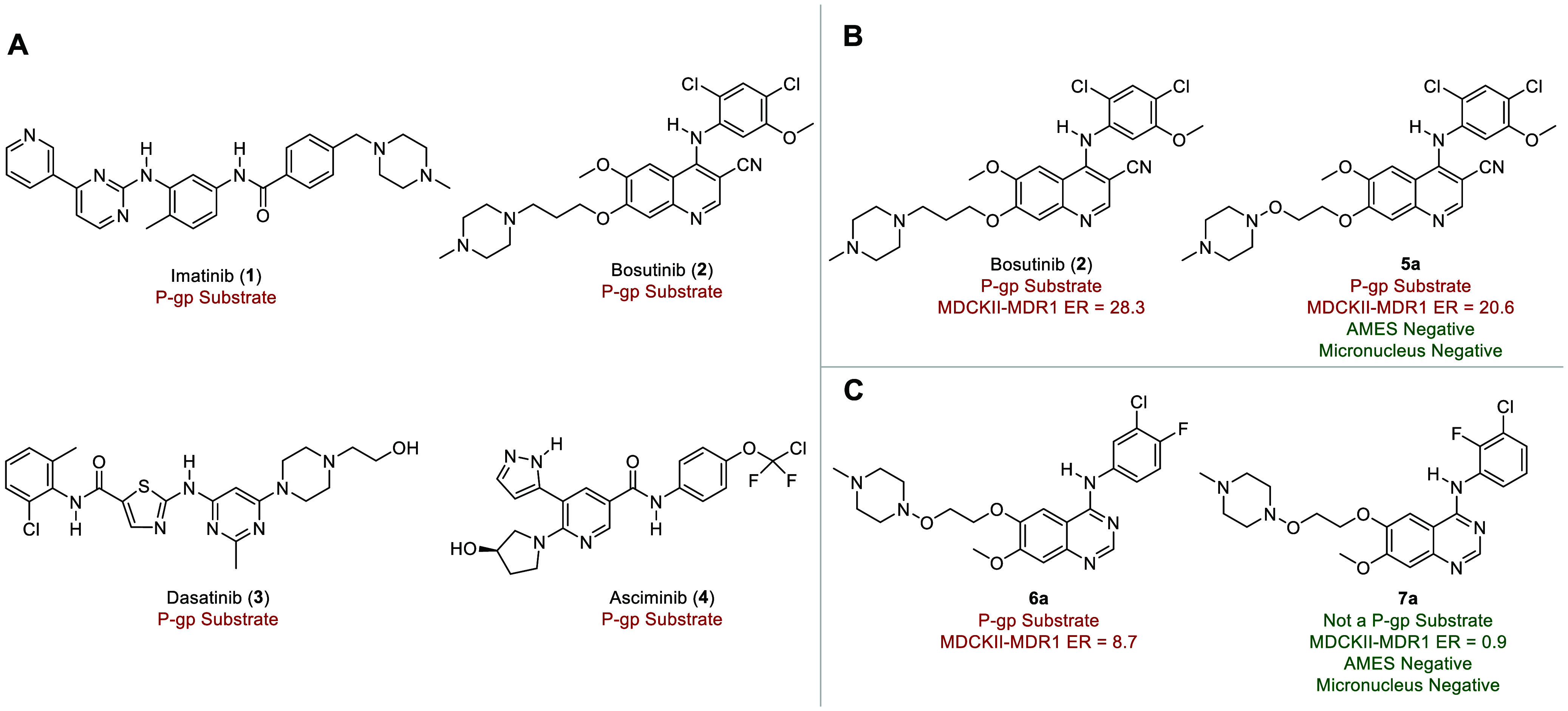
Overview of selected BCR-ABL1 inhibitors and hydroxylamine-based EGFR inhibitors and their efflux status against P-glycoprotein (P-gp). A) Chemical structures of selected BCR-ABL1 inhibitors that suffer from P-gp-mediated efflux. B) Chemical structures of BCR-ABL1 inhibitor, bosutinib (**2**) and its hydroxylamine-based analog (**5a**) and selected absorption, distribution, metabolism, excretion, and toxicity (ADMET) properties previously reported in ref (34). C) Chemical structures of hydroxylamine-based EGFR inhibitors and selected ADMET properties previously reported in ref (33).

Multidrug resistance (MDR) is a major obstacle in the successful clinical management of leukemia,^1−5^ for which several mechanisms have been proposed. While on-target resistance mechanisms, including mutations in the kinase binding domain, remain most common, a growing body of evidence suggests that off-target resistance mechanisms in chronic myeloid leukemia (CML), such as upregulation of the permeability glycoprotein (P-gp; MDR1) efflux pump, a 170 kDa protein encoded by the *ABCB1* gene, can pose a significant clinical issue.^6−13^ This is because most small-molecule targeted therapies for CML are P-gp substrates, including the breakpoint cluster region – Abelson proto-oncogene 1 (BCR-ABL1) first-generation inhibitor imatinib (**1**) and the second- and third-generation inhibitors bosutinib (**2**), dasatinib (**3**) and asciminib (**4**). As a result, it has been suggested that P-gp expression levels can be used as a predictive biomarker for patients at risk of developing off-target resistance in CML (Figure 1A).^9−17^ Thus, future treatment options for Philadelphia chromosome positive (Ph+) leukemias would benefit from reduced P-gp-mediated efflux, and the consequent minimization of potential off-target resistance by P-gp overexpression.

Several strategies have been proposed to overcome P-gp-mediated MDR in oncology, such as coadministration of P-gp inhibitors or modulators, including the coadministration of a second kinase inhibitor, to minimize P-gp-mediated efflux and iterative compound optimization approaches.^18−26^ In our laboratories, we have been exploring trisubstituted hydroxylamines as bioisosteric replacements of alkylidene moieties, ether units and tertiary amines so as to improve drug-like properties without incurring a significant molecular weight (MW) increase.^27−34^ Recently, we reported^34^ a novel *N*-alkyl to *N*-noralkoxy (**5a**) switch in bosutinib that reduced both drug efflux in colon carcinoma (Caco-2) cells and human ether-à-go-go-related gene (hERG) affinity without introducing the metabolic instability, genotoxicity or mutagenicity commonly surmised to accompany hydroxylamine units (Figure 1B).^35,36^ However, in the Madin-Darby canine-kidney (MDCKII)-MDR1 cell line, which overexpresses only P-gp, **5a** showed only a negligible change in efflux over **2** suggesting that the *N*-alkyl to *N*-noralkoxy switch has minimal influence on P-gp substrate recognition in this series, and that **5a** would be subject to off-target resistance by P-gp overexpression. In addition, working with the 4-anilinoquinazoline epidermal growth factor receptor (EGFR) inhibitors (**6a** and **7a**), we recently^33^ showed that a 3-chloro-4-fluoro-substitution pattern on the aniline ring led to moderate P-gp-mediated efflux (MDCKII-MDR1 efflux ratio = 8.7), while a 3-chloro-2-fluoroanilino-substitution pattern completely evaded P-gp-mediated efflux (MDCKII-MDR1 efflux ratio = 0.9) (Figure 1C). Extrapolating from these studies, we now describe an integrated theoretical and experimental approach to shed light on substituent effects in the pendant anilino moiety governing P-gp-mediated efflux in these two critically important classes of kinase inhibitors (4-anilinoquinazolines and 4-anilinoquinoline-3-carbonitriles). This analysis resulted in the identification of a BCR-ABL1 kinase inhibitor, with a reduced P-gp-mediated efflux ratio relative to bosutinib and potent activity in a Ph+ patient derived cell line (K562) and an MDR-Ph+ patient derived cell line (K562/Dox) overexpressing P-gp. Compared to bosutinib, the optimal compound has an over 300-fold improvement in relative resistance in K562/Dox cells, which overexpress P-gp, and exhibits an approximately 60-fold improvement in activity compared to imatinib, and thus is a potential lead in the treatment of MDR Ph+ CML characterized by P-gp overexpression. More broadly, we demonstrate that P-gp-mediated efflux ratios can be minimized by computationally guided optimization of molecular dipole and/or calculated p*K*_a_ (cp*K*_a_) without involvement of intramolecular hydrogen bonds or modification of tPSA (topological polar surface area). We observed that compounds with high cp*K*_a_ values generally exhibited improvements in P-gp-mediated efflux ratios compared to bosutinib, and compounds with lower or no change in cp*K*_a_ values could be optimized by a corresponding decrease in molecular dipole.

## Results and Discussion

**Figure 2:**
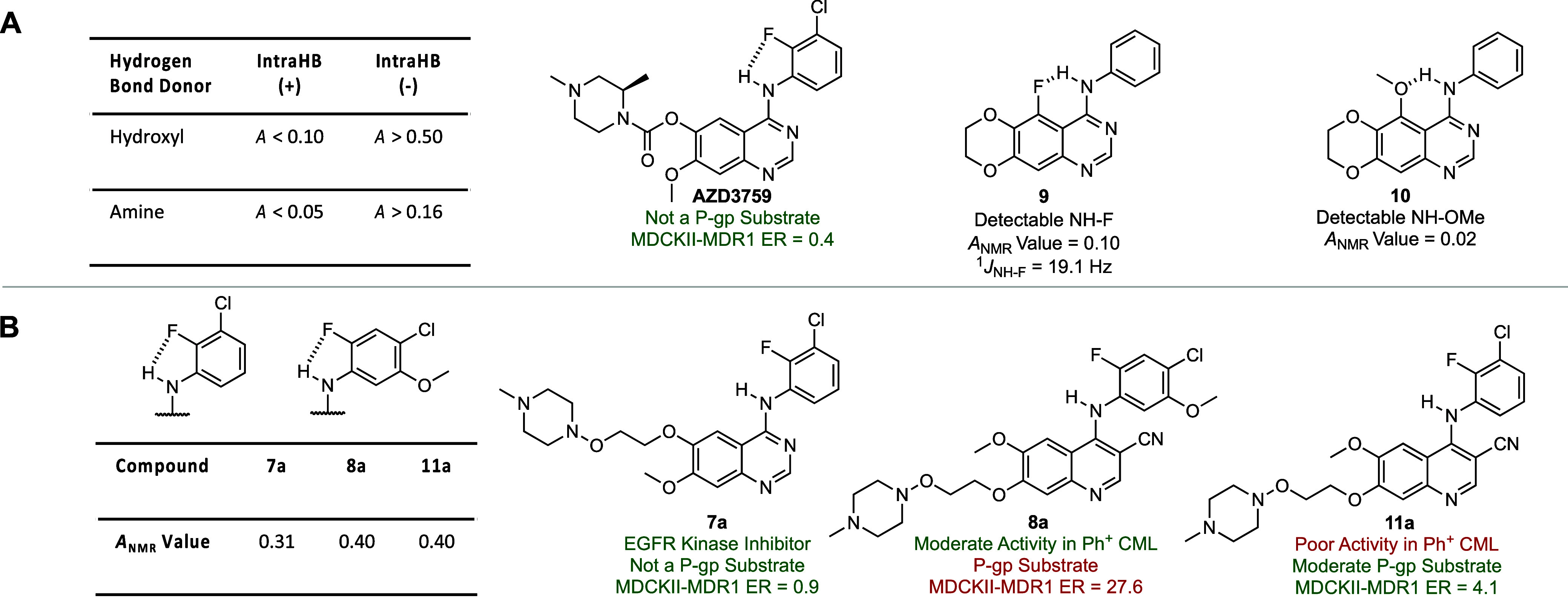
Intramolecular N–H–F hydrogen bond (HB) investigation by NMR and absorption, distribution, metabolism, excretion and toxicity (ADMET) properties of selected 4-anilinoquinazoline inhibitors and 4-anilinoquinoline-3-carbonitrile inhibitors. A) Abraham *A*_NMR_ values for assessing intramolecular hydrogen bonds by NMR and previously reported quinazoline-based kinase inhibitors with potential intramolecular hydrogen bonds. B) Determination of Abraham *A*_NMR_ values by NMR and P-glycoprotein (P-gp) efflux status of hydroxylamine-bearing EGFR inhibitors and BCR-ABL1 inhibitors. See Supporting Information for details on Abraham *A*_NMR_ values.

Recalling the positive influence of the *ortho*-fluorine atom in **7a** on P-gp-mediated efflux ratio, we first prepared **8a** characterized by the *ortho*-chlorine to *ortho*-fluorine switch from **5a** (Figure 1C).^33^ This modification had minimal effect on biochemical inhibition of the relevant mutant kinases (IC_50_’s < 1 nM), but unexpectedly increased the P-gp-mediated efflux ratio (MDCKII-MDR1 efflux ratio = 27.6) relative to parent **5a** (MDCKII-MDR1 efflux ratio = 20.6) (*vide infra*). Previously, intramolecular N–H–F hydrogen bonds (HBs) have been proposed as a strategy to reduce P-gp-mediated efflux and improve passive permeability, as highlighted in the discovery of the brain-penetrant EGFR inhibitor, AZD-3759 (Figure 2A).^37−40^ However, a recent study by Jung and Houk cast doubt on the existence of anilino N–H–F HBs except in conformationally restricted cases such as **9** and showed that they are substantially weaker than comparable N–H–O HBs as in **10** (Figure 2A).^41^ Consistent with this work, we conducted an NMR study of the EGFR inhibitor **7a** and the BCR-ABL1 inhibitor **8a** in deuterated chloroform (CDCl_3_) and in the more polar, HB-accepting dimethyl sulfoxide (DMSO-*d*_6_). Interestingly, we measured Abraham *A*_NMR_ values^42^ inconsistent with an intramolecular HB between the *ortho*-fluorine and adjacent aniline (*A*_NMR_ values >0.30 for aniline N–H in **7a** and **8a**) (see Supporting Information for details) (Figure 2B). This finding is further supported by the lack of ^1^*J*_NH-F_ coupling (≤1 Hz) in **7a** and **8a**, as contrasted with bona fide intramolecular HBs to fluorine, which typically have ^1^*J*_NH-F_ coupling constants >10 Hz as exemplified by **9** with its ^1^*J*_NH-F_ of 19.1 Hz.^41,43^ This leads us to believe the reduced P-gp efflux ratio of **7a**, while caused by the presence of the *ortho*-fluorine atom, is not the result of an intramolecular N–H–F HB; in **8a** the *ortho*-fluorine has the opposite effect and actually increases the efflux ratio in MDCKII-MDR1 cells.

**Figure 3:**
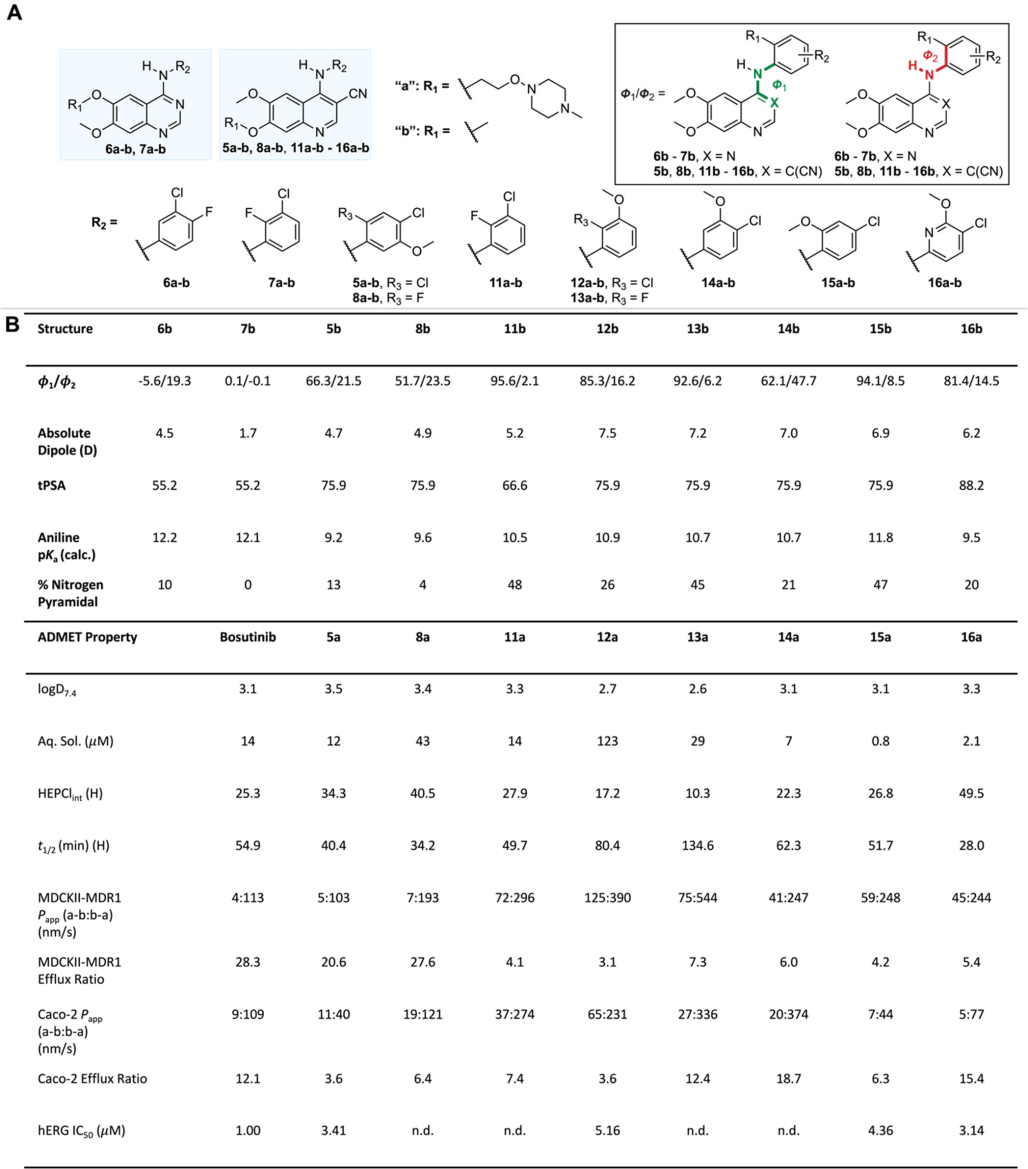
Computational analysis and early absorption, distribution, metabolism, excretion and toxicity (ADMET) profile of the inhibitors. A) Chemical structures of the hydroxylamine-bearing EGFR inhibitors and kinase inhibitors used in this study and definitions of torsional angles ϕ_1_ and ϕ_2_. B) Computational results on truncated derivatives. See Experimental Methods for details on computations. *In vitro* absorption, distribution, metabolism, excretion and toxicity (ADMET) properties of the inhibitors. Values represent the mean of *n* = 2 independent replicates unless otherwise stated. ADMET values for bosutinib and **5a** are from ref (34). Abbreviations: H, human; tPSA, topological polar surface area; calc., calculated; HEPCl_int_, intrinsic clearance in hepatocytes; *t*_1/2_, half-life; MDCKII, Madine-Darby canine-kidney; MDR1, multidrug resistance 1 (or P-glycoprotein); *P*_app_, apparent permeability; hERG, human ether-à-go-go-related gene.

In an attempt to understand the influence on P-gp efflux ratio wrought by the *ortho*-fluorine atom we used density functional theory (DFT) computations on truncated derivatives (**5b**–**8b**) (see Supporting Information for details).^44^ This consisted of computing the overall dipole moments, the torsional angles ϕ_1_ and ϕ_2_, which define the relative orientation of the two aromatic systems, and the p*K*_a_ (calculated p*K*_a_ or cp*K*_a_ in H_2_O)^45^ of the aniline N–H (Figure 3). Examining first the quinazoline pair **6b** and **7b**, the ϕ_1_ torsional angle was not influenced by the nature of the *ortho*-substituent in the aniline ring, while ϕ_2_ changed from ∼20° for **6b** (bearing an *ortho*-hydrogen) to ∼0° for **7b** (bearing an *ortho*-fluorine) (Figure 3B). This change in overall conformation is reflected by a significant reduction in molecular dipole (Δμ = −2.8 D), which is presumably the root cause of the reduced P-gp efflux ratio, as no significant change in cp*K*_a_ was seen between the two isomers (**6b** and **7b**) (Figure 3B). The coplanar arrangement of the aniline ring with the quinazoline core in **7b** is presumably stabilized by the antiparallel dipoles of the eclipsed N–H and C–F bonds as no evidence for a N–H–F HB was seen in the NMR studies (*vide supra*) (Figure 2B; Figure 3B). Finally, in both **6b** and **7b**, the anilino nitrogen is essentially planar with sums of bond angles at nitrogen of 357° and 360°, respectively.^46^

Turning to the 4-anilinoquinoline-3-carbonitrile pair **5b** and **8b**, the impact of the nitrile group on molecular structure is apparent in their ϕ_1_ torsional angles of ∼66° and 52°, as compared to the ∼0° in **6b** and **7b** (Figure 3B). This change in ϕ_1_ from the 4-anilinoquinazoline to 4-anilinoquinoline-3-carbonitrile series is accompanied by a significant substituent-dependent pyramidalization of the anilino nitrogen atom, as reflected in the sum of its bond angles.^46^ These changes in the ϕ_1_ torsion angle and hybridization at nitrogen clearly arise from steric destabilization of the planar conformation by the nitrile group. With respect to ϕ_2_ in **5b** and **8b**, the *ortho*-halogens are 20° out of plane with the N–H reflecting the greater stabilization of the antiparallel N–H C–X dipole pair for **5b** owing to the greater magnitude of the Ar–Cl dipole compared to the Ar–F dipole.^47,48^ The changes in individual substituent dipoles (Ar–Cl vs Ar–F) between **5b** and **8b** result in a small increase in molecular dipole which presumably underlies the reduced P-gp efflux ratio seen with **5a** in relation to **8a**. Moving forward, we carried out analogous DFT computations on the model compounds **11b**–**16b** to probe the effect of substituents on the pendant anilino ring in the 4-anilinoquinoline-3-carbonitrile series (Figure 3B).^44^

The presence of the nitrile group dramatically impacts ϕ_1_ with all such compounds (**11b**–**16b**) having 62° ≤ ϕ_1_ ≤ 96° for obvious steric reasons, with the smaller ϕ_1_ values found in those compounds lacking the *ortho*-substituents in the aniline ring or for compounds having a 2,4,5-trisubstituted aniline system (Figure 3B). Conversely, ϕ_2_ is a measure of the coplanarity of the *ortho*-substituent of the aniline ring with the N–H bond and shows variation according to substituents. For **11b**, which bears the identical 3-chloro-2-fluoro substitution pattern to **7b**,^33^ the *ortho*-fluorine is again roughly planar (ϕ_2_ = 2.1°) with the N–H while the aniline ring is approximately orthogonal to the quinoline (ϕ_1_ = 95.6°) as compared to a ϕ_1_ of ∼0° in **6b** and **7b**. Compounds **12b** and **13b** which bear a *meta*-methoxy and either an *ortho*-chlorine (**12b**) or *ortho*-fluorine (**13b**) atom, show roughly orthogonal relationships of the aniline ring and quinoline rings (ϕ_1_ ∼ 90°). Again, the *ortho*-fluorine (**13b**) adopts a roughly planar orientation with the N–H (ϕ_2_ = 6.2°), and the *ortho*-chlorine (**12b**) was 16° out of plane with the N–H (ϕ_2_ = 16.2°). In the absence of an *ortho*-substituent, as in **14b**, a considerable change in ϕ_1_ was observed (ϕ_1_ = 62.1°) and the *ortho*-hydrogen adopted an approximately 50° out of plane relationship with the N–H. With compound **15b**, which bears an *ortho*-methoxy group, the aniline ring again takes up an approximately orthogonal orientation to the quinoline ring (ϕ_1_ = 94.1°) while the *ortho*-methoxy substituent is 9° out of plane with the N–H, resulting in a conformation similar to that observed with *ortho*-fluorine-bearing compounds (**11b** and **13b**). Finally, we computed **16b**, which bears a pyridylamine in place of the aniline in **14b**, again resulting in close to orthogonal orientation of the two aromatic rings (ϕ_1_ = 81.4°) but only a 15° distortion of the pyridine ring from the N–H plane. With regard to nitrogen pyramidalization, compounds bearing a higher number of electron-withdrawing substituents generally had anilino nitrogen atoms closer to planarity, with the most planar compounds in the 4-anilinoquinoline-3-carbonitrile series being **5b** and **8b**, each with three electron-withdrawing groups.^46^

With regard to molecular dipole (μ), all substitution patterns displayed an increased dipole relative to **5b** and **8b**, as expected based on the anticipated vectorial sums of the individual substituent dipoles (Figure 3B). The initially surprising difference between **12b** and **13b** (Δμ = −0.3 D) is rationalized by the reduced strength of the Ar–F dipole as compared to the Ar–Cl dipole.^48^ Most importantly, introduction of the heteroatom in the form of the pyridine **16b** results in a reduction in overall dipole relative to the corresponding benzene derivative (**14b**) (Δμ = −0.8 D) presumably due to dipole cancellation between the nitrile and pyridyl moieties. The considerable difference in dipole moments in the two isomeric 4-anilinoquinazoline models **6b** and **7b** contrasts with the unchanged tPSA of the two molecules. This points to the limitations of such simplified parameters that do not take into account the overall molecular structure and shape to predicting physical and absorption, distribution, metabolism, excretion and toxicity (ADMET) properties.^49^ This conclusion is confirmed by inspection of the 4-anilinoquinoline-3-carbonitrile series of models **11b**–**16b** and is especially emphasized by comparison of **14b** and **16b**, where the latter, with the additional heteroatom, necessarily has the greater tPSA but because of cancellation of dipoles, is in fact, less polar. Finally, we calculated cp*K*_a_ (in H_2_O)^45^ for the entire series of models **5b**–**8b** and **11b**–**16b** as a proxy for HB donating ability and find that these follow the expected pattern with electron-withdrawing groups *ortho* or *para* to the anilino nitrogen having greater impact than comparable *meta*-substituents, with the most acidic compounds being **5b** and **8b** each with three electron-withdrawing substituents and **16b** with the electron-deficient heteroaromatic ring (Figure 3b).

**Figure 4:**
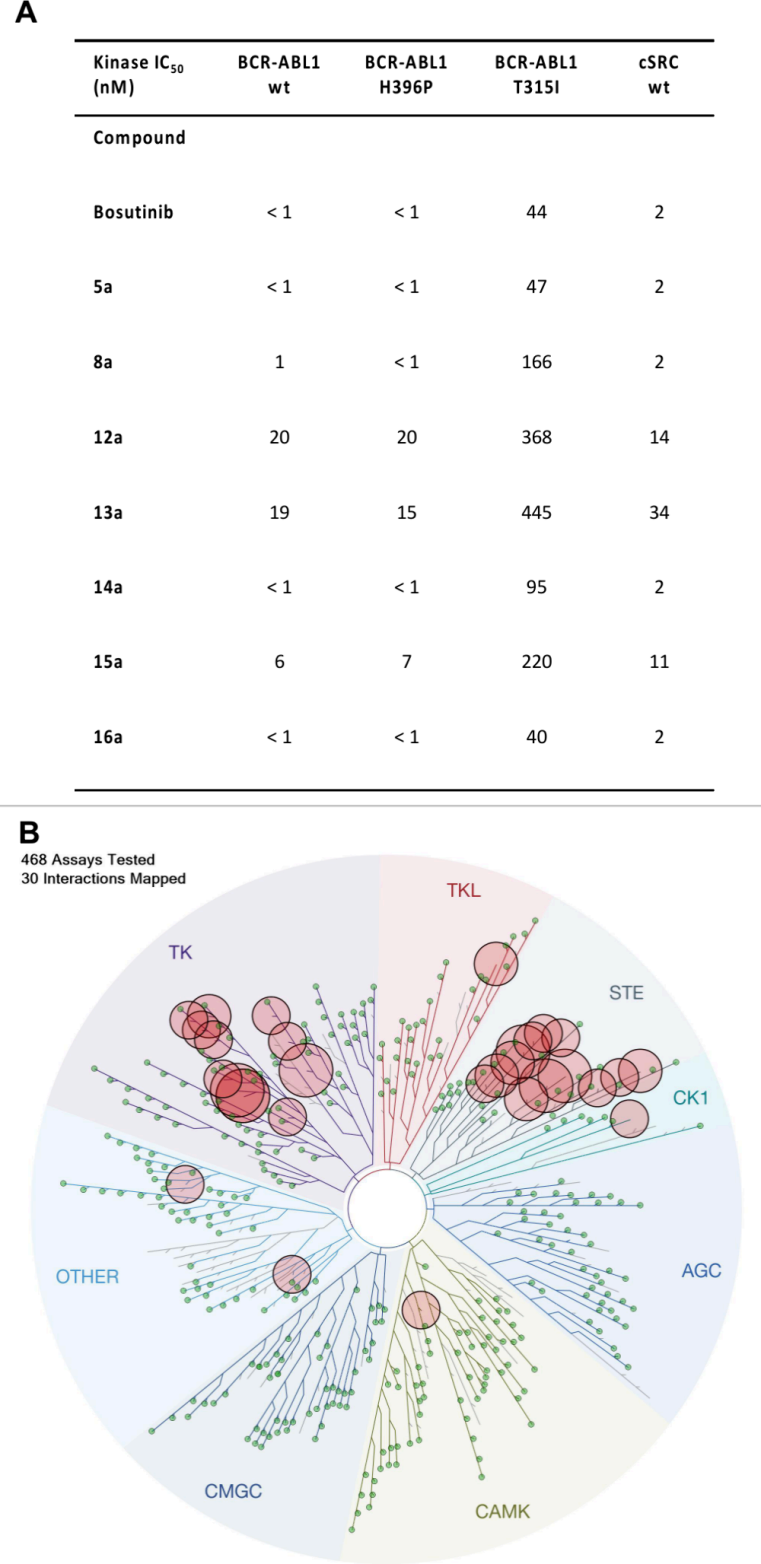
Kinase activity and kinase selectivity profile for **16a**. A) Kinase activity (IC_50_) against mutant BCR-ABL1 and cSRC. Values represent the mean of *n* = 2 independent replicates. Full kinase activity on inhibitors (bosutinib, **5a**, **8a**, **12a**-**16a**) is provided in the Supporting Information. Values for bosutinib and **5a** are from ref (34). B) KINOMEScan nonmutant or nonlipid kinase screening results of **16a** at a screening concentration of 1 μM. The size of the circles mapped onto the kinase phylogenetic tree using DiscoverX TREEspot corresponds to the strength of binding affinity. The plot is a representation of the S(1) score of 0.074 (30/403 nonmutant kinases showing ≤1% activity at 1 μM). Full KINOMEScan results are listed in the Supporting Information.

Armed with this insight into molecular properties, we synthesized the corresponding kinase inhibitors (**11a**–**16a**) and determined their early ADMET parameters (Figures 3) and their ability to inhibit mutant BCR-ABL1 and cSRC (*vide infra*). Interestingly, **12a**, which had the highest cp*K*_a_ value for the 2,3-disubstituted aniline derivatives, displayed a 31-fold enhancement in permeability and a greatly reduced efflux ratio in MDCKII-MDR1 cells relative to bosutinib, thereby highlighting the utility of decreasing hydrogen bond acidity to improve P-gp permeability (Figure 3B). Moreover, **12a** exhibited high aqueous solubility at pH 7.4, good stability in human hepatocytes and minimal hERG inhibition (IC_50_ = 5.16 μM). With regards to kinase inhibition, however, **12a** and **13a** displayed comparatively poor activity against mutant BCR-ABL1 kinases with IC_50_ values ≥15 nM (Figure 4A). This result is consistent with previous structure activity relationships (SAR) that have established the importance of *para*-substituents for cSRC and BCR-ABL1 kinase activity (Figure 4A).^50,51^ Thus, we focused our attention on **14a** and **15a** which bear the 4-chloro substituent, but whose core structures **14b** and **15b** exhibit markedly increased cp*K*_a_ values relative to bosutinib. Fortunately, the increased cp*K*_a_ values again translated to both improved permeability and reduced efflux ratios in MDCKII-MDR1 cells and both compounds (**14a** and **15a**) displayed good stability in human hepatocytes. Finally, we assayed compound **16a**, which bears the pyridylamine moiety, and observed excellent activity against both BCR-ABL1 and cSRC kinases, markedly improved permeability and a reduced efflux ratio in MDCKII-MDR1 cells relative to bosutinib. We note that while **16a** necessarily has a lower cp*K*_a_ than previous derivatives due to the inductive effect of the heterocycle, the increase in hydrogen bond acidity is offset by a corresponding decrease in absolute dipole leading to a compound with a net improvement in P-gp-mediated efflux ratio.

Although efflux by P-gp is considered the main mode of off-target resistance in MDR CML,^8−17^ it was of interest to determine if the increased permeability and improvement in P-gp efflux ratios wrought by modification of the pendant anilino moiety extend to other transporter proteins, such as breast cancer resistance protein (BCRP; ABCG2) (Figure 3B).^52,53^ To this end, we determined the permeability of all new compounds (**8a**, **11a**–**16a**) in Caco-2 cells, which express the three major efflux transporters: ABCB1, ABCC2, and ABCG2. No obvious trend was observed, with several compounds exhibiting decreased Caco-2 efflux ratios relative to bosutinib and some increased. In the 4-anilinoquinoline-3-carbonitrile series therefore, optimization of the substituent pattern around the aniline ring to minimize the MDCKII-MDR1 efflux ratio does not translate into a corresponding reduction in Caco-2 efflux ratio.

**Figure 5:**
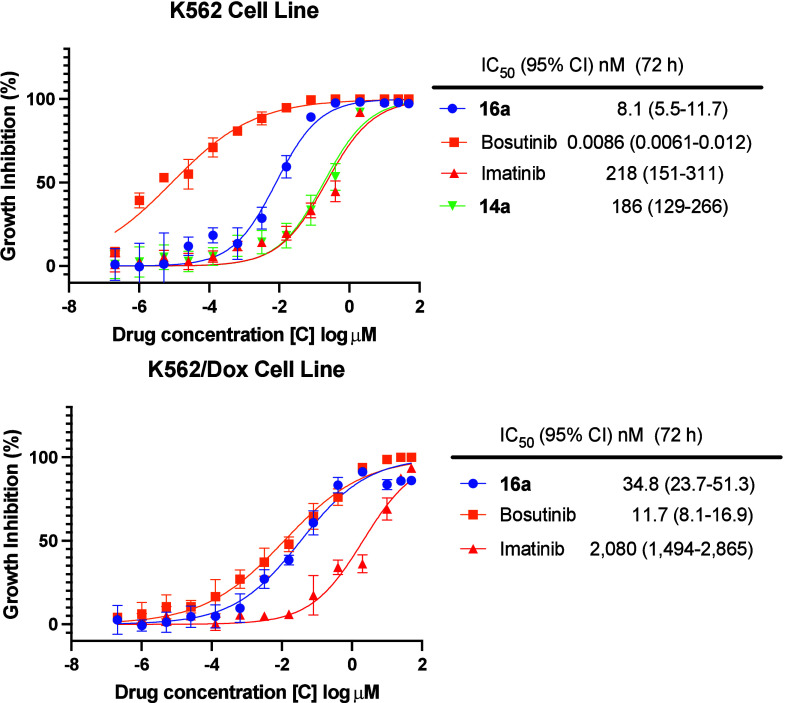
Antiproliferative activity of **14a** and **16a**. Compound **16a** displays potent antiproliferative activity in the patient-derived Philadelphia chromosome positive (Ph+) leukemia cell line K562 and exhibits only moderate resistance in doxorubicin pretreated K562 cells (K562/Dox) which overexpress P-glycoprotein. For all antiproliferative assays, points indicate mean, and error bars indicate SD; *n* = 3 independent replicates; IC_50_ values (nM) are reported beside the dose–response curve and represent the mean (95% confidence interval). IC_50_ values are unadjusted for FBS.

Taking kinase activity and efflux ratio in MDCKII-MDR1 cells into consideration, we profiled **14a** and **16a** in a Ph+ patient-derived leukemia cell line, K562 (Figure 5). Imatinib and **14a** both had activity in the low triple-digit nanomolar range (IC_50_ values of 186 nM and 218 nM), while **16a** with an IC_50_ of 8.1 nM was closer to the picomolar activity of bosutinib, leading us to focus subsequent studies on **16a**. In additional ADMET profiling, **16a** displayed a pH-dependent solubility profile with excellent solubility below pH 6.5, good stability in human and mouse hepatocytes and high human and mouse plasma protein binding (see Supporting Information for details). With regard to potential toxicity, **16a** showed only moderate inhibition of the hERG potassium ion channel with an IC_50_ value of 3.14 μM and a maximal inhibition of approximately 20% at 1 μM (Figure 3B). The cytochrome P450 (CYP) inhibitory activity of **16a** was then assayed across four major isoforms (3A4, 1A2, 2D6, 2C9) resulting in IC_50_’s > 10 μM except for CYP3A4 against which **16a** had an IC_50_ of 1.0 μM (see Supporting Information for details). Fortunately, **16a** was negative in a follow-up time-dependent inhibition (TDI) IC_50_-shift experiment in human liver microsomes against CYP3A4, thereby dispelling concerns of potential drug–drug interactions (DDI).^54^ Using KINOMEScan technology,^55^ we next determined the selectivity of **16a** across a panel of >400 human kinases at a concentration of 1 μM, and observed moderate kinase selectivity with an S(1) score of 0.074 (30/403 nonmutant kinases showing ≤1% activity at 1 μM) (Figure 4B). In addition to BCR-ABL1, ABL2 and cSRC, **16a** showed high affinity (≤0.1% activity at 1 μM) for the STE family of kinases, specifically the STE20 subfamily, which are well-known^56^ additional kinase targets for the parent molecule, bosutinib (**2**). In addition to the STE family of kinases, **16a** showed high affinity (≤0.1% activity at 1 μM) for ERBB3 (HER3) (0% activity at 1 μM). Like bosutinib, **16a** showed minimal affinity for mast/stem cell (KIT) or platelet-derived growth factor receptors (PDGFR) as compared to BCR-ABL1, cSRC and the STE20 subfamily of kinases (see Supporting Information for details).^56^

Finally, we determined the antiproliferative activity of **16a** in patient-derived K562 cells pretreated with the chemotherapeutic doxorubicin (Dox), which leads to an MDR phenotype characterized by P-gp overexpression (Figure 5).^8,9,57^ Gratifyingly, we observed only a minimally increased IC_50_ value of 34.8 nM, representing an approximately 4-fold shift for resistance as compared to wild-type K562 cells. In contrast, imatinib and bosutinib, both potent P-gp substrates, had IC_50_ values of 2,080 nM and 11.7 nM, respectively, in the K562/Dox cells representing approximately 10-fold and 1360-fold resistance, respectively. Overall, **16a** exhibits a >10-fold improvement in permeability and a markedly reduced efflux ratio in MDCKII-MDR1 cells relative to bosutinib and is a potent inhibitor in imatinib-resistant and bosutinib-resistant patient-derived Ph+ leukemia cells that overexpress P-gp (K562/Dox cells).

## Chemistry

**Figure 6:**
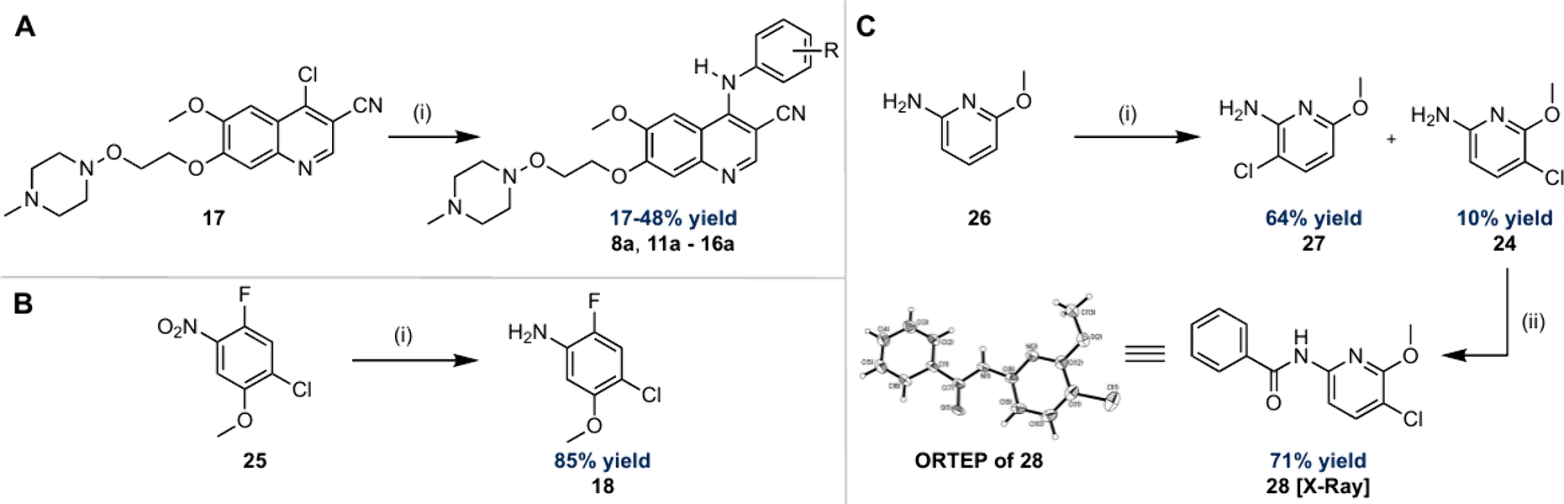
Chemical synthesis of the hydroxylamine-bearing BCR-ABL1 inhibitors (**8a**, **11a**–**16a**) and anilines (**18**, **24**). A) Chemical synthesis of inhibitors (**8a**, **11a**–**16a**) following a literature procedure.^34^ (i) Pyridine-hydrochloride (pyr·HCl), aniline, 2-ethoxyethanol, 135 °C, 6 h −14 h. B) Chemical synthesis of aniline (**18**). (i) H_2_ (1 atm), Pd/C, dioxane, r.t., 20 h. (C) Chemical synthesis of aniline (**24**) by modification of a literature procedure.^58^ Reagents and conditions: (i) *N*-chlorosuccinimide (NCS), DCM, 0 °C to r.t., 2 h. (ii) Benzoyl chloride (BzCl), 4-(dimethylamino)pyridine (DMAP), pyridine, DCM, 0 °C to r.t., 12 h.

The synthesis of all inhibitors was carried out in an analogous fashion to that previously reported for preparation of **5a** (Figure 6A).^34^ To this end, a nucleophilic aromatic substitution (S_N_Ar) reaction with the substituted anilines (**18**–**24**) under acidic conditions with quinoline (**17**) gave the desired inhibitors in 17–48% yield (Figure 6A). The anilines (**18**–**24**) were commercially available except for **18** and **24**. Consequently, aniline **18** was prepared in 85% yield by hydrogenation of the corresponding nitro derivative (**25**) over palladium on carbon (Pd/C), while aniline **24** was prepared by chlorination of **26** with *N*-chlorosuccinimide (NCS) to give a separable mixture of regioisomers (**27** and **24**) in 64 and 10% yields, respectively (Figure 6B,C).^58^ The regiochemistry of the chlorination was confirmed through a series of 1D-NOESY experiments and was proved for **24** after conversion to the corresponding benzamide derivative (**28**) and single crystal X-ray analysis (see Supporting Information for details) (Figure 6C).

## Conclusions

In summary, a hydroxylamine-bearing BCR-ABL1 kinase inhibitor with a reduced P-gp efflux ratio and potent activity in a MDR patient-derived leukemia cell line overexpressing P-gp (K562/Dox), compound **16a**, has been developed. While bosutinib exhibits higher activity in K562 cells over **16a**, the latter displays an over 300-fold improvement in relative resistance in MDR K562/Dox cells and an approximately 60-fold improvement in activity compared to imatinib. Key to the discovery of **16a** was a theoretical and experimental investigation on substituent effects in the pendant anilino moiety that influenced P-gp efflux ratios. In a series of isomeric 4-anilinoquinazoline-based kinase inhibitors, a dipole-canceling hypothesis to reduce P-gp efflux ratios, is proposed. Conversely, in the 4-anilinoquinoline-3-carbonitrile series, steric destabilization of the planar conformation by the nitrile substituent leads to a combination of changes in HB donor acidity and molecular dipoles, which in turn influence P-gp efflux ratios. Overall, we show that P-gp efflux ratios can be optimized without compromising biological activity and without evidence of any intramolecular hydrogen bonding. We find that tPSA is not a useful parameter for the estimation of polarity in the compounds studied owing to their changing conformations (and sometimes regioisomeric natures) but may be replaced by computed molecular dipoles. The current study underscores the value of investigating physical organic phenomena such as dipole moments and/or hydrogen bond acidity to influence P-gp efflux ratios, in optimization campaigns where strategic deployment of intramolecular hydrogen bonds to reduce P-gp efflux ratios is not viable. We anticipate that the results described herein could aid future inhibitor design targeting MDR phenotypes characterized by P-gp overexpression, or more broadly, in discovery campaigns where P-gp efflux poses a significant challenge such as in central nervous system (CNS) drug discovery.

## Experimental Methods

### Chemistry

#### General Experiments and Information

All reactions were conducted in single-neck, oven-dried glassware fitted with a rubber-septa under an argon atmosphere unless otherwise stated. All organic solutions were concentrated under reduced pressure on a rotary evaporator and water bath. Flash-column chromatography was performed using silica gel (Fisher Silica Gel Sorbent (230–400 Mesh, grade 60)).^59^ Thin-layer chromatography (TLC) was carried out with 250 μM glass back silica (XHL) plates with fluorescent indicator (254 nm). TLC plates were visualized by exposure to ultraviolet light (UV) and/or submersion in ceric ammonium molybdate (CAM) in ethanol followed by heating on a hot plate (120 °C, 10–15 s). Solvents were purchased from Sigma-Aldrich and used without further purification. 4-Chloro-6-methoxy-7-(2-((4-methylpiperazin-1-yl)oxy)ethoxy)quinoline-3-carbonitrile (**17**) was prepared according to a literature procedure^34^ and had spectral data in accord with that previously reported. Pyridine hydrochloride was purchased from Sigma-Aldrich. Palladium on carbon was purchased from Sigma-Aldrich. 1-Chloro-5-fluoro-2-methoxy-4-nitrobenzene (**25**) was purchased from Sigma-Aldrich. 6-Methoxypyridin-2-amine (**26**) was purchased from Ambeed. *N*-Chlorosuccinimide was purchased from Sigma-Aldrich. Benzoyl chloride was purchased from Sigma-Aldrich. 4-(Dimethylamino)pyridine was purchased from Sigma-Aldrich. Pyridine was purchased from OakWood Chemicals. NMR spectra of all compounds were obtained in CDCl_3_ (δ_H_ 7.26 and δ_C_ 77.16 ppm, respectively) or DMSO-*d*_6_ (δ_H_ 2.50 and δ_C_ 39.52 ppm, respectively) using a 500 MHz, EZC500 JEOL instrument at 298 K unless otherwise specified. The chemical shifts (δ) are calculated with respect to residual solvent peak and are given in ppm. Multiplicities are abbreviated as followed: s (singlet), m (multiplet), b (broad), d (doublet), t (triplet), q (quartet). High-resolution mass spectra (HRMS) were obtained on a ThermoFisher Orbitrap Q-Exactive instrument using electrospray ionization (ESI). Copies of HRMS spectra of compounds **8a**, **11a**–**16a** are provided on pages S50–S56 of the Supporting Information. UHPLC traces of compounds **8a**, **11a**–**16a** were obtained using a ThermoFisher Vanquish UHPLC with PDA detector and an Acclaim 120 ^18^C 4.6 × 50 mm column, and the %purity was determined using the Avalon peak area algorithm. Purities of all final compounds were >95% as determined by UHPLC. UHPLC conditions and copies of UHPLC traces for **8a**, **11a**–**16a** are given in the catalog of spectra section on pages S133–S139 of the Supporting Information. Melting points of solid compounds were obtained on a Barstead Electrothermal 9100. The purity of commercial imatinib (**1**) and bosutinib (**2**) was confirmed by NMR and HRMS prior to *in vitro* study initiation.

#### 4-Chloro-2-fluoro-5-methoxyaniline (**18**)

To a stirred solution of 1-chloro-5-fluoro-2-methoxy-4-nitrobenzene (**25**) (2.5 g, 12.16 mmol, 1.0 equiv) in dioxane at r.t. was added Pd/C (10% by wt) (250 mg), and the solution was evacuated of air and purged 3x with H_2_ (g) (1 atm, balloon), after which H_2_ (g) (1 atm) was allowed to flow and the reaction was stirred for 20 h at r.t. After such time, the mixture was filtered over Celite and the solid washed with EtOAc (50 mL). The resulting filtrate was concentrated and the residue obtained was purified by flash column chromatography on silica (eluent: 25:75 EtOAc:hexanes) to afford the title compound **18** (1.82 g, 10.40 mmol, 85%) as a light purple solid. TLC R_f_ = 0.10 (25:75 EtOAc:hexanes; UV, CAM). ^1^H NMR (500 MHz, CDCl_3_) δ 7.01 (d, *J* = 10.3 Hz, 1H), 6.35 (d, *J* = 8.0 Hz, 1H), 3.80 (s, 3H), 3.73 (br s, 2H). ^13^C NMR (126 MHz, CDCl_3_) δ 151.9 (d, ^4^*J*_C–F_ = 2.5 Hz), 145.4 (d, ^1^*J*_C–F_ = 235.6 Hz), 133.9 (d, ^2^*J*_C–F_ = 13.9 Hz), 117.0 (d, ^2^*J*_C–F_ = 23.9 Hz), 110.3 (d, ^3^*J*_C–F_ = 10.1 Hz), 101.2 (d, ^3^*J*_C–F_ = 3.8 Hz), 56.7. ^13^C {^19^F} NMR (126 MHz, CDCl_3_) δ 151.9, 145.4, 133.9, 117.0, 110.3, 101.2, 56.7. ^19^F {^1^H} NMR (470 MHz, CDCl_3_) δ −143.1. HRMS-ESI (*m*/*z*): [M + H]^+^ calculated for [C_7_H_8_NO^35^ClF]^+^: 176.0273, found: 176.0271. mp 49.2–51.5 °C (mean of *n* = 3 determinations).

#### 3-Chloro-6-methoxypyridin-2-amine (27) and 5-chloro-6-methoxypyridin-2-amine (**24**)

To a stirred solution of **26** (10 g, 80.55 mmol, 1.0 equiv) in DCM at 0 °C was added *N*-chlorosuccinimide (11.83 g, 88.61 mmol, 1.1 equiv) portion-wise over 10 min. After full addition, the reaction was stirred for 1 h at 0 °C, then warmed to r.t. and stirred for an additional 1 h. After such time, NaHCO_3_ (200 mL) was added and the layers separated. The organic layer was washed with NaHCO_3_ (2x, 200 mL), dried over Na_2_SO_4_, filtered and concentrated *in vacuo*. The residue obtained was purified by flash column chromatography on silica (eluent: 20:80 EtOAc:hexanes) to afford the title compounds as a separable mixture (9.39 g, 59.4 mmol, 74% overall yield; 64% of 3-chloro-6-methoxypyridin-2-amine (**27**) and 10% of 5-chloro-6-methoxypyridin-2-amine (**24**)). Spectral data are in accord with the literature.^58^ 1D-NOESY studies (outlined on pages S65–S67 and S71–S73 of the Supporting Information) were carried out to support regiochemical assignments. Additionally, **24** was converted to the corresponding benzamide (**28**) to prove the regiochemistry of chlorination. Characterization data for **27**: Physical state: brown oil. TLC R_f_*=* 0.50 (20:80 EtOAc:hexanes; CAM, UV). ^1^H NMR (500 MHz, CDCl_3_) δ 7.34 (d, *J* = 8.4 Hz, 1H), 6.07 (d, *J* = 8.4 Hz, 1H), 4.72 (br s, 2H), 3.82 (s, 3H). ^13^C NMR (126 MHz, CDCl_3_) δ 162.2, 152.8, 139.5, 105.3, 100.2, 53.8. Characterization data for **24**: Physical state: Thick purple oil. TLC R_f_ = 0.40 (20:80 EtOAc:hexanes; CAM, UV). ^1^H NMR (500 MHz, CDCl_3_) δ 7.33 (d, *J* = 8.2 Hz, 1H), 6.01 (d, *J* = 8.2 Hz, 1H), 4.32 (br s, 2H), 3.92 (s, 3H). ^13^C NMR (126 MHz, CDCl_3_) δ 158.3, 155.5, 139.9, 105.4, 100.6, 54.0. HRMS-ESI (*m*/*z*): [M + H]^+^ calculated for [C_6_H_8_ClN_2_O]^+^: 159.0319, found: 159.0315.

#### N-5-(Chloro-6-methoxypyridin-2-yl)benzamide (**28**)

To a stirred solution of **24** (320 mg, 2.77 mmol, 1.0 equiv) in anhydrous DCM (14 mL) was added DMAP (33.8 mg, 0.277 mmol, 0.1 equiv) followed by pyridine (445 μL, 5.53 mmol, 2.0 equiv) and BzCl (322 μL, 2.77 mmol, 1.0 equiv). The reaction was stirred for 12 h at r.t. After such time, the mixture was quenched by addition of NaHCO_3_ (25 mL) and the layers separated. The organic layer was further washed with NaHCO_3_ (2x, 25 mL), dried over Na_2_SO_4_, filtered and concentrated *in vacuo*. The residue obtained was purified by flash column chromatography on silica (eluent: 20:80 EtOAc:hexanes) to afford the title compound **28** (526 mg, 1.98 mmol, 71%) as a light purple solid. TLC R_f_ = 0.50 (20:80 EtOAc:hexanes; CAM, UV). ^1^H NMR (500 MHz, CDCl_3_) δ 8.31 (s, 1H), 7.92–7.90 (m, 3H), 7.66 (d, *J* = 8.4 Hz, 1H), 7.58 (m, 1H), 7.59–7.49 (m, 2H), 3.97 (s, 3H). ^13^C NMR (126 MHz, CDCl_3_) δ 165.5, 158.0, 147.4, 140.5, 134.3, 132.5, 129.0, 127.3, 112.5, 106.7, 54.5. HRMS-ESI (*m*/*z*): [M + H]^+^ calculated for [C_13_H_12_O_2_N_2_^35^Cl]^+^: 263.0582, found: 263.0574. The so obtained light purple solid was crystallized from hot EtOAc to obtain colorless crystals suitable for X-ray crystallographic analysis (see details on X-ray crystallography on pages S140–S141 of the Supporting Information). mp 122.7–124.4 °C (mean of *n* = 3 determinations).

#### 4-((4-Chloro-2-fluoro-5-methoxyphenyl)amino)-6-methoxy-7-(2-((4-methylpiperazin-1-yl)oxy)ethoxy)quinoline-3-carbonitrile (**8a**)

To a stirred solution of **17** (890 mg, 2.37 mmol, 1.0 equiv) in 2-ethoxyethanol (12 mL) at r.t. was added pyridine·HCl (547 mg, 4.74 mmol, 2.0 equiv) followed by 4-chloro-2-fluoro-5-methoxyaniline (**18**) (658 mg, 3.56 mmol, 1.5 equiv). The solution was then brought to reflux (135 °C) and stirred for 10 h. After such time, the solvent was removed *in vacuo* and the resulting residue coconcentrated with toluene (3x, 20 mL) to remove remaining 2-ethoxyethanol. The residue obtained was dissolved in EtOAc (75 mL) and washed with saturated aq. K_2_CO_3_ (2x, 75 mL) and the organic layers combined, dried over Na_2_SO_4_, filtered and concentrated *in vacuo*. The resulting residue was purified by flash column chromatography on silica (eluent: 85:10:5 EtOAc:MeOH:Et_3_N) to afford the title compound (**8a**) (432 mg, 0.837 mmol, 35%) as a yellow solid.* TLC R_f_ = 0.30 (85:10:5 EtOAc:MeOH:Et_3_N; CAM, UV). ^1^H NMR (500 MHz, CDCl_3_) δ 8.66 (s, 1H), 7.42 (s, 1H), 7.24 (s, 1H), 6.95 (s, 1H), 6.68 (s, 1H), 6.58 (d, *J* = 7.2 Hz, 1H), 4.34 (t, *J* = 5.0 Hz, 2H), 4.14 (t, *J* = 5.0 Hz, 2H), 3.77 (s, 3H), 3.72 (s, 3H), 3.22–3.20 (m, 2H), 2.85–2.68 (m, 4H), 2.27–2.22 (m, 5H). ^13^C NMR (126 MHz, CDCl_3_) δ 153.9, 151.9 (d, ^4^*J*_C–F_ = 1.3 Hz), 150.4, 150.0, 149.1 (d, ^1^*J*_C–F_ = 241.9 Hz), 148.1, 147.6, 127.5 (d, ^2^*J*_C–F_ = 13.9 Hz), 118.8 (d, ^3^*J*_C–F_ = 10.1 Hz), 118.1 (d, ^2^*J*_C–F_ = 23.9 Hz), 116.7, 114.5, 110.2, 107.1, 101.1, 93.0, 69.4, 67.5, 57.0, 56.2, 55.5, 54.2, 45.6. ^13^C {^19^F} NMR (126 MHz, CDCl_3_) δ 153.9, 151.9, 150.4, 150.0, 149.1, 148.1, 147.6, 127.5, 118.8, 118.1, 116.7, 114.5, 110.2, 107.1, 101.1, 93.0, 69.4, 67.5, 57.0, 56.2, 55.5, 45.6. ^19^F {^1^H} NMR (470 MHz, CDCl_3_) δ - 133.5. HRMS-ESI (*m*/*z*): [M + H]^+^ calculated for [C_25_H_28_O_4_N_5_^35^ClF]^+^: 516.1808, found: 516.1805. [*To ensure no palladium contamination had been carried through from the synthesis of aniline (**18**), **8a** was screened by ICP-MS at the University of Georgia’s Plasma Chemistry Laboratory in the Center for Applied Isotope Studies and was found to contain <0.009 μg/g of ^105^Pd prior to initiation of biological tests. mp 203.6–204.9 °C (mean of *n* = 3 determinations).]

#### 4-((3-Chloro-2-fluorophenyl)amino)-6-methoxy-7-(2-((4-methylpiperazin-1-yl)oxy)ethoxy)quinoline-3-carbonitrile (**11a**)

To a stirred solution of **17** (1.05 g, 2.79 mmol, 1.0 equiv) in 2-ethoxyethanol (15 mL) at r.t. was added pyridine·HCl (645 mg, 5.58 mmol, 2.0 equiv) followed by 3-chloro-2-fluoroaniline (**19**) (461 μL, 4.19 mmol, 1.5 equiv). The solution was then brought to reflux (135 °C) and stirred for 10 h. After such time, the solvent was removed *in vacuo* and the resulting residue coconcentrated with toluene (3x, 25 mL) to remove remaining 2-ethoxyethanol. The residue obtained was dissolved in EtOAc (75 mL) and washed with saturated aq. K_2_CO_3_ (2x, 75 mL) and the organic layers combined, dried over Na_2_SO_4_, filtered and concentrated *in vacuo*. The resulting residue was purified by flash column chromatography on silica (eluent: 90:5:5 EtOAc:MeOH:Et_3_N) to afford the title compound (**11a**) (235 mg, 0.484 mmol, 17%) as a light yellow solid. TLC R_f_ = 0.30 (90:5:5 EtOAc:MeOH:Et_3_N; CAM, UV). ^1^H NMR (500 MHz, CDCl_3_) δ 8.62 (s, 1H), 7.38 (s, 1H), 7.15 (t, *J* = 7.4 Hz, 1H), 7.06 (s, 1H), 7.01–6.97 (m, 2H), 6.88 (t, *J* = 7.7 Hz, 1H), 4.31 (t, *J* = 4.9 Hz, 2H), 4.11 (t, *J* = 4.9 Hz, 2H), 3.73 (s, 3H), 3.24–3.18 (m, 2H), 2.77–2.75 (m, 4H), 2.55–2.22 (m, 5H). ^13^C NMR (126 MHz, CDCl_3_) δ 153.9, 151.2 (d, ^1^*J*_C–F_ = 249.5 Hz), 150.4, 149.9, 148.2, 147.4, 130.3 (d, ^2^*J*_C–F_ = 11.3 Hz), 126.3, 124.4 (d, ^3^*J*_C–F_ = 5.0 Hz), 122.2 (d, ^2^*J*_C–F_ = 16.4 Hz), 121.2, 116.6, 114.9, 110.0, 101.4, 93.3, 69.4, 67.4, 56.1, 55.3, 54.0, 45.5. ^13^C {^19^F} NMR (126 MHz, CDCl_3_) δ 153.9, 151.2, 150.4, 149.9, 148.2, 147.4, 130.3, 126.3, 124.4, 122.2, 121.2, 116.6, 114.9, 110.0, 101.4, 93.4, 69.4, 67.4, 56.1, 55.3, 54.0, 45.5. ^19^F {^1^H} NMR (470 MHz, CDCl_3_) δ −127.6. HRMS-ESI (*m*/*z*): [M + H]^+^ calculated for [C_24_H_26_O_3_N_5_^35^ClF]^+^: 486.1703, found: 486.1688. mp 98.7–101.3 °C (mean of *n* = 3 determinations).

#### 4-((2-Chloro-3-methoxyphenyl)amino)-6-methoxy-7-(2-((4-methylpiperazin-1-yl)oxy)ethoxy)quinoline-3-carbonitrile (**12a**)

To a stirred solution of **17** (760 mg, 2.02 mmol, 1.0 equiv) in 2-ethoxyethanol (10 mL) at r.t. was added pyridine·HCl (467 mg, 4.04 mmol, 2.0 equiv) followed by 2-chloro-3-methoxyaniline (**20**) (478 mg, 3.03 mmol, 1.5 equiv). The solution was then brought to reflux (135 °C) and stirred for 10 h. After such time, the solvent was removed *in vacuo* and the resulting residue coconcentrated with toluene (3x, 50 mL) to remove remaining 2-ethoxyethanol. The residue obtained was dissolved in EtOAc (75 mL) and washed with saturated aq. K_2_CO_3_ (2x, 75 mL) and the organic layers combined, dried over Na_2_SO_4_, filtered and concentrated *in vacuo*. The resulting residue was purified by flash column chromatography on silica (eluent: 85:10:5 EtOAc:MeOH:Et_3_N) to afford the title compound (**12a**) (399 mg, 0.803 mmol, 40%) as an orange solid. TLC R_f_ = 0.50 (90:5:5 EtOAc:MeOH:Et_3_N; CAM, UV). ^1^H NMR (500 MHz, CDCl_3_) δ 8.68 (s, 1H), 7.41 (s, 1H), 7.11 (t, *J* = 8.3 Hz, 1H), 6.91 (s, 1H), 6.83 (s, 1H), 6.72 (d, *J* = 8.3 Hz, 1H), 6.51 (d, *J* = 8.1 Hz, 1H), 4.34 (t, *J* = 5.0 Hz, 2H), 4.13 (t, *J* = 5.0 Hz, 2H), 3.94 (s, 3H), 3.70 (s, 3H), 3.22–3.20 (m, 2H), 2.77–2.75 (m, 4H), 2.26–2.22 (m, 5H). ^13^C NMR (126 MHz, CDCl_3_) δ 156.2, 153.9, 150.2, 149.8, 148.3, 147.7, 139.3, 127.2, 116.6, 115.4, 114.2, 113.5, 110.0, 107.3, 102.0, 94.8, 69.4, 67.4, 56.6, 56.1, 55.5, 54.2, 45.6. HRMS-ESI (*m*/*z*): [M + H]^+^ calculated for [C_25_H_29_O_4_N_5_^35^Cl]^+^: 498.1903, found: 498.1891. mp 72.9–76.1 °C (mean of *n* = 3 determinations).

#### 4-((2-Fluoro-3-methoxyphenyl)amino)-6-methoxy-7-(2-((4-methylpiperazin-1-yl)oxy)ethoxy)quinoline-3-carbonitrile (**13a**)

To a stirred solution of **17** (400 mg, 1.06 mmol, 1.0 equiv) in 2-ethoxyethanol (6 mL) at r.t. was added pyridine·HCl (244 mg, 2.12 mmol, 2.0 equiv) followed by 2-fluoro-3-methoxyaniline (**21**) (226 mg, 1.60 mmol, 1.5 equiv). The solution was then brought to reflux (135 °C) and stirred for 10 h. After such time, the solvent was removed *in vacuo* and the resulting residue coconcentrated with toluene (3x, 50 mL) to remove remaining 2-ethoxyethanol. The residue obtained was dissolved in EtOAc (75 mL) and washed with saturated aq. K_2_CO_3_ (2x, 75 mL) and the organic layers combined, dried over Na_2_SO_4_, filtered and concentrated *in vacuo*. The resulting residue was purified by flash column chromatography on silica (eluent: 90:5:5 EtOAc:MeOH:Et_3_N) to afford the title compound (**13a**) (245 mg, 0.509 mmol, 48%) as a light orange solid. TLC R_f_ = 0.40 (90:5:5 EtOAc:MeOH:Et_3_N; CAM, UV). ^1^H NMR (500 MHz, CDCl_3_) δ 8.64 (s, 1H), 7.40 (s, 1H), 7.00 (t, *J* = 7.4 Hz, 1H), 6.96 (s, 1H), 6.79 (t, *J* = 8.0 Hz, 1H), 6.69 (s, 1H), 6.60 (t, *J* = 7.5 Hz, 1H), 4.34 (t, *J* = 5.0 Hz, 2H), 4.14 (t, *J* = 5.1 Hz, 2H), 3.92 (s, 3H), 3.70 (s, 3H), 3.27–3.16 (m, 2H), 2.88–2.62 (m, 4H), 2.27–2.23 (m, 5H). ^13^C NMR (126 MHz, CDCl_3_) δ 153.7, 150.1, 149.9, 148.75 (d, ^2^*J*_C–F_ = 10.8 Hz), 148.73, 147.6, 145.6 (d, ^1^*J*_C–F_ = 245.7 Hz), 129.8 (d, ^2^*J*_C–F_ = 8.8 Hz), 123.8 (d, ^3^*J*_C–F_ = 5.0 Hz), 116.8, 115.0, 114.6, 110.1, 109.7, 101.7, 93.2, 69.4, 67.4, 56.6, 56.0, 55.5, 54.2, 45.6. ^13^C {^19^F} NMR (126 MHz, CDCl_3_) δ 153.7, 150.1, 149.9, 148.8, 148.7, 147.6, 145.6, 129.8, 123.8, 116.7, 115.0, 114.6, 110.1, 109.7, 101.8, 93.2, 69.4, 67.4, 56.6, 56.0, 55.5, 54.2, 45.6. ^19^F {^1^H} NMR (470 MHz, CDCl_3_) −148.2. HRMS-ESI (*m*/*z*): [M + H]^+^ calculated for [C_25_H_29_O_4_N_5_F]^+^: 482.2198, found: 482.2196. mp 76.9–79.3 °C (mean of *n* = 3 determinations).

#### 4-((4-Chloro-3-methoxyphenyl)amino)-6-methoxy-7-(2-((4-methylpiperazin-1-yl)ethoxy)quinoline-3-carbonitrile (**14a**)

To a stirred solution of **17** (900 mg, 2.39 mmol, 1.0 equiv) in 2-ethoxyethanol (12 mL) at r.t. was added pyridine·HCl (552 mg, 4.78 mmol, 2.0 equiv) followed by 4-chloro-3-methoxyaniline (**22**) (566 mg, 3.59 mmol, 1.5 equiv). The solution was then brought to reflux (135 °C) and stirred for 10 h. After such time, the solvent was removed *in vacuo* and the resulting residue coconcentrated with toluene (3x, 20 mL) to remove remaining 2-ethoxyethanol. The residue obtained was dissolved in EtOAc (100 mL) and washed with saturated aq. K_2_CO_3_ (2x, 75 mL) and the organic layers combined, dried over Na_2_SO_4_, filtered and concentrated *in vacuo*. The resulting residue was purified by flash column chromatography on silica (eluent: 90:5:5 EtOAc:MeOH:Et_3_N) to afford the title compound (**14a**) (240 mg, 0.483 mmol, 20%) as a tan solid. TLC R_f_ = 0.25 (90:5:5 EtOAc:MeOH:Et_3_N; CAM, UV). ^1^H NMR (500 MHz, CDCl_3_) δ 8.63 (s, 1H), 7.37 (s, 1H), 7.29 (d, *J* = 8.4 Hz, 1H), 6.97 (s, 1H), 6.88 (s, 1H), 6.66 (d, *J* = 2.4 Hz, 1H), 6.58 (dd, *J* = 8.4, 2.4 Hz, 1H), 4.31 (t, *J* = 5.0 Hz, 2H), 4.12 (t, *J* = 4.9 Hz, 2H), 3.80 (s, 3H), 3.63 (s, 3H), 3.22–3.19 (m, 2H), 2.78–2.76 (m, 4H), 2.26 (br s, 5H). ^13^C NMR (126 MHz, CDCl_3_) δ 155.8, 153.7, 149.8, 148.9, 147.7, 140.7, 130.6, 118.9, 117.0, 114.8, 114.1, 110.0, 106.6, 102.5, 92.8, 69.4, 67.4, 56.4, 56.0, 55.4, 54.0, 45.5. HRMS-ESI (*m*/*z*): [M + H]^+^ calculated for [C_25_H_29_O_4_N_5_^35^Cl]^+^: 498.1903, found: 498.1906. mp 73.1–75.4 °C (mean of *n* = 3 determinations).

#### 4-((4-Chloro-2-methoxyphenyl)amino)-6-methoxy-7-(2-((4-methylpiperazin-1-yl)oxy)ethoxy)quinoline-3-carbonitrile (**15a**)

To a stirred solution of **17** (700 mg, 1.86 mmol, 1.0 equiv) in 2-ethoxyethanol (12 mL) at r.t. was added pyridine·HCl (430 mg, 3.72 mmol, 2.0 equiv) followed by 4-chloro-2-methoxyaniline (**23**) (440 mg, 2.79 mmol, 1.5 equiv). The solution was then brought to reflux (135 °C) and stirred for 14 h. After such time, the solvent was removed *in vacuo* and the resulting residue coconcentrated with toluene (3x, 50 mL) to remove remaining 2-ethoxyethanol. The residue obtained was dissolved in EtOAc (100 mL) and washed with saturated aq. K_2_CO_3_ (2x, 75 mL) and the organic layers combined, dried over Na_2_SO_4_, filtered and concentrated *in vacuo*. The resulting residue was purified by flash column chromatography on silica (eluent: 90:5:5 EtOAc:MeOH:Et_3_N) to afford the title compound (**15a**) (153 mg, 0.307 mmol, 17%) as a brown solid. TLC R_f_ = 0.40 (90:5:5 EtOAc:MeOH:Et_3_N; CAM, UV). ^1^H NMR (500 MHz, CDCl_3_) δ 8.62 (s, 1H), 7.39 (s, 1H), 6.98–6.92 (m, 2H), 6.87–6.85 (m, 1H), 6.81 (d, *J* = 8.1 Hz, 2H), 4.33 (t, *J* = 5.0 Hz, 2H), 4.14 (t, *J* = 5.0 Hz, 2H), 3.90 (s, 3H), 3.72 (s, 3H), 3.25–3.23 (m, 2H). 2.83–2.81 (m, 4H), 2.31 (s, 5H). ^13^C NMR (126 MHz, CDCl_3_) δ 153.6, 151.6, 150.0, 149.9, 148.8, 147.5, 130.1, 128.5, 121.6, 120.4, 117.0, 114.6, 112.1, 110.1, 102.1, 93.1, 69.5, 67.4, 56.2, 56.1, 54.8, 45.4. HRMS-ESI (*m*/*z*): [M + H]^+^ calculated for [C_25_H_29_O_4_N_5_^35^Cl]^+^: 498.1903, found: 498.1890. mp 177.3–179.0 °C (mean of *n* = 3 determinations).

#### 4-((5-Chloro-6-methoxypyridin-2-yl)amino)-6-methoxy-7-(2-((4-methylpiperazin-1-yl)oxy)ethoxy)quinoline-3-carbonitrile (**16a**)

To a stirred solution of **17** (420 mg, 1.12 mmol, 1.0 equiv) in 2-ethoxyethanol (10 mL) at r.t. was added 4 Å acid washed molecular sieves (2.2 g) pyridine·HCl (259 mg, 2.24 mmol, 2.0 equiv) followed by 5-chloro-6-methoxypyridin-2-amine (**24**) (265 mg, 1.68 mmol, 1.5 equiv). The solution was then brought to reflux (135 °C) and stirred for 12 h. After such time, the solvent was removed *in vacuo* and the resulting residue coconcentrated with toluene (3x, 50 mL) to remove remaining 2-ethoxyethanol. The residue obtained was dissolved in EtOAc (100 mL) and washed with saturated aq. K_2_CO_3_ (2x, 50 mL) and the organic layers combined, dried over Na_2_SO_4_, filtered and concentrated *in vacuo*. The resulting residue was purified by flash column chromatography on silica (eluent: 90:5:5 EtOAc:MeOH:Et_3_N) to afford the title compound (**16a**) (202 mg, 0.405 mmol, 36%) as a light orange solid. TLC R_f_ = 0.15 (90:5:5 EtOAc:MeOH:Et_3_N; CAM, UV). ^1^H NMR (500 MHz, CDCl_3_) δ 8.74 (s, 1H), 7.50 (d, *J* = 8.1 Hz, 1H), 7.43 (s, 1H), 7.38 (s, 1H), 7.08 (s, 1H), 6.29 (d, *J* = 8.2 Hz, 1H), 4.34 (t, *J* = 5.0 Hz, 2H), 4.14 (t, *J* = 4.9 Hz, 2H), 3.85 (s, 3H), 3.82 (s, 3H), 3.28–3.19 (m, 2H), 2.84–2.82 (m, 4H), 2.37–2.30 (m, 5H). ^13^C NMR (126 MHz, CDCl_3_) δ 158.3, 154.1, 150.6, 150.4, 149.4, 147.8, 146.7, 140.0, 117.0, 116.9, 110.5, 109.8, 103.7, 102.1, 97.4, 69.5, 67.5, 56.3, 54.9, 54.4, 53.7, 45.3. HRMS-ESI (*m*/*z*): [M + H]^+^ calculated for [C_24_H_28_O_4_N_6_^35^Cl]^+^: 499.1855, found: 499.1850. mp 102.3–104.1 °C (mean of *n* = 3 determinations).

### Computational Studies

All structures were optimized with the Hartree–Fock (HF) method and the Dunning basis set cc-pVDZ.^60−64^ Relative to the crystal structure of **29** (CCDC identification code: 2270277), the average bond error was 0.014 Å and the average angle error was 0.8° at the HF/cc-pVDZ level of theory (see Supporting Information for more details; Figure S14). To simplify the computation, the 2-(morpholinooxy)ethoxy unit in **29** was replaced by a methyl group and this truncated core was used for the optimization of all structures (**5b**–**8b**, **11b**–**16b**). Harmonic frequencies were computed analytically with the HF/cc-pVDZ level of theory to verify that each structure was a stationary point. Full coordinates are provided in the Supporting Information (Tables S21–S30).

The cp*K*_a_ of aniline (NH) was computed for each structure in H_2_O and DMSO using the Jaguar software.^65^ The cp*K*_a_ calculations utilize optimized geometries, single-point energies, and solvation-free energies for both the protonated and deprotonated species. Jaguar utilizes an automated process that applies the following methodology for calculating cp*K*_a_: geometry optimizations were computed with the B3LYP method and the 6-31G* basis set. Single-point energies were calculated with a larger basis set, cc-pVTZ(+). Solvation-free energies utilize empirical parametrization for the NH acid in either DMSO or H_2_O solvent.^65−68^ Given the intrinsic error in calculating p*K*_a_ values, Jaguar includes empirical corrections to yield optimal results with experimentally determined p*K*_a_ values. The corrections take a linear form (eq 1) where the *b* term relates to the surface tension correction and the *a* term related to the variation in charge on the ionizable group:
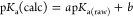
1

Jaguar presents an extensive list of training results showing a strong relationship between the calculated p*K*_a_ values and their experimental values based on functional group.

### Kinase Activity

*In vitro* kinase activity (biochemical inhibition (IC_50_’s)) were assessed by Eurofins Cerep. For biochemical IC_50_ determinations, compounds were run in duplicate (*n* = 2) and tested in an enzymatic radiometric assay using a 9-point, half-log dilution series at a top compound testing concentration of 10 μM and an ATP concentration of 10 μM with Eurofin’s KinaseProfiler technology.

### Kinase Selectivity Determination

Profiling of a 468-member human kinase panel was performed with Eurofins DiscoverX using the KINOMEScan platform.^55^ A panel of 468 kinases was assayed at a single concentration of 1 μM for **16a**. Percent control was mapped onto the kinome tree using TREE*spot*. The S scores were calculated as previously described^55^ and are reflective of the number of kinases which **16a** has affinity to over the total number of kinases.

### Cell Lines

The K562 cells were obtained from ATCC. K562/Dox cells were prepared by treatment of K562 cells with 60 nM doxorubicin (in DMSO) for 1 week.^69^ MDCKII-MDR1 cells were obtained from The Netherlands Cancer Institute. Caco-2 cells were obtained from the ATCC for permeability studies and flow cytometry studies. DLD1 and DU4475 cells were obtained from the ATCC. MDCKII-MDR1 cells were seeded at a density of 1.56 × 10^6^ cells/mL and cultivated for 4–8 days prior to assays. Caco-2 cells were seeded at a density of 6.86 × 10^5^ cells/mL and cultivated for 14–18 days prior to permeability assays. To exclude the effect of DMSO on K562 cells, K562 cells treated with 0.1% DMSO (K562-vehicle control cells) were used in flow cytometry and Western blot studies.

### Cell Viability Assay

Cells were plated in 96-well plates at 2,000 cells per well and dosed in triplicate (*n* = 3) in a 12-point, 5-fold dilution series with compounds (0.2048 pM to 10 μM; 25 μM and 50 μM doses were also included in the run) in DMSO and incubated for 72 h. After 72 h, cell viability was assayed by CellTiter-Glo Luminescnet Viability Assay (Promega). Dose–response curves were generated and used to calculate the IC_50_ values which were calculated on GraphPad Prism from the nonlinear regression equation fitted with a sigmoidal dose–response and are presented as mean (95% confidence interval).

### Flow Cytometry

Quantitative flow cytometry was performed to quantify single-cell expression of P-glycoprotein on five cell lines (DU4475, DLD1, Caco-2, K562-vehicle control and K562/Dox). First, culture media was removed, and cells were taken up in “staining buffer” (PBS + 0.5 mM EDTA + 1% BSA) to a density of 1 × 10^6^ cells/mL following trypsinization (Caco-2 and DLD1) and two washes. Second, 100 μL of each suspension (∼1 × 10^5^ cells) was added to four different Eppendorf tubes on ice and 4 μL of blocking IgG (Fischer PI31154) was added and incubated for 5 min. Third, 10 μL of phycoerythrin-conjugated anti-Pgp antibodies or isotype controls was added to two Eppendorf tubes each and incubated on ice for 30 min. Stained cells were washed with “staining buffer” (2x, 1 mL) and PBS (1x, 1 mL) prior to being taken up in 100 μL of PBS. Quantitation of P-gp expression was conducted on a BD Accuri benchtop flow cytometer using phycoerythin-conjugated calibration beads according to the manufacturer’s instruction (Bangs Laboratories #821). Flow cytometry results are provided in the Supporting Information (Figures S5–S11).

### Western Blot Studies

Total protein lysates were prepared with RIPA buffer and quantified with the DC Protein Assay kid (Bio-Rad). After the denatured proteins were separated on an 8% SDS-PAGE gel, they were transferred to a PVDF filter (Bio-Rad). After blocking with 5% nonfat milk-TBST (Bio-Rad), the blots were incubated with P-glycoprotein monoclonal antibody (ThermoFisher Scientific) or β-actin antibody (Cell Signaling) at 4 °C overnight, respectively. After washing with TBST (3x), the blots were incubated with peroxidase-linked secondary goat-antimouse IgG Ab (Bio-Rad) for 1 h at r.t. Finally, visual imaging was conducted by Odyssey Fc Imaging Systems (LI-COR) after incubation with SuperSignal West Pico PLUS chemiluminescent substrate (Thermo Fisher Scientific). Western blot results on K562-vehicle control cells and K562/Dox cells are provided in the Supporting Information (Figures S12–S13).

### *In Vitro* ADMET

Lipophilicity, solubility, plasma protein binding, metabolic stability in hepatocytes, permeability studies in Caco-2 cells and MDCKII-MDR1 cells, hERG channel inhibition and CYP inhibition was determined by Pharmaron Inc. using methods previously described.^33^

### Statistical Analyses

Statistical analysis was performed using GraphPad Prism 9.0. Data are presented as the mean ± SD or SEM as indicated when *n* ≥ 3, or as the geometric mean when *n* = 2. For *in vitro* ADMET and kinase activity studies, data is presented as mean of *n* ≥ 2 independent replicates. For *in vitro* short-term growth delay experiments, IC_50_ values were determined from the nonlinear regression equation fitted with a sigmoidal dose–response curve and are presented as the mean ± SD, *n* = 3 independent replicates and IC_50_ values are reported beside the dose–response curve and represent the mean (95% confidence interval).

## Supporting Information Available

Additional *in vitro* ADMET data and characterization data, including Tables S1–S47 and Figures S1–S30, with NMR spectra, X-ray crystallographic data for **28**, and references (PDF)
Molecular strings (CSV)

## Abbreviations Used

ABL1: Abelson murine leukemia viral oncogene homologue 1
Aq. Sol.: aqueous solubility at pH 7.4
BCRP: breast cancer resistance protein
Caco-2: colon carcinoma cell line
CNS: central nervous system
DFT: density functional theory
FBS: fetal bovine serum
*f*_u_: fraction unbound
H: human
HEPCl_int_: intrinsic clearance in hepatocytes
logD_7.4_: logarithm of distribution at pH 7.4
M: mouse
MDCK: madine-darby canine kidney cell line
nd: not determined
*P*_app_: apparent permeability
SD: standard deviation
SEM: standard error of the mean
TDI: time-dependent inhibition
tPSA: total polar surface area
