# Supplementary material for: Computationally Driven Discovery of a BCR-ABL1 Kinase Inhibitor with Activity in Multidrug-Resistant Chronic Myeloid Leukemia
Source: J Med Chem. 2024 Sep 23;67(19):17820–32. doi: 10.1021/acs.jmedchem.4c01852 (PMC11472320; doi:10.1021/acs.jmedchem.4c01852)
Supplement: Supplementary file 1 — jm4c01852_si_001.pdf [file jm4c01852_si_001.pdf]

Supporting Information for

Computationally-Driven Discovery of a BCR-ABL1 Kinase Inhibitor with Activity in Multidrug Resistant Chronic Myeloid Leukemia

Jarvis Hill,[1,2,‡] R. Houston Givhan,[2,3] Bin Yi,[1] Robert M. Jones,[4] Eugene F. Douglass,[1] Yaguang Xi,[1] Henry F. Schaefer III[2,3] and David Crich[1,2,5,*]

[1]Department of Pharmaceutical and Biomedical Sciences, University of Georgia, 250 West Green Street, Athens, GA 30602, USA.

[2]Department of Chemistry, University of Georgia, 302 East Campus Road, Athens, GA 30602, USA.

[3]Center of Computational Quantum Chemistry, University of Georgia, 1004 Cedar Street, Athens, GA 30602, USA.

[4]P.O. Box 568, Oakley, UT 84055-0568, USA.

[5]Complex Carbohydrate Research Center, University of Georgia, 315 Riverbend Road, Athens, GA 30602, USA.

[‡]Current Address: Department of Chemistry, Yale University, 225 Prospect Street, New Haven, CT 06520, USA.

*To whom correspondence should be addressed: David.Crich@uga.edu (D.C.)

Table of Contents

1. Extended Figure S2
2. Biological Materials and Data S3
3. Supporting Chemistry Experimental Details S34
4. Catalog of Spectra S57
5. X-ray Crystal Structure of **28** S140
6. References S142

1. Extended Figure

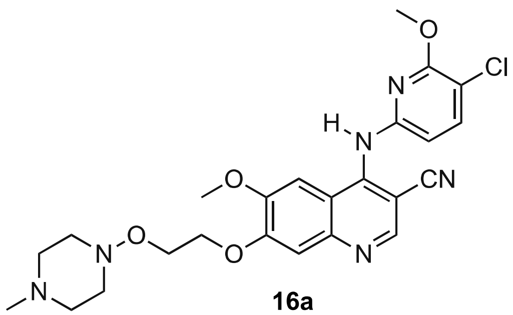

16a

**ADMET Property**	**16a**
Aq. Solubility (μM) (pH 1.2/6.8/7.4)	289/8.2/2.1
Aq. Solubility (μM) (FaSSIF/FeSSIF/SGF/FeSSGF)	154/189/303/107
$f_{\text{u, plasma}}$% (H/M)	2.5/5.7
$HEPCl_{int}$ (H/M)	49.5/55.7
$t_{1/2}$ (min) (H/M)	28.0/24.9
CYP (IC_{50}) (μM) (3A4/1A2/2D6/2C9)	1.0/>30/27.1/16.5
CYP 3A4 TDI	Negative

Figure S1. Extended *in vitro* absorption, distribution, metabolism, excretion and toxicity (ADMET) profile of **16a**. All values represent the mean of n = 2 independent replicates unless otherwise stated. Low drug-drug interactions (DDI) are predicted for **16a** with only moderate inhibition of CYP3A4 observed, which was negative in a follow-up time-dependent inhibition (TDI) study in human liver microsomes. See Supporting Information for details. Aq. Sol., aqueous solubility; $f_{\text{u,plasma}}$ %, percent fraction unbound in plasma; $HEPCl_{int}$, intrinsic clearance in hepatocytes; $t_{1/2}$, half-life; CYP (IC_{50}), cytochrome-P450 inhibition. Abbreviations; H, human; M, mouse.

2. Biological Materials and Data

Biochemical human RTK kinase enzymatic radiometric assay, KinaseProfiler

Table S1. Biochemical IC_{50} results for compounds against BCR-ABL1 and cSRC.

Kinase	Bosutinib[b]	5a	8a	12a	13a	14a	15a	16a
**BCR-ABL1 IC_{50} (nM)[a]**								
Wt	<1	< 1	1	20	19	< 1	6	< 1
H396P	< 1	< 1	< 1	20	15	< 1	7	< 1
M351T	< 1	< 1	2	31	24	1	13	< 1
Q252H	< 1	< 1	2	37	30	< 1	10	< 1
T315I	44	47	166	368	445	95	220	40
**cSRC IC_{50} (nM)[a]**								
wt	2	2	2	14	34	2	11	2

[a]Conducted by Eurofins Cerep, SA. IC_{50} values represent the mean of $n = 2$ technical replicates conducted at [ATP] = 10 µM. Values < 1 nM were below the sensitivity of the test and as such are reported as < 1 nM. [b]IC_{50} values as previously reported in Ref. 1.

Plasma protein binding by equilibrium dialysis

Table S2. Plasma details used in the plasma protein binding assay.

Item	Supplier
Human plasma (mixed gender)	BiolVT
Mouse plasma (CD-1/mixed gender)	IPHASE or BiolVT

Table S3. Results for plasma protein binding in human and mouse plasma.

Compound	Species	%Bound	%Unbound	%Recovery	%Remaining at 6 h
Ketoconazole	Human	99.20	0.80	101.64	94.60
Bosutinib[a]	Human	95.47	4.52	94.01	102.03
**5a**[a]	Human	95.05	4.95	92.41	101.74
**8a**	Human	94.42	5.58	99.90	92.70
**12a**	Human	92.63	7.37	92.54	97.83
**13a**	Human	87.03	12.97	87.76	98.29
**15a**	Human	92.27	7.73	95.83	99.42
**16a**	Human	97.52	2.48	93.86	97.78
Ketoconazole	Mouse	99.39	0.61	102.46	103.53
**12a**	Mouse	88.07	11.93	92.03	96.71
**15a**	Mouse	90.29	9.71	95.87	106.50
**16a**	Mouse	94.31	5.69	93.05	97.78

[a]Values as previously reported in Ref. 1.

Metabolic stability in hepatocytes

Table S4. Hepatocyte details used in the hepatocyte stability assay.

Item	Supplier
Human Hepatocytes, Mixed-Gender	BiolVT (Cat No. X008001)
Mouse Hepatocytes, Male-Gender	BiolVT (Cat No. M005052)

Table S5. Results for hepatocyte stability data in human hepatocytes.

Compound	Species	Remaining Percentages (%)					
		0.5 min	15 min	30 min	60 min	90 min	120 min
Verapamil	Human	100.00	56.11	35.65	17.71	8.83	4.83
Bosutinib[a]	Human	100.00	73.99	79.28	53.95	34.15	20.73
**5a**[a]	Human	100.00	90.07	69.58	44.09	23.58	13.59
**8a**	Human	100.00	69.28	52.48	29.45	15.72	9.48
**11a**	Human	100.00	82.49	66.92	45.06	29.85	18.60
**12a**	Human	100.00	87.31	80.64	60.46	46.43	35.84
**13a**	Human	100.00	97.02	95.67	80.41	67.22	54.89
**14a**	Human	100.00	83.13	68.12	50.16	37.41	25.52
**15a**	Human	100.00	78.44	63.53	43.29	27.95	20.14
**16a**	Human	100.00	71.19	50.26	22.99	9.44	4.39
Verapamil	Mouse	100.00	16.71	4.39	1.24	BLOD	BLOD
**15a**	Mouse	100.00	55.53	28.57	13.26	6.75	2.89
**16a**	Mouse	100.00	66.11	33.68	19.55	8.83	3.16

[a]Values as previously reported in Ref. 1. Abbreviations: BLOD, below level of detection.

MDCKII-MDR1 Permeability

Table S6. MDCKII-MDR1 cellular details used in the MDCKII-MDR1 permeability assay.

Item	Supplier
MDCKII-MDR1 cells	Netherlands Cancer Institute (Amsterdam)
HTS Transwell 96 Well	Corning (Cat No. 3391)

Table S7. Results for MDCKII-MDR1 cellular permeability.

Compound	$P_{app(a\text{-}b)}$ (10^{-6}, cm/s)	$P_{app(b\text{-}a)}$ (10^{-6}, cm/s)	Efflux Ratio	Recovery% (AP-BL)	Recovery% (BL-AP)
Metoprolol	31.60	28.05	0.89	111.64	99.15
Digoxin	0.62	13.94	22.68	82.38	93.20
Bosutinib[a]	0.38	11.34	29.85	56.02	65.52
**5a**[a]	0.52	10.28	19.78	45.83	58.85
**8a**	0.73	19.30	26.45	79.78	64.87
**11a**	7.16	29.64	4.15	71.59	85.59
**12a**	12.48	38.99	3.22	94.96	91.60
**13a**	7.53	54.36	7.26	93.06	105.61
**14a**	4.08	24.71	6.05	75.42	77.51
**15a**	5.90	24.77	4.20	84.45	72.29
**16a**	4.53	24.42	5.39	76.05	73.24

[a]Values as previously reported in Ref. 1. Abbreviations: P_{app}, apparent permeability; AP, apical; BL, basolateral.

Caco-2 permeability

Table S8. Caco-2 cellular details used in the Caco-2 cellular permeability assay.

Item	Supplier
Caco-2 cells	ATCC (ATCC No. HTB-37)
HTS Transwell 96 Well	Corning (Cat No. 3391)

Table S9. Results for Caco-2 cellular permeability.

Compound	$P_{app(a\text{-}b)}$ (10^{-6}, cm/s)	$P_{app(b\text{-}a)}$ (10^{-6}, cm/s)	Efflux Ratio	Recovery% (AP-BL)	Recovery% (BL-AP)
Metoprolol	18.76	19.39	1.04	95.33	95.54
Digoxin	0.22	12.58	58.64	89.90	92.72
Bosutinib[a]	0.86	10.87	12.70	52.12	70.73
**5a**[a]	1.05	4.04	3.88	39.68	54.25
**8a**	1.94	12.08	6.22	61.16	72.31
**11a**	3.66	27.39	7.49	60.54	80.51
**12a**	6.46	23.06	3.58	76.24	87.19
**13a**	2.72	33.58	12.36	84.83	92.56
**14a**	1.99	37.41	18.96	65.43	92.37
**15a**	0.71	4.44	6.24	72.15	78.22
**16a**	0.46	7.70	16.70	72.75	68.21

[a]Values as previously reported in Ref. 1. Abbreviations: P_{app}, apparent permeability; AP, apical; BL, basolateral.

hERG Safety evaluation by manual patch-clamp system

Table S10. HEK293 cellular details used in hERG safety evaluation.

**Item**	**Supplier**
HEK 293 Cell Line	Invitrogen (Cat No. K1236)
TrypLE^{TM} Express	Gibco (Cat No. 12604)
Dofetilide	TRC (Cat No. D525700)

Table S11. hERG safety evaluation results.

**Compound**	**hERG IC$_{50}$ (μM)[a]**
Dofetilide[b,c]	0.015 $\pm$ 0.0008
Bosutinib[c]	1.01 $\pm$ 0.4
**5a**[c]	3.41 $\pm$ 0.3
**12a**	5.16 $\pm$ 1.1
**15a**[d]	4.36 $\pm$ 1.4
**16a**[d]	3.14 $\pm$ 0.8

[a]IC$_{50}$ values are presented as the mean $\pm$ SEM, n = 3 independent replicates unless otherwise specified. [b]Dofetilide tested at 5 concentrations (0.00185, 0.00556, 0.01667, 0.05000, 0.15000 μM) and run in triplicate. [c]Values as previously reported in Ref. 1.[d]Mean of n = 2 independent replicates.

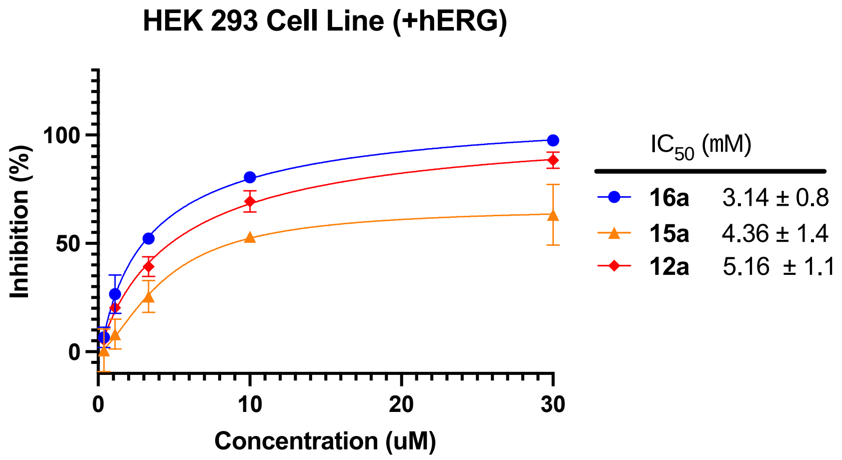

Figure S2. **16a** has low hERG inhibitory potential. Points indicate the mean, and error bars indicate the SD; $n \geq 2$ independent replicates. IC$_{50}$ values (μM) are reported beside the dose-response curves and represent the mean $\pm$ SEM.

CYP Inhibition of CYP1A2, CYP2C9, CYP2D6 and CYP3A4 in human liver microsomes

Table S12. CYP450 control compound details used in the CYP inhibition assay.

CYP Isoform	Positive Inhibitors	Final Concentration (μM)
CYP1A2	Furafylline	0, 0.0075, 0.025, 0.075, 0.25, 0.75, 2.5, 7.5, 25
CYP2C9	Sulfaphenazole	0, 0.0015, 0.005, 0.015, 0.05, 0.15, 0.5, 1.5, 5
CYP2D6	Quinidine	0, 0.0015, 0.005, 0.015, 0.05, 0.15, 0.5, 1.5, 5
CYP3A4	Ketoconazole	0, 0.0015, 0.005, 0.015, 0.05, 0.15, 0.5, 1.5, 5

Table S13. CYP450 substrate concentration details.

CYP Isoform	Substrate	Working Concentration (μM)	Final Concentration (μM)	Incubation Time
CYP1A2	Phenacetin	800	40	20 min
CYP2C9	Diclofenac	120	6	5 min
CYP2D6	Dextromethorphan	40	2	20 min
CYP3A4	Midazolam	20	1	5 min

Table S14. Results for CYP450 inhibition assay.

Compound	IC_{50} (μM)[a]			
	**CYP1A2**	**CYP2C9**	**CYP2D6**	**CYP3A4-M**
Furafylline	3.01	-	-	-
Sulfaphenazole	-	0.21	-	-
Quinidine	-	-	0.023	-
Ketoconazole	-	-	-	0.016
**16a**	> 30	16.5	27.1	1.0

[a]IC_{50} values are presented as the mean (n = 2 independent replicates).

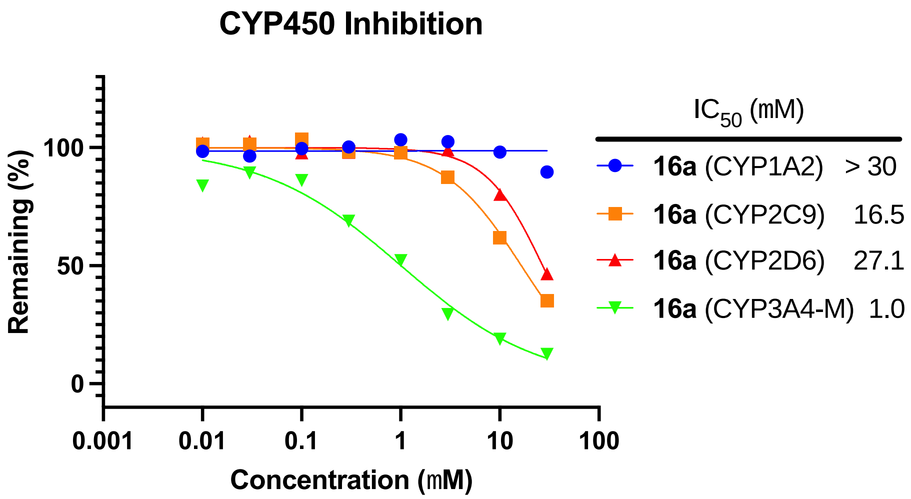

Figure S3. Low DDI is predicted for compound **16a**. Only moderate CYP3A4 inhibition was observed. Points indicate mean; *n* = 2 independent replicates. IC_{50} (µM) are reported beside the dose-response curves and represent the mean.

Direct and time-dependent inhibition of CYP3A4 in human liver microsomes

Table S15. Microsome details used in direct and time-dependent CYP3A4 inhibition study.

**Item**	**Supplier**
Pooled Human Liver Microsomes, Mixed Gender	BD Gentest (Cat. No. 452117)

Table S16. CYP3A4 inhibitor compound concentration details.

**CYP Isoform**	**Positive Control**	**Working Concentration (μM)**	**Final Concentration (μM)**
CYP3A4	Mifepristone	0.02, 0.06, 0.2, 0.6, 2, 6	0.1, 0.3, 1, 3, 10, 30
CYP3A4	**16a**	6, 20, 60, 200, 600, 2000	0.03, 0.1, 0.3, 1, 3, 10

Table S17. Final CYP3A4 substrate concentrations used in assay.

**CYP Isoform**	**Substrate**	**Working Concentration (μM)**	**Final Concentration (μM)**	**Incubation Time**
CYP3A4	Midazolam	20	1	5 min

Table S18. Results for CYP3A4 time-dependent inhibition assay in human liver microsomes.

**Compound**	**Pre-incubation**	**CYP2D6**	
		**IC_{50} (μM)[a]**	**Inhibition percentage(%) at Top conc.**
Mifepristone	0 min	1.13	84.46
	30 min without NADPH	1.00	80.51
	30 min with NADPH	0.10	96.70
**16a**	0 min	1.47	72.41
	30 min without NADPH	1.50	72.07
	30 min with NADPH	2.64	76.57

[a]IC_{50} values are presented as the mean (n = 2 independent replicates).

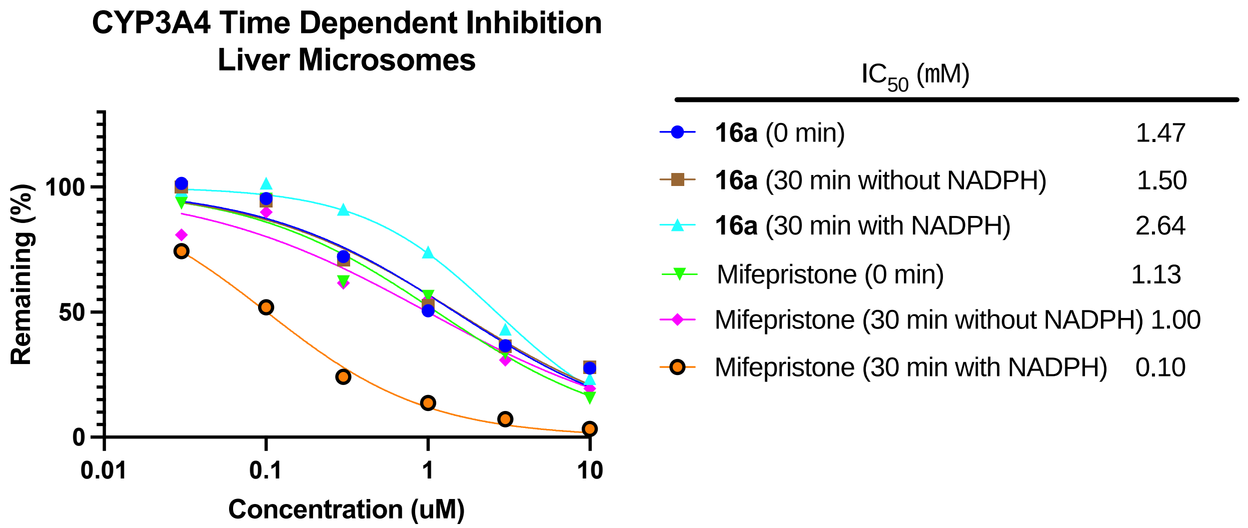

Figure S4. Compound **16a** was negative in a follow-up CYP3A4 time-dependent inhibition (TDI) study. Points indicate mean; $n = 2$ independent replicates. IC_{50} (µM) are reported beside the dose-response curves and represent the mean.

KINOMEScan

Table S19. Full KINOMEScan results for **16a** at a single concentration of 1 μM.

DiscoveRx Gene Symbol	Entrez Gene Symbol	Percent Control
AAK1	AAK1	6.8
ABL1(E255K)-phosphorylated	ABL1	0.55
ABL1(F317I)-nonphosphorylated	ABL1	2.3
ABL1(F317I)-phosphorylated	ABL1	18
ABL1(F317L)-nonphosphorylated	ABL1	0.45
ABL1(F317L)-phosphorylated	ABL1	0
ABL1(H396P)-nonphosphorylated	ABL1	0.15
ABL1(H396P)-phosphorylated	ABL1	0.05
ABL1(M351T)-phosphorylated	ABL1	0
ABL1(Q252H)-nonphosphorylated	ABL1	0
ABL1(Q252H)-phosphorylated	ABL1	0.65
ABL1(T315I)-nonphosphorylated	ABL1	1.3
ABL1(T315I)-phosphorylated	ABL1	0.15
ABL1(Y253F)-phosphorylated	ABL1	0.2
ABL1-nonphosphorylated	ABL1	0
ABL1-phosphorylated	ABL1	0
ABL2	ABL2	0
ACVR1	ACVR1	100
ACVR1B	ACVR1B	93

ACVR2A	ACVR2A	100
ACVR2B	ACVR2B	100
ACVRL1	ACVRL1	100
ADCK3	CABC1	100
ADCK4	ADCK4	52
AKT1	AKT1	100
AKT2	AKT2	97
AKT3	AKT3	100
ALK	ALK	76
ALK(C1156Y)	ALK	31
ALK(L1196M)	ALK	10
AMPK-alpha1	PRKAA1	31
AMPK-alpha2	PRKAA2	60
ANKK1	ANKK1	97
ARK5	NUAK1	74
ASK1	MAP3K5	100
ASK2	MAP3K6	71
AURKA	AURKA	100
AURKB	AURKB	100
AURKC	AURKC	61
AXL	AXL	7.4
BIKE	BMP2K	1.2
BLK	BLK	0.2
BMPR1A	BMPR1A	76
BMPR1B	BMPR1B	100
BMPR2	BMPR2	29
BMX	BMX	23
BRAF	BRAF	100
BRAF(V600E)	BRAF	100
BRK	PTK6	96
BRSK1	BRSK1	100
BRSK2	BRSK2	87

BTK	BTK	0.4
BUB1	BUB1	100
CAMK1	CAMK1	65
CAMK1B	PNCK	91
CAMK1D	CAMK1D	2
CAMK1G	CAMK1G	29
CAMK2A	CAMK2A	44
CAMK2B	CAMK2B	80
CAMK2D	CAMK2D	47
CAMK2G	CAMK2G	55
CAMK4	CAMK4	100
CAMKK1	CAMKK1	47
CAMKK2	CAMKK2	32
CASK	CASK	68
CDC2L1	CDK11B	100
CDC2L2	CDC2L2	96
CDC2L5	CDK13	100
CDK11	CDK19	99
CDK2	CDK2	93
CDK3	CDK3	99
CDK4	CDK4	100
CDK4-cyclinD1	CDK4	70
CDK4-cyclinD3	CDK4	99
CDK5	CDK5	97
CDK7	CDK7	26
CDK8	CDK8	91
CDK9	CDK9	100
CDKL1	CDKL1	70
CDKL2	CDKL2	39
CDKL3	CDKL3	76
CDKL5	CDKL5	100
CHEK1	CHEK1	6.9

CHEK2	CHEK2	0.75
CIT	CIT	1.2
CLK1	CLK1	24
CLK2	CLK2	34
CLK3	CLK3	36
CLK4	CLK4	8.1
CSF1R	CSF1R	23
CSF1R-autoinhibited	CSF1R	65
CSK	CSK	0.8
CSNK1A1	CSNK1A1	2
CSNK1A1L	CSNK1A1L	4
CSNK1D	CSNK1D	3.4
CSNK1E	CSNK1E	0.5
CSNK1G1	CSNK1G1	77
CSNK1G2	CSNK1G2	92
CSNK1G3	CSNK1G3	97
CSNK2A1	CSNK2A1	100
CSNK2A2	CSNK2A2	76
CTK	MATK	100
DAPK1	DAPK1	50
DAPK2	DAPK2	74
DAPK3	DAPK3	83
DCAMKL1	DCLK1	70
DCAMKL2	DCLK2	85
DCAMKL3	DCLK3	9.1
DDR1	DDR1	25
DDR2	DDR2	19
DLK	MAP3K12	11
DMPK	DMPK	3.3
DMPK2	CDC42BPG	31
DRAK1	STK17A	91
DRAK2	STK17B	100

DYRK1A	DYRK1A	40
DYRK1B	DYRK1B	42
DYRK2	DYRK2	92
EGFR	EGFR	0.6
EGFR(E746-A750del)	EGFR	0
EGFR(G719C)	EGFR	0.7
EGFR(G719S)	EGFR	0.3
EGFR(L747-E749del, A750P)	EGFR	1.1
EGFR(L747-S752del, P753S)	EGFR	0.5
EGFR(L747-T751del,Sins)	EGFR	4.5
EGFR(L858R)	EGFR	0.15
EGFR(L858R,T790M)	EGFR	29
EGFR(L861Q)	EGFR	1
EGFR(S752-I759del)	EGFR	1.9
EGFR(T790M)	EGFR	21
EIF2AK1	EIF2AK1	2.6
EPHA1	EPHA1	69
EPHA2	EPHA2	7.1
EPHA3	EPHA3	7.3
EPHA4	EPHA4	7
EPHA5	EPHA5	12
EPHA6	EPHA6	90
EPHA7	EPHA7	96
EPHA8	EPHA8	1.1
EPHB1	EPHB1	13
EPHB2	EPHB2	6.7
EPHB3	EPHB3	72
EPHB4	EPHB4	8.8
EPHB6	EPHB6	73
ERBB2	ERBB2	7

ERBB3	ERBB3	0
ERBB4	ERBB4	0.65
ERK1	MAPK3	100
ERK2	MAPK1	100
ERK3	MAPK6	49
ERK4	MAPK4	78
ERK5	MAPK7	100
ERK8	MAPK15	99
ERN 1.00	ERN 1.00	100
FAK	PTK2	43
FER	FER	13
FES	FES	14
FGFR1	FGFR1	77
FGFR2	FGFR2	81
FGFR3	FGFR3	86
FGFR3(G697C)	FGFR3	91
FGFR4	FGFR4	100
FGR	FGR	5.4
FLT1	FLT1	61
FLT3	FLT3	27
FLT3(D835H)	FLT3	25
FLT3(D835V)	FLT3	0
FLT3(D835Y)	FLT3	13
FLT3(ITD)	FLT3	32
FLT3(ITD,D835V)	FLT3	2.6
FLT3(ITD,F691L)	FLT3	18
FLT3(K663Q)	FLT3	34
FLT3(N841I)	FLT3	17
FLT3(R834Q)	FLT3	35
FLT3-autoinhibited	FLT3	100
FLT4	FLT4	67
FRK	FRK	6.1

FYN	FYN	11
GAK	GAK	3.9
GCN2(Kin.Dom.2,S808G)	EIF2AK4	1.8
GRK1	GRK1	64
GRK2	ADRBK1	97
GRK3	ADRBK2	76
GRK4	GRK4	18
GRK7	GRK7	61
GSK3A	GSK3A	100
GSK3B	GSK3B	100
HASPIN	GSG2	89
HCK	HCK	0.6
HIPK1	HIPK1	16
HIPK2	HIPK2	64
HIPK3	HIPK3	90
HIPK4	HIPK4	2.5
HPK1	MAP4K1	0.1
HUNK	HUNK	37
ICK	ICK	100
IGF1R	IGF1R	96
IKK-alpha	CHUK	100
IKK-beta	IKBKB	94
IKK-epsilon	IKBKE	38
INSR	INSR	93
INSRR	INSRR	90
IRAK1	IRAK1	73
IRAK3	IRAK3	72
IRAK4	IRAK4	72
ITK	ITK	86
JAK1(JH1domain-catalytic)	JAK1	100
JAK1(JH2domain-pseudokinase)	JAK1	94

JAK2(JH1domain-catalytic)	JAK2	4.3
JAK3(JH1domain-catalytic)	JAK3	3.1
JNK1	MAPK8	94
JNK2	MAPK9	97
JNK3	MAPK10	100
KIT	KIT	56
KIT(A829P)	KIT	44
KIT(D816H)	KIT	31
KIT(D816V)	KIT	9.8
KIT(L576P)	KIT	41
KIT(V559D)	KIT	45
KIT(V559D,T670I)	KIT	93
KIT(V559D,V654A)	KIT	20
KIT-autoinhibited	KIT	98
LATS1	LATS1	100
LATS2	LATS2	91
LCK	LCK	0.35
LIMK1	LIMK1	97
LIMK2	LIMK2	100
LKB1	STK11	68
LOK	STK10	0.1
LRRK2	LRRK2	90
LRRK2(G2019S)	LRRK2	37
LTK	LTK	56
LYN	LYN	1.4
LZK	MAP3K13	92
MAK	MAK	100
MAP3K1	MAP3K1	84
MAP3K15	MAP3K15	100
MAP3K2	MAP3K2	0.55
MAP3K3	MAP3K3	0.1

MAP3K4	MAP3K4	2.6
MAP4K2	MAP4K2	0
MAP4K3	MAP4K3	0.75
MAP4K4	MAP4K4	0.9
MAP4K5	MAP4K5	0.4
MAPKAPK2	MAPKAPK2	100
MAPKAPK5	MAPKAPK5	100
MARK1	MARK1	43
MARK2	MARK2	32
MARK3	MARK3	95
MARK4	MARK4	60
MAST1	MAST1	96
MEK1	MAP2K1	0
MEK2	MAP2K2	0
MEK3	MAP2K3	36
MEK4	MAP2K4	100
MEK5	MAP2K5	0.1
MEK6	MAP2K6	46
MELK	MELK	65
MERTK	MERTK	27
MET	MET	41
MET(M1250T)	MET	68
MET(Y1235D)	MET	80
MINK	MINK1	3.8
MKK7	MAP2K7	100
MKNK1	MKNK1	100
MKNK2	MKNK2	100
MLCK	MYLK3	61
MLK1	MAP3K9	11
MLK2	MAP3K10	42
MLK3	MAP3K11	12
MRCKA	CDC42BPA	88

MRCKB	CDC42BPB	93
MST1	STK4	20
MST1R	MST1R	100
MST2	STK3	29
MST3	STK24	25
MST4	MST4	4
MTOR	MTOR	80
MUSK	MUSK	22
MYLK	MYLK	35
MYLK2	MYLK2	16
MYLK4	MYLK4	100
MYO3A	MYO3A	43
MYO3B	MYO3B	32
NDR1	STK38	80
NDR2	STK38L	85
NEK1	NEK1	62
NEK10	NEK10	100
NEK11	NEK11	61
NEK2	NEK2	50
NEK3	NEK3	81
NEK4	NEK4	38
NEK5	NEK5	96
NEK6	NEK6	98
NEK7	NEK7	94
NEK9	NEK9	87
NIK	MAP3K14	18
NIM1	MGC42105	5.5
NLK	NLK	97
OSR1	OXSR1	98
p38-alpha	MAPK14	50
p38-beta	MAPK11	97
p38-delta	MAPK13	100

p38-gamma	MAPK12	97
PAK1	PAK1	48
PAK2	PAK2	27
PAK3	PAK3	24
PAK4	PAK4	47
PAK6	PAK6	99
PAK7	PAK7	39
PCTK1	CDK16	100
PCTK2	CDK17	100
PCTK3	CDK18	99
PDGFRA	PDGFRA	100
PDGFRB	PDGFRB	8.8
PDPK1	PDPK1	100
PFCDPK1(P.falciparum)	CDPK1	18
PFPK5(P.falciparum)	MAL13P1.279	100
PFTAIRE2	CDK15	65
PFTK1	CDK14	95
PHKG1	PHKG1	34
PHKG2	PHKG2	76
PIK3C2B	PIK3C2B	100
PIK3C2G	PIK3C2G	76
PIK3CA	PIK3CA	100
PIK3CA(C420R)	PIK3CA	100
PIK3CA(E542K)	PIK3CA	100
PIK3CA(E545A)	PIK3CA	79
PIK3CA(E545K)	PIK3CA	89
PIK3CA(H1047L)	PIK3CA	100
PIK3CA(H1047Y)	PIK3CA	73
PIK3CA(I800L)	PIK3CA	100
PIK3CA(M1043I)	PIK3CA	100
PIK3CA(Q546K)	PIK3CA	100
PIK3CB	PIK3CB	96

PIK3CD	PIK3CD	74
PIK3CG	PIK3CG	97
PIK4CB	PI4KB	100
PIKFYVE	PIKFYVE	79
PIM1	PIM1	91
PIM2	PIM2	100
PIM3	PIM3	84
PIP5K1A	PIP5K1A	15
PIP5K1C	PIP5K1C	55
PIP5K2B	PIP4K2B	10
PIP5K2C	PIP4K2C	64
PKAC-alpha	PRKACA	100
PKAC-beta	PRKACB	100
PKMYT1	PKMYT1	18
PKN1	PKN1	66
PKN2	PKN2	48
PKNB(M.tuberculosis)	pknB	53
PLK1	PLK1	100
PLK2	PLK2	42
PLK3	PLK3	90
PLK4	PLK4	99
PRKCD	PRKCD	42
PRKCE	PRKCE	70
PRKCH	PRKCH	84
PRKCI	PRKCI	61
PRKCQ	PRKCQ	30
PRKD1	PRKD1	78
PRKD2	PRKD2	71
PRKD3	PRKD3	58
PRKG1	PRKG1	100
PRKG2	PRKG2	100
PRKR	EIF2AK2	64

PRKX	PRKX	100
PRP4	PRPF4B	33
PYK2	PTK2B	55
QSK	KIAA0999	5.5
RAF1	RAF1	100
RET	RET	48
RET(M918T)	RET	44
RET(V804L)	RET	70
RET(V804M)	RET	59
RIOK1	RIOK1	16
RIOK2	RIOK2	100
RIOK3	RIOK3	0.8
RIPK1	RIPK1	100
RIPK2	RIPK2	79
RIPK4	RIPK4	100
RIPK5	DSTYK	22
ROCK1	ROCK1	31
ROCK2	ROCK2	8.6
ROS1	ROS1	100
RPS6KA4(Kin.Dom.1-N-terminal)	RPS6KA4	95
RPS6KA4(Kin.Dom.2-C-terminal)	RPS6KA4	91
RPS6KA5(Kin.Dom.1-N-terminal)	RPS6KA5	100
RPS6KA5(Kin.Dom.2-C-terminal)	RPS6KA5	84
RSK1(Kin.Dom.1-N-terminal)	RPS6KA1	37
RSK1(Kin.Dom.2-C-terminal)	RPS6KA1	81
RSK2(Kin.Dom.1-N-terminal)	RPS6KA3	64
RSK2(Kin.Dom.2-C-terminal)	RPS6KA3	100

RSK3(Kin.Dom.1-N-terminal)	RPS6KA2	75
RSK3(Kin.Dom.2-C-terminal)	RPS6KA2	63
RSK4(Kin.Dom.1-N-terminal)	RPS6KA6	46
RSK4(Kin.Dom.2-C-terminal)	RPS6KA6	87
S6K1	RPS6KB1	34
SBK1	SBK1	86
SGK	SGK1	100
SgK110	SgK110	100
SGK2	SGK2	98
SGK3	SGK3	100
SIK	SIK1	1.1
SIK2	SIK2	3
SLK	SLK	0.2
SNARK	NUAK2	7.2
SNRK	SNRK	69
SRC	SRC	0.1
SRMS	SRMS	11
SRPK1	SRPK1	96
SRPK2	SRPK2	100
SRPK3	SRPK3	95
STK16	STK16	91
STK33	STK33	4.3
STK35	STK35	0.65
STK36	STK36	5.5
STK39	STK39	79
SYK	SYK	3.6
TAK1	MAP3K7	0.1
TAOK1	TAOK1	73
TAOK2	TAOK2	56

TAOK3	TAOK3	28
TBK1	TBK1	30
TEC	TEC	93
TESK1	TESK1	98
TGFBR1	TGFBR1	100
TGFBR2	TGFBR2	100
TIE1	TIE1	81
TIE2	TEK	70
TLK1	TLK1	55
TLK2	TLK2	89
TNIK	TNIK	5
TNK1	TNK1	74
TNK2	TNK2	2.2
TNNI3K	TNNI3K	30
TRKA	NTRK1	62
TRKB	NTRK2	69
TRKC	NTRK3	100
TRPM6	TRPM6	100
TSSK1B	TSSK1B	25
TSSK3	TSSK3	76
TTK	TTK	96
TXK	TXK	2.3
TYK2(JH1domain-catalytic)	TYK2	59
TYK2(JH2domain-pseudokinase)	TYK2	84
TYRO3	TYRO3	7
ULK1	ULK1	83
ULK2	ULK2	14
ULK3	ULK3	0.55
VEGFR2	KDR	84
VPS34	PIK3C3	100
VRK2	VRK2	46

WEE1	WEE1	12
WEE2	WEE2	1.1
WNK1	WNK1	60
WNK2	WNK2	64
WNK3	WNK3	7.5
WNK4	WNK4	100
YANK1	STK32A	100
YANK2	STK32B	100
YANK3	STK32C	79
YES	YES1	3.2
YSK1	STK25	35
YSK4	MAP3K19	0.35
ZAK	ZAK	1.6
ZAP70	ZAP70	58

Selectivity scores (S-scores) is a quantitaive measure of compound selectivity and was calculated as previously described.[2] KINOMEScan individual data points are listed above and were used to calculate the following selectivity scores.

Table S20. Selectivity scores for **16a** at a screening concentration of 1 μM.

**Selectivity Score Type**	**Number of Hits**	**Number of Non-Mutant Kinases**	**Screening Concentration**	**Selectivity Score**
S(35)[a]	131	403	1000 nM	0.325
S(10)[b]	79	403	1000 nM	0.196
S(1)[c]	30	403	1000 nM	0.074

[a]S(35) refers to: (the number of non-mutant kinases with percent control < 35) / (number of non-mutant kinases tested). [b]S(10) refers to: (the number of non-mutant kinases with percent control < 10) / (number of non-mutant kinases tested). [c]S(1) refers to: (the number of non-mutant kinases with percent control < 1) / (number of non-mutant kinases tested).

Flow Cytometry

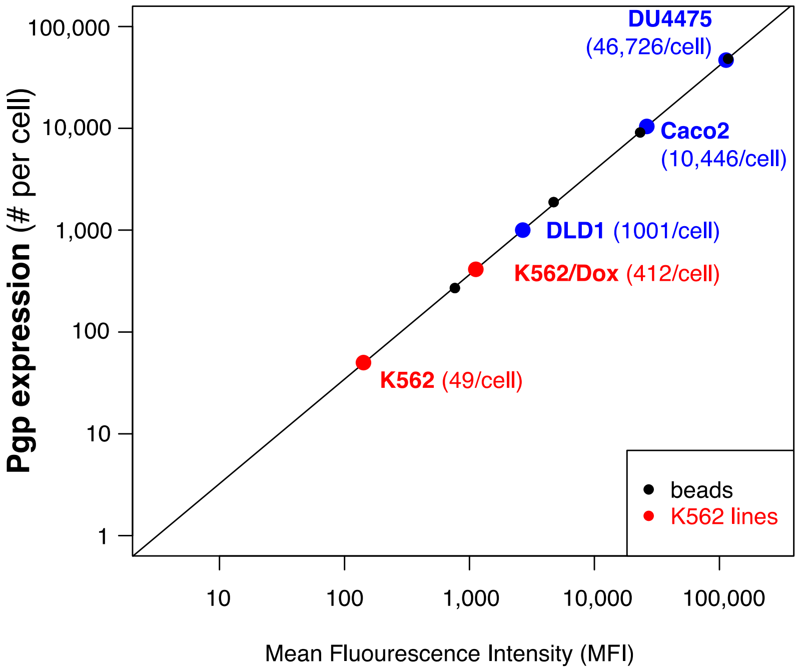

Figure S5. Flow cytometry analysis curve of K562 cells, calibration beads and control cells (DLD1, Caco-2 and DU4475).

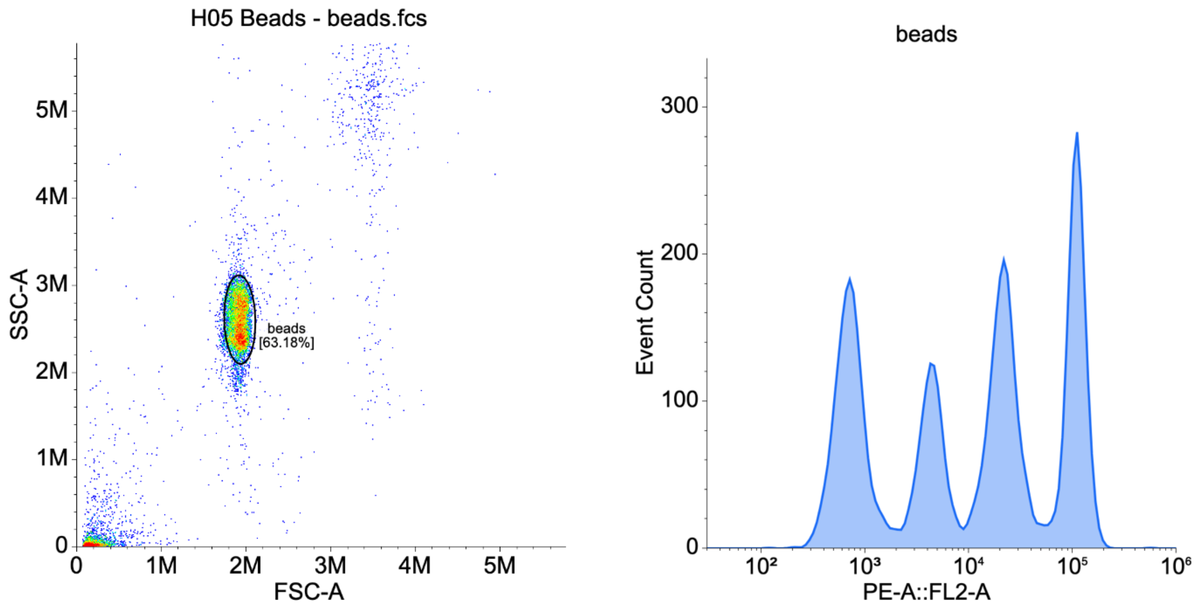

Figure S6. Bead calibration for flow cytometry analysis.

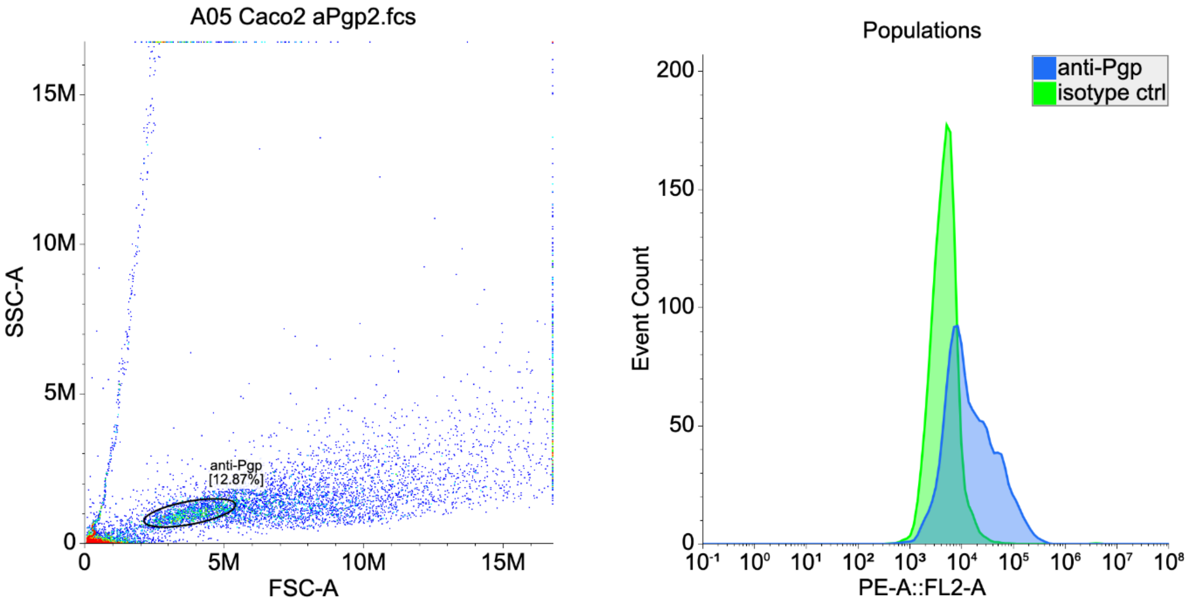

Figure S7. Flow cytometry analysis of Caco-2 cells.

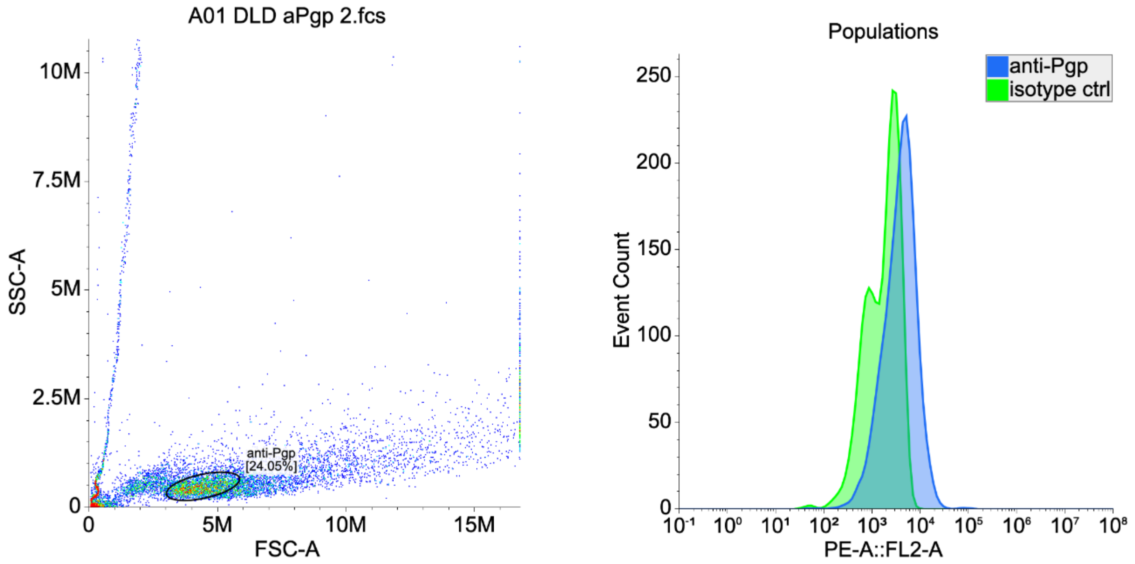

Figure S8. Flow cytometry analysis of DLD1 cells.

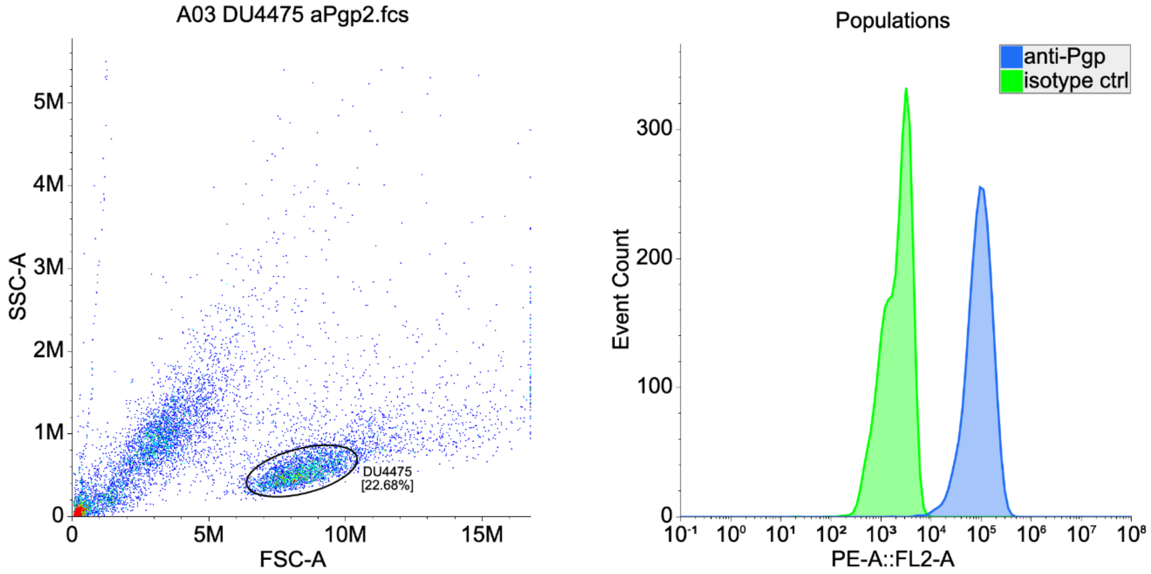

Figure S9. Flow cytometry analysis of DU4475 cells.

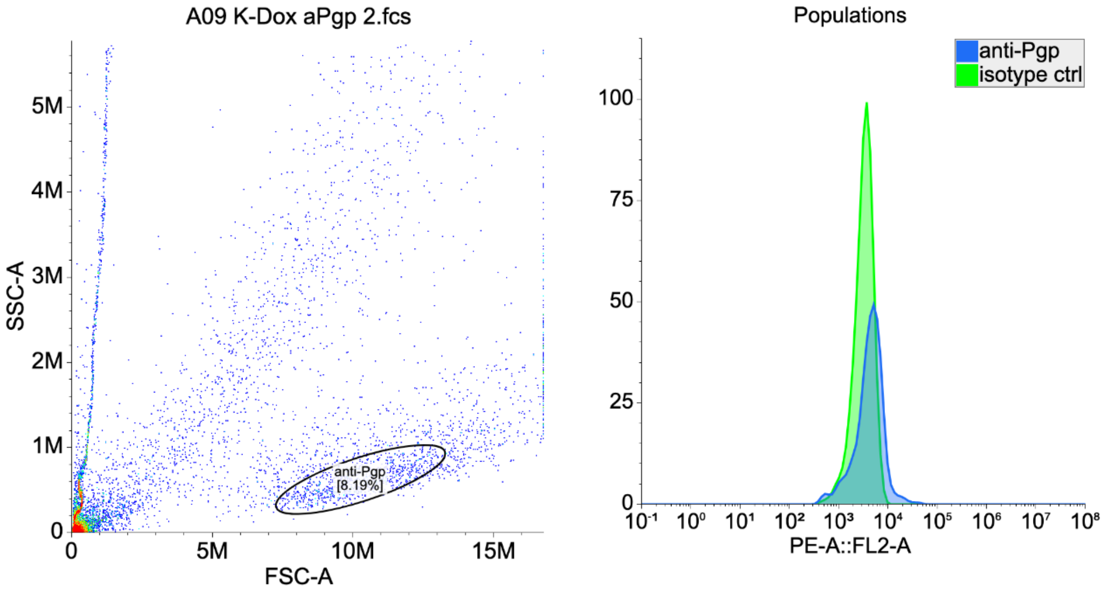

Figure S10. Flow cytometry analysis of K562/Dox cells.

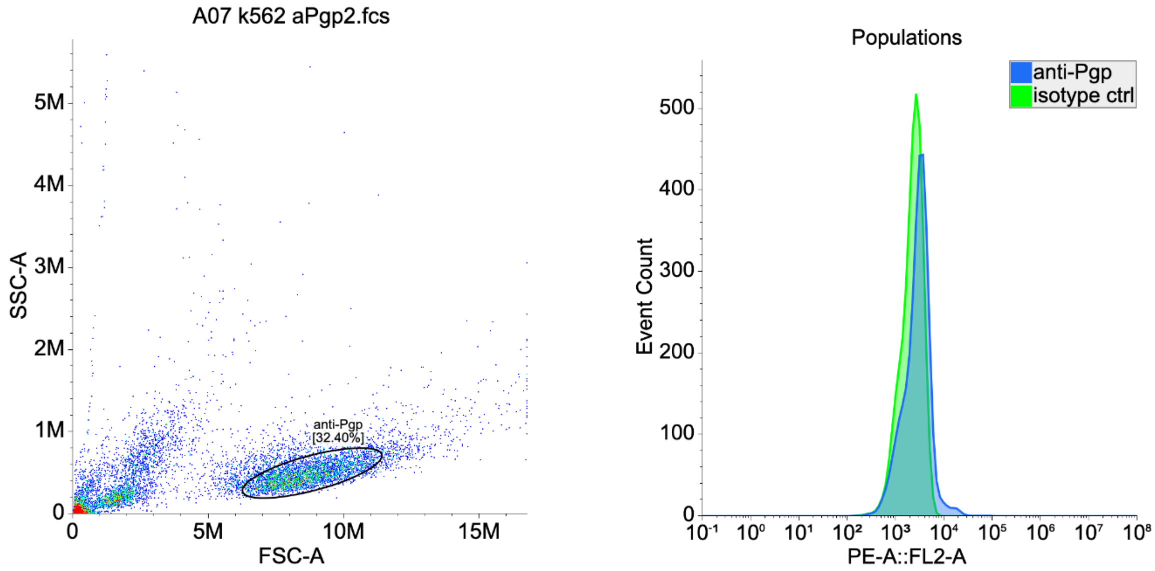

Figure S11. Flow cytometry analysis of K562 cells.

Western Blot

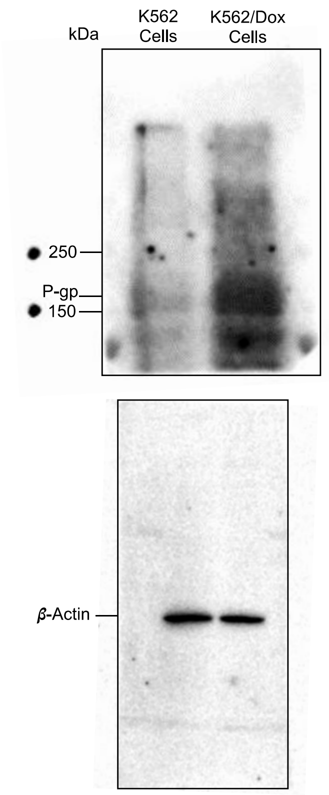

Figure S12. Western blot analysis of K562 and K562/Dox cells.

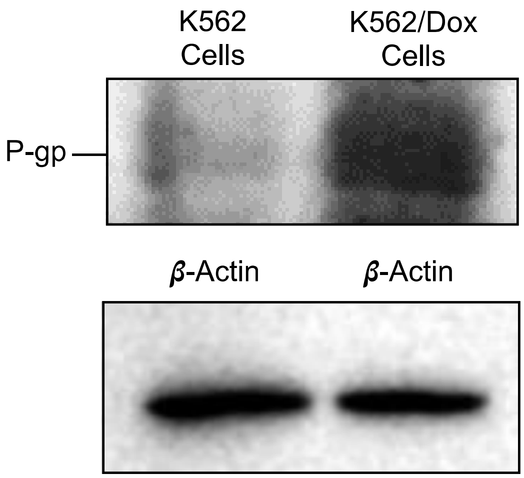

Figure S13. Magnified Western blot analysis of K562 and K562/Dox cells.

3. Supporting Chemistry Experimental Details

X-ray Crystal Structure for Computational Calculations

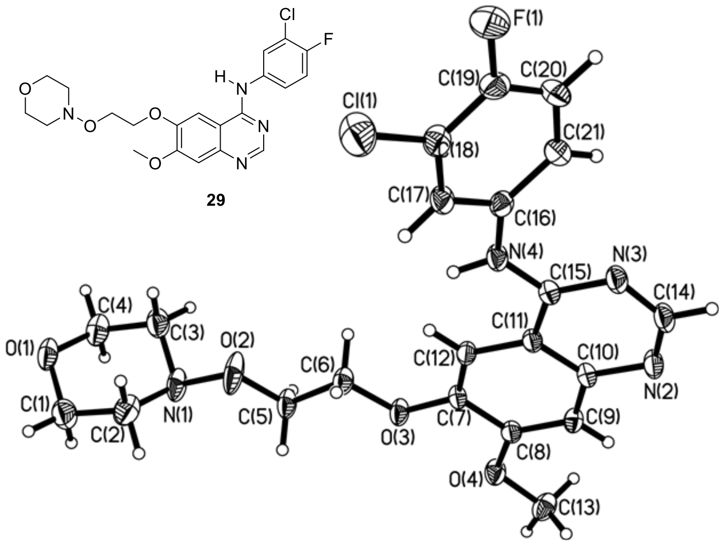

Figure S14. ORTEP drawing of compound **29** with 30% probability for thermal ellipsoids as described in Ref. 3. Crystallographic data for **29** has been deposited with the CCDC as entry 2270277 and was used to benchmark computational analysis on truncated derivatives (**5b-8b**, **11b-16b**).

Computational results are provided in Tables S21-S30. All geometries are in Cartesian Coordinates in Å calculated at the HF/cc-pVDZ level of theory.

Table S21. Cartesian Coordinates for **5b**

H	1.814829	1.527309	11.058995
C	3.054272	2.402309	9.560904
H	3.863286	0.934821	6.634140
Cl	1.000198	-0.991163	10.024562
C	5.267861	3.391609	4.193479
H	8.363574	4.221683	10.287473
H	9.123034	3.655119	4.361234
O	9.536522	3.422999	8.786184
O	10.703666	3.691149	6.411904
C	2.012215	0.256227	9.351634
O	2.405346	-1.016779	7.370281
Cl	3.337816	3.834701	10.529836
C	5.147819	3.315827	6.603479
N	1.934855	3.321765	5.149298
N	6.555522	3.472036	4.167379
C	4.502440	3.326225	5.385994
N	4.451202	3.305488	7.814406
H	4.821583	3.909525	8.515751
C	3.408725	1.102422	7.597202
C	2.254602	1.404934	10.081925
C	2.591241	0.101611	8.098364
C	3.647294	2.277492	8.301541
H	4.747857	3.392442	3.243646
H	8.448510	2.440277	10.245094
H	6.859147	3.248806	8.739602
C	11.462762	2.510816	6.575862
H	9.859341	3.388874	10.758379
H	1.314919	-1.942087	5.953390
C	7.352503	3.357318	7.789297
C	3.065868	3.328052	5.275960
C	8.631898	3.565386	5.317858
C	9.363181	3.555882	6.457830
C	1.304570	-0.991131	6.478612
C	8.709084	3.441264	7.732572
C	6.568952	3.385289	6.595871
C	7.213357	3.478059	5.349986
H	0.366224	-0.885177	7.023278
H	1.404591	-0.172810	5.763880
C	9.007945	3.364119	10.084626
H	12.506281	2.806515	6.508555
H	11.276696	2.054129	7.546796
H	11.236340	1.796329	5.781519

Table S22. Cartesian Coordinates for **6b**

Cl	1.168216	2.459068	11.396058
C	3.150204	2.193277	9.564598
H	3.406318	0.433911	6.694379
F	-0.095224	0.631509	9.462275
C	5.415231	2.658003	4.676872
H	10.856905	5.388128	4.776986
C	9.756123	3.038429	10.045167
O	10.074797	3.408257	8.734821
O	10.855285	4.128540	6.415772
C	1.136563	0.979828	9.127922
H	1.103926	-0.185484	7.354314
H	3.698600	2.875143	10.198750
C	5.600267	2.375348	6.911797
H	8.947344	3.653042	10.448560
N	6.612977	3.094965	4.471636
N	4.858157	2.292741	5.842741
N	5.063014	2.036054	8.126051
H	5.572168	2.351424	8.920054
C	2.981552	0.826288	7.601239
C	1.863442	1.830474	9.932698
C	1.693013	0.482525	7.965893
C	3.722666	1.693312	8.402281
H	4.765651	2.577044	3.814134
H	11.399812	3.719512	4.464291
H	7.553050	2.551454	8.922350
C	6.964077	2.805507	6.840375
C	7.407655	3.169616	5.570215
C	9.590393	3.709764	6.464402
H	9.039938	3.910727	4.407635
C	8.741692	3.632196	5.404410
H	9.476113	1.983969	10.106846
C	9.142469	3.310280	7.775069
H	12.424985	4.829754	5.399089
C	11.400741	4.535991	5.188667
C	7.868762	2.873405	7.943999
H	10.654047	3.202801	10.633930

Table S23. Cartesian Coordinates for **7b**

Cl	1.972375	0.950637	11.833215
C	3.432232	1.550096	9.647680
H	2.800765	1.626888	6.347749
H	0.201099	0.609086	9.608577
C	5.482946	2.555275	4.500464
H	11.508741	3.377468	4.504739
C	9.560058	3.295998	10.037348
O	9.960084	3.521884	8.715378
O	10.886646	3.977710	6.382166
C	1.164486	0.920117	9.235245
H	0.596411	0.916745	7.180464
F	4.435388	1.775890	10.498709
C	5.561741	2.399726	6.756649
C	9.607079	3.603821	6.403137
N	6.705635	2.925259	4.322026
N	4.862954	2.283123	5.661087
N	4.982121	2.136101	7.964844
H	5.566991	2.245297	8.759667
C	2.636900	1.495624	7.400120
C	2.204325	1.153712	10.123260
C	1.397511	1.095121	7.884174
C	3.683071	1.731093	8.286851
H	4.866631	2.445829	3.616753
H	12.527475	4.534259	5.388724
C	6.938042	2.802489	6.720806
H	11.012279	5.083862	4.640709
H	9.161559	3.640729	4.311771
H	8.750179	3.968413	10.330283
C	7.452417	3.053025	5.450117
C	8.807581	3.458144	5.312462
C	9.079960	3.345465	7.719044
H	9.245552	2.260688	10.188769
C	11.504504	4.255391	5.153310
H	7.410385	2.769693	8.851038
C	7.786624	2.958352	7.859604
H	10.430535	3.497515	10.655127

Table S24. Cartesian Coordinates for **8b**

H	11.459384	2.096566	5.651355
C	3.109185	2.346029	9.605406
H	3.925095	0.723022	6.773784
Cl	0.715175	-0.796309	10.063645
C	5.357470	3.366722	4.333173
H	8.799271	2.357984	10.231004
C	6.734671	3.342785	6.689363
O	9.771294	3.470084	8.783431
O	10.834241	3.924077	6.386703
C	1.877766	0.321192	9.408320
O	2.235050	-1.053699	7.488396
F	3.432484	3.445388	10.294903
C	5.316854	3.183525	6.743456
H	9.185253	3.872878	4.389872
N	6.634408	3.529059	4.266762
C	4.633234	3.205501	5.543668
N	4.690192	3.074342	7.974592
H	5.088709	3.611766	8.712772
C	3.439621	0.964360	7.706210
C	2.195254	1.467062	10.124270
C	2.496515	0.066404	8.193929
C	3.749683	2.128612	8.390561
H	4.804263	3.374798	3.402061
N	2.064447	3.131389	5.345319
C	9.507368	3.699347	6.470965
C	3.196784	3.159733	5.459203
H	10.163808	3.373338	10.740560
C	11.672031	2.789505	6.468312
C	1.188194	-0.947221	6.540613
H	7.114995	3.119174	8.813020
C	7.328353	3.529456	5.430841
C	7.561784	3.309536	7.852819
H	8.603215	4.127017	10.355198
C	8.907821	3.484543	7.757749
H	1.135219	-1.907309	6.034551
C	8.735966	3.710227	5.357378
H	0.237622	-0.741864	7.033758
H	1.398311	-0.158038	5.817118
C	9.293096	3.322247	10.093427
H	12.691443	3.154461	6.375588
H	11.554408	2.280535	7.423850
H	1.730781	1.678526	11.074791

Table S25. Cartesian Coordinates for **11b**

Cl	3.539934	1.282077	11.956379
C	3.922326	2.341319	9.505467
H	3.519405	1.187305	6.371692
H	2.812487	-0.780334	10.110741
C	5.512885	3.495514	3.948809
C	11.379041	2.359450	7.003031
C	8.590487	3.513004	7.811966
O	9.307037	3.518584	8.942941
O	10.717618	3.567390	6.688316
C	3.125777	0.086427	9.549785
H	8.018483	2.696960	10.337607
F	4.336659	3.436843	10.144626
C	5.162040	3.508131	6.333627
C	6.575062	3.505042	6.469510
N	6.799720	3.494052	4.051871
C	4.636707	3.512070	5.065496
N	4.308304	3.546678	7.463046
H	4.568072	4.262756	8.110369
C	3.533927	1.206424	7.449784
C	3.522563	1.232083	10.220210
C	3.141574	0.085423	8.164953
C	3.931908	2.359140	8.116629
H	5.090250	3.492478	2.952029
C	3.213263	3.552626	4.834836
H	9.429349	3.594458	10.937004
N	2.098117	3.562011	4.609553
H	2.831660	-0.800627	7.629208
C	8.760308	3.506576	5.397230
H	6.640768	3.505132	8.635578
C	7.342443	3.504787	5.290937
H	8.042739	4.478203	10.266197
C	9.372937	3.513432	6.605126
C	8.648841	3.574492	10.182209
H	9.348284	3.519330	4.492480
C	7.232121	3.508093	7.736469
H	12.441009	2.588964	7.025401
H	11.068716	1.981590	7.975979
H	11.187199	1.606615	6.235335

Table S26. Cartesian Coordinates for **12b**

H	7.897738	4.980610	10.064909
C	3.057725	3.096225	9.192279
H	3.931486	1.189361	6.544797
H	1.642364	0.143991	10.009567
C	5.662535	3.066691	3.851218
C	9.375403	3.401634	6.688455
C	6.590857	3.463336	6.391454
O	9.187493	3.777243	8.989606
O	10.715207	3.426254	6.841685
C	2.212816	0.862812	9.440118
H	11.126630	1.404932	6.717756
H	7.793766	3.236013	10.418417
C	5.187900	3.495539	6.175831
H	3.456404	2.754055	12.268977
N	6.941865	3.042862	4.026114
C	4.730484	3.301542	4.894843
N	4.294598	3.767976	7.229894
H	4.530723	4.577432	7.763839
C	3.496683	1.471370	7.490304
C	2.348717	2.153180	9.928368
C	2.791717	0.535414	8.226587
C	3.630418	2.778574	7.955674
H	5.292089	2.909434	2.846085
H	9.207967	4.170940	10.949032
H	1.899664	2.554770	13.102671
C	2.505410	2.218602	12.265393
C	8.530969	3.617596	7.832295
C	11.309736	2.260077	7.372089
N	2.229414	3.394630	4.271371
H	9.462551	3.065572	4.604403
C	8.827714	3.218412	5.463528
C	7.418318	3.243917	5.275779
C	3.326231	3.360582	4.571309
H	6.537741	3.795028	8.529583
H	2.693073	1.147068	12.359416
C	7.179813	3.644939	7.678851
Cl	3.223407	4.717504	9.836683
O	1.768502	2.502972	11.097205
C	8.468540	4.055243	10.161950
H	12.377748	2.455348	7.419971
H	10.935025	2.045995	8.371986
H	2.689334	-0.468253	7.837271

Table S27. Cartesian Coordinates for **13b**

H	7.558994	5.004376	10.121018
C	3.257042	2.999167	9.150343
H	3.379735	1.294944	6.252389
H	1.777403	0.125096	10.054763
C	5.721582	3.082037	3.782632
C	9.237855	3.372970	6.864615
C	6.480690	3.470214	6.380097
O	8.901360	3.774953	9.144128
O	10.564674	3.378943	7.107604
C	2.279145	0.844735	9.424900
H	10.950687	1.350373	7.027105
H	7.406744	3.264087	10.478119
C	5.095416	3.518070	6.070036
H	4.156381	3.091196	11.924537
N	6.984819	3.037305	4.044673
C	4.724197	3.331716	4.761488
N	4.125361	3.791096	7.060266
H	4.365866	4.577883	7.627402
C	3.184611	1.519048	7.288890
C	2.629711	2.073047	9.963489
C	2.562655	0.578453	8.094618
C	3.538710	2.758169	7.813832
H	5.417940	2.929117	2.754665
H	8.794726	4.183811	11.097510
H	2.988374	2.541732	13.143039
C	3.408203	2.334024	12.162172
C	8.321817	3.611545	7.946688
C	11.103547	2.208342	7.685491
N	2.272568	3.458986	3.969230
H	9.460081	3.021762	4.793078
C	8.771135	3.189318	5.606571
C	7.378328	3.234394	5.324172
C	3.345662	3.410727	4.343941
H	6.290690	3.828464	8.507382
H	3.876306	1.347059	12.170455
C	6.984322	3.655884	7.702592
F	3.631099	4.180473	9.668516
O	2.334522	2.372096	11.249625
C	8.108936	4.072157	10.262687
H	12.169038	2.387182	7.803193
H	10.659640	2.008982	8.659811
H	2.286202	-0.376509	7.669802

Table S28. Cartesian Coordinates for **14b**

H	8.423030	3.677866	10.296722
C	2.602182	2.717782	9.028997
H	4.109086	0.978714	6.534600
Cl	0.383546	-0.614454	9.193347
C	5.369995	3.765045	4.119254
C	8.738410	3.147082	7.674099
H	9.226617	3.667263	4.347745
O	9.542920	2.938394	8.727093
O	10.770788	3.350505	6.404056
C	1.578322	0.569895	8.738810
H	2.381939	-0.647510	7.198462
H	8.344724	1.910800	10.062854
C	5.196598	3.475024	6.510788
C	7.379163	3.184135	7.710071
N	6.660681	3.732184	4.111375
C	4.579362	3.657558	5.290351
N	4.466980	3.413642	7.693249
H	4.942684	3.769968	8.493918
C	3.423902	1.220617	7.332573
C	1.645023	1.792820	9.418103
C	2.451232	0.301448	7.709357
C	3.494941	2.433051	7.994814
H	4.869213	3.903815	3.169098
H	9.827183	2.663793	10.685788
H	0.504203	4.042826	10.413417
C	0.730009	3.214020	11.086818
C	3.153096	3.821755	5.169854
C	11.415621	2.094408	6.439170
H	-0.064730	3.122322	11.821239
H	6.865012	3.015194	8.640500
C	6.620091	3.410244	6.521044
C	7.292238	3.565096	5.296817
C	9.422047	3.334001	6.424751
H	1.673078	3.407333	11.602219
N	2.032853	3.969308	5.034452
C	8.713785	3.526171	5.286675
H	2.654901	3.684551	9.503818
O	0.761714	1.991890	10.400223
C	8.989430	2.790534	10.006232
H	12.482988	2.296818	6.409524
H	11.174562	1.552754	7.352792
H	11.136121	1.495382	5.569736

Table S29. Cartesian Coordinates for **15b**

H	7.966488	4.423041	10.295865
C	3.690536	2.410830	9.428432
H	3.719926	1.163869	6.286797
Cl	2.390773	-1.271865	10.260566
C	5.552573	3.535891	3.909852
C	8.577874	3.524138	10.194660
H	3.092124	1.307705	11.170467
O	9.259203	3.509381	8.969315
O	10.708366	3.589227	6.741637
C	2.964139	0.131603	9.390596
H	2.930426	-0.831291	7.484457
H	7.947078	2.640284	10.309597
C	5.158736	3.534578	6.289784
C	9.364569	3.536760	6.632710
N	6.837523	3.537847	4.033184
C	4.657225	3.543651	5.011636
N	4.289405	3.567760	7.401359
H	4.540011	4.265937	8.070744
C	3.595302	1.208776	7.357612
C	3.234411	1.296088	10.102711
C	3.144073	0.074925	8.029890
C	3.870008	2.381280	8.034437
H	5.146193	3.538152	2.906213
H	9.342920	3.522637	10.965793
N	2.128719	3.616600	4.504921
O	4.020050	3.583029	10.024659
C	3.238212	3.588035	4.755271
C	7.204378	3.518897	7.725392
C	6.570716	3.530403	6.446795
H	9.377971	3.565562	4.519861
C	8.561031	3.520612	7.824421
C	8.773906	3.543206	5.413804
C	11.362224	2.373404	7.039432
H	6.592027	3.503747	8.610689
C	7.358269	3.540613	5.282460
H	12.424382	2.599339	7.083972
H	11.035904	1.974605	7.998906
H	11.180954	1.637746	6.252733
C	3.697551	3.796153	11.374896
H	3.972759	4.824101	11.594168
H	2.628793	3.663284	11.549468
H	4.260482	3.127566	12.028965

Table S30. Cartesian Coordinates for **16b**

H	8.018821	4.405489	10.378811
N	3.180163	2.726422	9.293434
H	3.929571	1.014940	6.498750
Cl	1.396169	-0.673237	10.298619
C	5.578197	3.383019	4.021147
C	9.397553	3.440373	6.733689
C	6.602231	3.440458	6.555715
O	9.298086	3.447855	9.070118
O	10.740904	3.493649	6.838099
C	2.290125	0.527036	9.412273
H	11.213318	1.534058	6.382101
H	7.989690	2.623112	10.442968
C	5.191247	3.450938	6.399967
H	1.727583	4.123517	11.015370
N	6.863852	3.386652	4.142402
C	4.684936	3.427297	5.122448
N	4.339349	3.529048	7.515366
H	4.547510	4.248140	8.177263
C	3.522552	1.223389	7.473763
C	2.506754	1.806384	9.939678
C	2.811828	0.254117	8.170079
C	3.670790	2.457527	8.087386
H	5.170125	3.358670	3.018599
H	9.393574	3.515764	11.065297
H	1.666862	3.359778	12.625453
C	2.176465	3.376105	11.666692
C	8.596924	3.445389	7.928489
C	11.396704	2.283195	7.155394
N	2.156846	3.533119	4.622130
H	9.404745	3.426248	4.620920
C	8.803376	3.423511	5.516911
C	7.387102	3.421366	5.389007
C	3.267087	3.486330	4.865802
H	6.630566	3.433897	8.719823
H	3.230103	3.611101	11.808016
C	7.240036	3.445425	7.832616
H	2.663630	-0.724350	7.735920
O	2.014158	2.078523	11.134089
C	8.623836	3.500214	10.299284
H	12.458642	2.510982	7.192362
H	11.073664	1.901503	8.122798

Bond Angles About Nitrogen

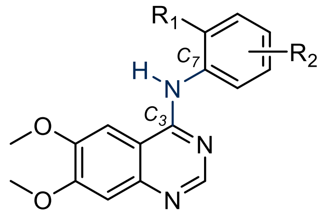

6b-7b

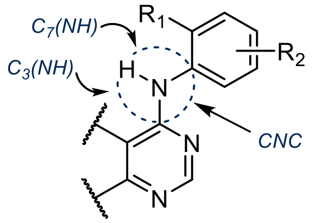

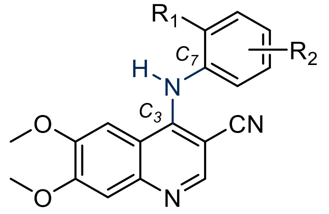

5b, **8b**, **11b-14b**

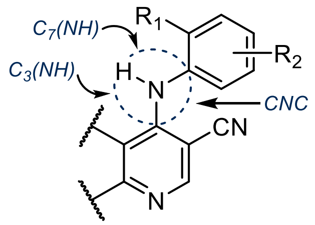

Figure S15. Bond angle definitions for compounds **5b-8b**, **11b-16b**.

Table S31. Computed bond angles around nitrogen.

Compound	C(7)NH Angle (°)	CNC Angle (°)	C(3)NH Angle (°)	Sum of Angles (°)
**6b**	114.0	127.5	115.4	356.9
**7b**	113.3	130.8	115.9	360
**5b**	114.6	126.6	114.8	356
**8b**	114.4	128.5	115.8	358.7
**11b**	111.9	120.6	112.3	344.8
**12b**	114.2	123.6	114.0	351.8
**13b**	112.0	121.3	112.6	345.9
**14b**	113.9	125.0	114.6	353.5
**15b**	111.2	121.2	112.9	345.2
**16b**	112.5	125.2	116.1	353.8

Percent (%) nitrogen pyramidalization can be estimated according to the following equation:
Percent (%) nitrogen planarization = ((Sum of bond angles at nitrogen) - 328.5 / 31.5) *100
Percent (%) nitrogen pyramidalization = 100 - Percent (%) nitrogen planarization
Where, 328.5 is sum of bond angles (°) at nitrogen if fully pyramidal (sp^3) with bond angles of 109.5 °, and 31.5 is the difference in bond angles (°) between a fully planar nitrogen (sum of bond angles = 360 °) and a fully pyramidal nitrogen.

pK_a Calculations

Table S32. Calculated pK_a values using Jaguar[4] Software.[a]

**Compound**	**N-H pK_a (in DMSO)**	**N-H pK_a (in H_2O)**
**6b**	19.4	12.2
**7b**	19.4	12.1
**5b**	15.8	9.2
**8b**	16.0	9.6
**11b**	17.3	10.5
**12b**	18.7	10.9
**13b**	17.9	10.7
**14b**	17.8	10.7
**15b**	19.4	11.8
**16b**	16.6	9.5

[a]For an in-depth explanation regarding pK_a calculations, please refer to the "Computational Studies" section of the manuscript.

Intramolecular Hydrogen Bond NMR Study on *N*-(3-chloro-2-fluorophenyl)-7-methoxy-6-(2-((4-methylpiperazin-1-yl)oxy)ethoxy)quinazolin-4-amine (**7a**)

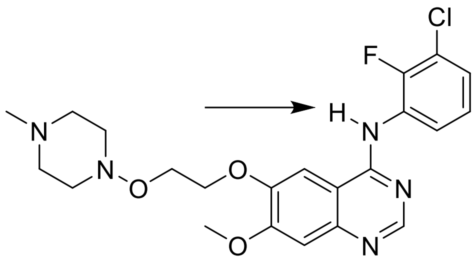

7a

An NMR experiment was performed according to Abraham *et al.*[5] to quantify A_{NMR} values which correlate with intramolecular hydrogen bonding. Briefly, two samples were prepared at 5 mM in $CDCl_3$ and DMSO-D_6 and the difference in chemical shift of the aniline N-H proton was used in the following equation:

$A_{NMR} = 0.0065 + 0.133\Delta\delta$

Where, $\Delta\delta = \delta$ (DMSO-D_6) - δ ($CDCl_3$)

$\Delta\delta = \delta$ (9.60 ppm) - δ (7.31 ppm)

$\Delta\delta = 2.29$ ppm

$A_{NMR} = 0.0065 + 0.133\ (2.29)$

$A_{NMR} = 0.31$

According to Abraham *et al.* if $A_{NMR} > 0.16$, the N-H group is not part of an intramolecular hydrogen bond. Thus, with an A_{NMR} value of 0.31, the N-H group is not engaged in an intramolecular hydrogen bond.

Intramolecular Hydrogen Bond NMR Study on 4-((4-chloro-2-fluoro-5-methoxyphenyl)amino)-6-methoxy-7-(2-((4-methylpiperazin-1-yl)oxy)ethoxy)quinoline-3-carbonitrile (**8a**)

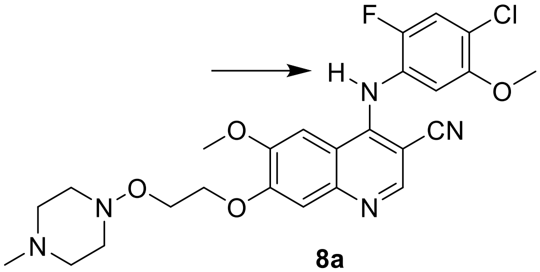

8a

An NMR experiment was performed according to Abraham *et al.*[5] to quantify A_{NMR} values which correlate with intramolecular hydrogen bonding. Briefly, two samples were prepared at 5 mM in $CDCl_3$ and DMSO-D_6 and the difference in chemical shift of the aniline N-H proton was used in the following equation:

$A_{NMR} = 0.0065 + 0.133\Delta\delta$

Where, $\Delta\delta = \delta$ (DMSO-D_6) - δ ($CDCl_3$)

$\Delta\delta = \delta$ (9.53 ppm) - δ (6.59 ppm)

$\Delta\delta = 2.94$ ppm

$A_{NMR} = 0.0065 + 0.133$ (2.94)

$A_{NMR} = 0.40$

According to Abraham *et al.* if $A_{NMR} > 0.16$, the N-H group is not part of an intramolecular hydrogen bond. Thus, with an A_{NMR} value of 0.40, the N-H group is not engaged in an intramolecular hydrogen bond.

Intramolecular Hydrogen Bond NMR Study on 4-((3-chloro-2-fluorophenyl)amino)-6-methoxy-7-(2-((4-methylpiperazin-1-yl)oxy)ethoxy)quinoline-3-carbonitrile (**11a**)

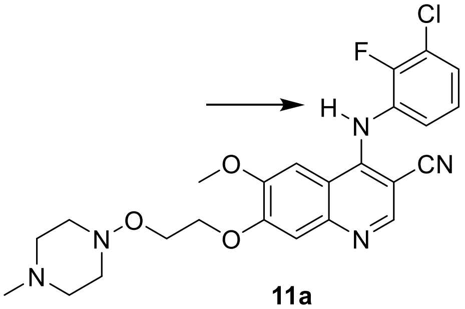

11a

An NMR experiment was performed according to Abraham *et al.*[5] to quantify A_{NMR} values which correlate with intramolecular hydrogen bonding. Briefly, two samples were prepared at 5 mM in $CDCl_3$ and DMSO-D_6 and the difference in chemical shift of the aniline N-H proton was used in the following equation:

$A_{\mathrm{NMR}} = 0.0065 + 0.133\Delta\delta$

Where, $\Delta\delta = \delta$ (DMSO-D_6) - δ ($CDCl_3$)

$\Delta\delta = \delta$ (9.57 ppm) - δ (6.63 ppm)

$\Delta\delta$ = 2.94 ppm

$A_{\mathrm{NMR}} = 0.0065 + 0.133$ (2.94)

$A_{\mathrm{NMR}} = 0.40$

According to Abraham *et al.* if $A_{\mathrm{NMR}} > 0.16$, the N-H group is not part of an intramolecular hydrogen bond. Thus, with an A_{NMR} value of 0.40, the N-H group is not engaged in an intramolecular hydrogen bond.

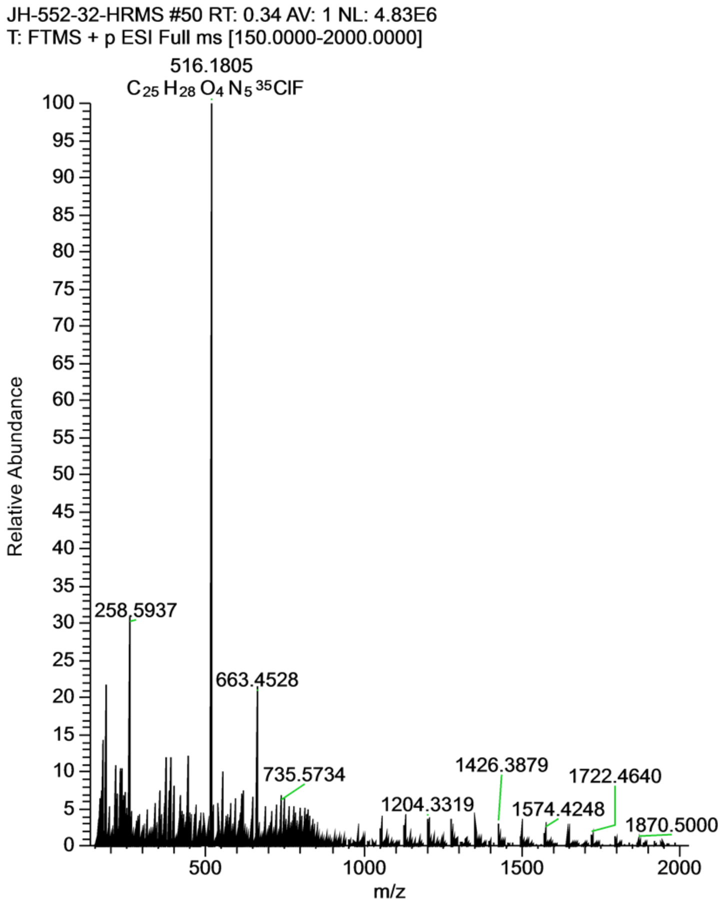

Figure S16. HRMS of 4-((4-chloro-2-fluoro-5-methoxyphenyl)amino)-6-methoxy-7-(2-((4-methylpiperazin-1-yl)oxy)ethoxy)quinoline-3-carbonitrile (**8a**).

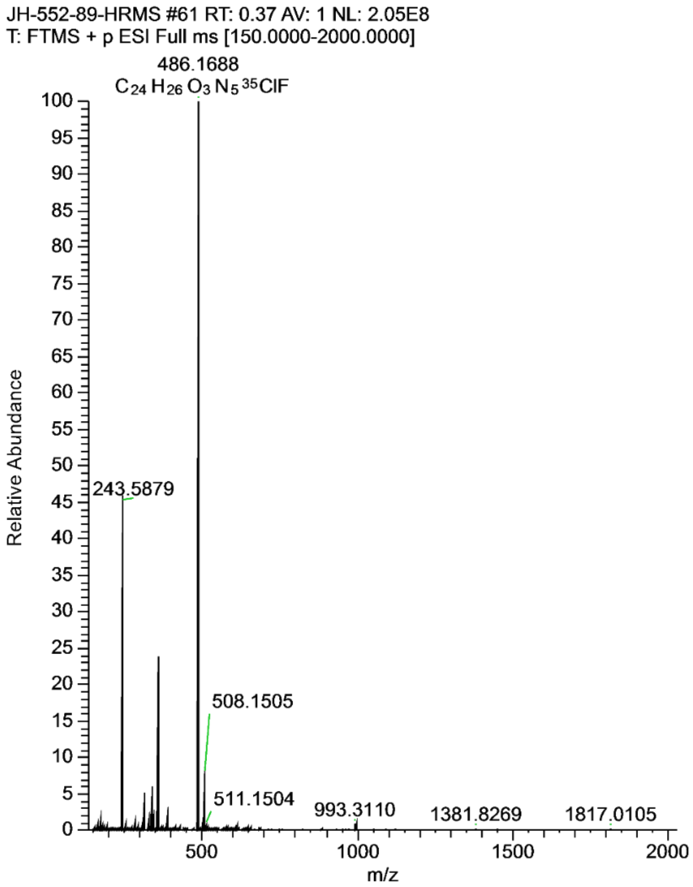

Figure S17. HRMS of 4-((3-chloro-2-fluorophenyl)amino)-6-methoxy-7-(2-((4-methylpiperazin-1-yl)oxy)ethoxy)quinoline-3-carbonitrile (**11a**).

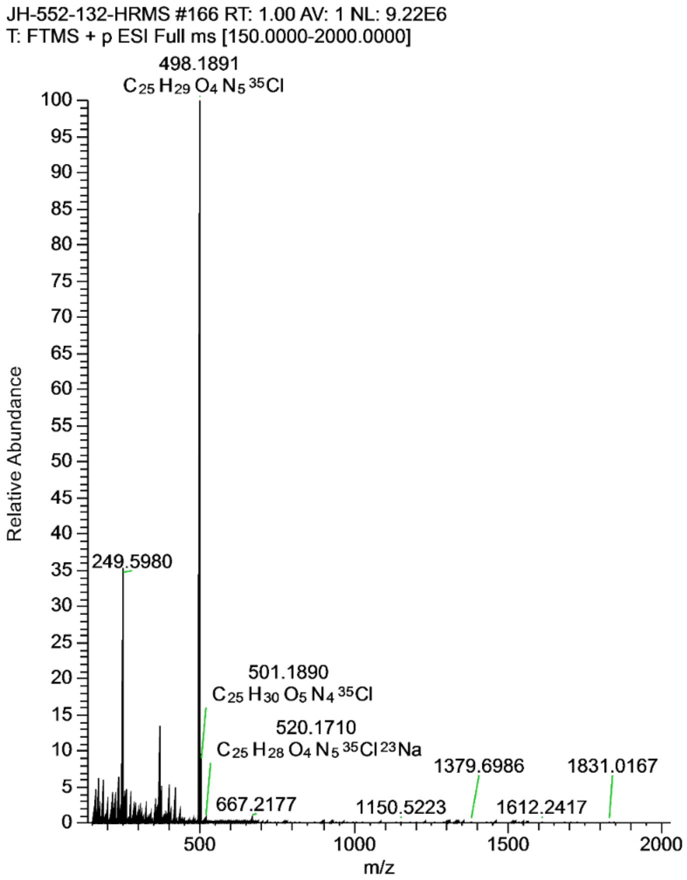

Figure S18. HRMS of 4-((2-chloro-3-methoxyphenyl)amino)-6-methoxy-7-(2-((4-methylpiperazin-1-yl)oxy)ethoxy)quinoline-3-carbonitrile (**12a**).

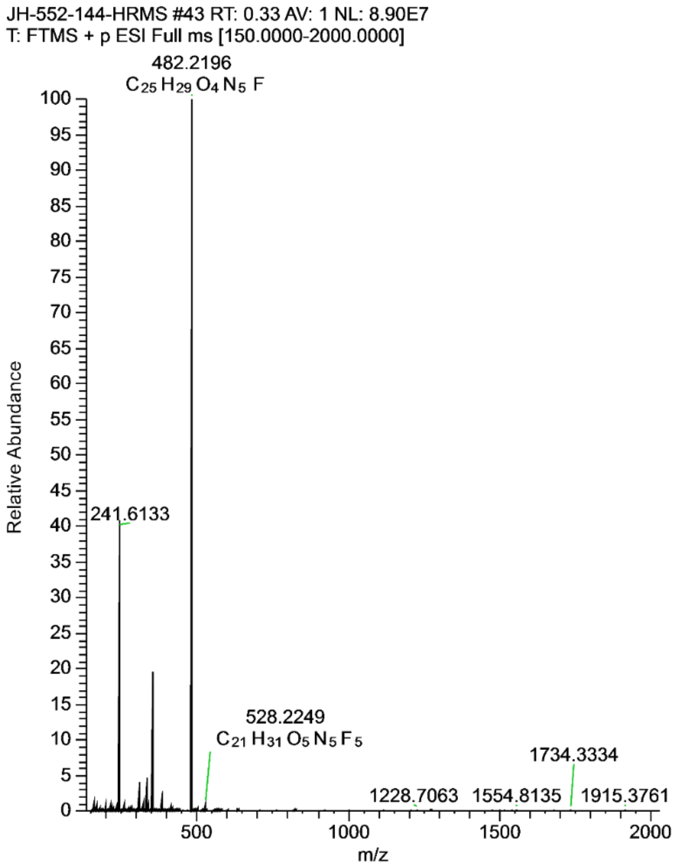

Figure S19. HRMS of 4-((2-fluoro-3-methoxyphenyl)amino)-6-methoxy-7-(2-((4-methylpiperazin-1-yl)oxy)ethoxy)quinoline-3-carbonitrile (**13a**).

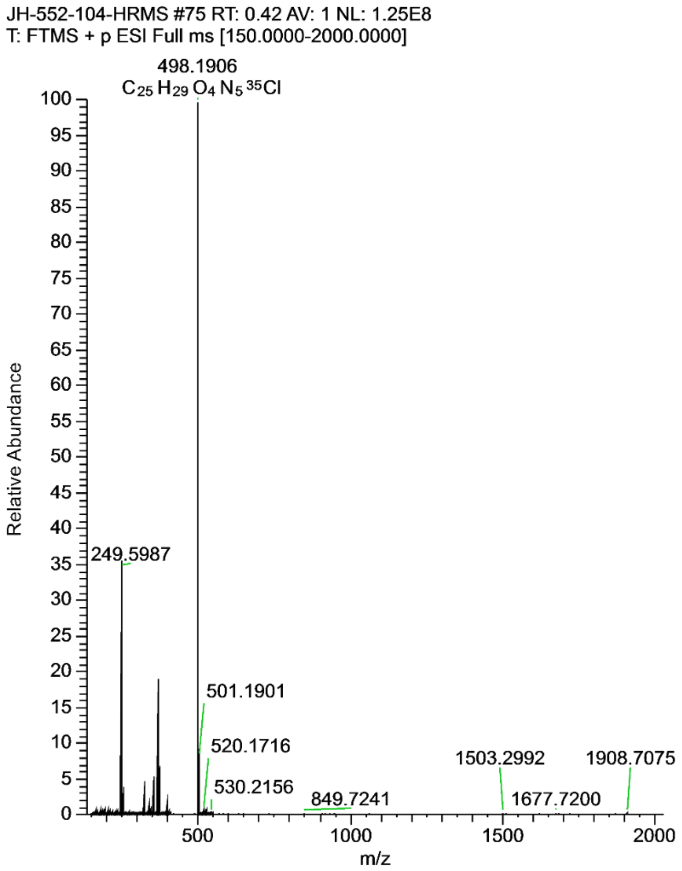

Figure S20. HRMS of 4-((4-chloro-3-methoxyphenyl)amino)-6-methoxy-7-(2-((4-methylpiperazin-1-yl)ethoxy)quinoline-3-carbonitrile (**14a**).

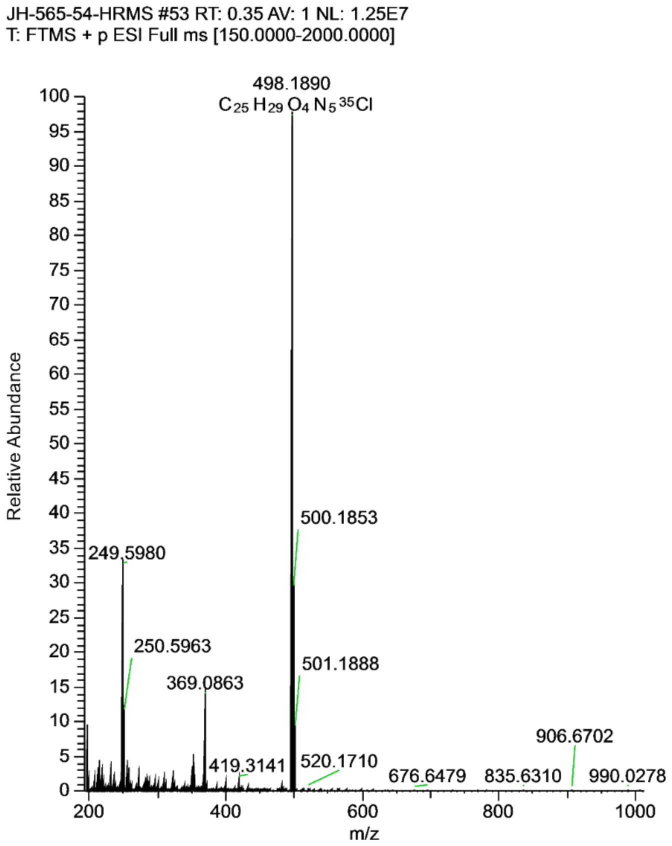

Figure S21. HRMS of 4-((4-chloro-2-methoxyphenyl)amino)-6-methoxy-7-(2-((4-methylpiperazin-1-yl)oxy)ethoxy)quinoline-3-carbonitrile (**15a**).

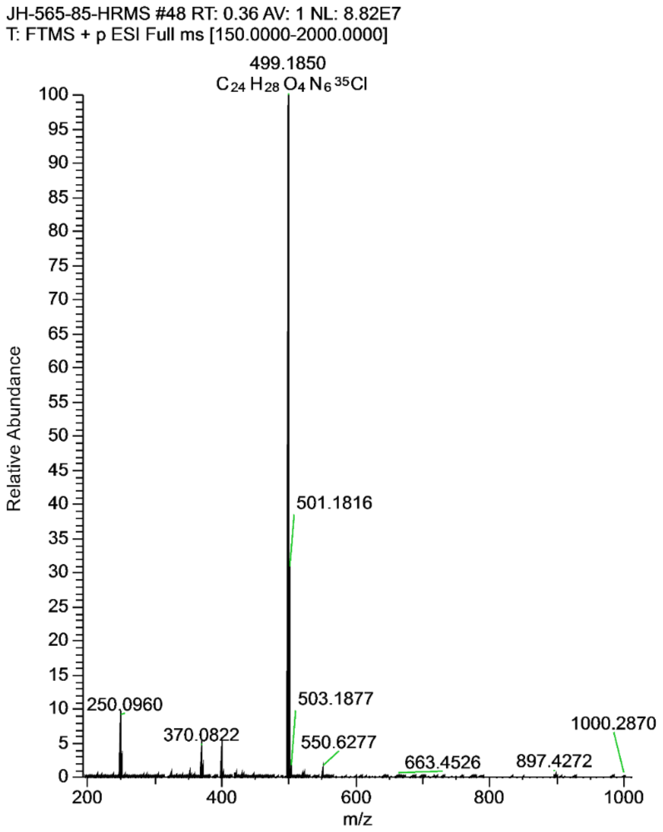

Figure S22. HRMS of 4-((5-chloro-6-methoxypyridin-2-yl)amino)-6-methoxy-7-(2-((4-methylpiperazin-1-yl)oxy)ethoxy)quinoline-3-carbonitrile (**16a**).

[1]**H NMR** (500 MHz, $CDCl_3$) spectrum of 4-chloro-2-fluoro-5-methoxyaniline (**18**).

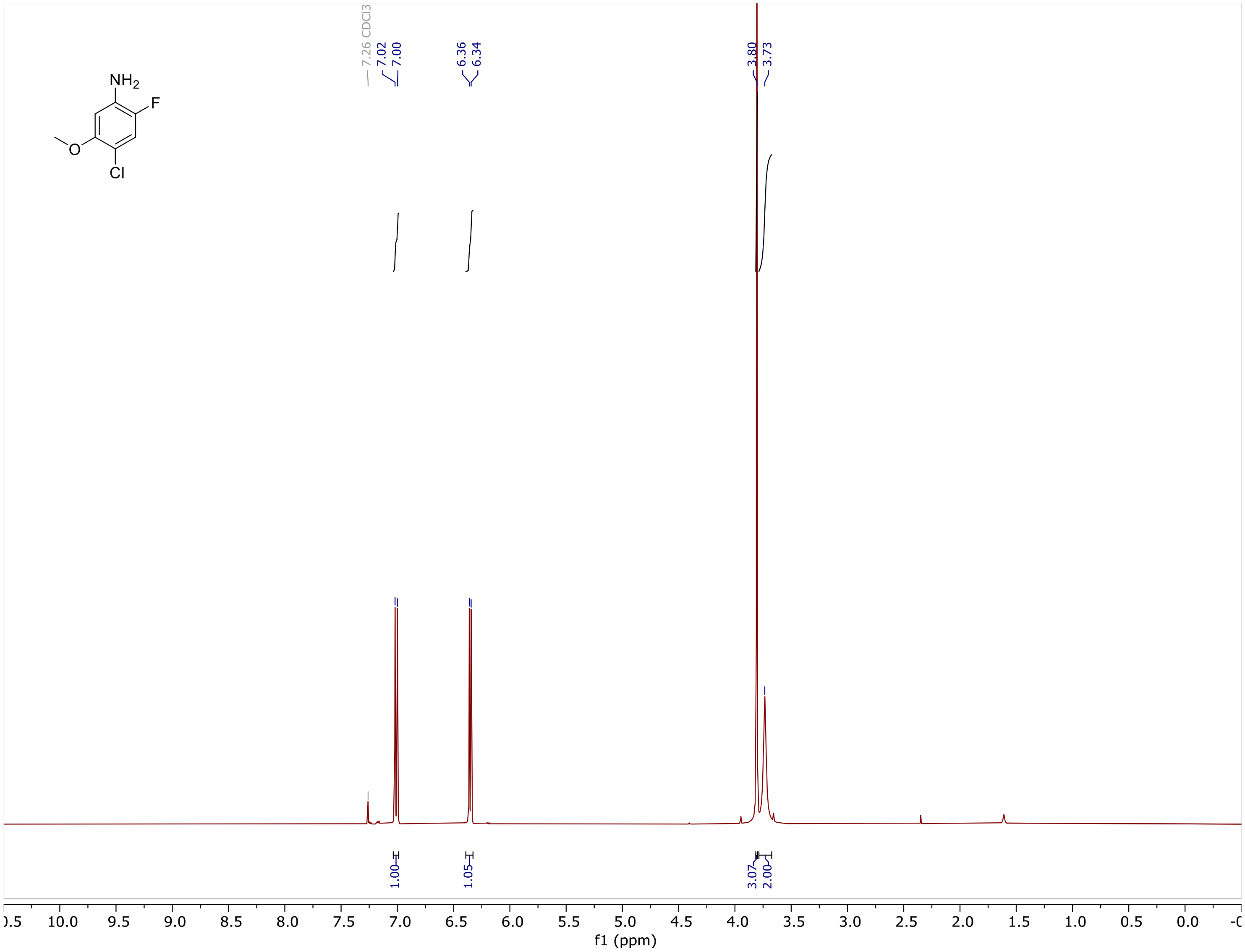

^{13}C NMR (126 MHz, $CDCl_3$) spectrum of 4-chloro-2-fluoro-5-methoxyaniline (**18**).

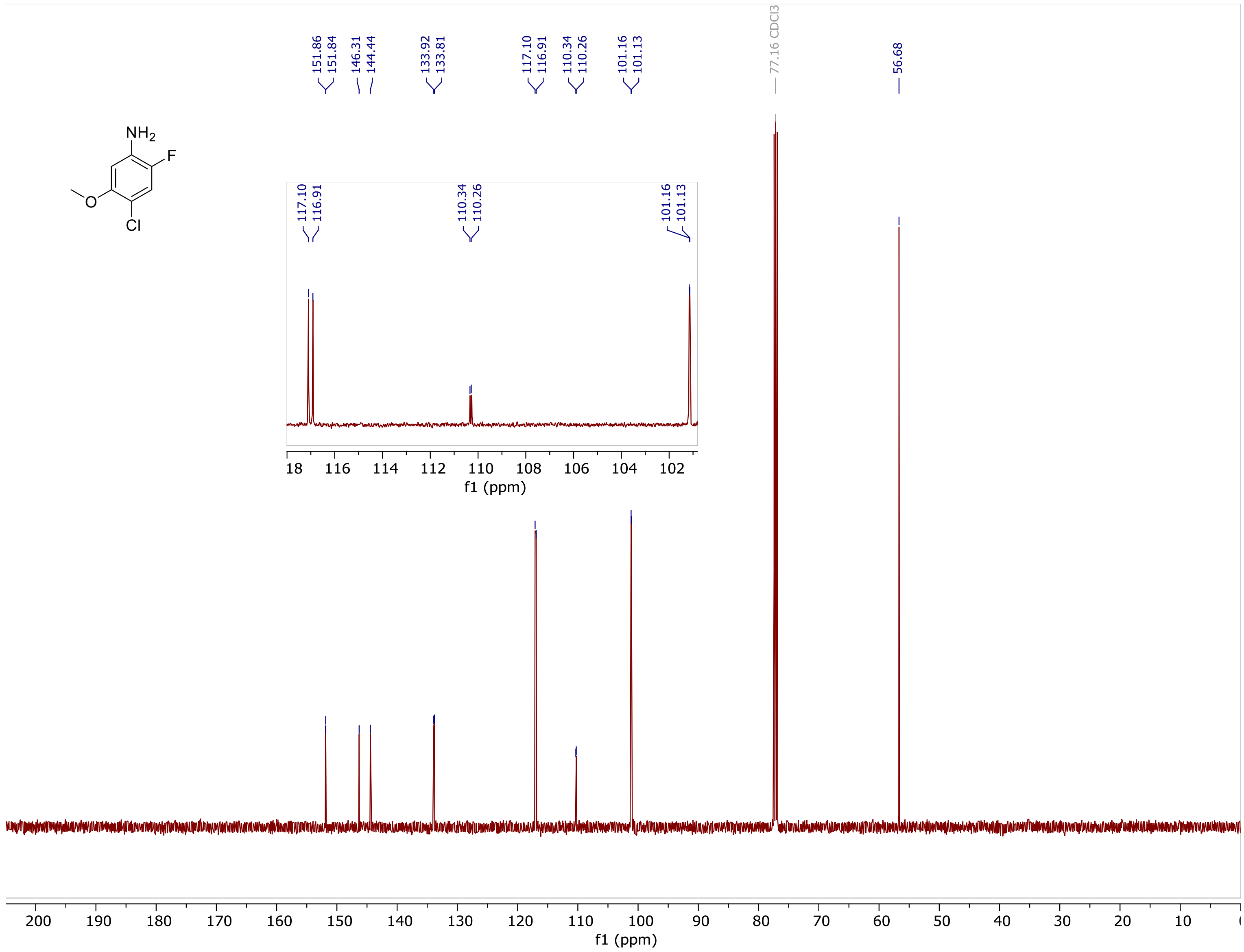

^{13}C {^{19}F} NMR (126 MHz, $CDCl_3$) spectrum of 4-chloro-2-fluoro-5-methoxyaniline (**18**).

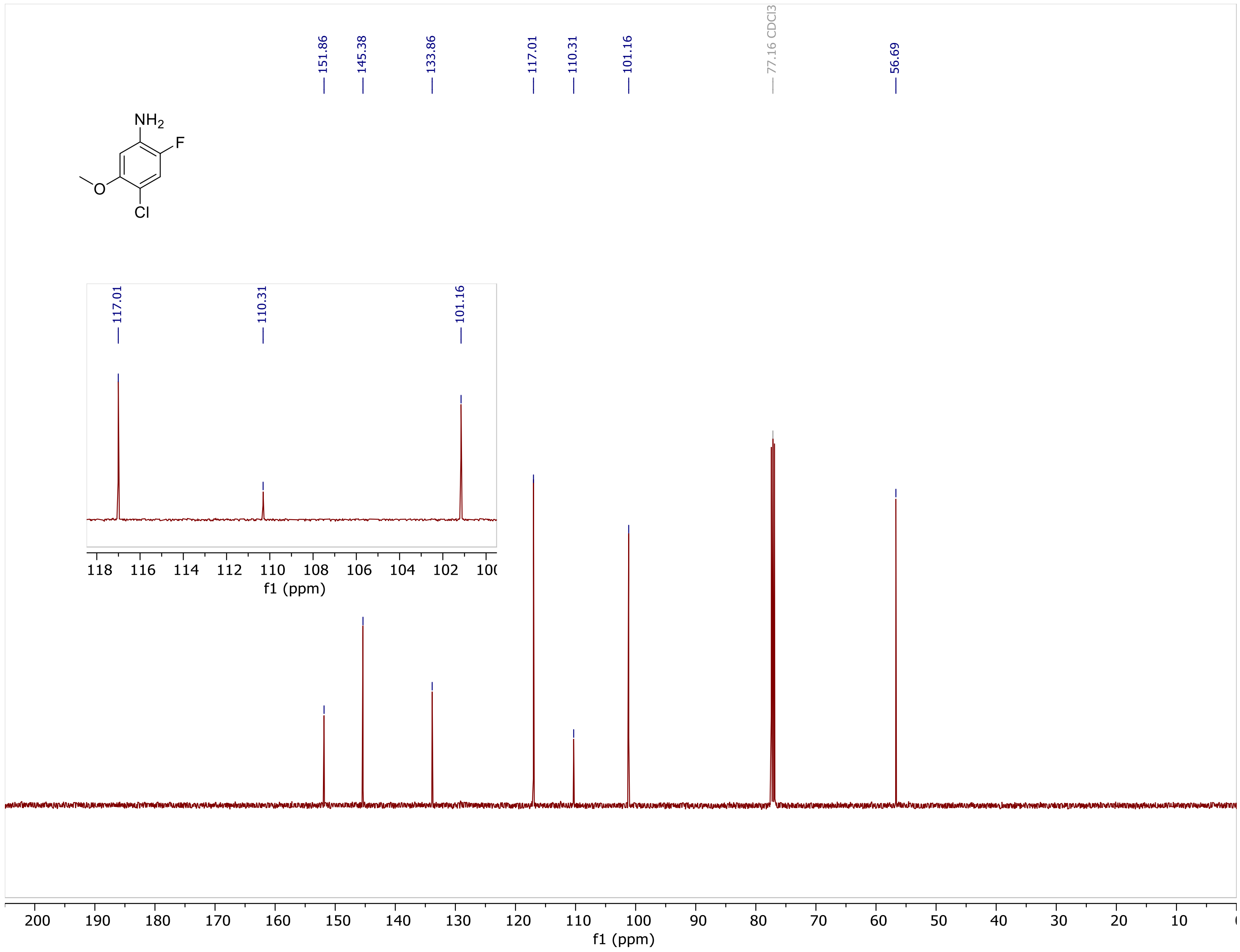

HSQC NMR (500 MHz, $CDCl_3$) spectrum of 4-chloro-2-fluoro-5-methoxyaniline (**18**).

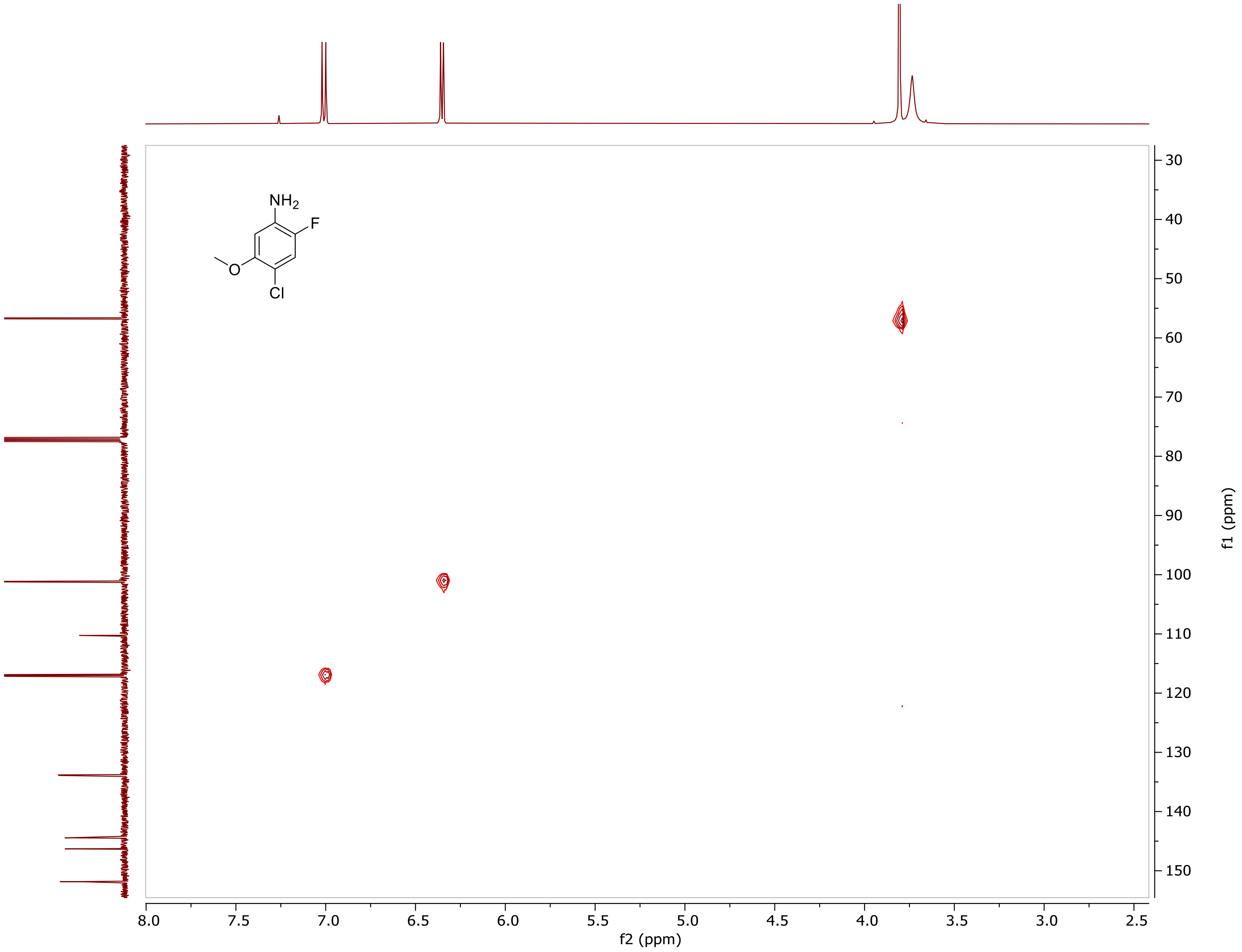

^{19}F {^{1}H} NMR (470 MHz, $CDCl_3$) spectrum of 4-chloro-2-fluoro-5-methoxyaniline (**18**).

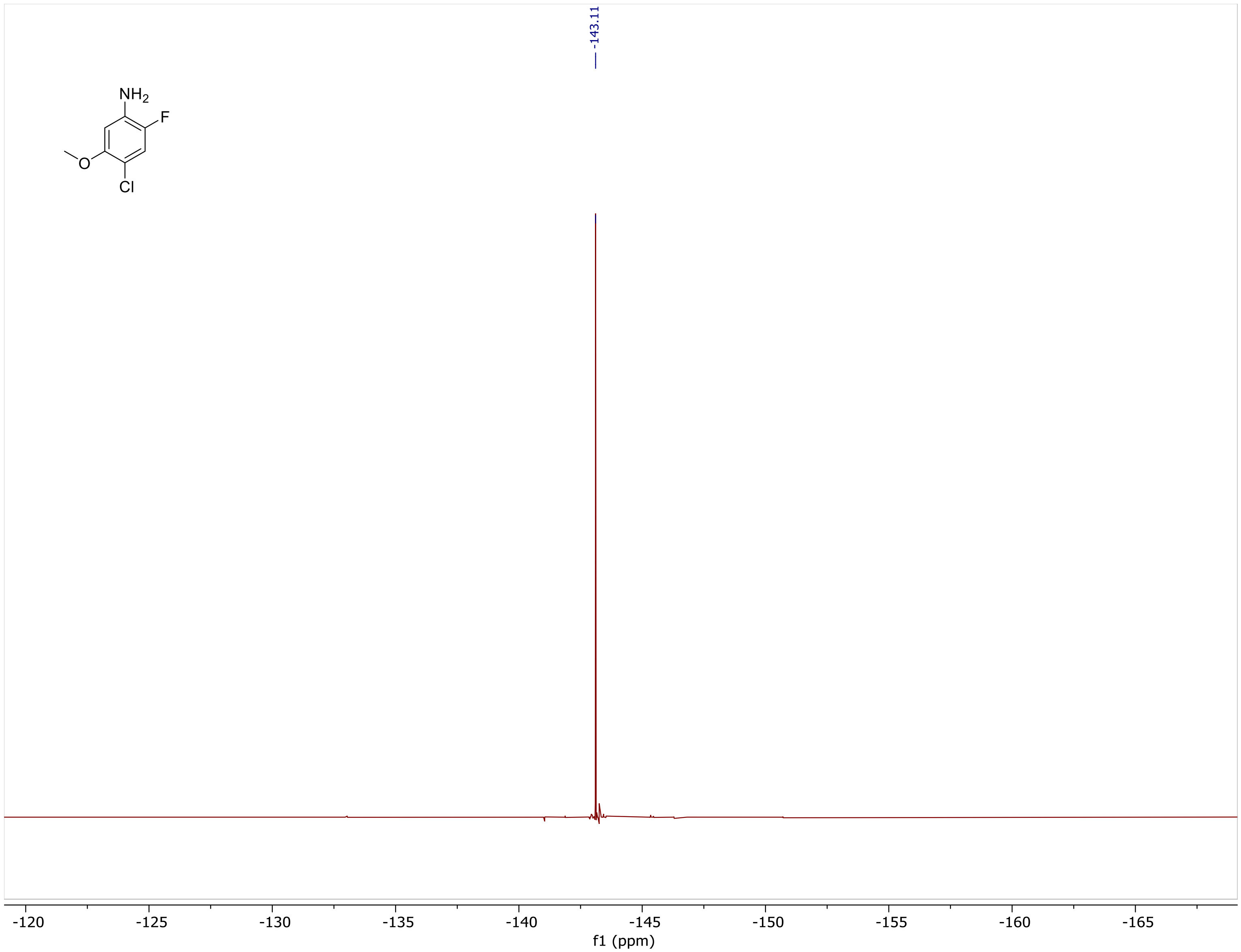

^{1}H NMR (500 MHz, $CDCl_3$) spectrum of 5-chloro-6-methoxypyridin-2-amine (**24**).

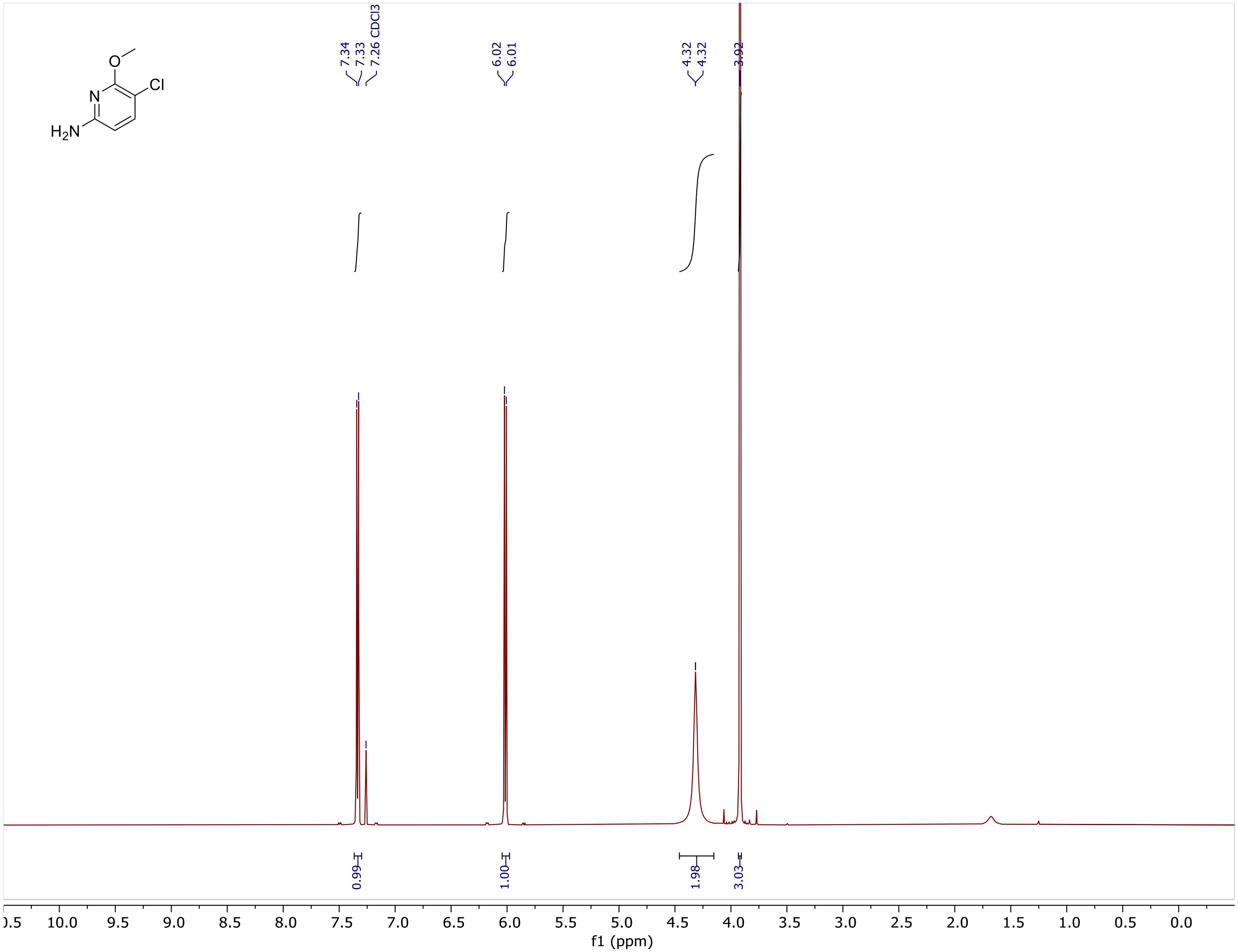

^{13}C NMR (126 MHz, $CDCl_3$) spectrum of 5-chloro-6-methoxypyridin-2-amine (**24**).

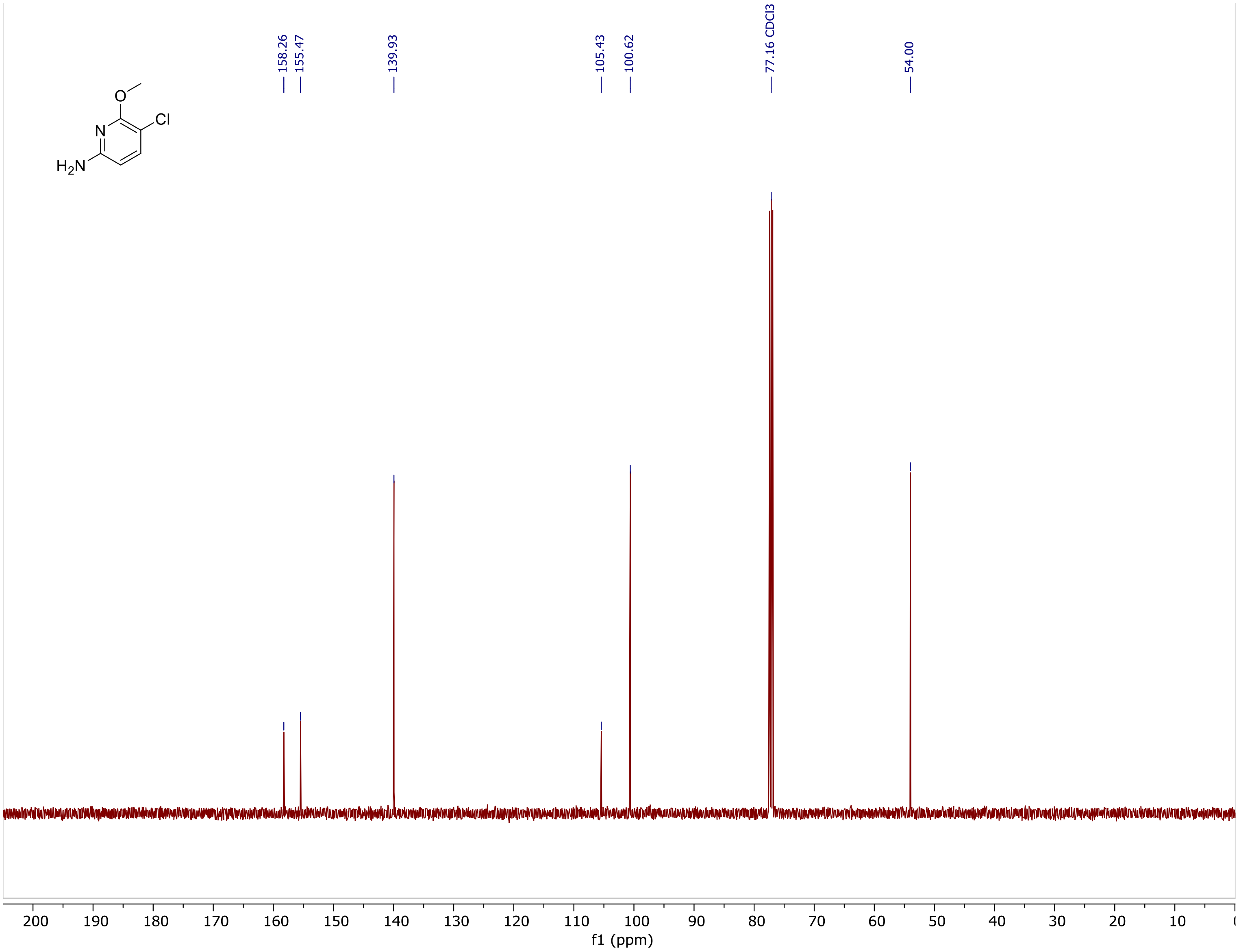

HSQC NMR (500 MHz, $CDCl_3$) spectrum of 5-chloro-6-methoxypyridin-2-amine (**24**).

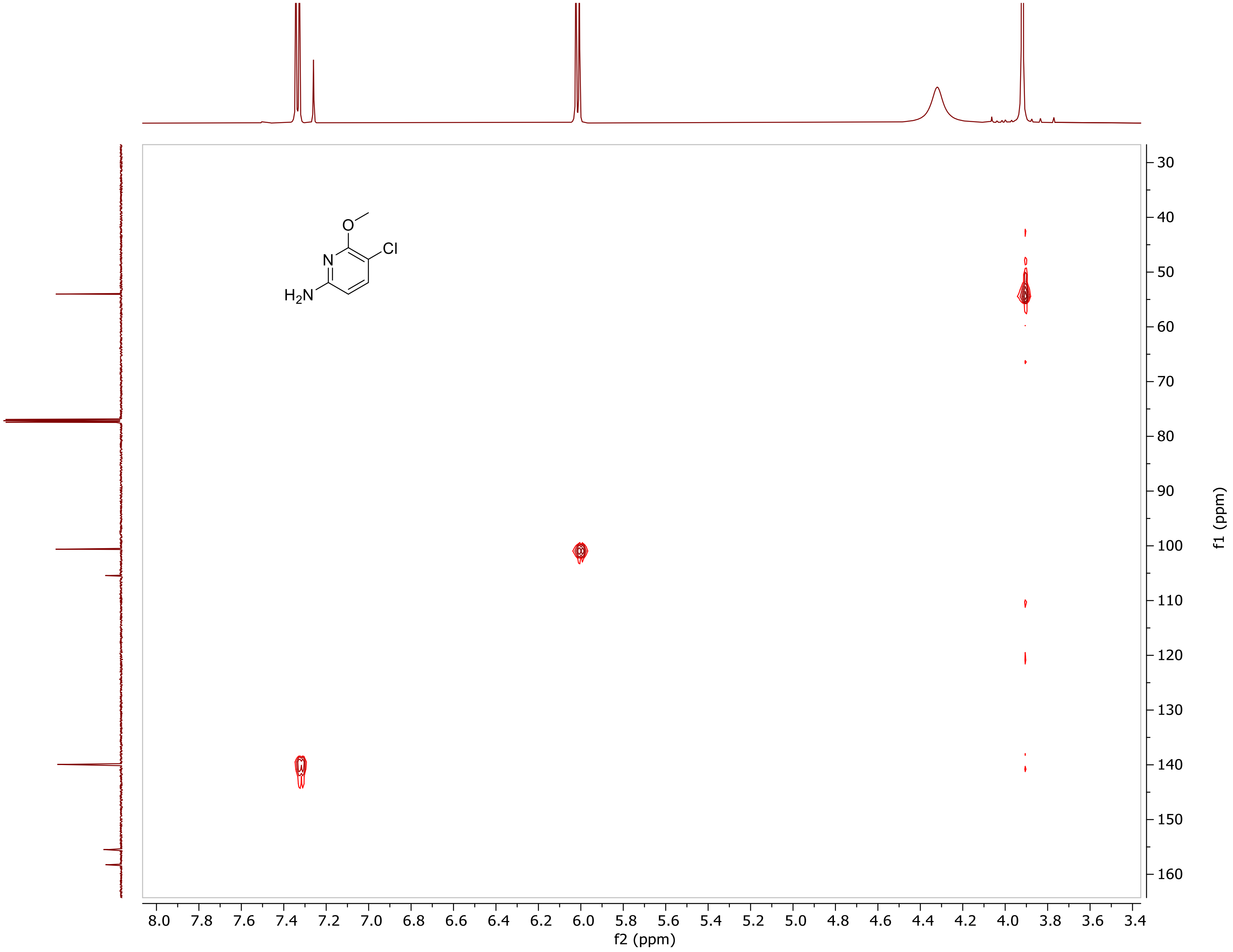

Expanded region of stacked a) **Selective 1D-NOESY** (500 MHz, $CDCl_3$) irradiated at 6.00 ppm and b)[1]**H NMR** (500 MHz, $CDCl_3$) spectrum of 5-chloro-6-methoxypyridin-2-amine (**24**). NOE interactions are shown with red arrows.

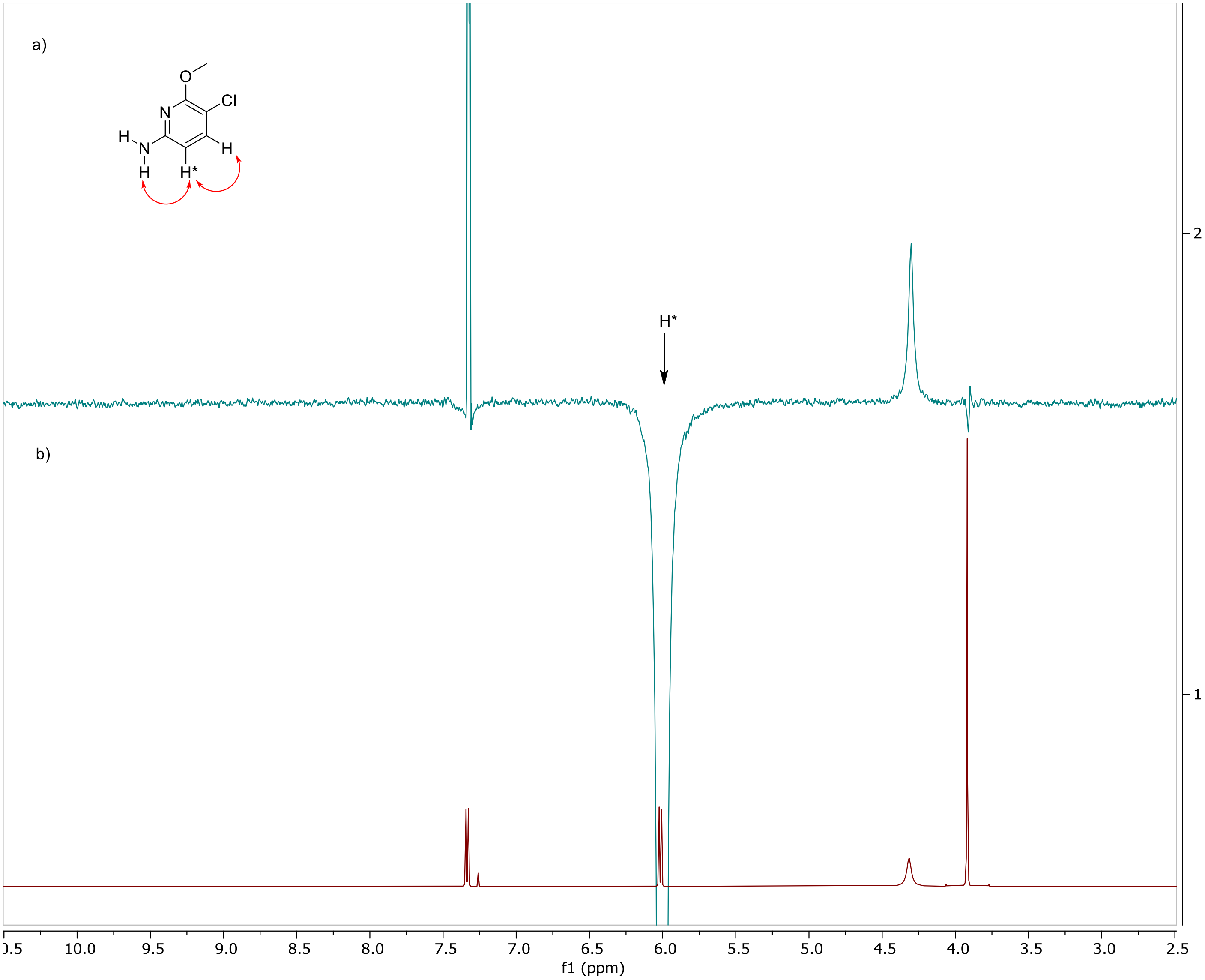

Expanded region of stacked a) **Selective 1D-NOESY** (500 MHz, $CDCl_3$) irradiated at 7.32 ppm and b)[1]**H NMR** (500 MHz, $CDCl_3$) spectrum of 5-chloro-6-methoxypyridin-2-amine (**24**). NOE interactions are shown with red arrows.

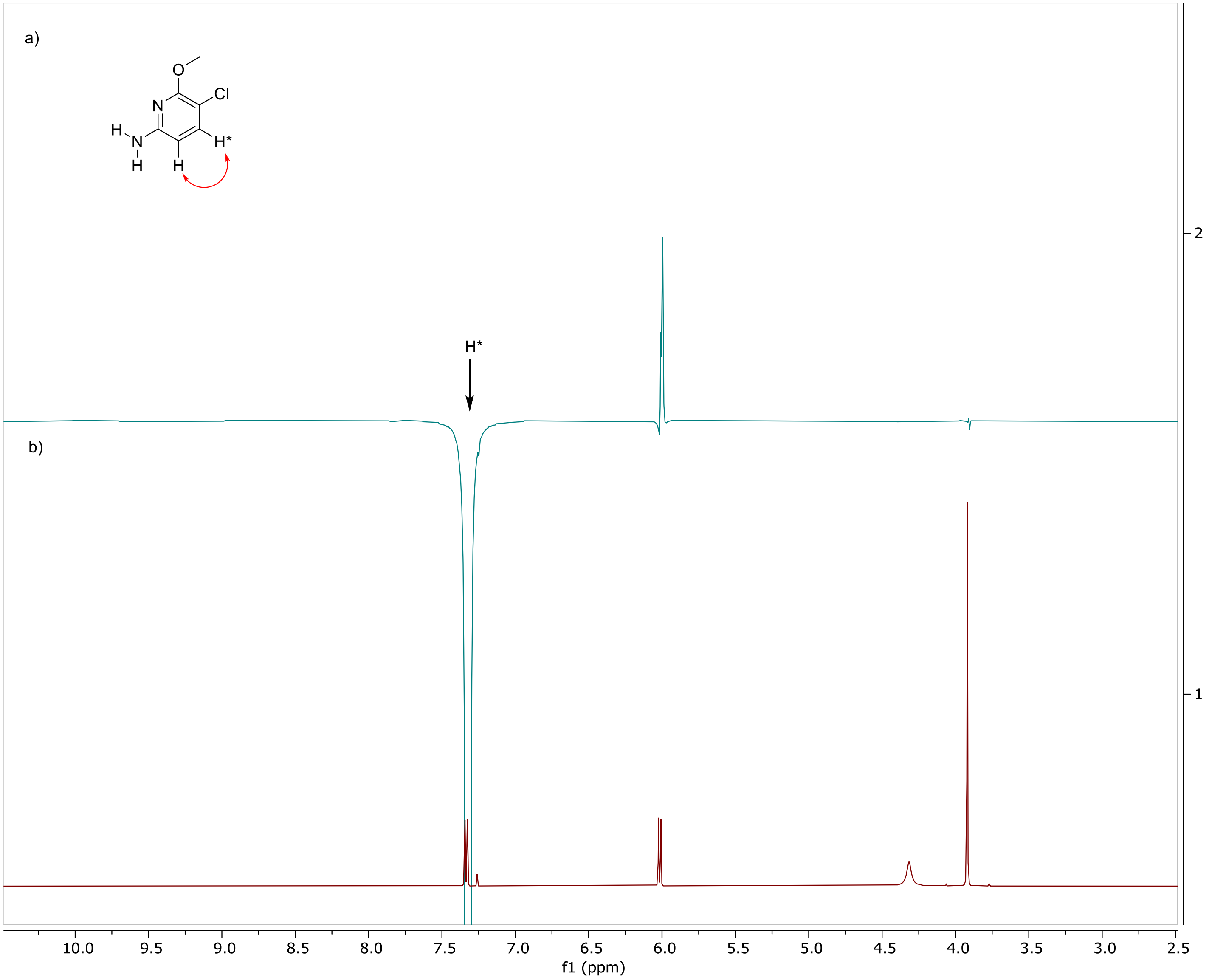

Expanded region of stacked a) **Selective 1D-NOESY** (500 MHz, $CDCl_3$) irradiated at 3.91 ppm and b) **^{1}H NMR** (500 MHz, $CDCl_3$) spectrum of 5-chloro-6-methoxypyridin-2-amine (**24**). NOE interactions are shown with red arrows.

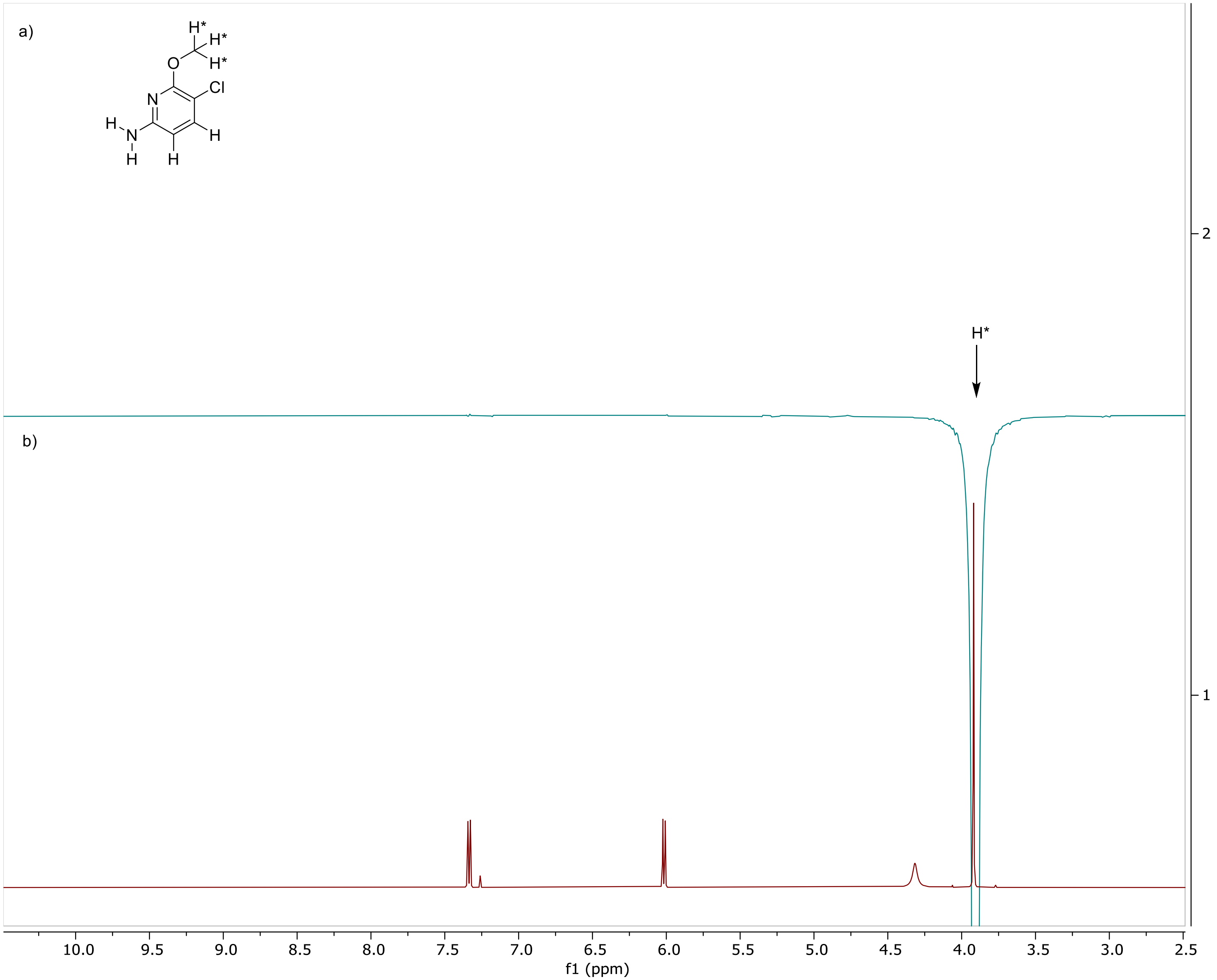

^{1}H NMR (500 MHz, $CDCl_3$) spectrum of 3-chloro-6-methoxypyridin-2-amine (**27**).

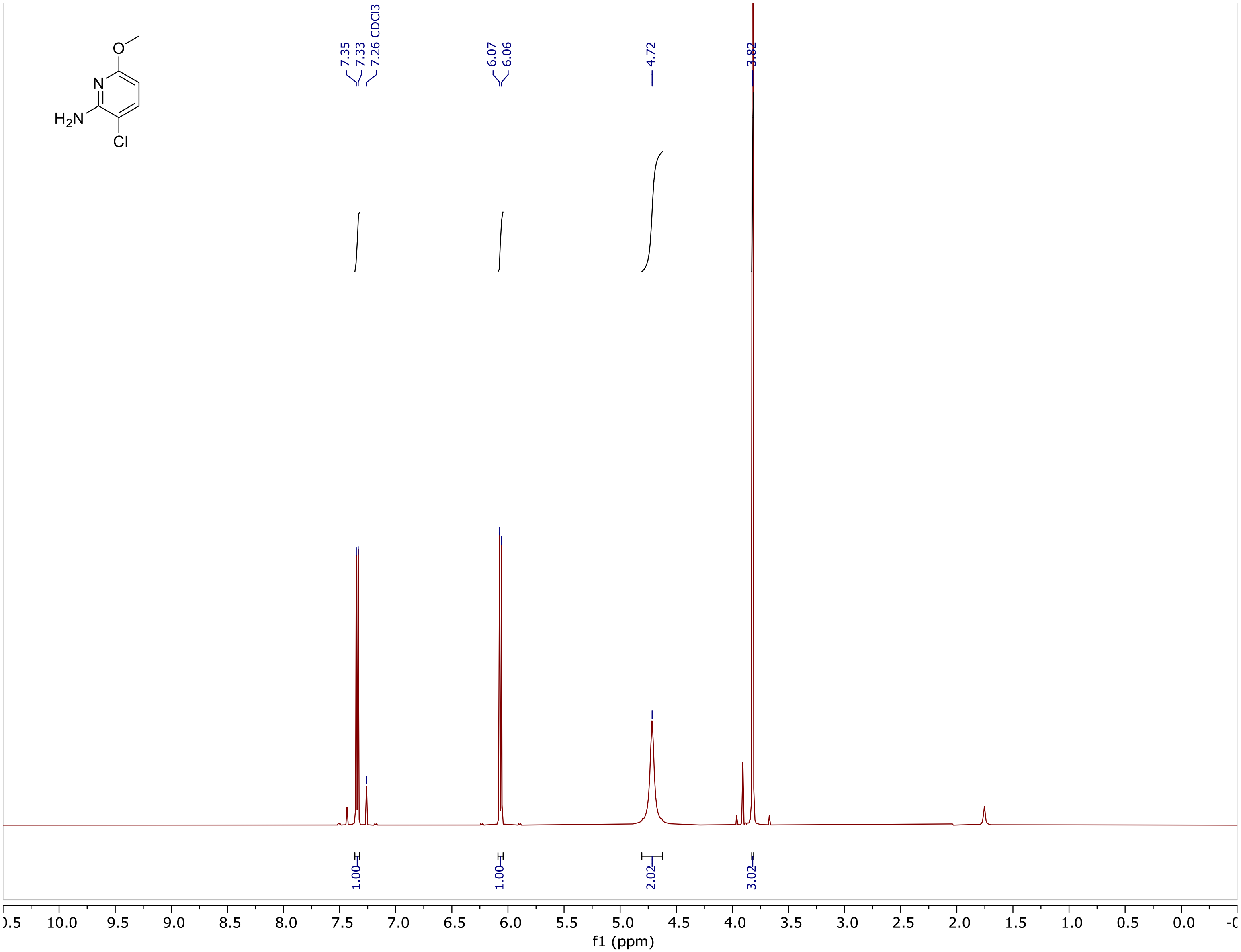

^{13}C NMR (126 MHz, $CDCl_3$) spectrum of 3-chloro-6-methoxypyridin-2-amine (**27**).

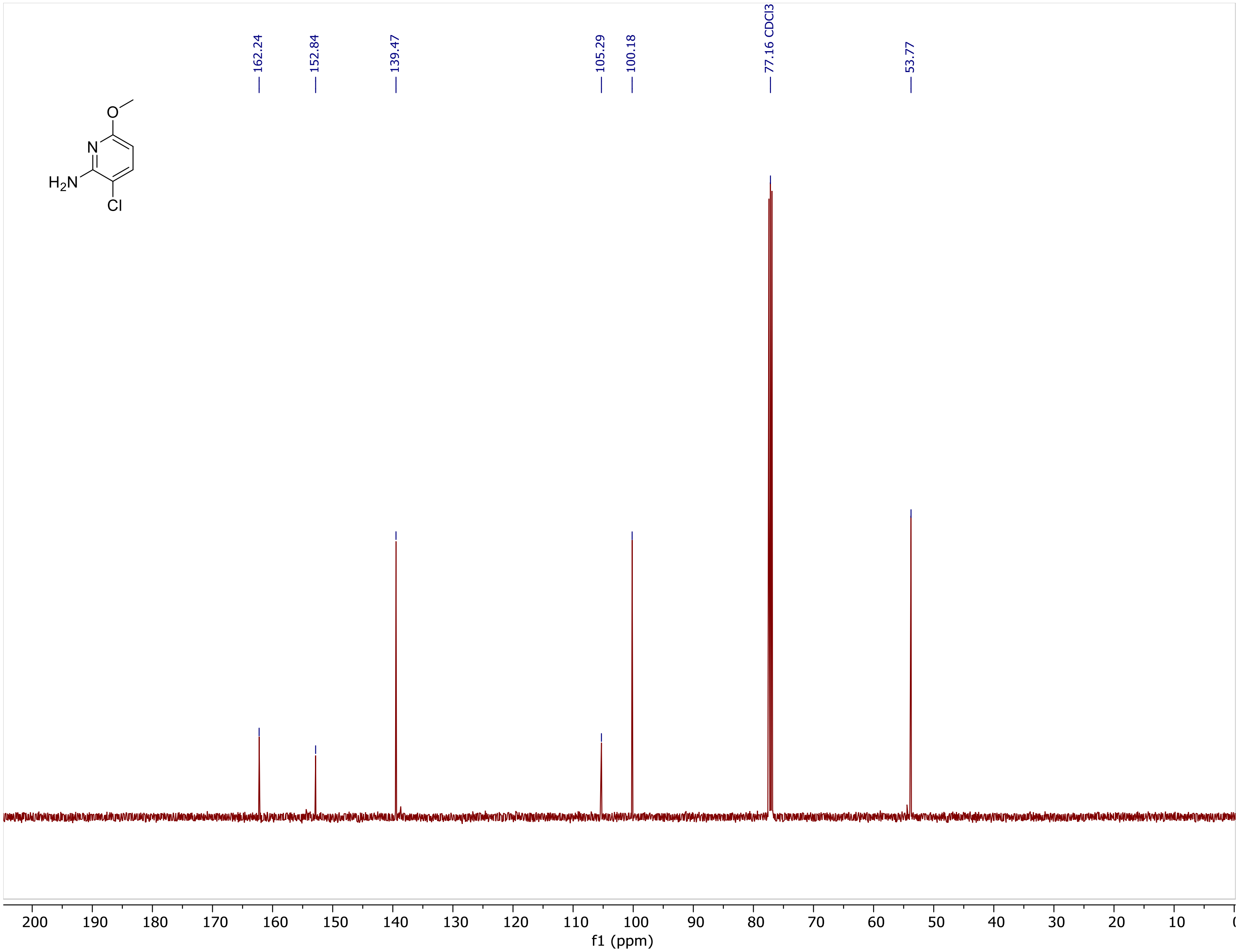

HSQC NMR (500 MHz, $CDCl_3$) spectrum of 3-chloro-6-methoxypyridin-2-amine (**27**).

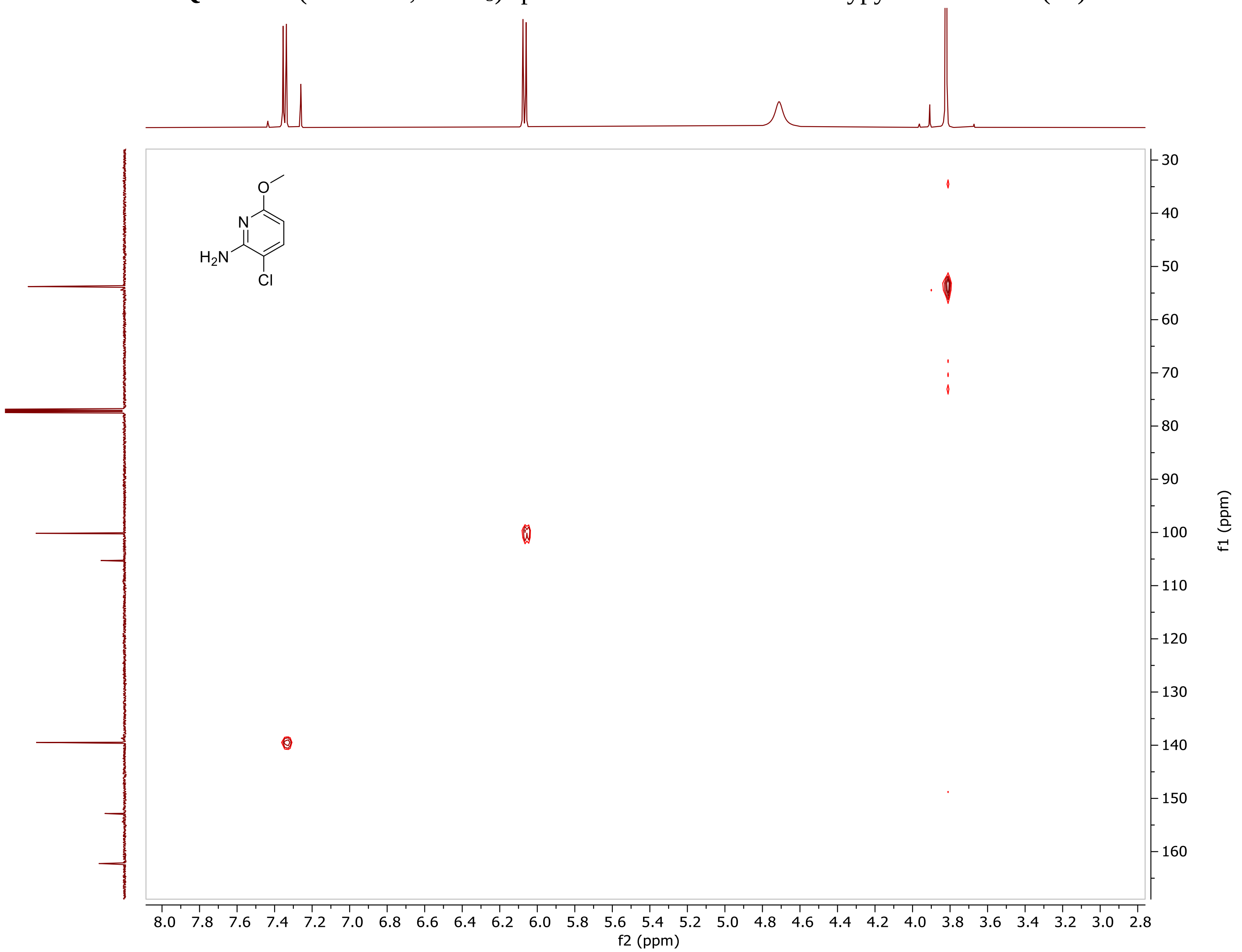

Expanded region of stacked a) **Selective 1D-NOESY** (500 MHz, $CDCl_3$) irradiated at 6.05 ppm and b)[1]**H NMR** (500 MHz, $CDCl_3$) spectrum of 3-chloro-6-methoxypyridin-2-amine (**27**). NOE interactions are shown with red arrows.

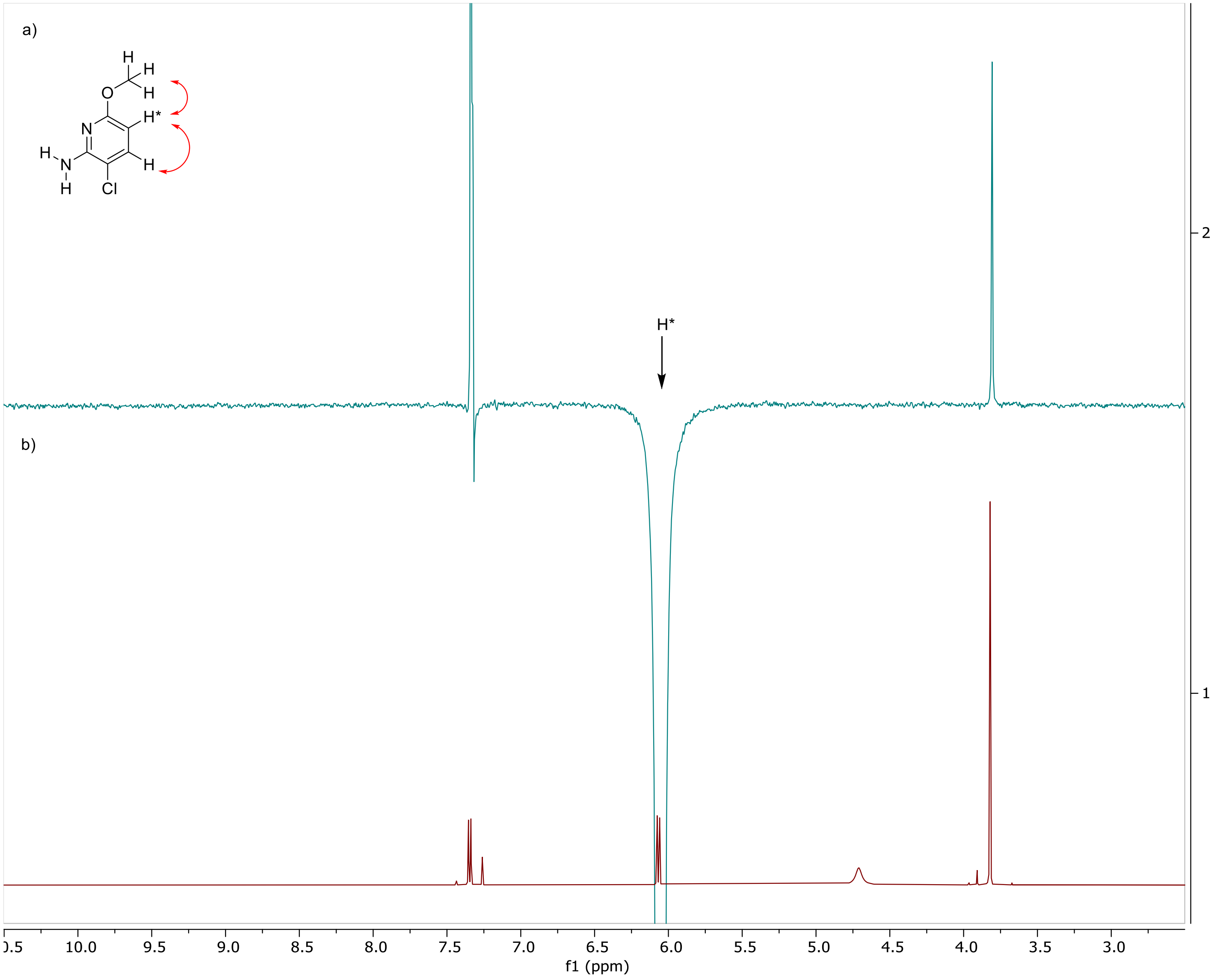

Expanded region of stacked a) **Selective 1D-NOESY** (500 MHz, $CDCl_3$) irradiated at 7.33 ppm and b)**^{1}H NMR** (500 MHz, $CDCl_3$) spectrum of 3-chloro-6-methoxypyridin-2-amine (**27**). NOE interactions are shown with red arrows.

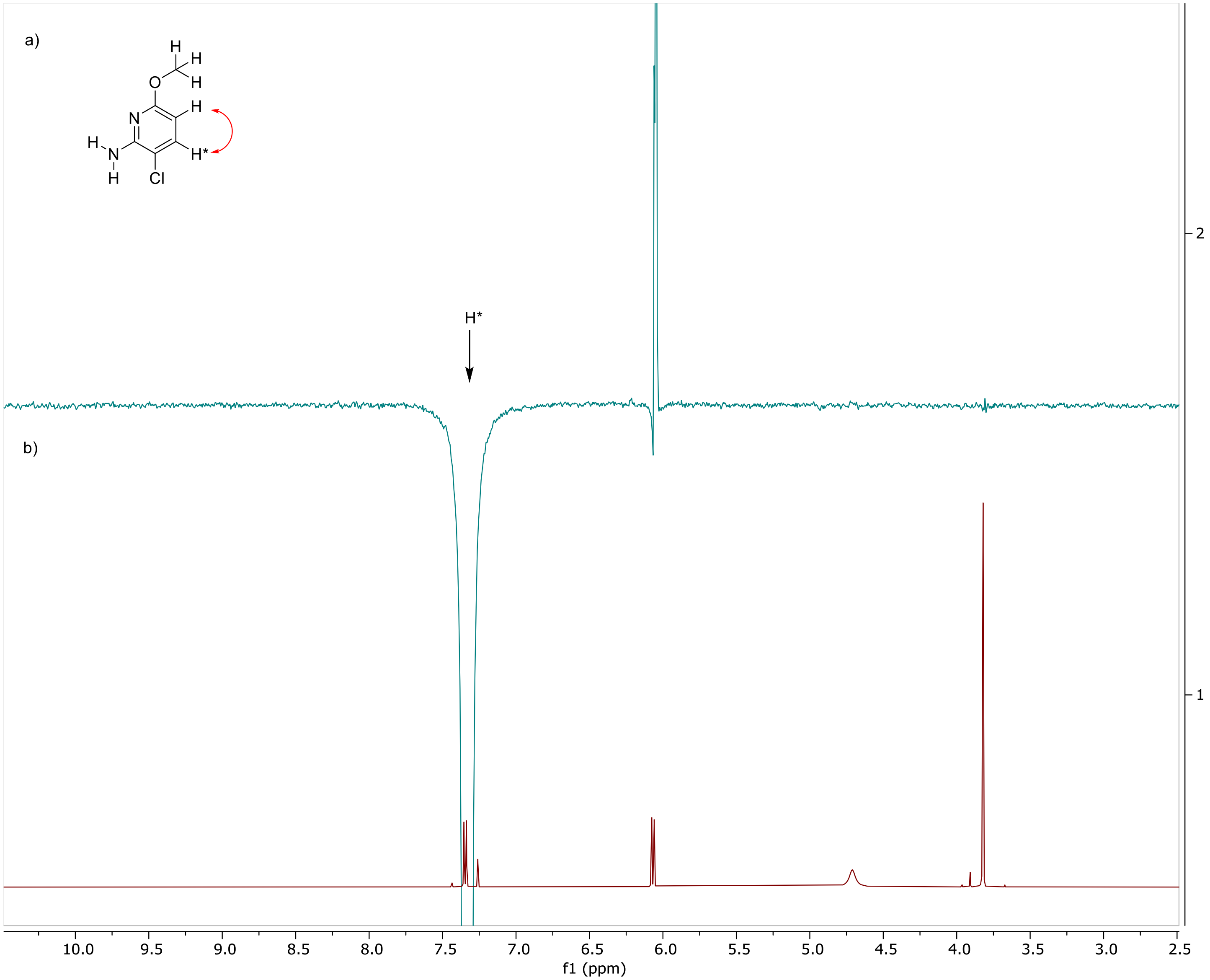

Expanded region of stacked a) **Selective 1D-NOESY** (500 MHz, $CDCl_3$) irradiated at 3.81 ppm and b)[1]**H NMR** (500 MHz, $CDCl_3$) spectrum of 3-chloro-6-methoxypyridin-2-amine (**27**). NOE interactions are shown with red arrows.

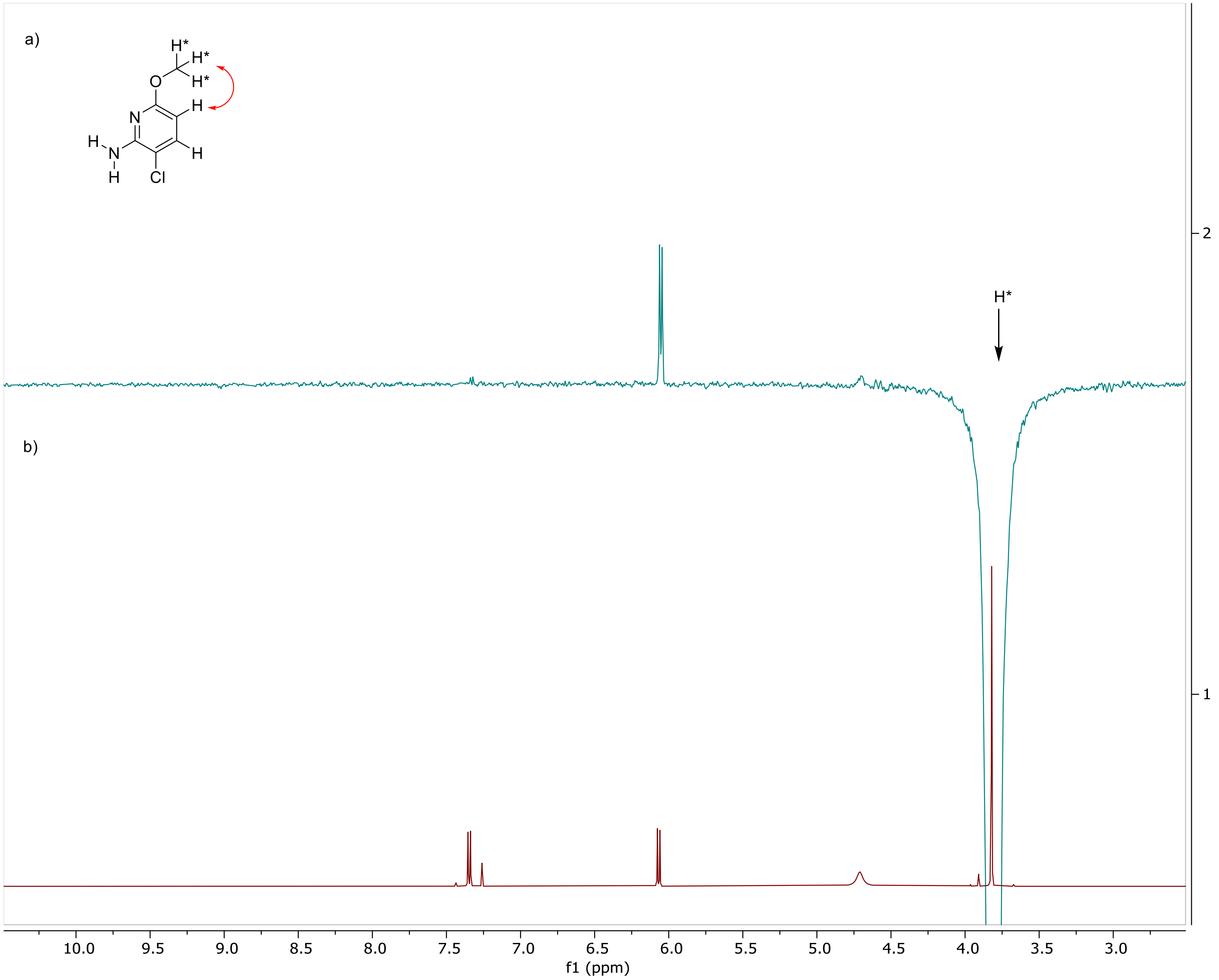

^{1}H NMR (500 MHz, $CDCl_3$) spectrum of *N*-(5-chloro-6-methoxypyridin-2-yl)benzamide (**28**).

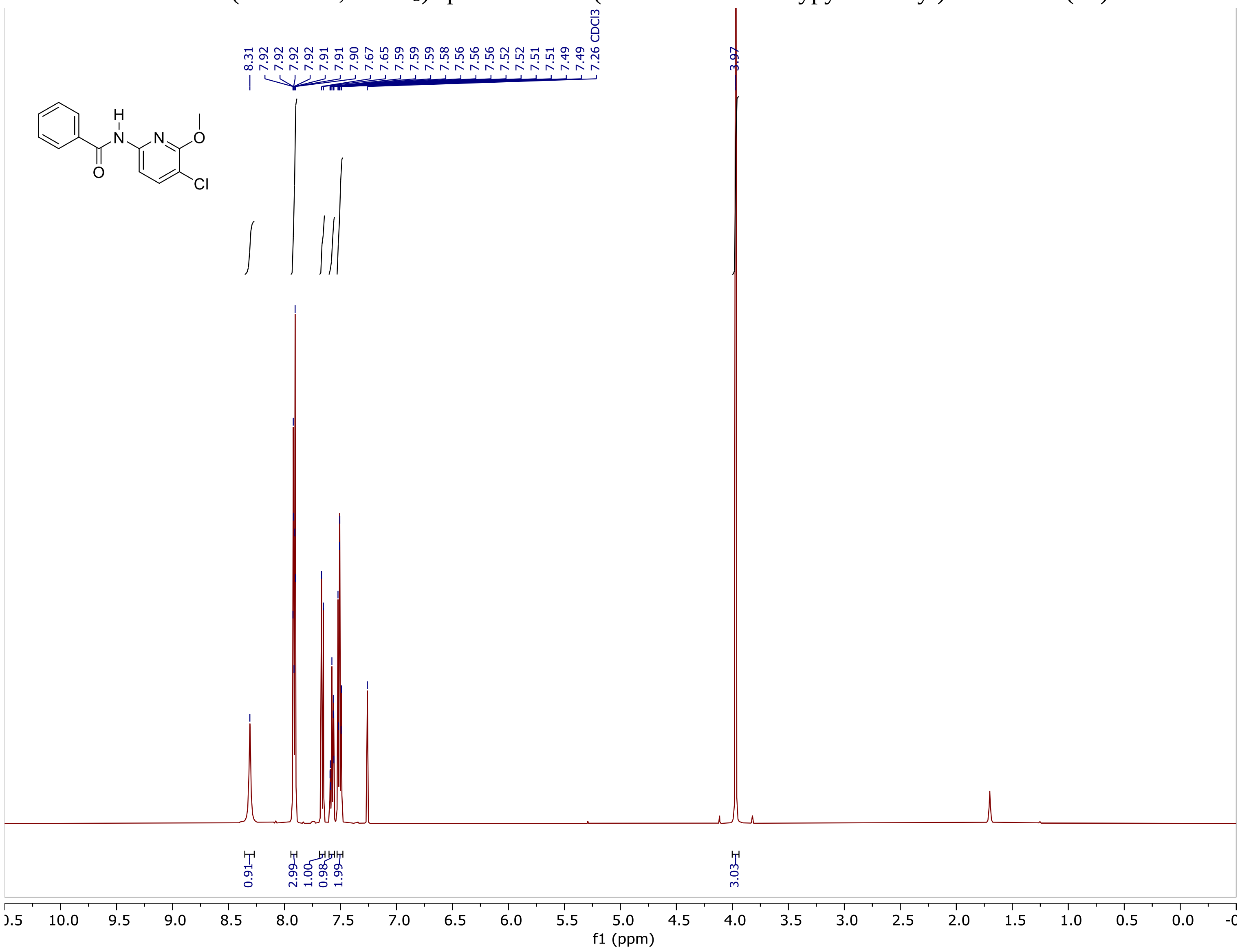

^{13}C NMR (126 MHz, $CDCl_3$) spectrum of *N*-(5-chloro-6-methoxypyridin-2-yl)benzamide (**28**).

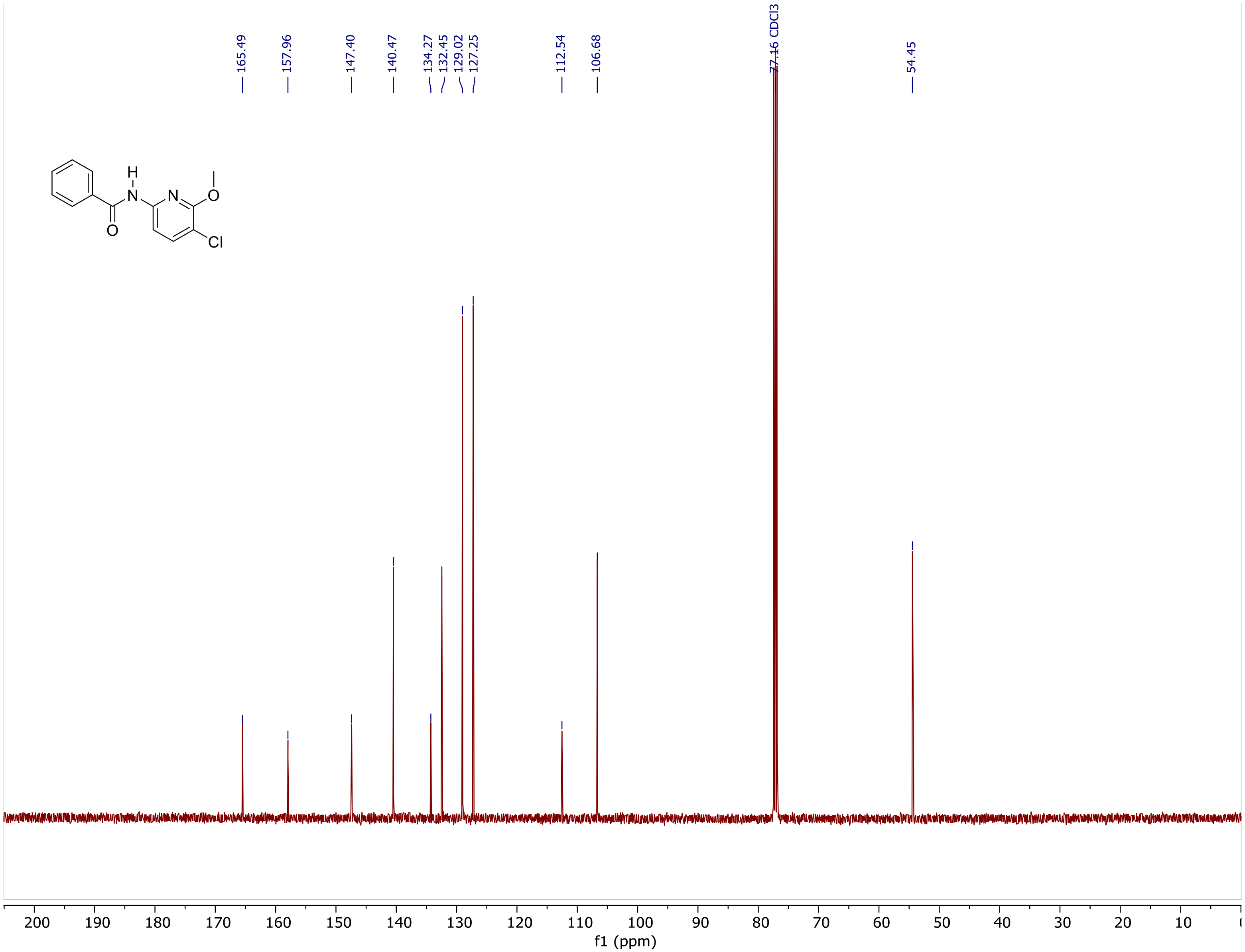

HSQC NMR (500 MHz, $CDCl_3$) spectrum of *N*-(5-chloro-6-methoxypyridin-2-yl)benzamide (**28**).

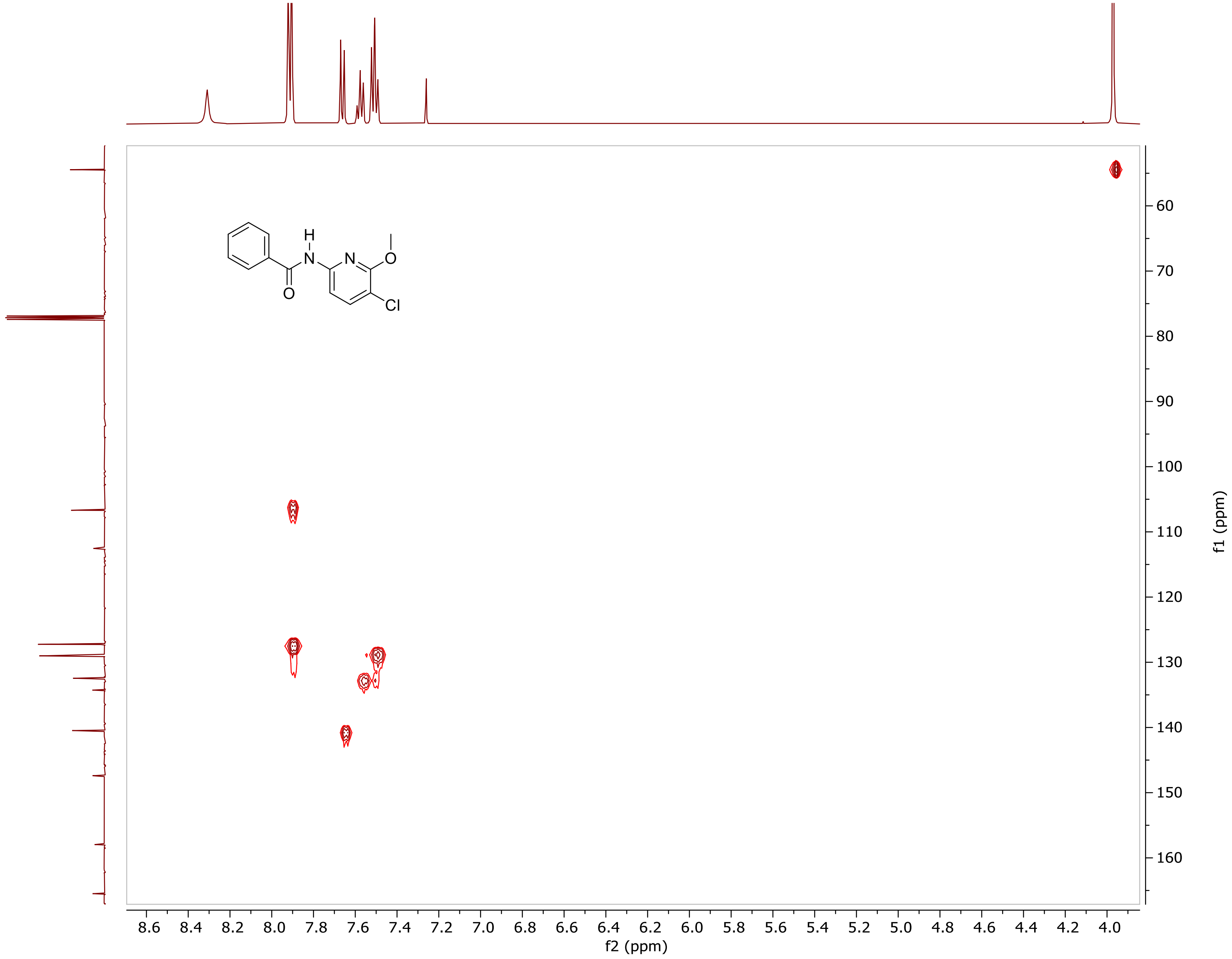

Expanded region of **HSQC NMR** (500 MHz, $CDCl_3$, 5 mM solution) spectrum of *N*-(3-chloro-2-fluorophenyl)-7-methoxy-6-(2-((4-methylpiperazin-1-yl)oxy)ethoxy)quinazolin-4-amine (**7a**).

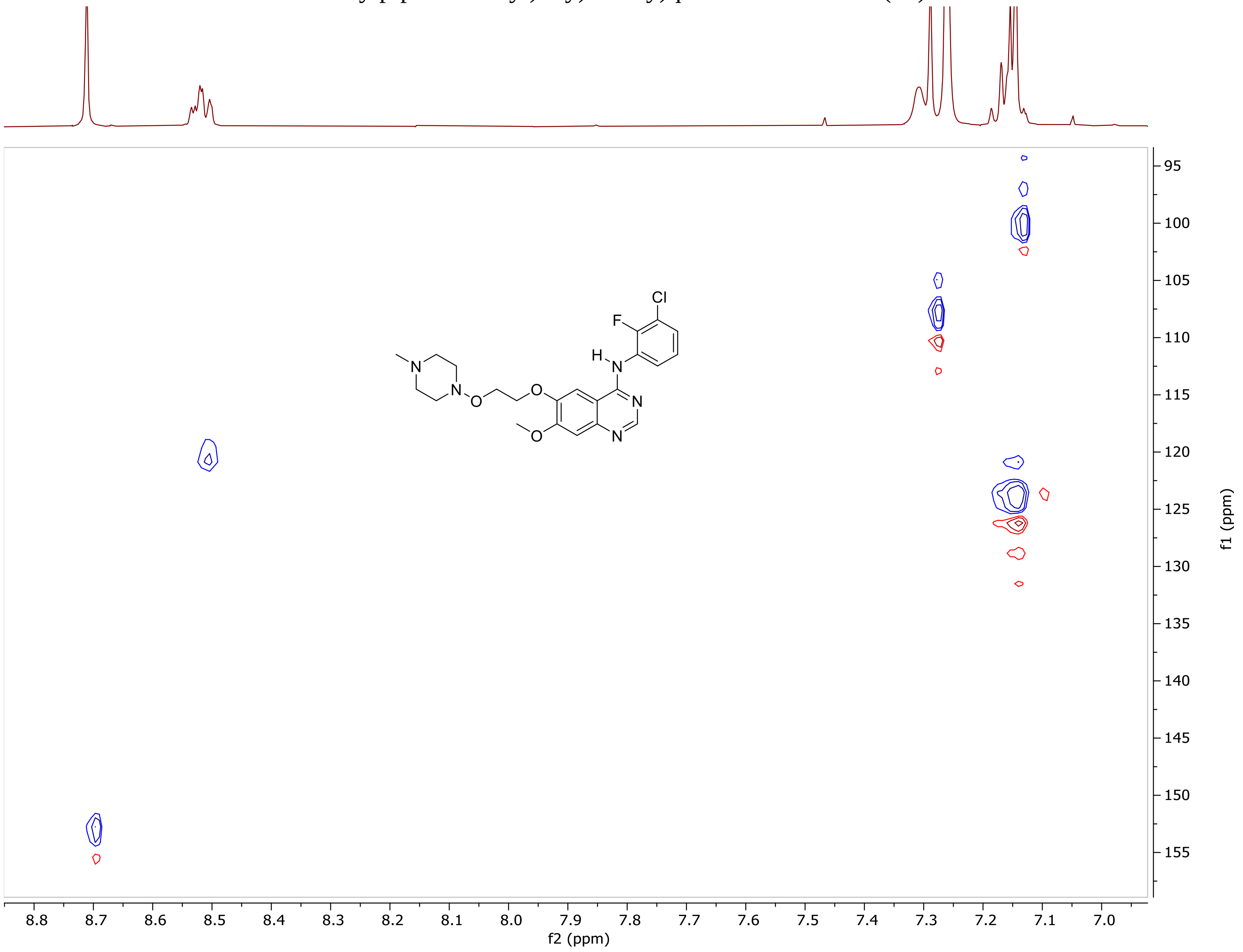

Expanded region of **^{1}H NMR** (500 MHz, $CDCl_3$, 5 mM solution) spectrum of *N*-(3-chloro-2-fluorophenyl)-7-methoxy-6-(2-((4-methylpiperazin-1-yl)oxy)ethoxy)quinazolin-4-amine (**7a**).

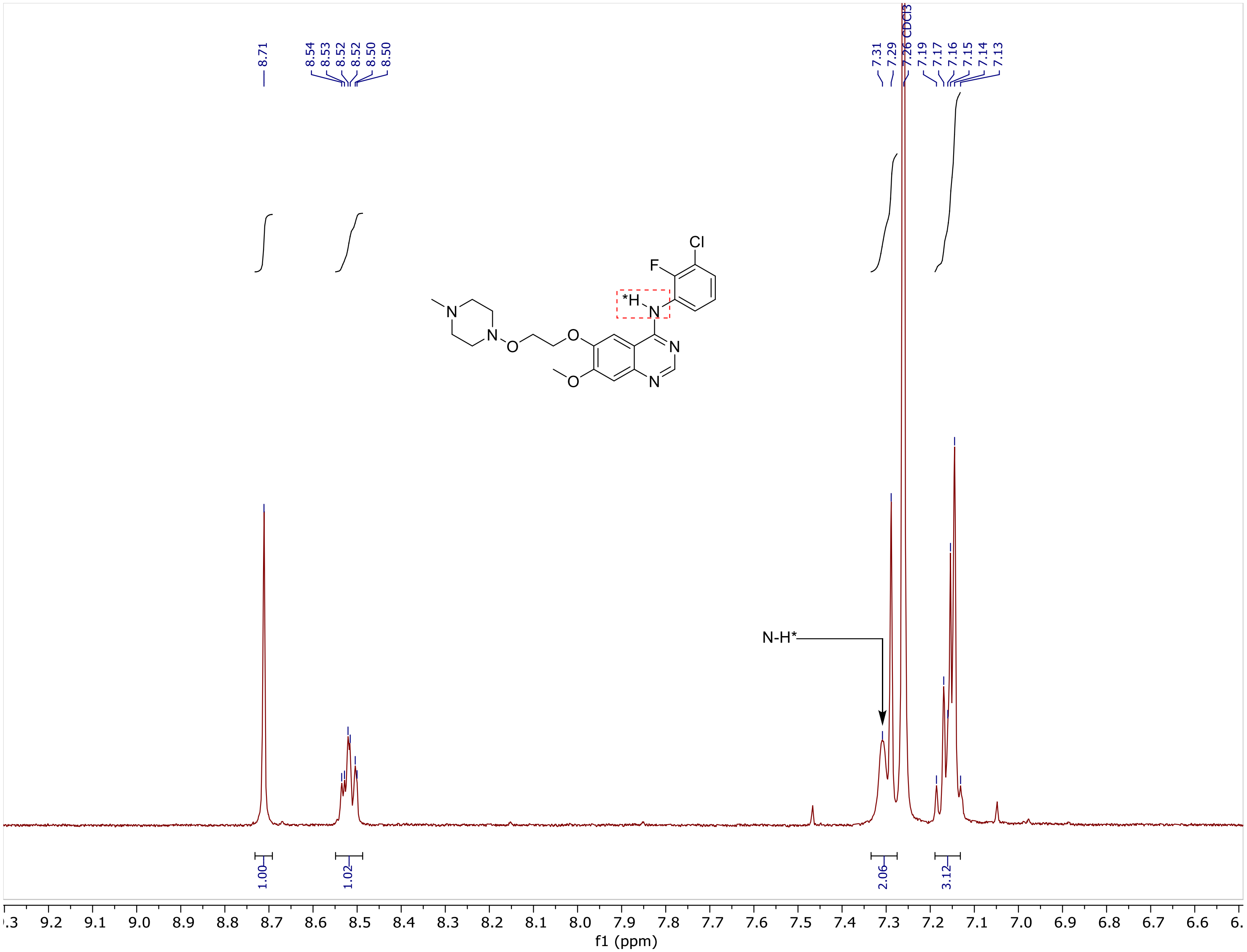

Expanded region of **HSQC NMR** (500 MHz, DMSO-D$_6$, 5 mM solution) spectrum of *N*-(3-chloro-2-fluorophenyl)-7-methoxy-6-(2-((4-methylpiperazin-1-yl)oxy)ethoxy)quinazolin-4-amine (**7a**).

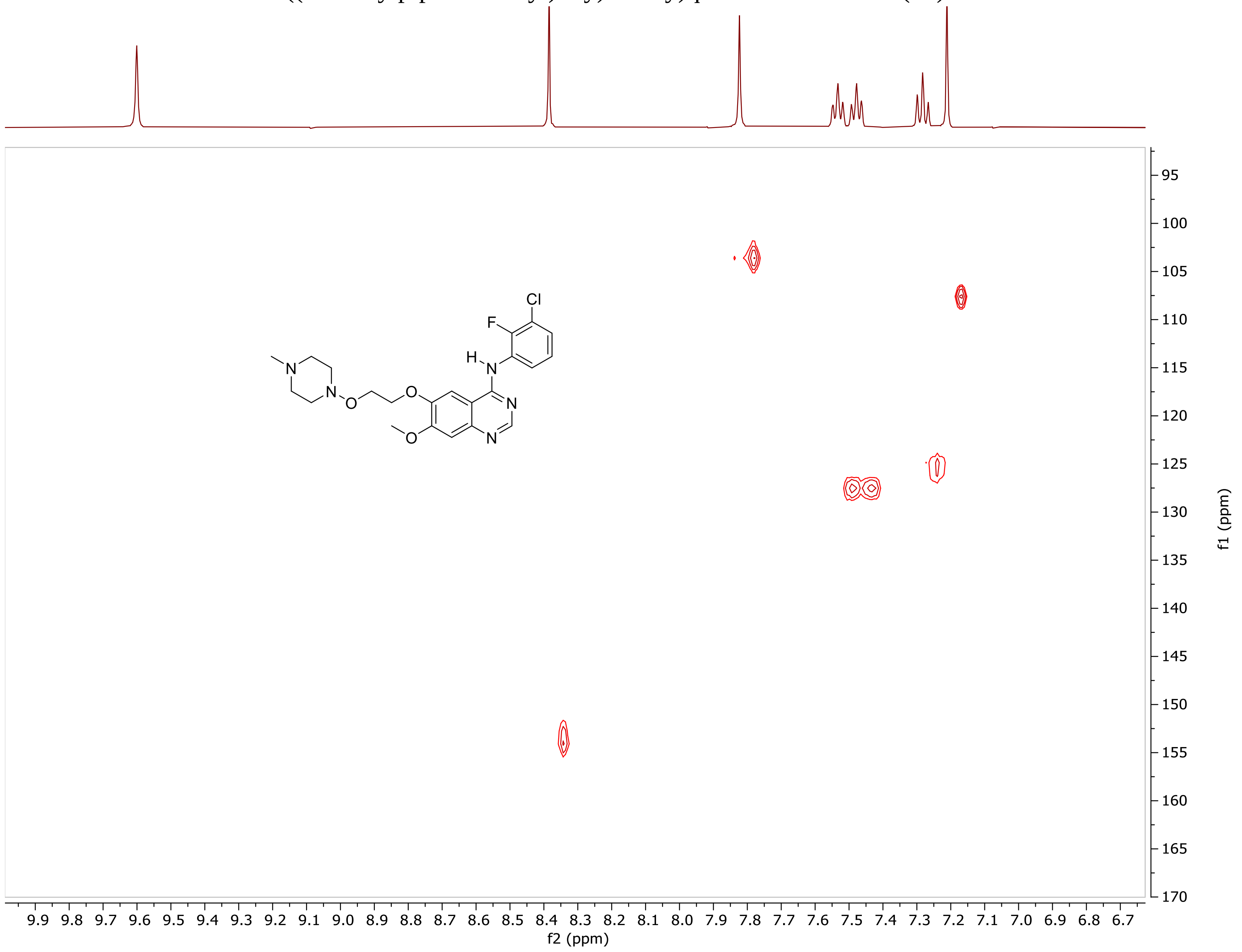

Expanded region of **^{1}H NMR** (500 MHz, DMSO-D$_6$, 5 mM solution) spectrum of *N*-(3-chloro-2-fluorophenyl)-7-methoxy-6-(2-((4-methylpiperazin-1-yl)oxy)ethoxy)quinazolin-4-amine (**7a**).

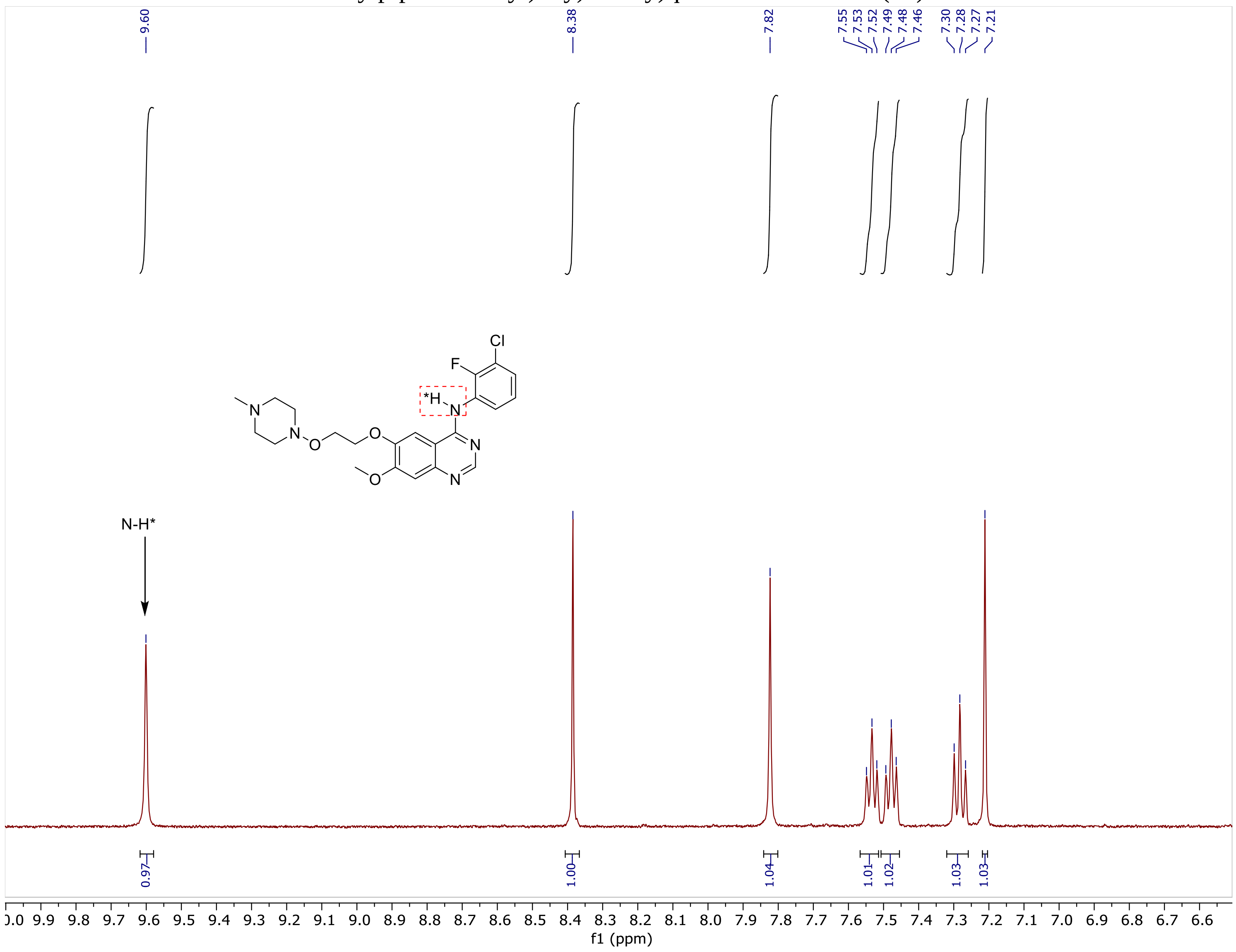

^{1}H NMR (500 MHz, $CDCl_3$) spectrum of 4-((4-chloro-2-fluoro-5-methoxyphenyl)amino)-6-methoxy-7-(2-((4-methylpiperazin-1-yl)oxy)ethoxy)quinoline-3-carbonitrile (**8a**).

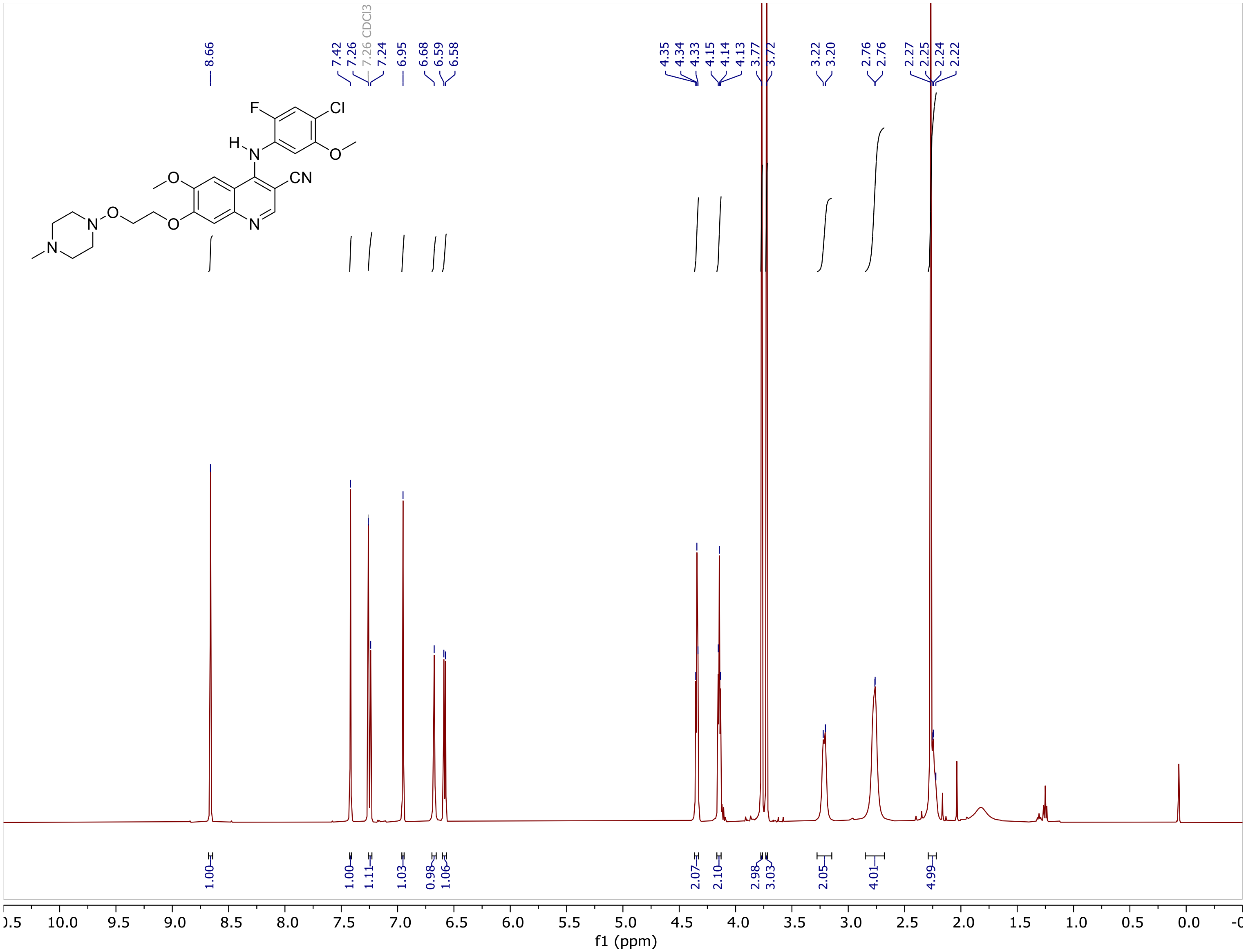

^{13}C NMR (126 MHz, $CDCl_3$) spectrum of 4-((4-chloro-2-fluoro-5-methoxyphenyl)amino)-6-methoxy-7-(2-((4-methylpiperazin-1-yl)oxy)ethoxy)quinoline-3-carbonitrile (**8a**).

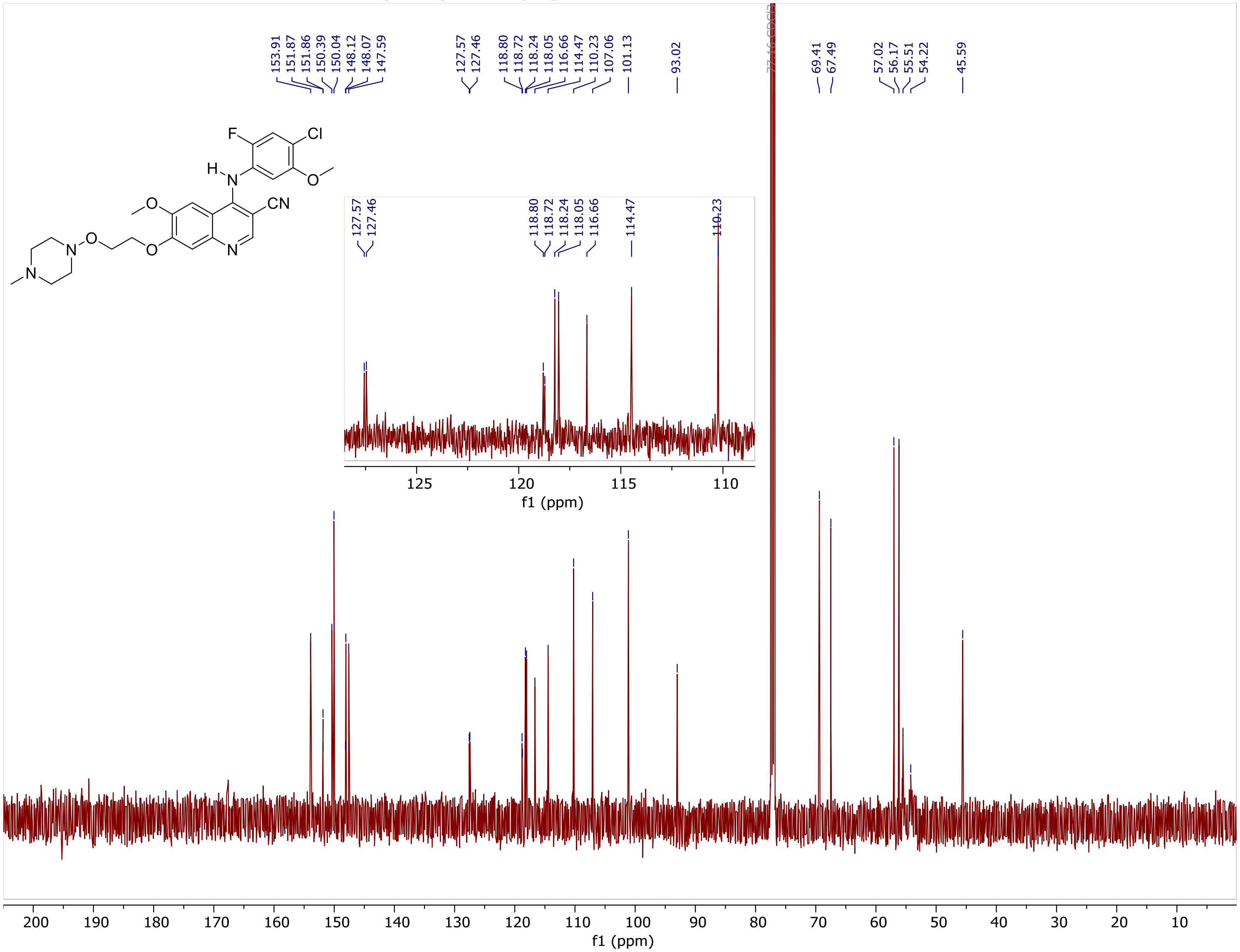

^{13}C {^{19}F} NMR (126 MHz, $CDCl_3$) spectrum of 4-((4-chloro-2-fluoro-5-methoxyphenyl)amino)-6-methoxy-7-(2-((4-methylpiperazin-1-yl)oxy)ethoxy)quinoline-3-carbonitrile (**8a**).

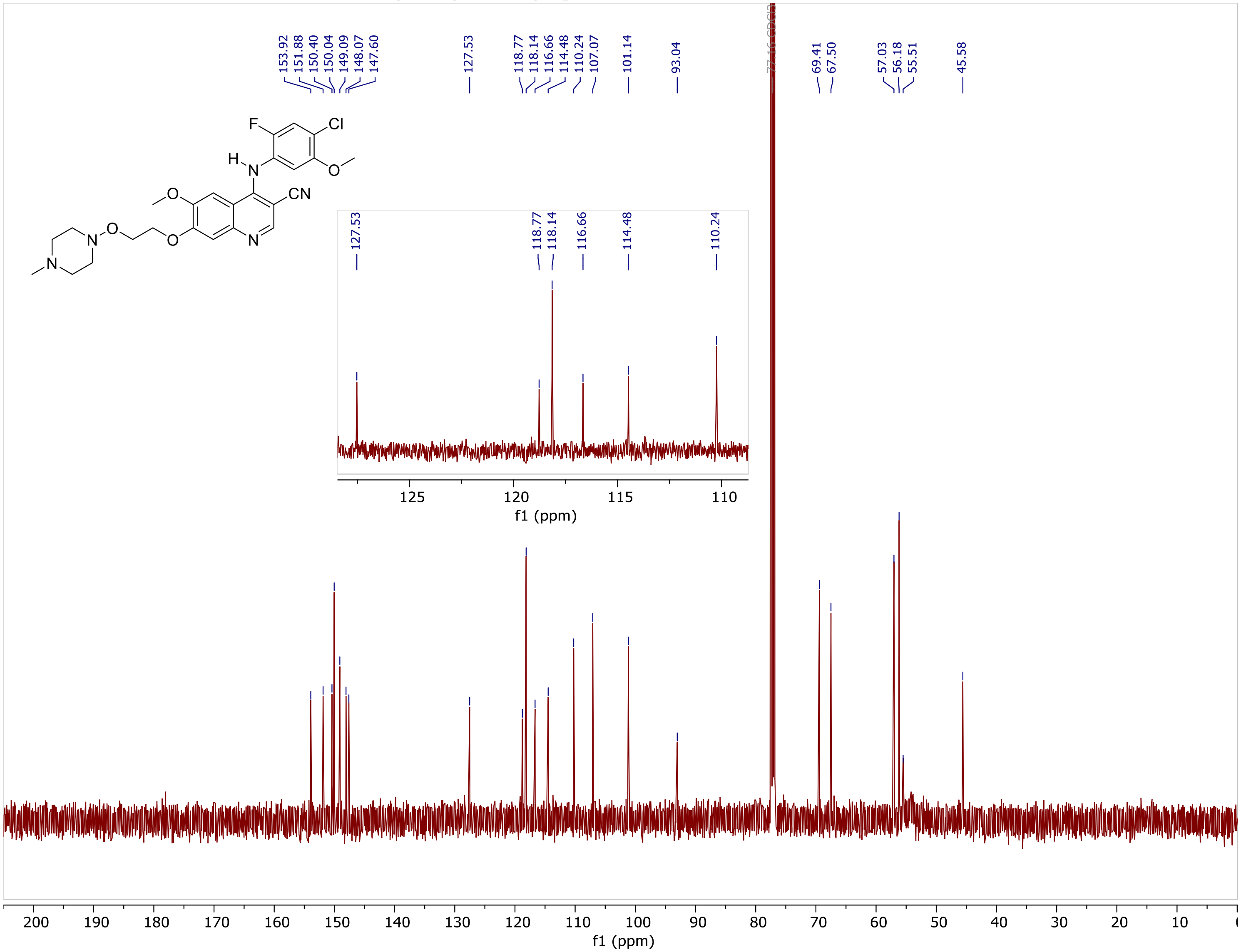

Expanded region of stacked a) **^{13}C {^{19}F} NMR** (126 MHz, $CDCl_3$) and b) **^{13}C NMR** (126 MHz, $CDCl_3$) spectrum of 4-((4-chloro-2-fluoro-5-methoxyphenyl)amino)-6-methoxy-7-(2-((4-methylpiperazin-1-yl)oxy)ethoxy)quinoline-3-carbonitrile (**8a**).

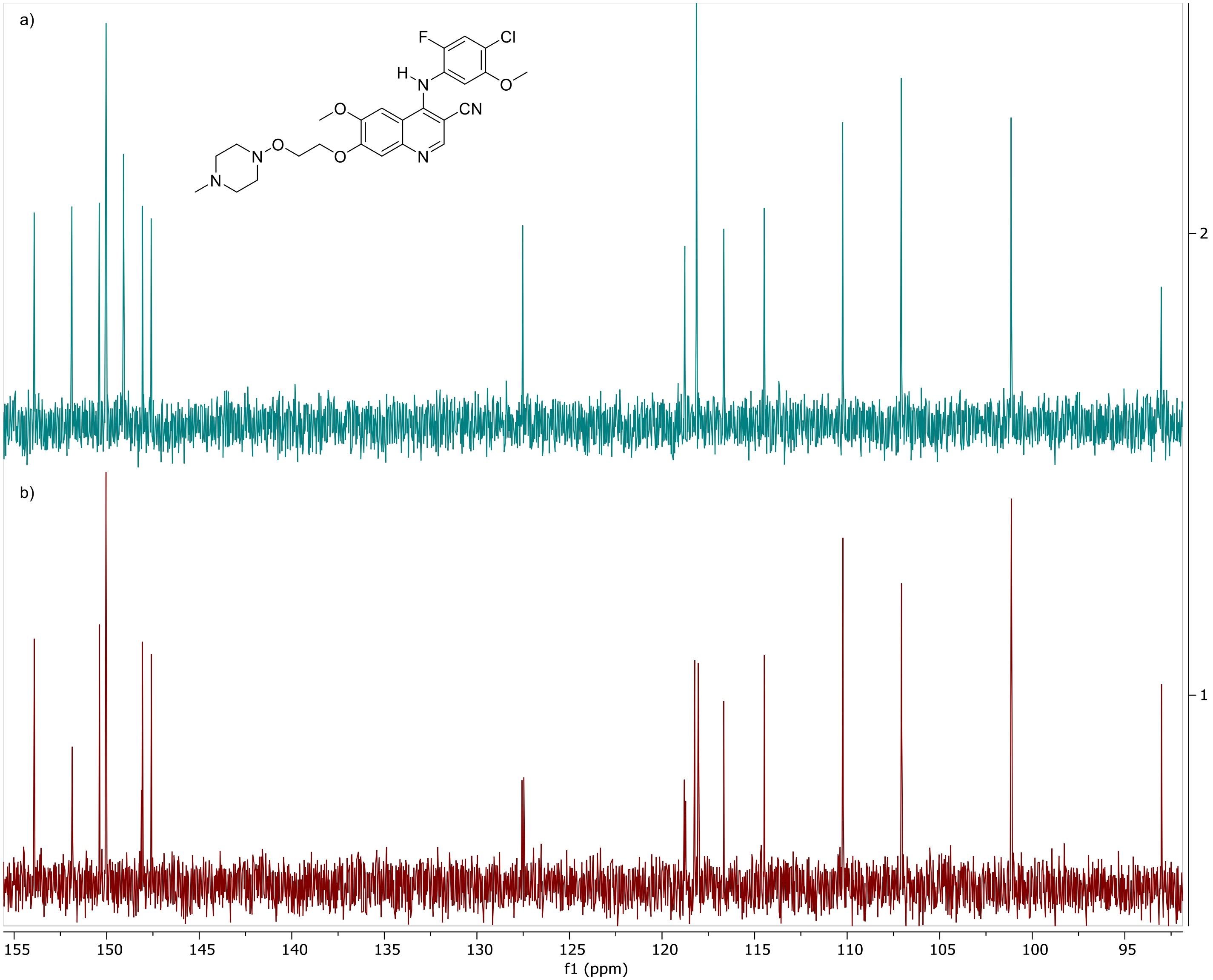

^{19}F {^{1}H} NMR (470 MHz, $CDCl_3$) spectrum of 4-((4-chloro-2-fluoro-5-methoxyphenyl)amino)-6-methoxy-7-(2-((4-methylpiperazin-1-yl)oxy)ethoxy)quinoline-3-carbonitrile (**8a**).

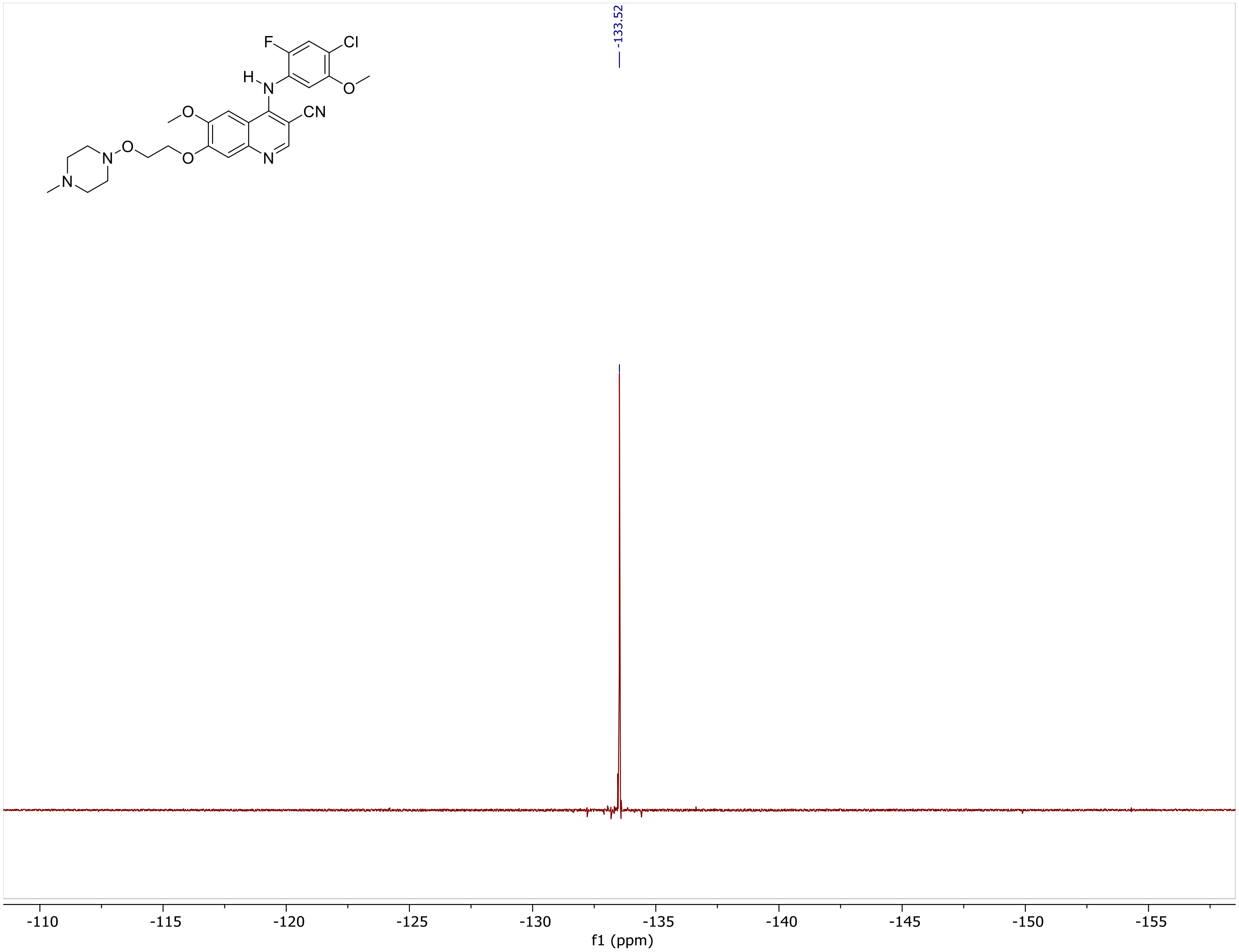

HSQC NMR (500 MHz, $CDCl_3$) spectrum of 4-((4-chloro-2-fluoro-5-methoxyphenyl)amino)-6-methoxy-7-(2-((4-methylpiperazin-1-yl)oxy)ethoxy)quinoline-3-carbonitrile (**8a**).

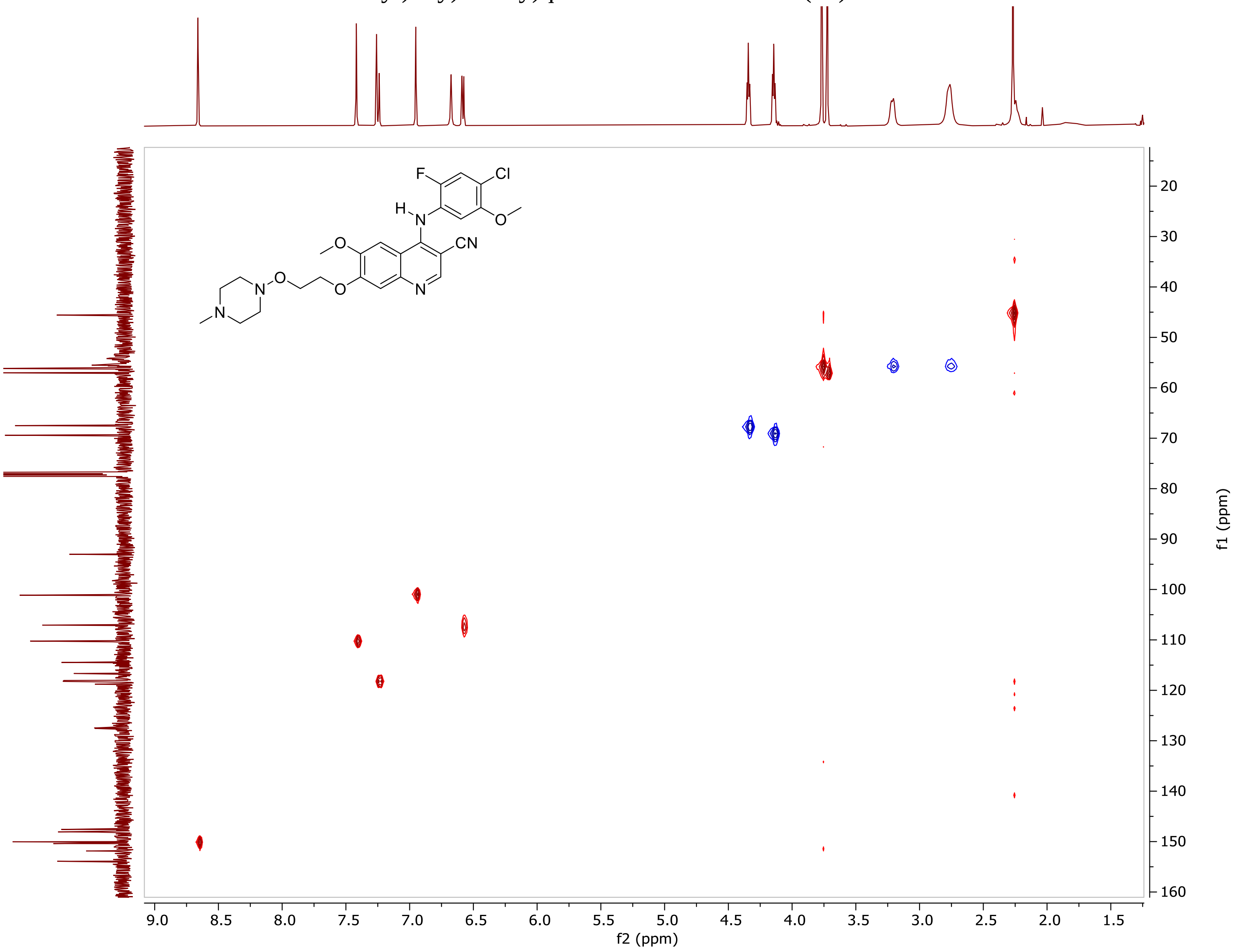

DQF-COSY NMR (500 MHz, $CDCl_3$) spectrum of 4-((4-chloro-2-fluoro-5-methoxyphenyl)amino)-6-methoxy-7-(2-((4-methylpiperazin-1-yl)oxy)ethoxy)quinoline-3-carbonitrile (**8a**).

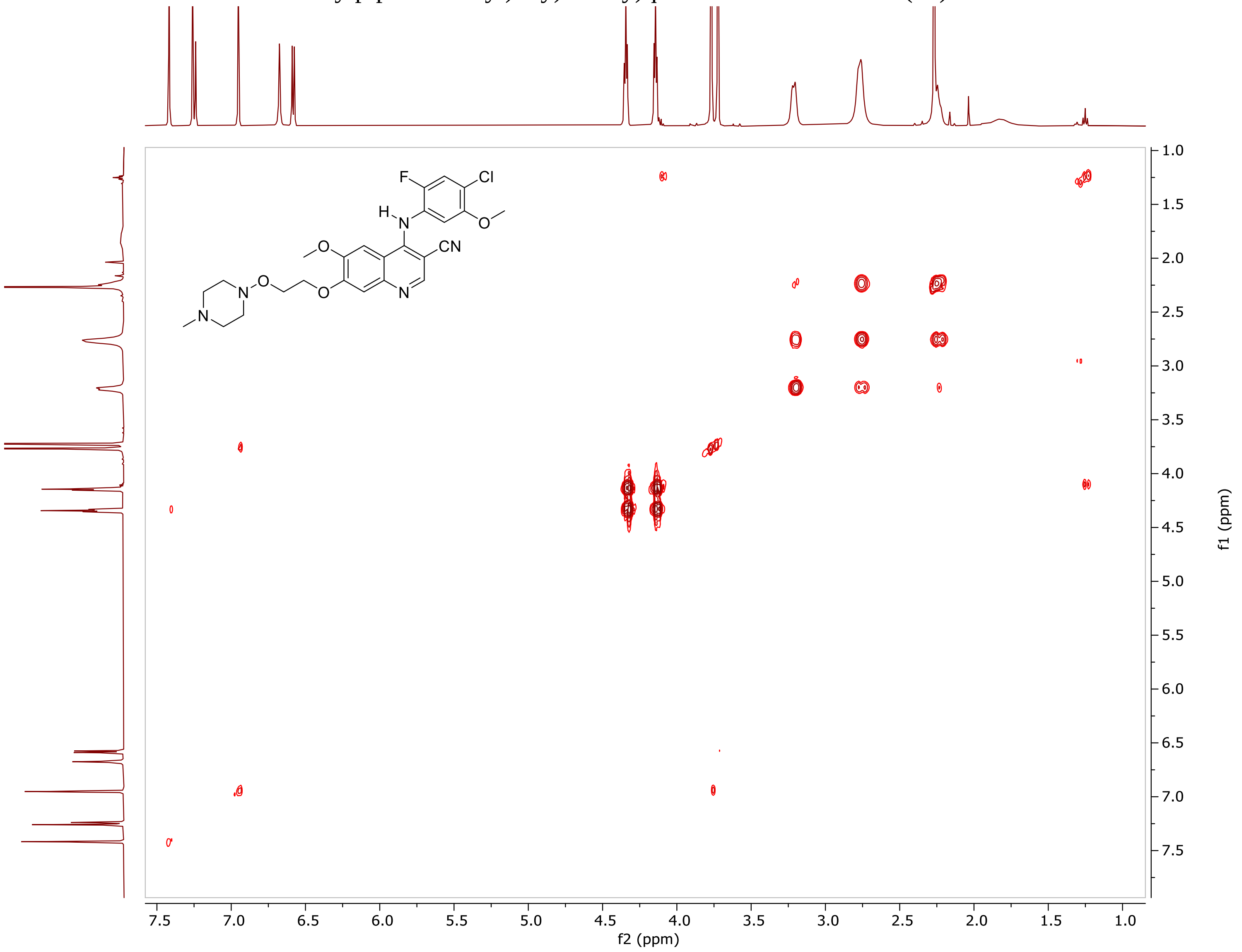

Expanded region of stacked ^{13}C **NMR** (126 MHz, $CDCl_3$) spectrum of 4-((4-chloro-2-fluoro-5-methoxyphenyl)amino)-6-methoxy-7-(2-((4-methylpiperazin-1-yl)oxy)ethoxy)quinoline-3-carbonitrile (**8a**) at a) 328K and b) 298 K.

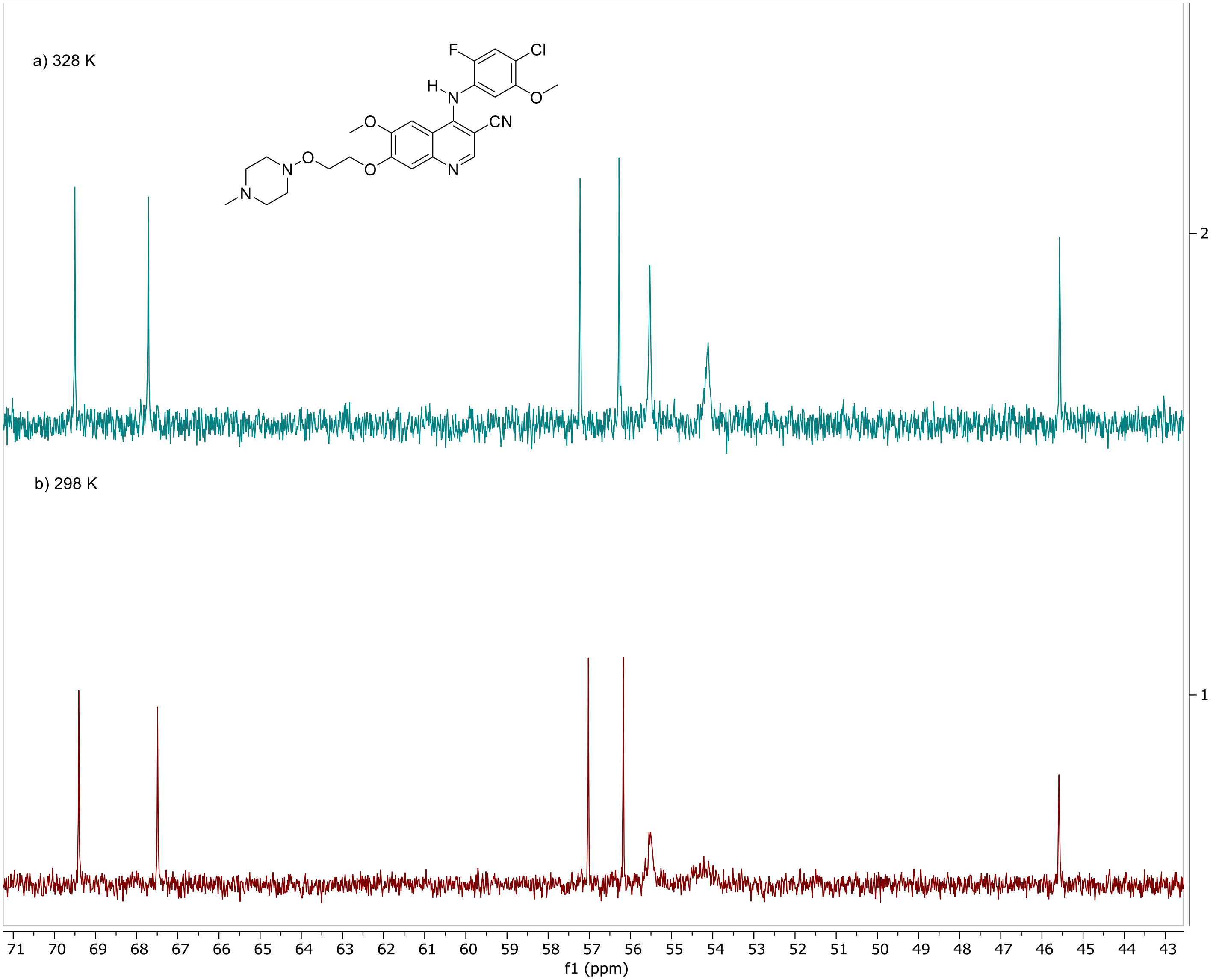

Expanded region of **HSQC NMR** (500 MHz, $CDCl_3$, 5 mM solution) spectrum of 4-((4-chloro-2-fluoro-5-methoxyphenyl)amino)-6-methoxy-7-(2-((4-methylpiperazin-1-yl)oxy)ethoxy)quinoline-3-carbonitrile (**8a**).

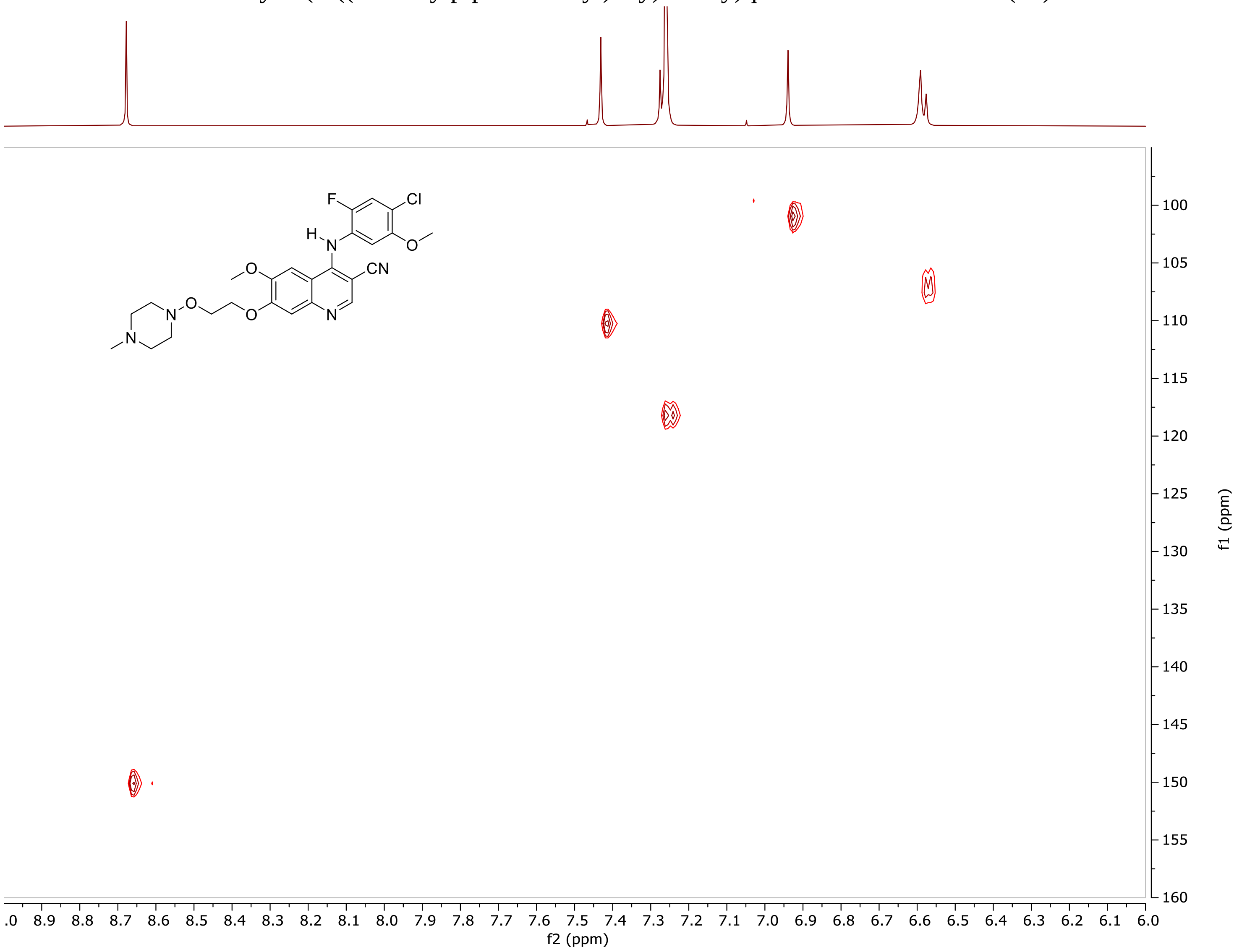

Expanded region of **¹H NMR** (500 MHz, $CDCl_3$, 5 mM solution) spectrum of 4-((4-chloro-2-fluoro-5-methoxyphenyl)amino)-6-methoxy-7-(2-((4-methylpiperazin-1-yl)oxy)ethoxy)quinoline-3-carbonitrile (**8a**).

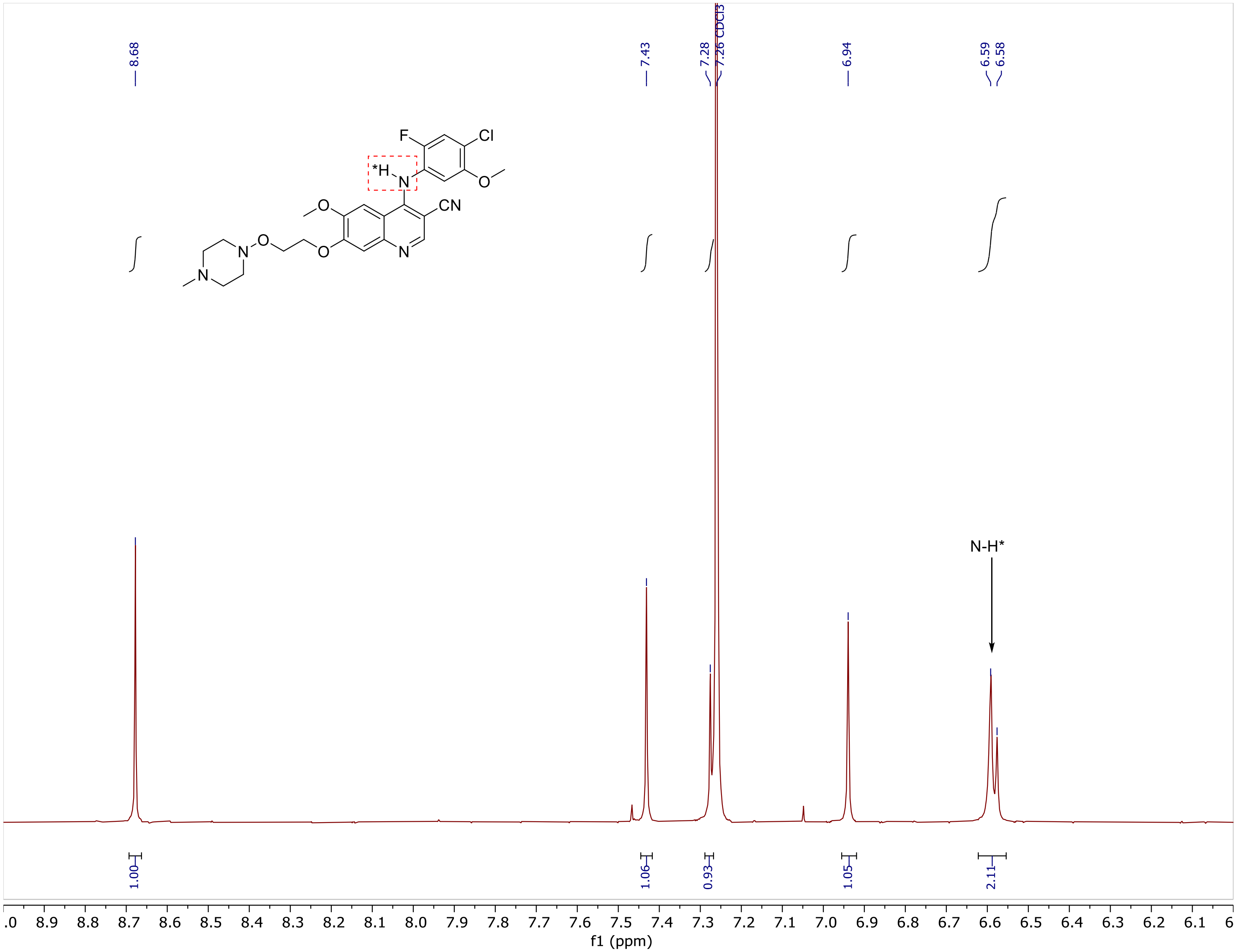

Expanded region of **¹H NMR** (500 MHz, DMSO-D$_6$, 5 mM solution) spectrum of 4-((4-chloro-2-fluoro-5-methoxyphenyl)amino)-6-methoxy-7-(2-((4-methylpiperazin-1-yl)oxy)ethoxy)quinoline-3-carbonitrile (**8a**).

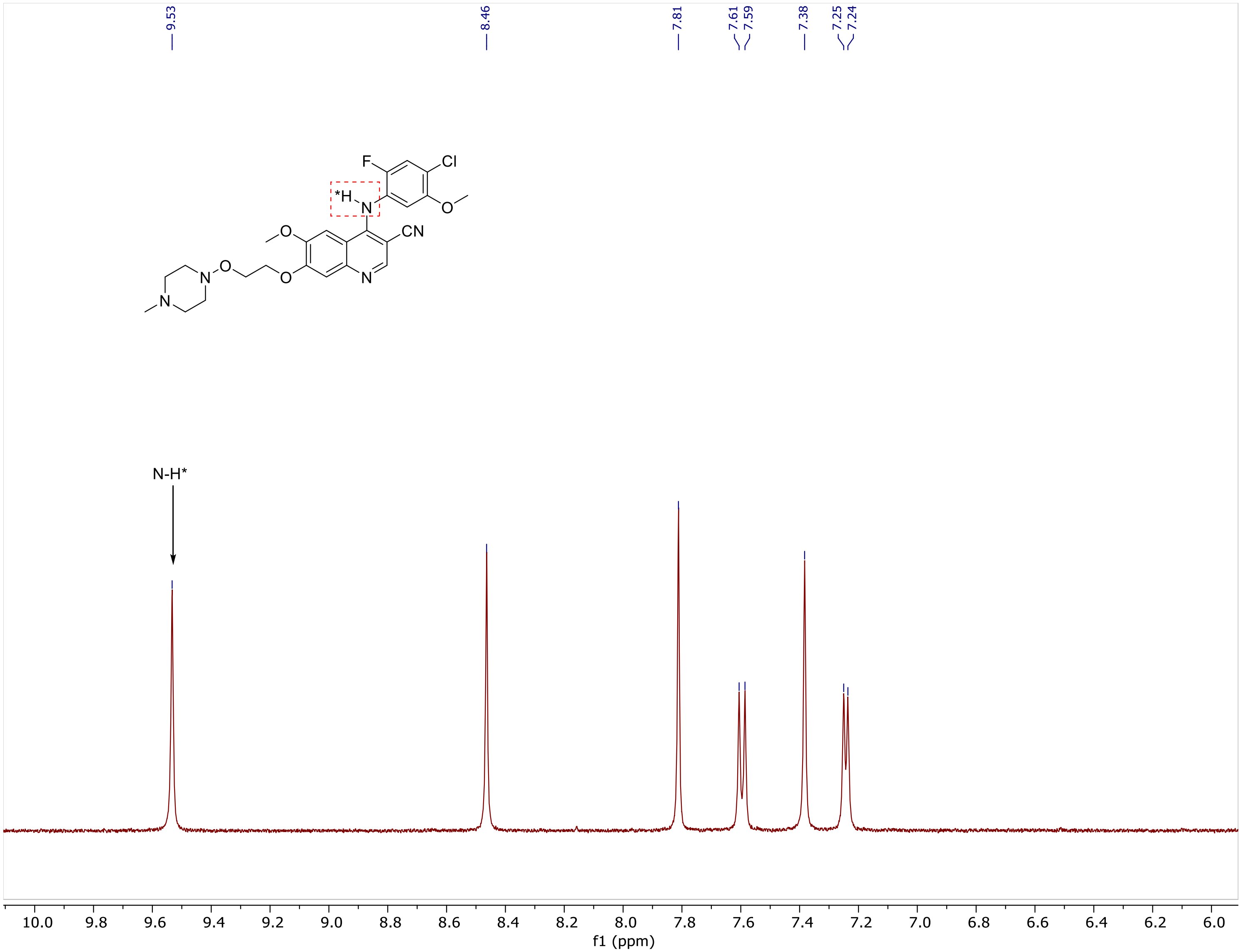

Expanded region of **HSQC NMR** (500 MHz, DMSO-D$_6$, 5 mM solution) spectrum of 4-((4-chloro-2-fluoro-5-methoxyphenyl)amino)-6-methoxy-7-(2-((4-methylpiperazin-1-yl)oxy)ethoxy)quinoline-3-carbonitrile (**8a**).

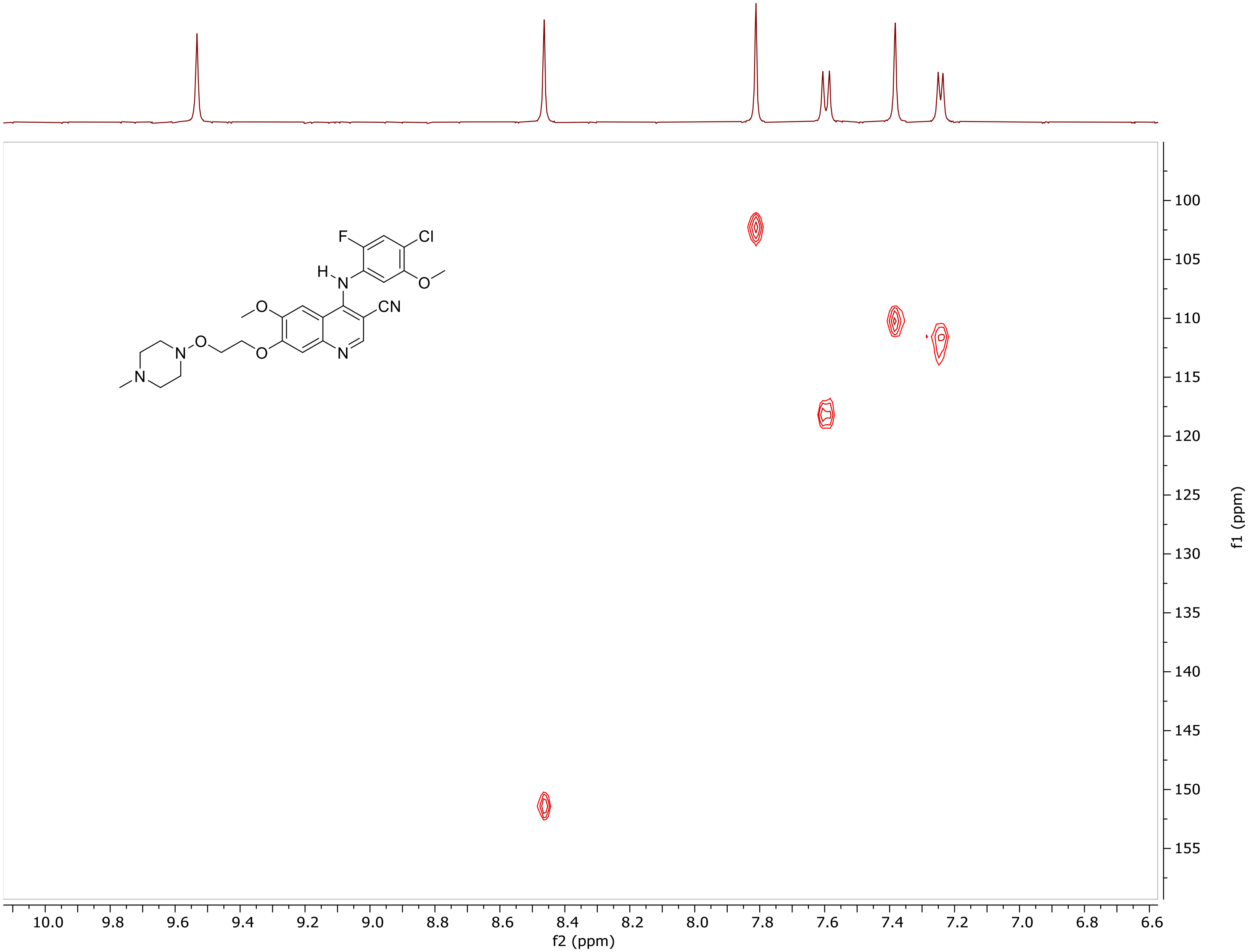

^{1}H NMR (500 MHz, $CDCl_3$) spectrum of 4-((3-chloro-2-fluorophenyl)amino)-6-methoxy-7-(2-((4-methylpiperazin-1-yl)oxy)ethoxy)quinoline-3-carbonitrile (**11a**).

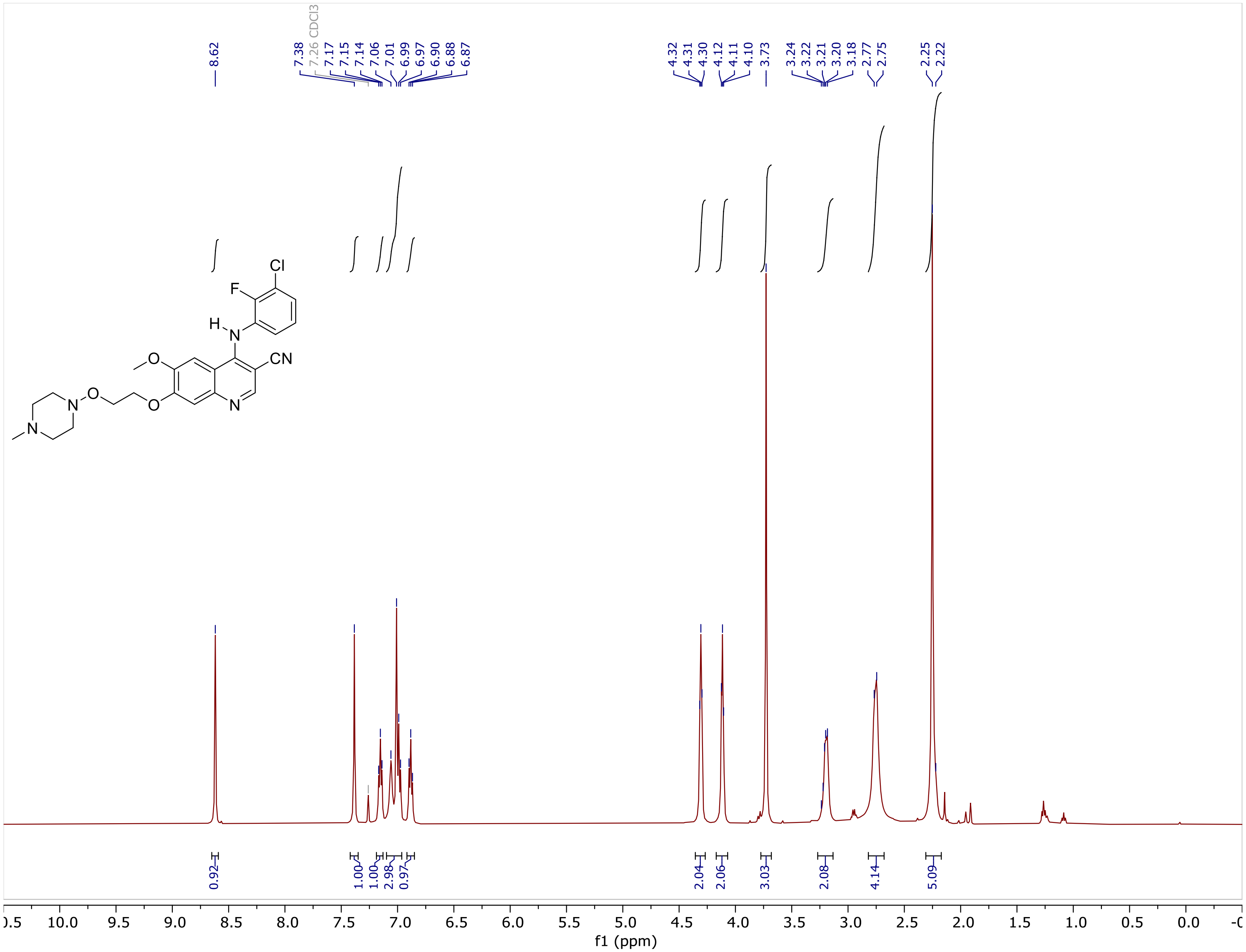

^{13}**C NMR** (126 MHz, $CDCl_3$) spectrum of 4-((3-chloro-2-fluorophenyl)amino)-6-methoxy-7-(2-((4-methylpiperazin-1-yl)oxy)ethoxy)quinoline-3-carbonitrile (**11a**).

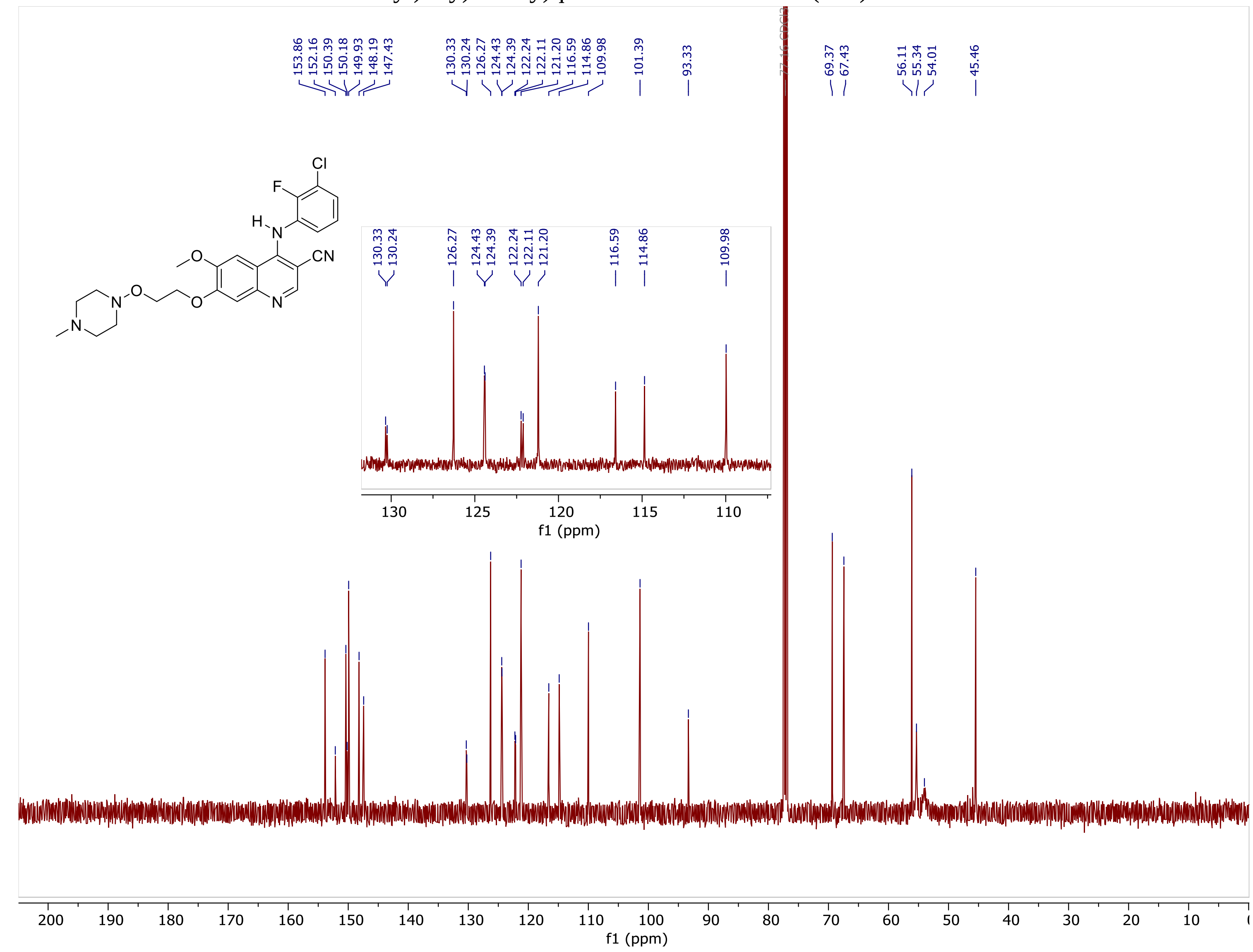

^{13}C {^{19}F} NMR (126 MHz, $CDCl_3$) spectrum of 4-((3-chloro-2-fluorophenyl)amino)-6-methoxy-7-(2-((4-methylpiperazin-1-yl)oxy)ethoxy)quinoline-3-carbonitrile (**11a**).

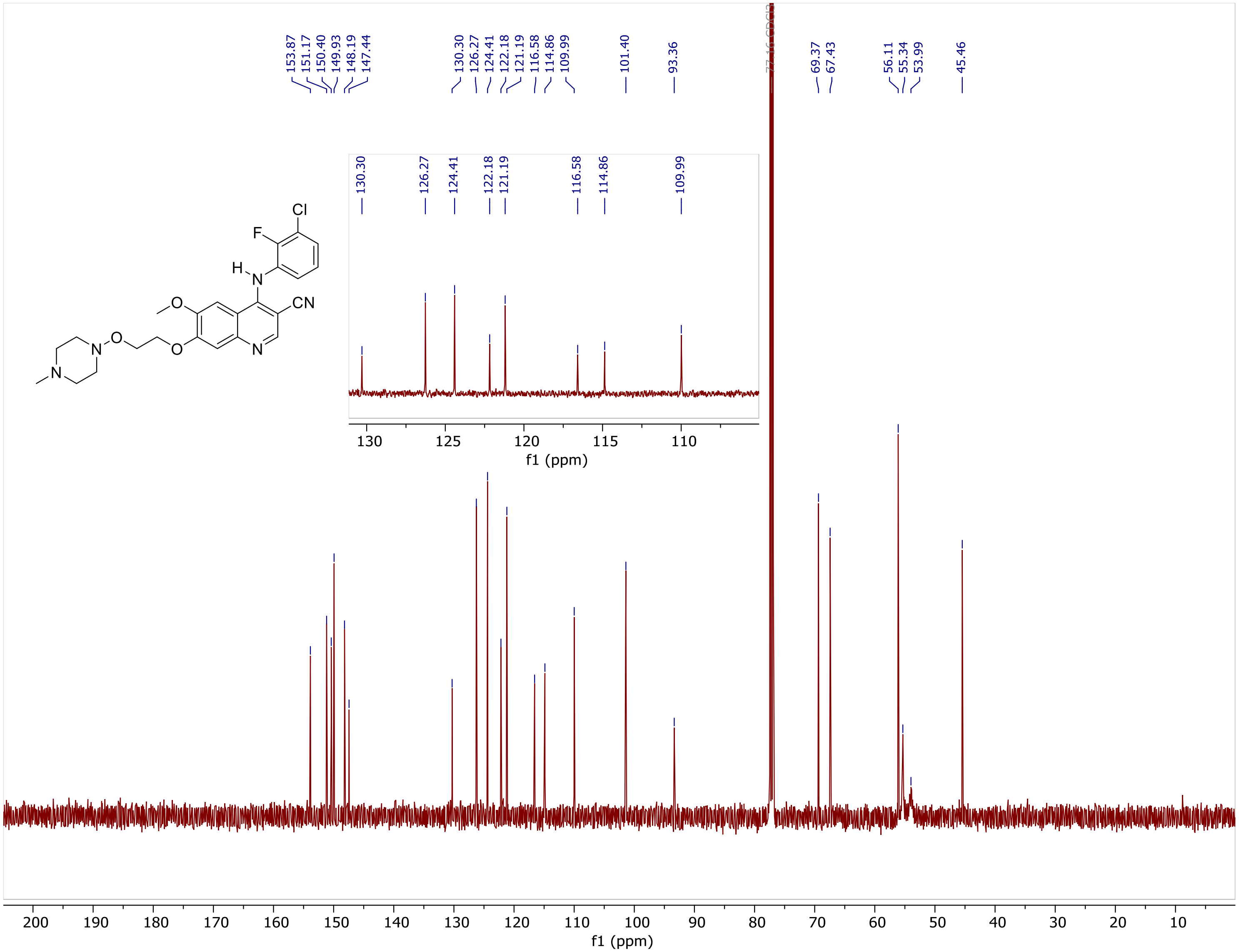

Expanded region of stacked a) ^{13}C {^{19}F} NMR (126 MHz, $CDCl_3$) and b) ^{13}C NMR (126 MHz, $CDCl_3$) spectrum of 4-((3-chloro-2-fluorophenyl)amino)-6-methoxy-7-(2-((4-methylpiperazin-1-yl)oxy)ethoxy)quinoline-3-carbonitrile (**11a**).

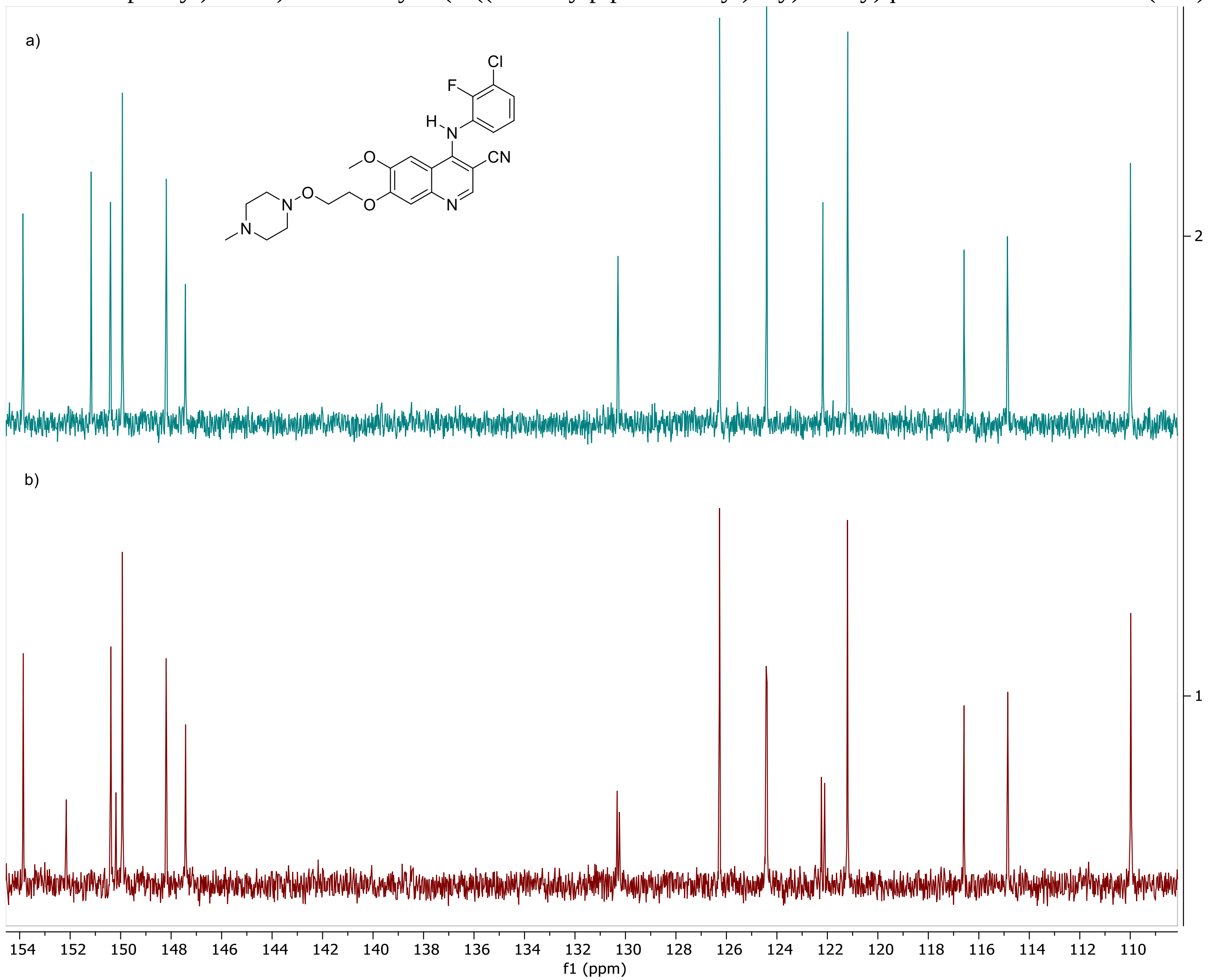

^{19}F {^{1}H} NMR (470 MHz, $CDCl_3$) spectrum of 4-((3-chloro-2-fluorophenyl)amino)-6-methoxy-7-(2-((4-methylpiperazin-1-yl)oxy)ethoxy)quinoline-3-carbonitrile (**11a**).

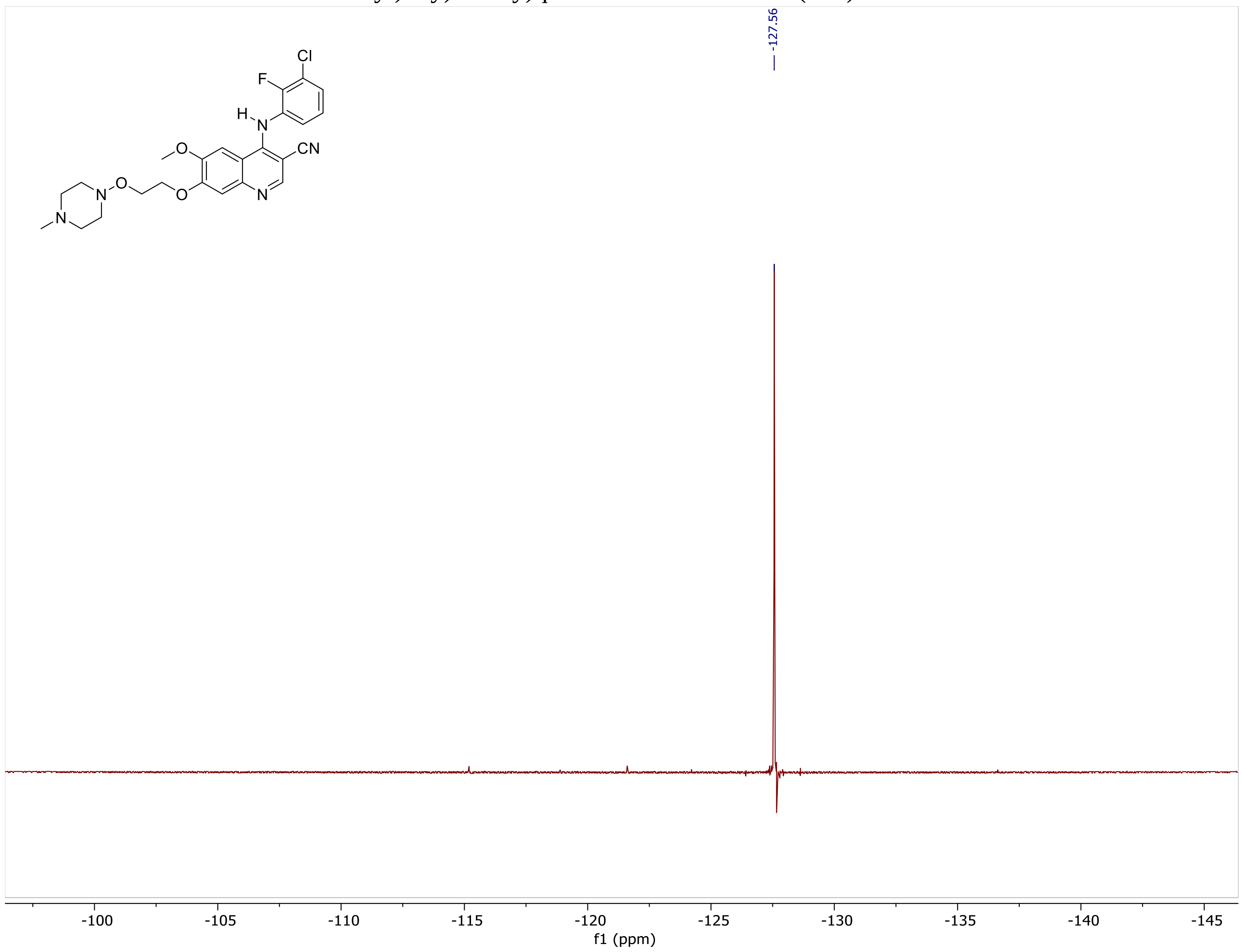

HSQC NMR (500 MHz, $CDCl_3$) spectrum of 4-((3-chloro-2-fluorophenyl)amino)-6-methoxy-7-(2-((4-methylpiperazin-1-yl)oxy)ethoxy)quinoline-3-carbonitrile (**11a**).

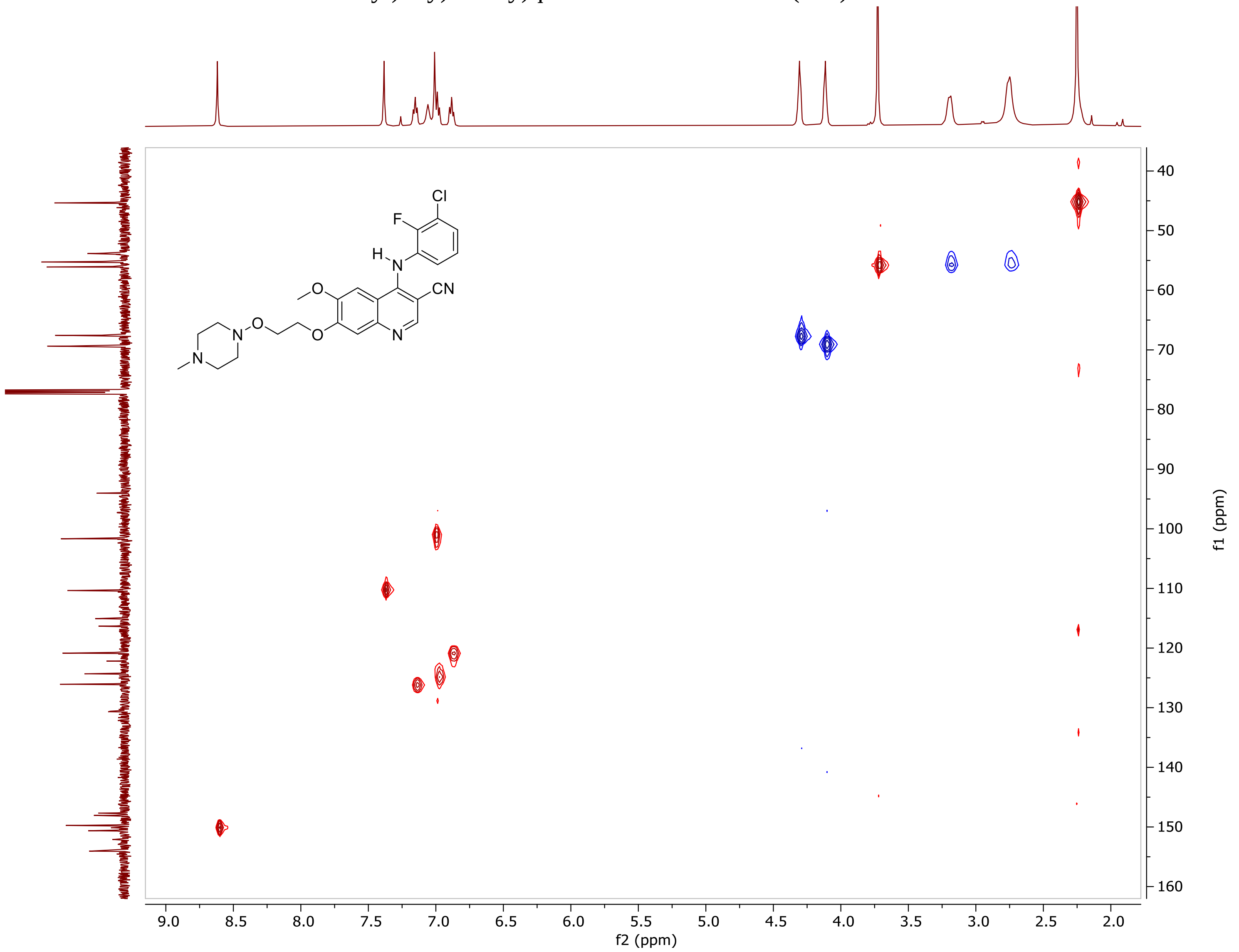

DQF-COSY NMR (500 MHz, $CDCl_3$) spectrum of 4-((3-chloro-2-fluorophenyl)amino)-6-methoxy-7-(2-((4-methylpiperazin-1-yl)oxy)ethoxy)quinoline-3-carbonitrile (**11a**).

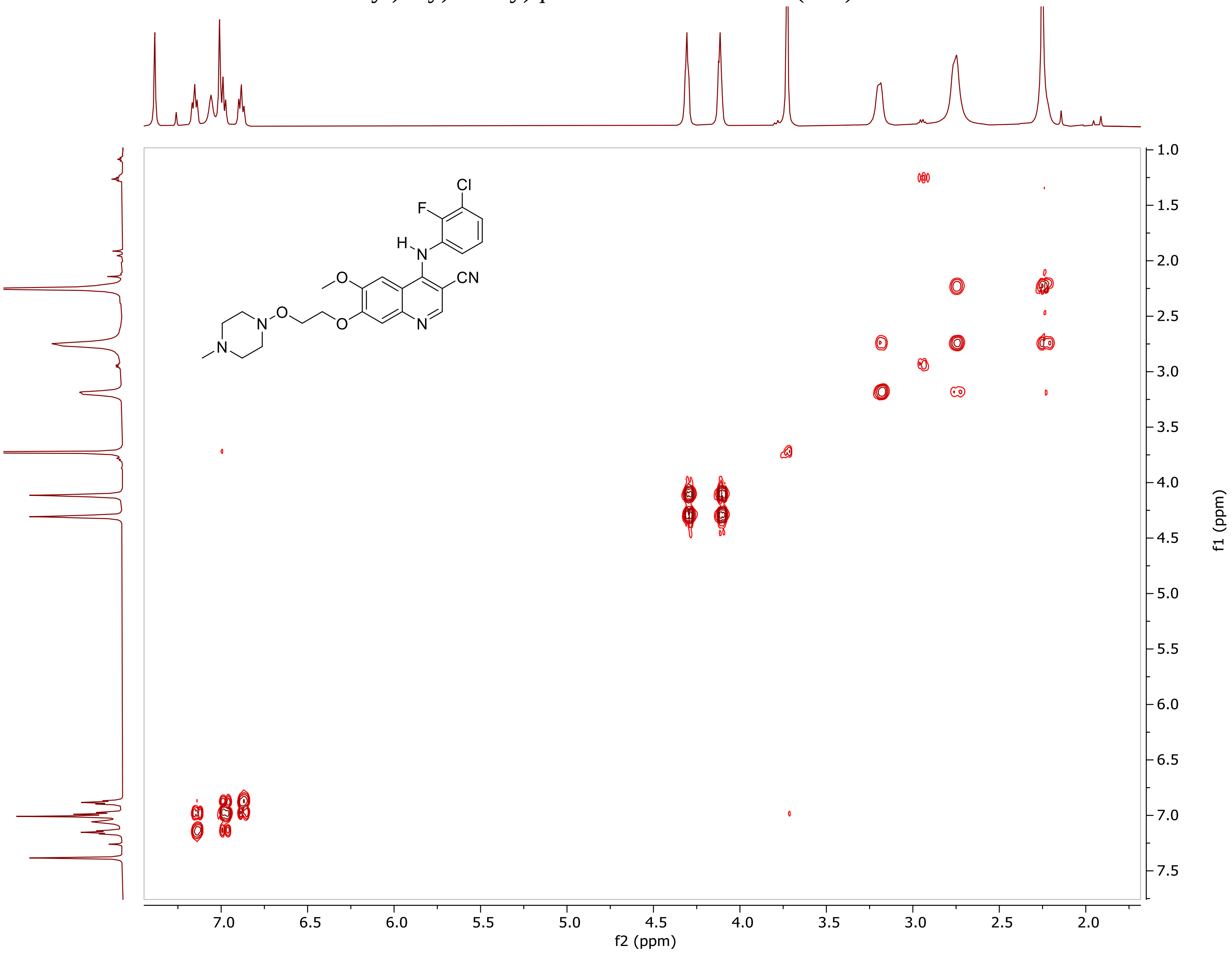

Expanded region of stacked ^{13}C **NMR** (126 MHz, $CDCl_3$) spectrum of 4-((3-chloro-2-fluorophenyl)amino)-6-methoxy-7-(2-((4-methylpiperazin-1-yl)oxy)ethoxy)quinoline-3-carbonitrile (**11a**) at a) 328K and b) 298 K.

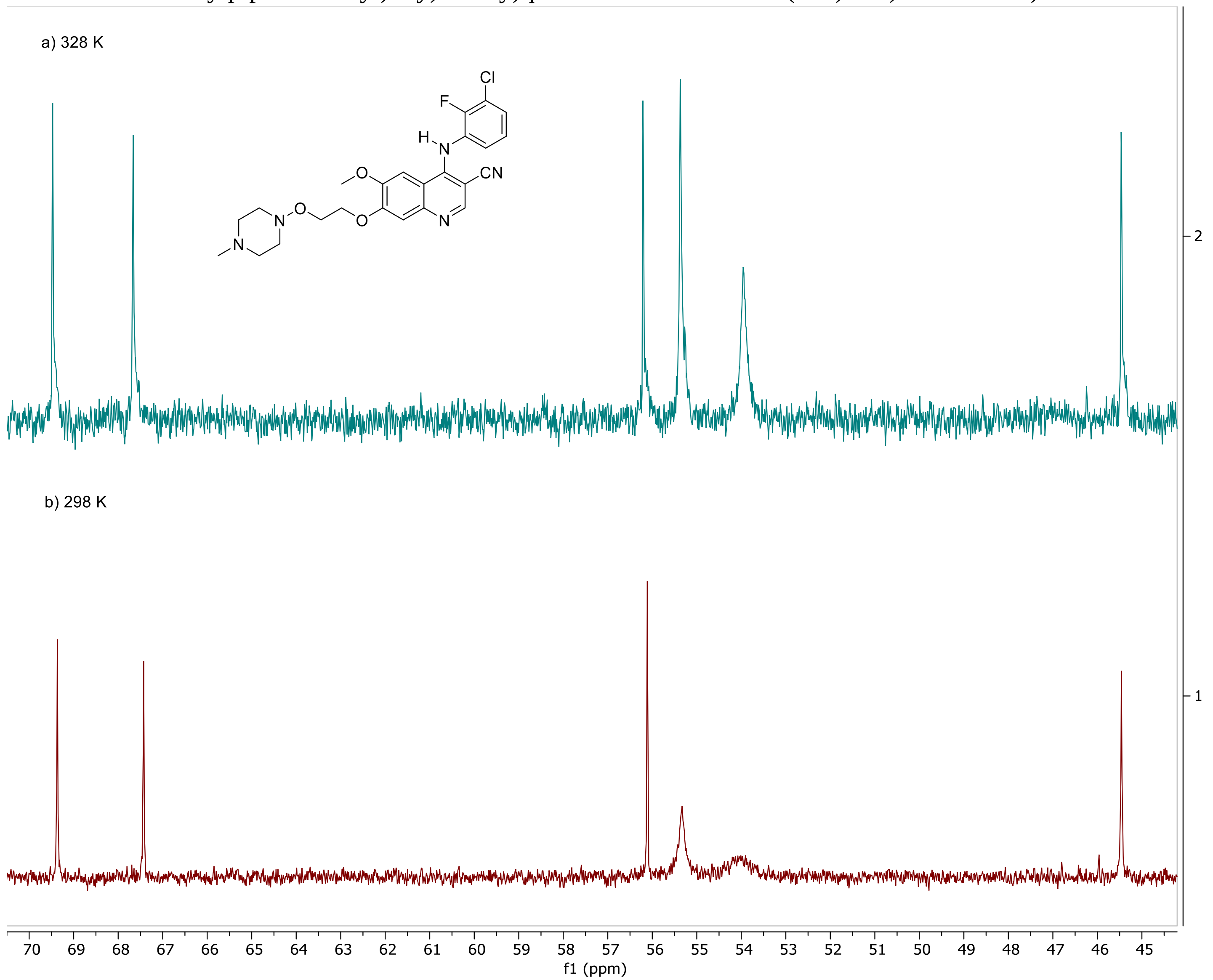

Expanded region of **HSQC NMR** (500 MHz, $CDCl_3$, 5 mM solution) spectrum of 4-((3-chloro-2-fluorophenyl)amino)-6-methoxy-7-(2-((4-methylpiperazin-1-yl)oxy)ethoxy)quinoline-3-carbonitrile (**11a**).

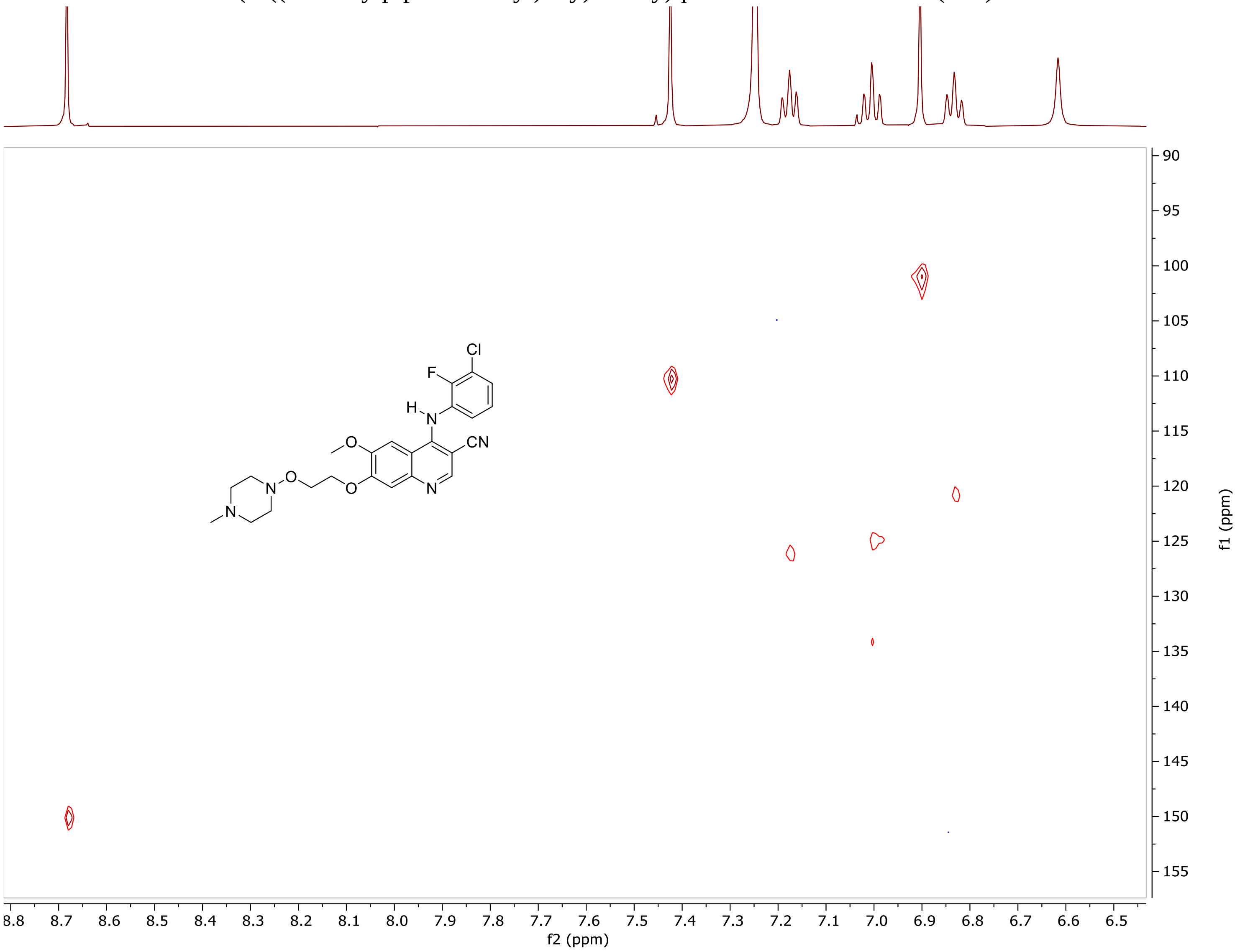

Expanded region of ^{1}**H NMR** (500 MHz, $CDCl_3$, 5 mM solution) spectrum of 4-((3-chloro-2-fluorophenyl)amino)-6-methoxy-7-(2-((4-methylpiperazin-1-yl)oxy)ethoxy)quinoline-3-carbonitrile (**11a**).

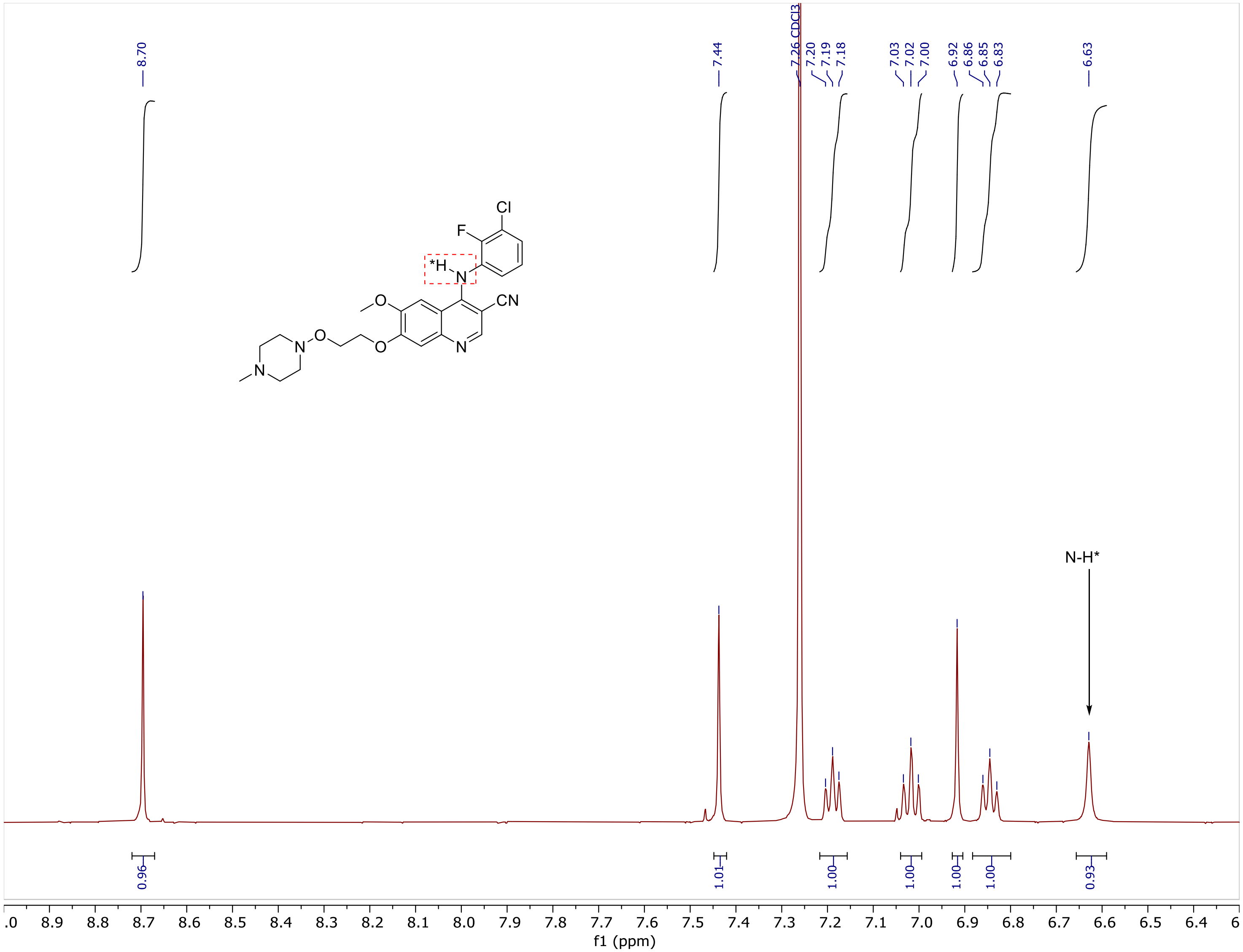

Expanded region of **HSQC** (500 MHz, DMSO-D6, 5 mM solution) spectrum of 4-((3-chloro-2-fluorophenyl)amino)-6-methoxy-7-(2-((4-methylpiperazin-1-yl)oxy)ethoxy)quinoline-3-carbonitrile (**11a**).

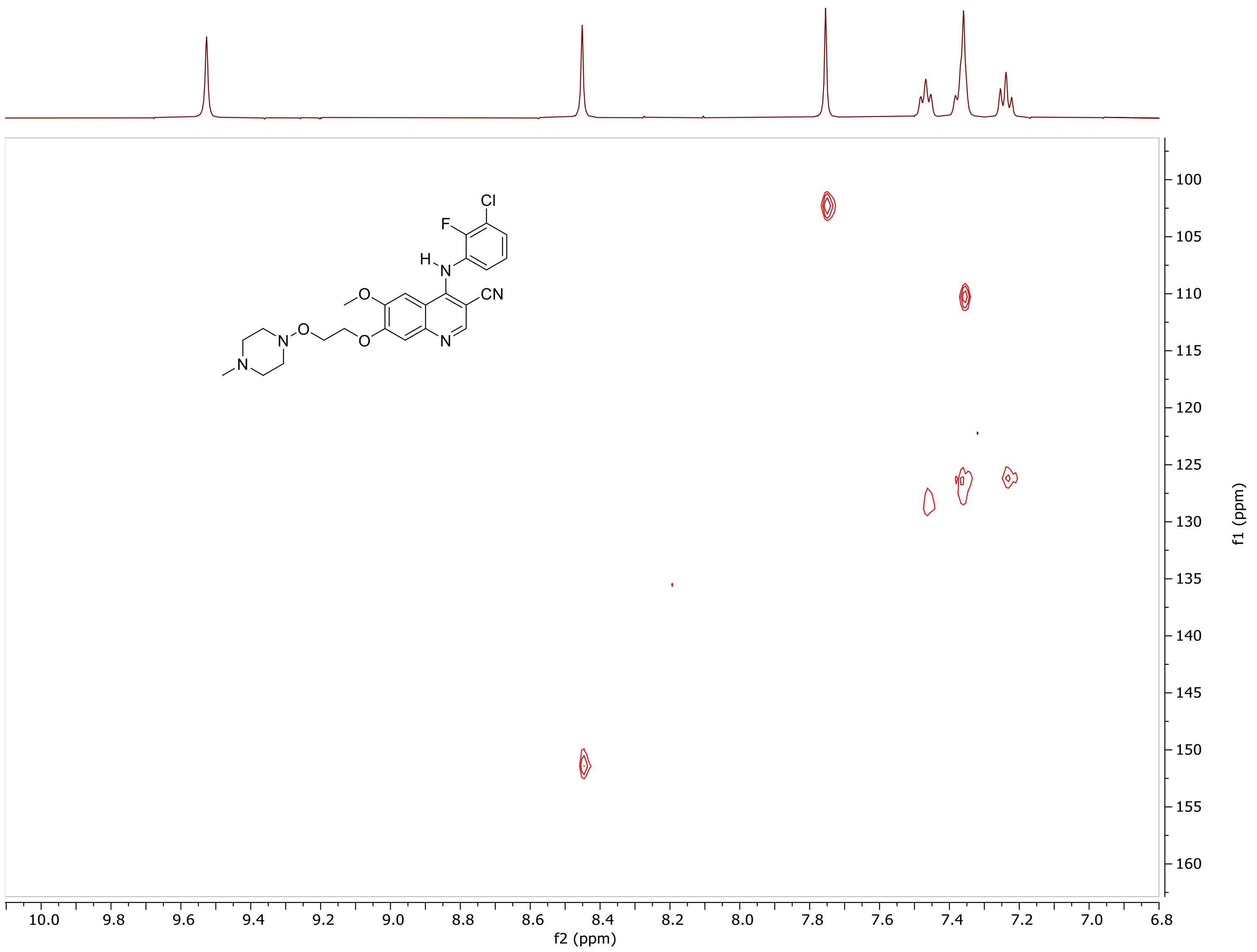

Expanded region of **^{1}H NMR** (500 MHz, DMSO-D$_6$, 5 mM solution) spectrum of 4-((3-chloro-2-fluorophenyl)amino)-6-methoxy-7-(2-((4-methylpiperazin-1-yl)oxy)ethoxy)quinoline-3-carbonitrile (**11a**).

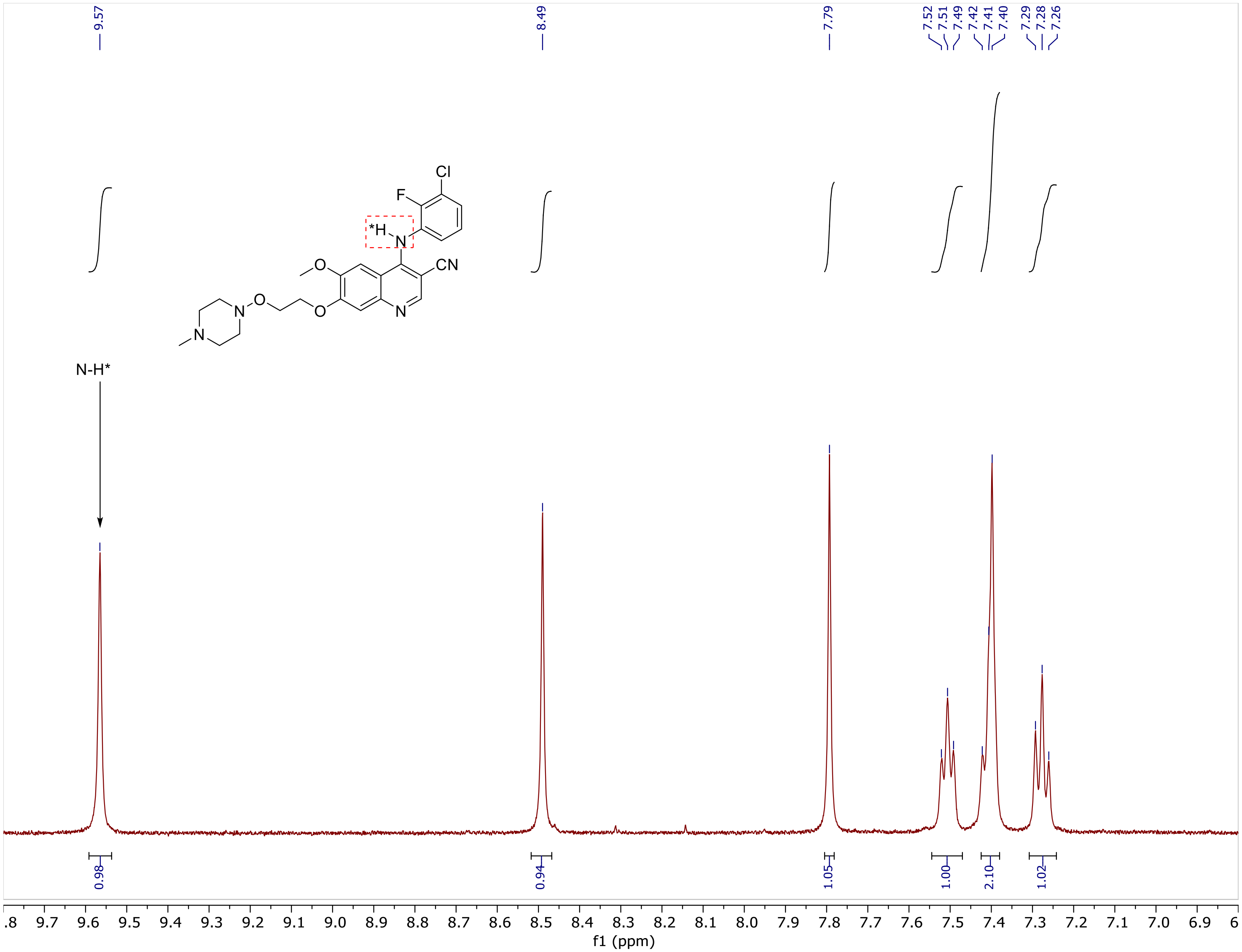

^{1}H NMR (500 MHz, $CDCl_3$) spectrum of 4-((2-chloro-3-methoxyphenyl)amino)-6-methoxy-7-(2-((4-methylpiperazin-1-yl)oxy)ethoxy)quinoline-3-carbonitrile (**12a**).

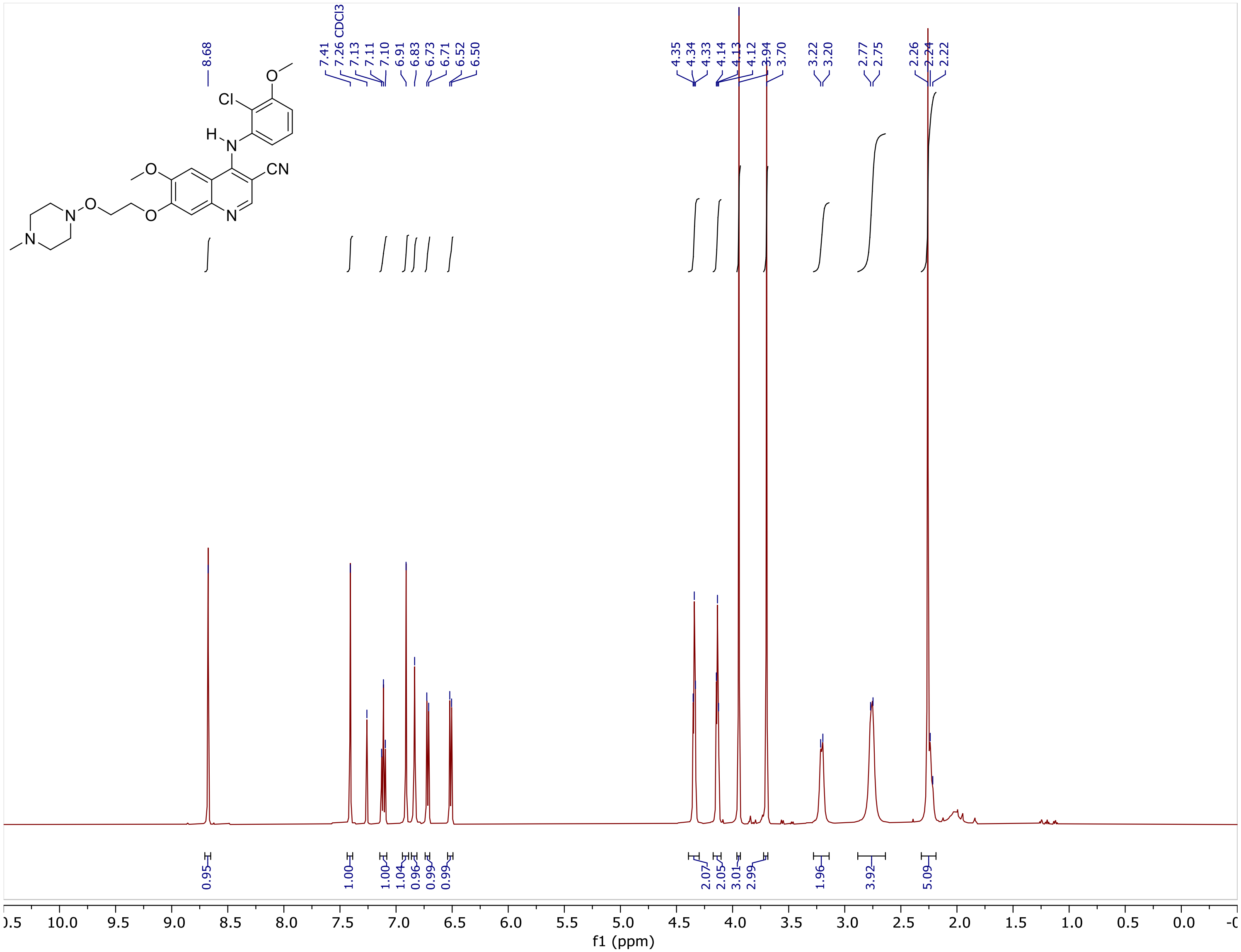

^{13}C NMR (126 MHz, $CDCl_3$) spectrum of 4-((2-chloro-3-methoxyphenyl)amino)-6-methoxy-7-(2-((4-methylpiperazin-1-yl)oxy)ethoxy)quinoline-3-carbonitrile (**12a**).

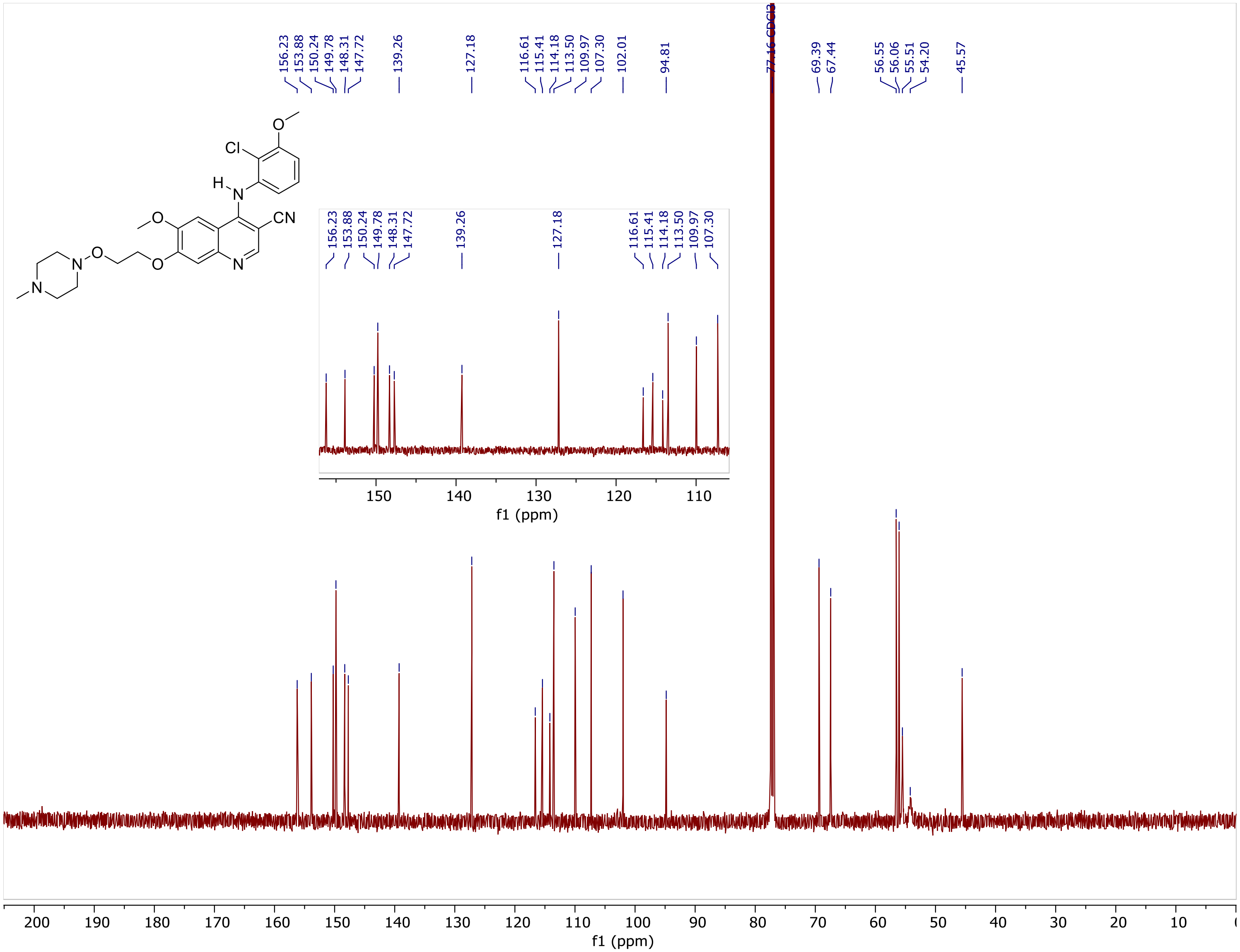

HSQC NMR (500 MHz, $CDCl_3$) spectrum of 4-((2-chloro-3-methoxyphenyl)amino)-6-methoxy-7-(2-((4-methylpiperazin-1-yl)oxy)ethoxy)quinoline-3-carbonitrile (**12a**).

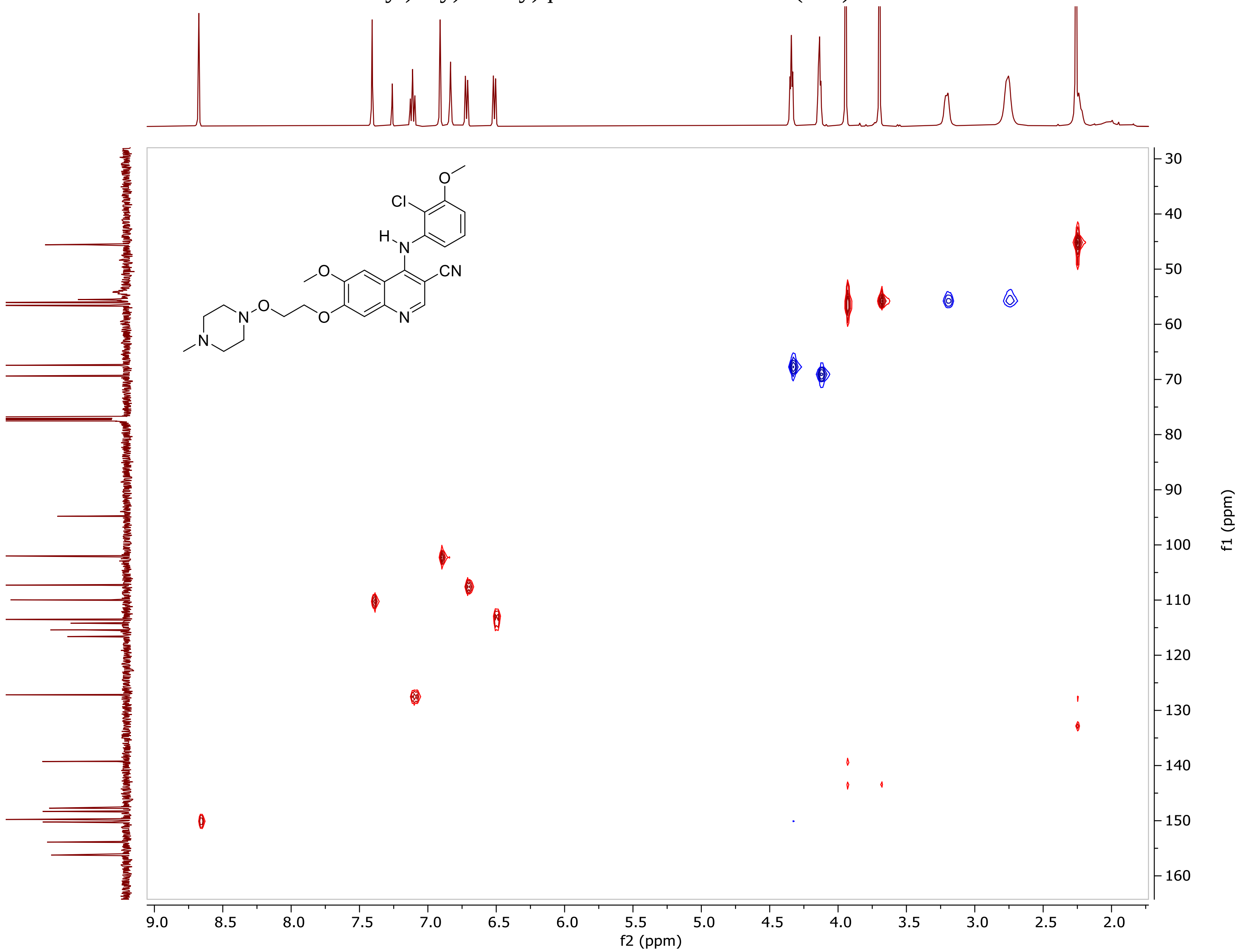

DQF-COSY NMR (500 MHz, $CDCl_3$) spectrum of 4-((2-chloro-3-methoxyphenyl)amino)-6-methoxy-7-(2-((4-methylpiperazin-1-yl)oxy)ethoxy)quinoline-3-carbonitrile (**12a**).

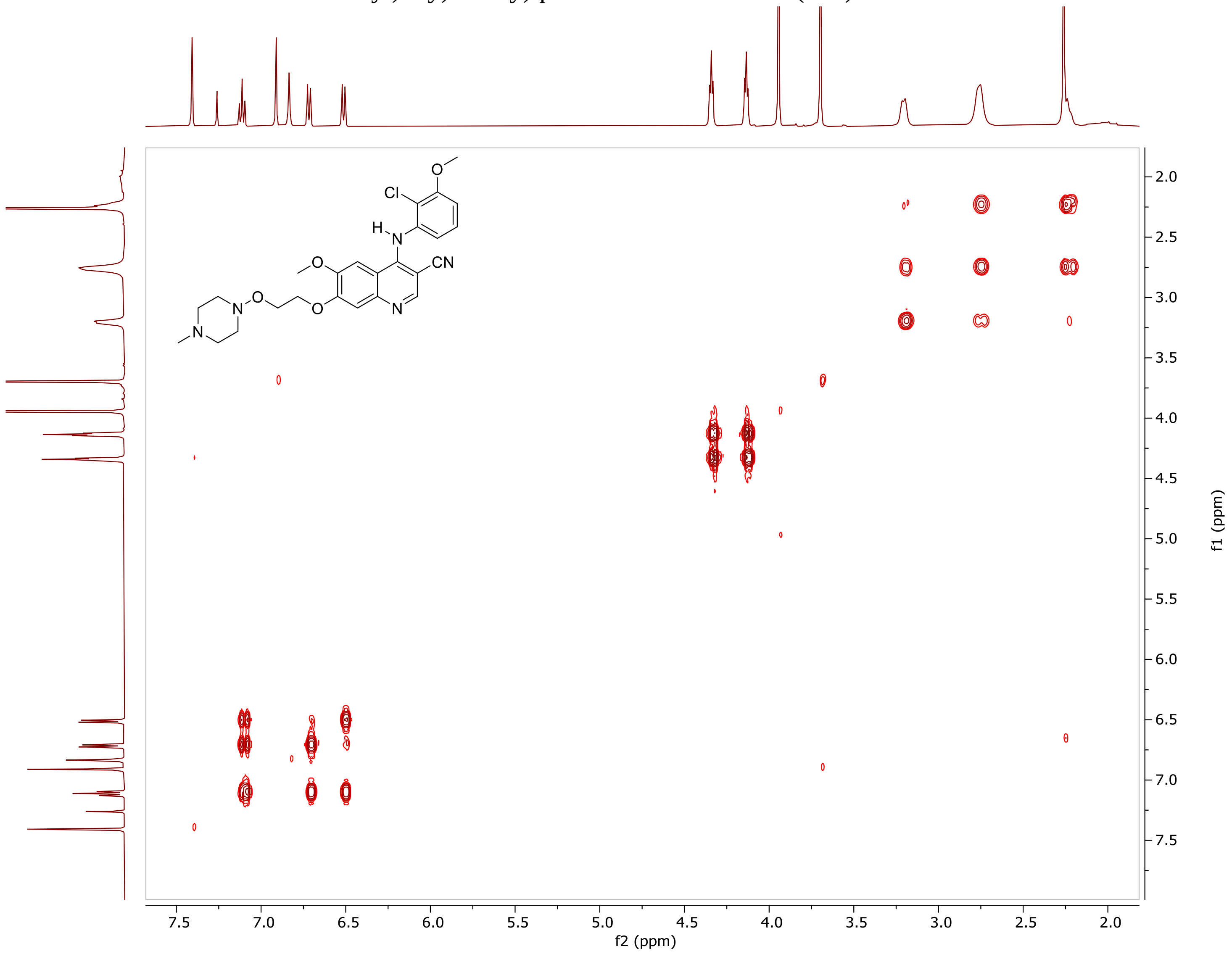

Expanded region of stacked [13]**C NMR** (126 MHz, $CDCl_3$) spectrum of 4-((2-chloro-3-methoxyphenyl)amino)-6-methoxy-7-(2-((4-methylpiperazin-1-yl)oxy)ethoxy)quinoline-3-carbonitrile (**12a**) at a) 328K and b) 298 K.

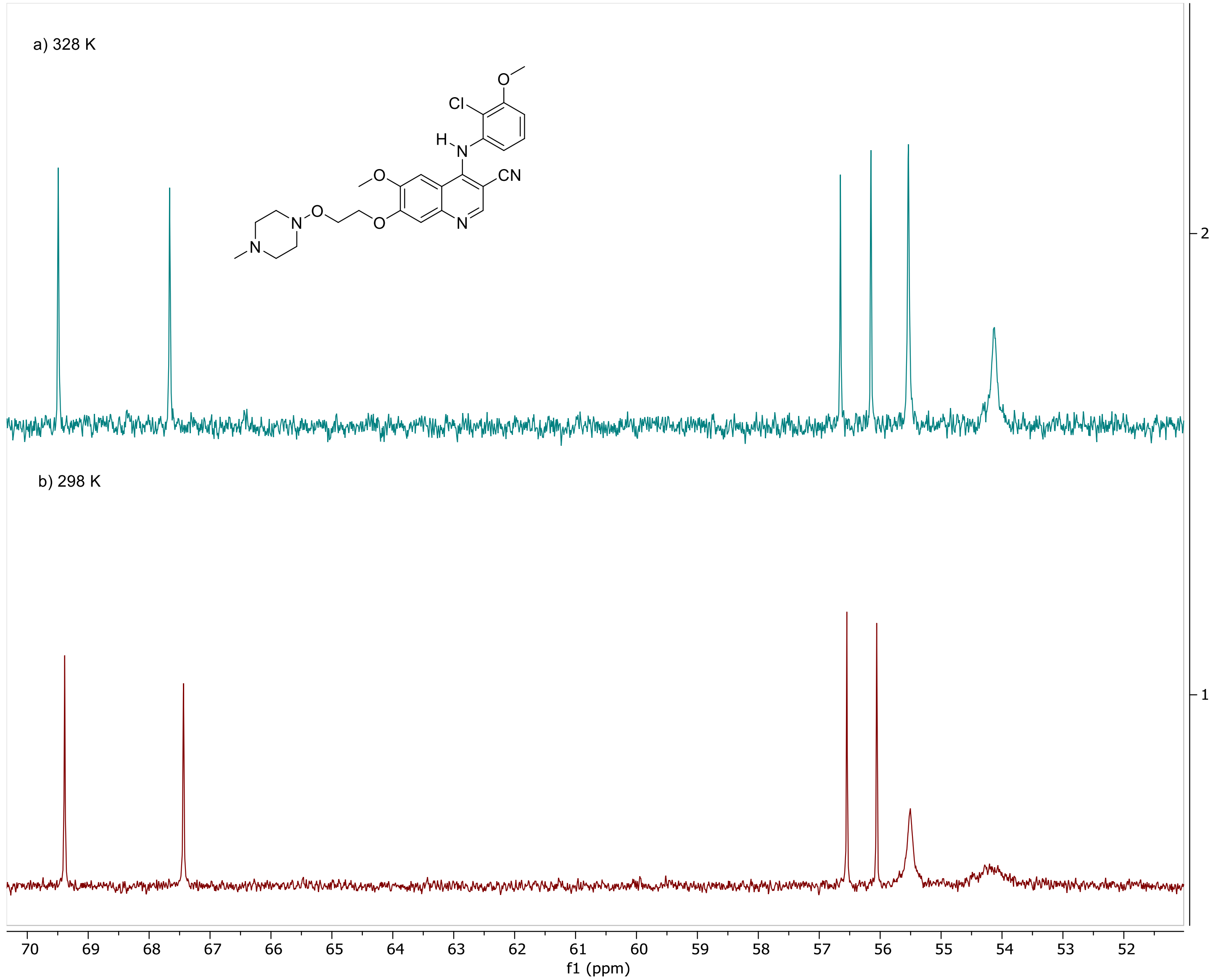

^{1}H NMR (500 MHz, $CDCl_3$) spectrum of 4-((2-fluoro-3-methoxyphenyl)amino)-6-methoxy-7-(2-((4-methylpiperazin-1-yl)oxy)ethoxy)quinoline-3-carbonitrile (**13a**).

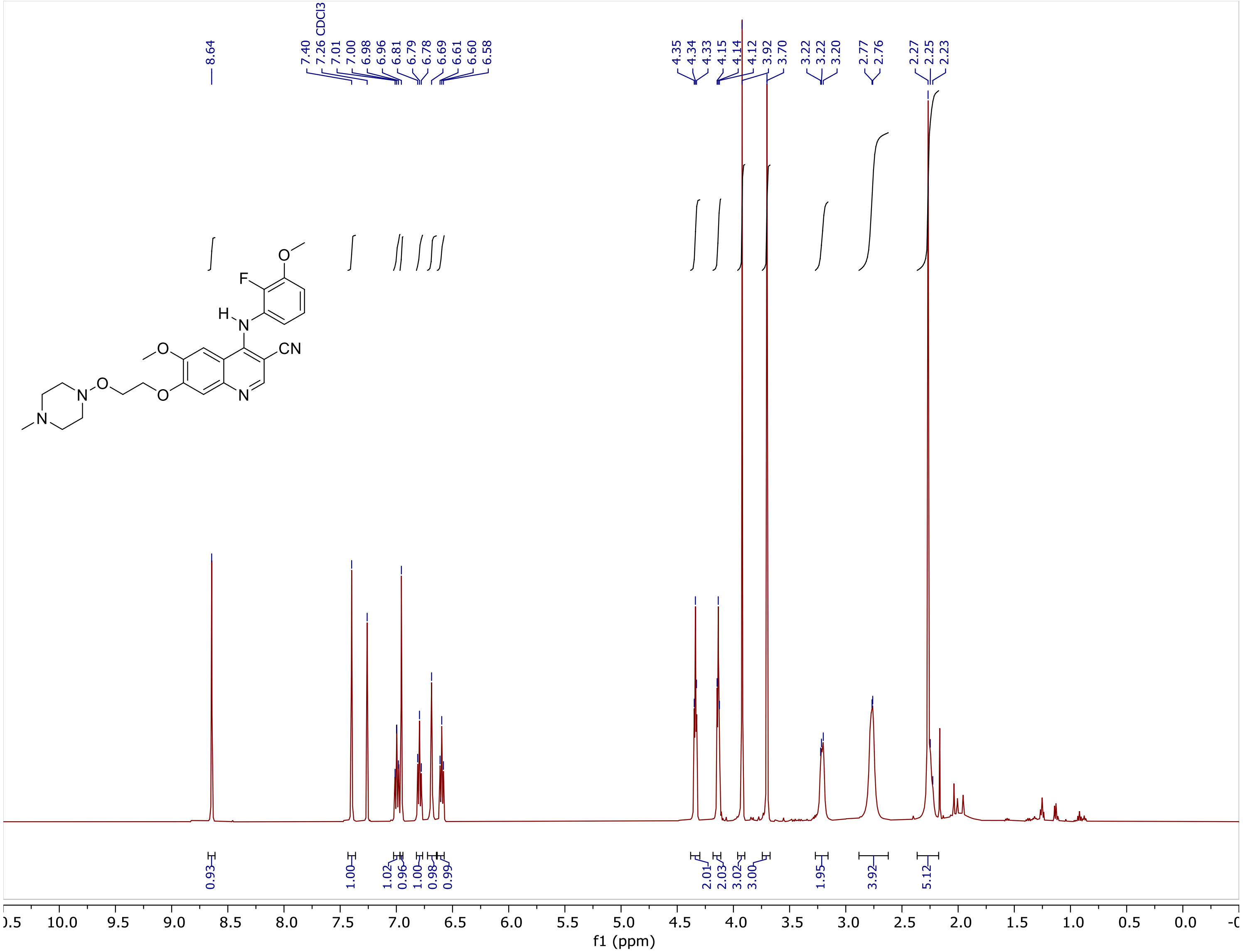

^{13}C NMR (126 MHz, CDCl$_3$) spectrum of 4-((2-fluoro-3-methoxyphenyl)amino)-6-methoxy-7-(2-((4-methylpiperazin-1-yl)oxy)ethoxy)quinoline-3-carbonitrile (**13a**).

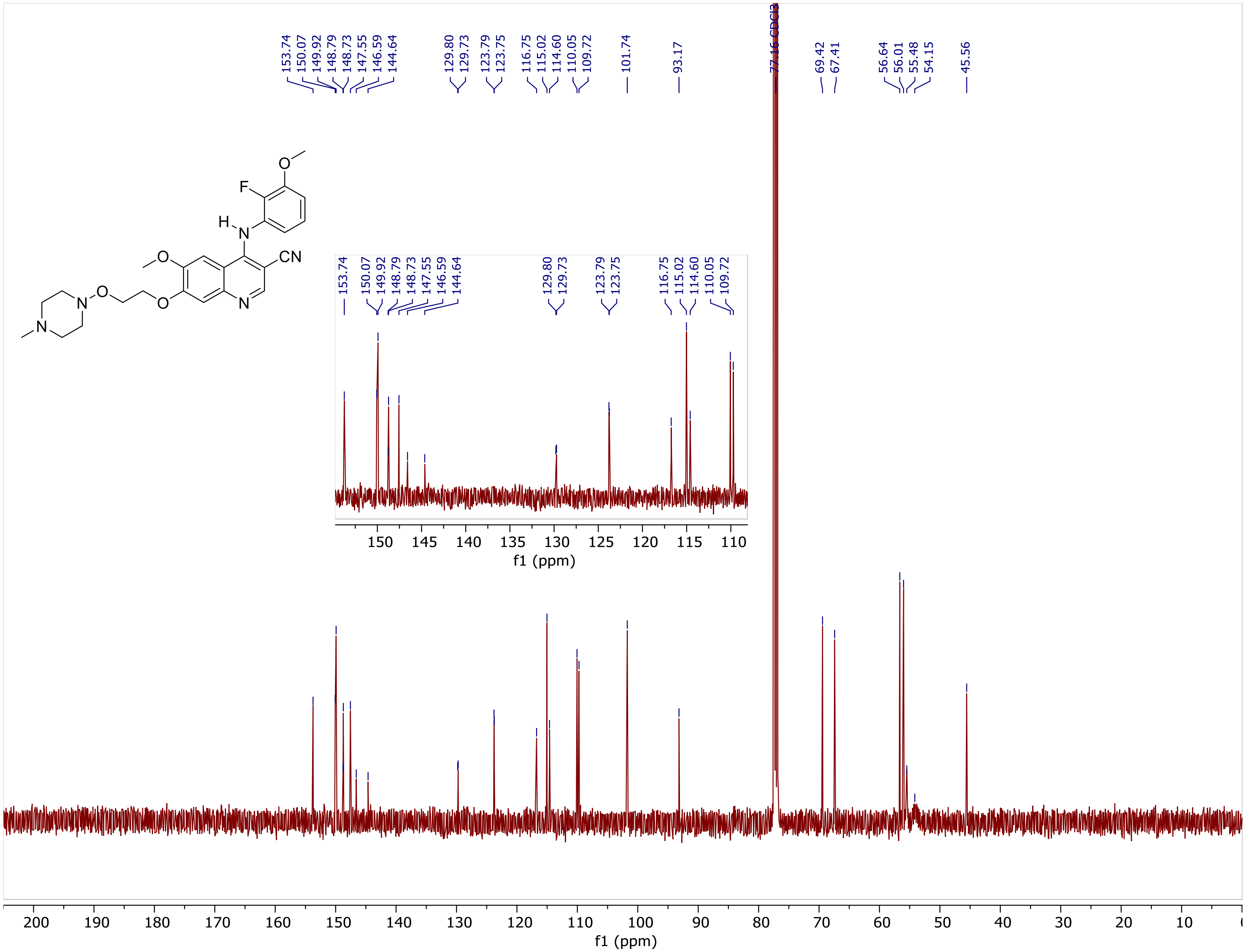

^{13}C {^{19}F} NMR (126 MHz, $CDCl_3$) spectrum of 4-((2-fluoro-3-methoxyphenyl)amino)-6-methoxy-7-(2-((4-methylpiperazin-1-yl)oxy)ethoxy)quinoline-3-carbonitrile (**13a**).

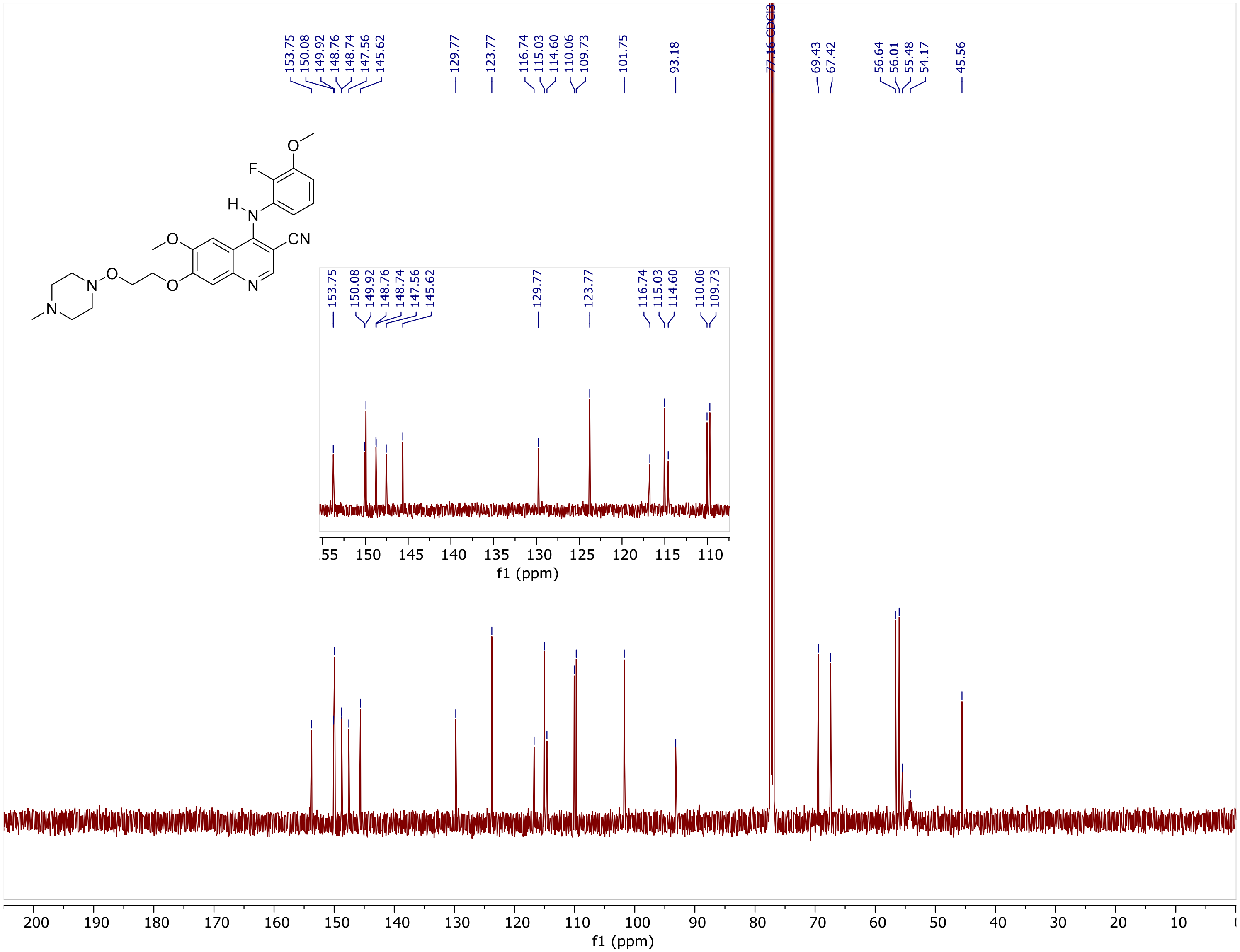

Expanded region of stacked a) ^{13}C {^{19}F} NMR (126 MHz, CDCl$_3$) and b) ^{13}C NMR (126 MHz, CDCl$_3$) spectrum of 4-((2-fluoro-3-methoxyphenyl)amino)-6-methoxy-7-(2-((4-methylpiperazin-1-yl)oxy)ethoxy)quinoline-3-carbonitrile (**13a**).

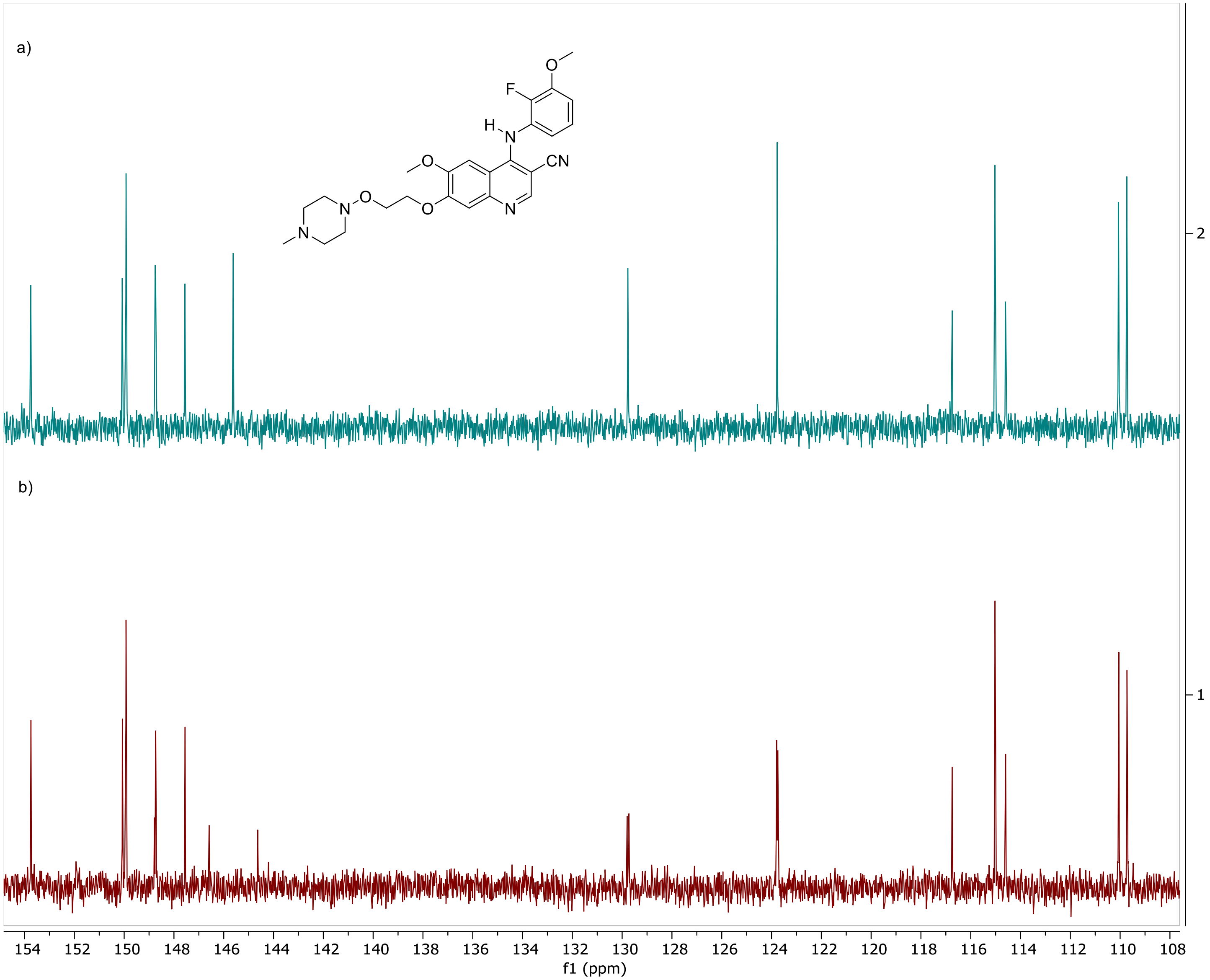

^{19}F {^{1}H} NMR (476 MHz, $CDCl_3$) spectrum of 4-((2-fluoro-3-methoxyphenyl)amino)-6-methoxy-7-(2-((4-methylpiperazin-1-yl)oxy)ethoxy)quinoline-3-carbonitrile (**13a**).

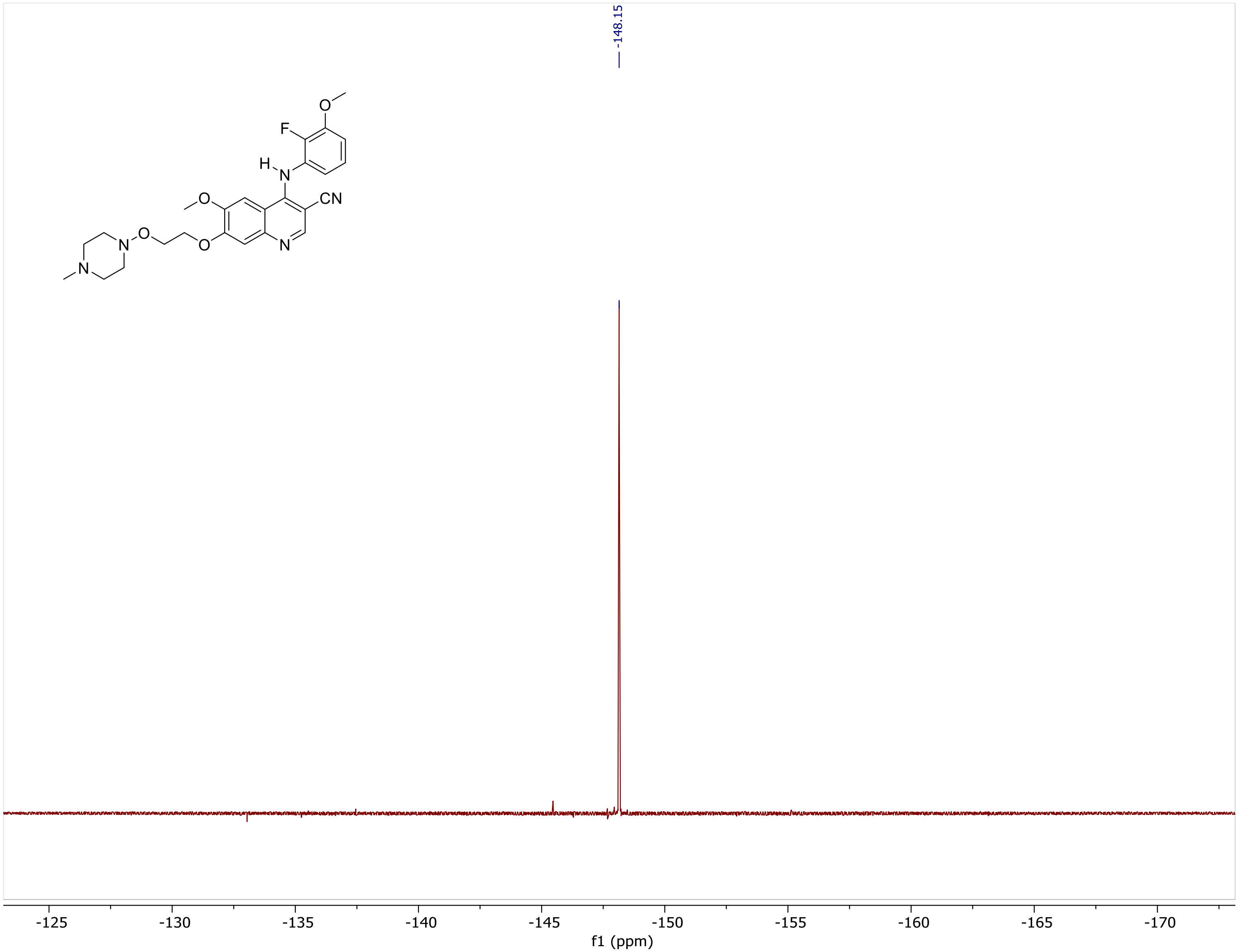

HSQC NMR (500 MHz, $CDCl_3$) spectrum of 4-((2-fluoro-3-methoxyphenyl)amino)-6-methoxy-7-(2-((4-methylpiperazin-1-yl)oxy)ethoxy)quinoline-3-carbonitrile (**13a**).

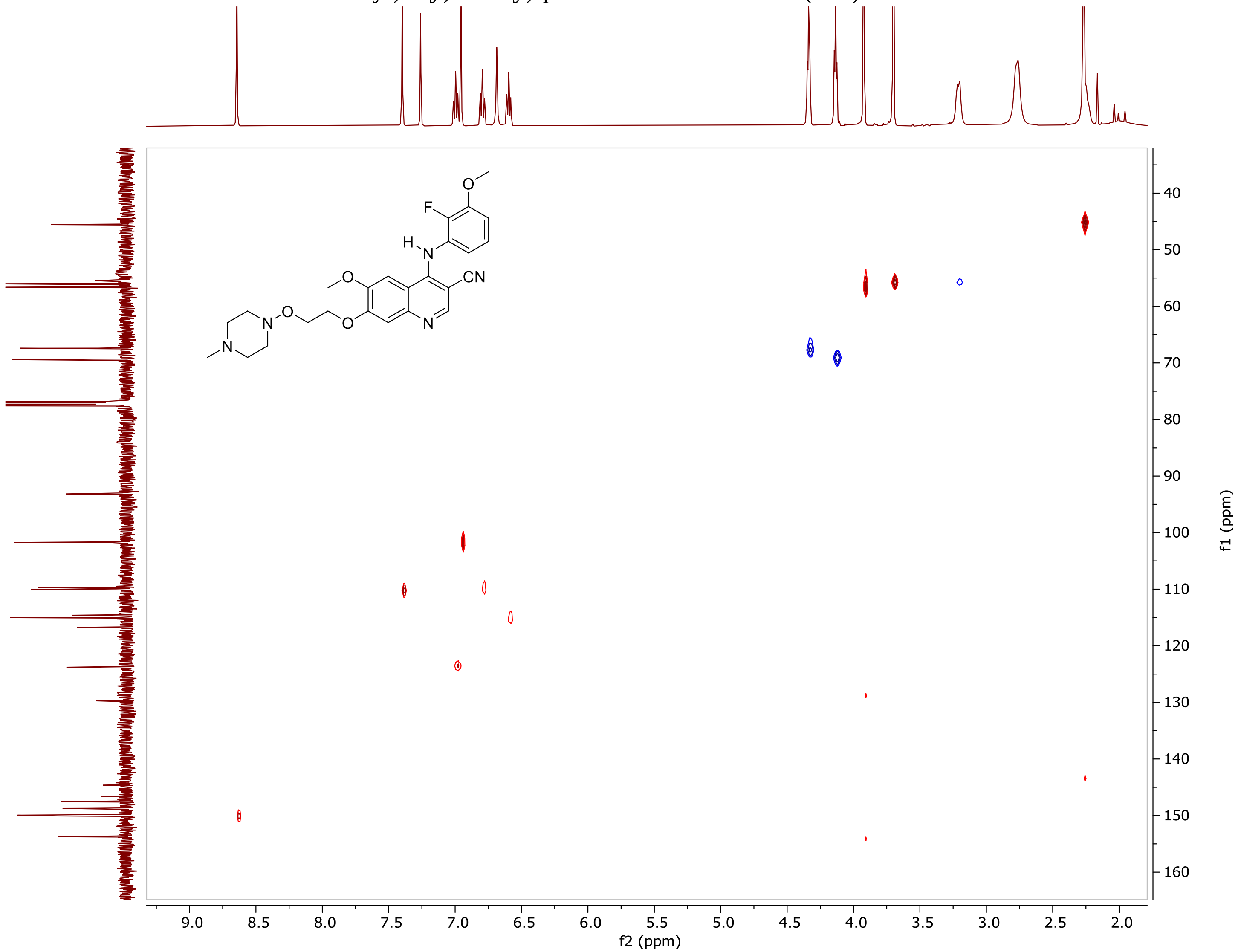

DQF-COSY NMR (500 MHz, $CDCl_3$) spectrum of 4-((2-fluoro-3-methoxyphenyl)amino)-6-methoxy-7-(2-((4-methylpiperazin-1-yl)oxy)ethoxy)quinoline-3-carbonitrile (**13a**).

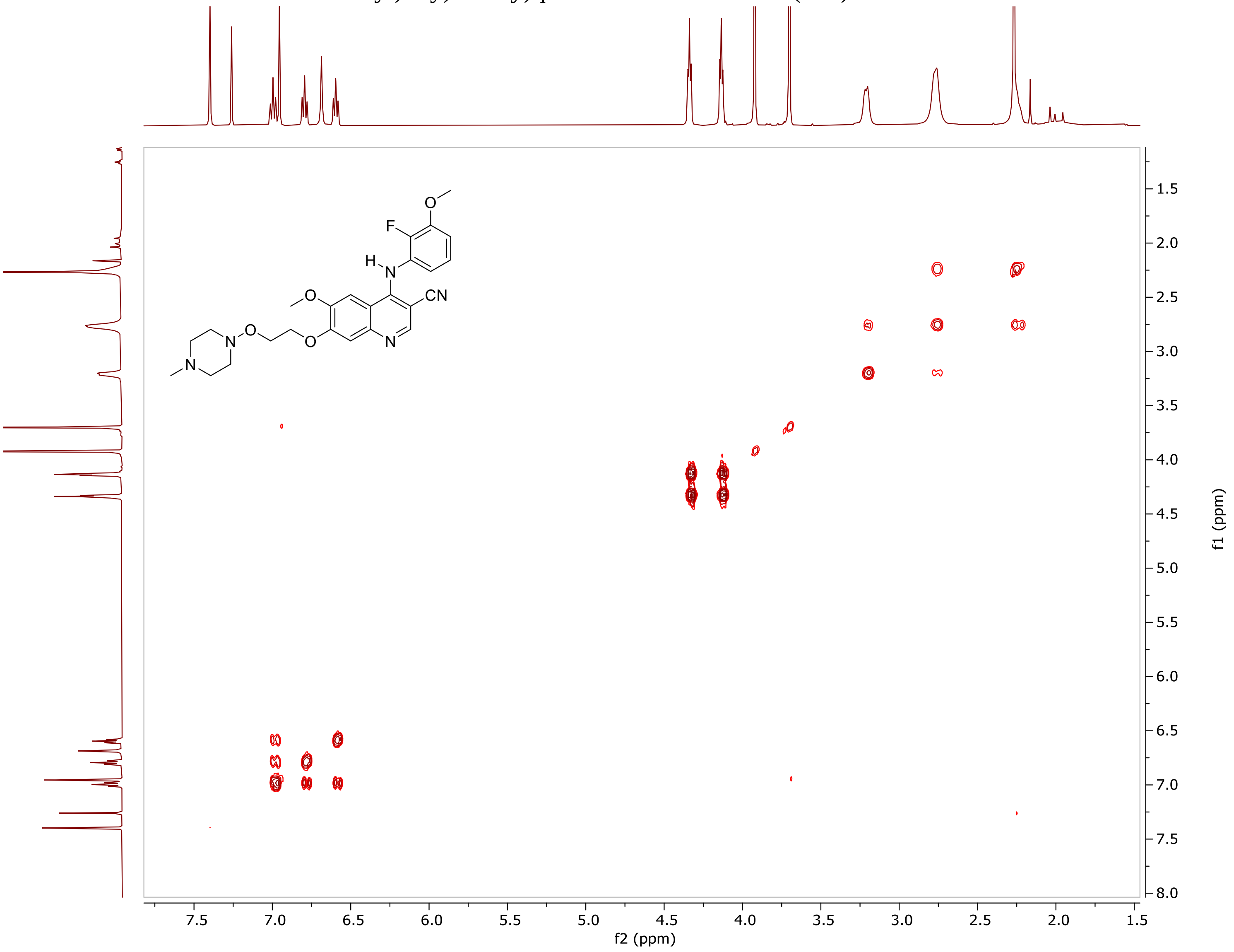

Expanded region of stacked **^{13}C NMR** (126 MHz, $CDCl_3$) spectrum of 4-((2-fluoro-3-methoxyphenyl)amino)-6-methoxy-7-(2-((4-methylpiperazin-1-yl)oxy)ethoxy)quinoline-3-carbonitrile (**13a**).at a) 328K and b) 298 K.

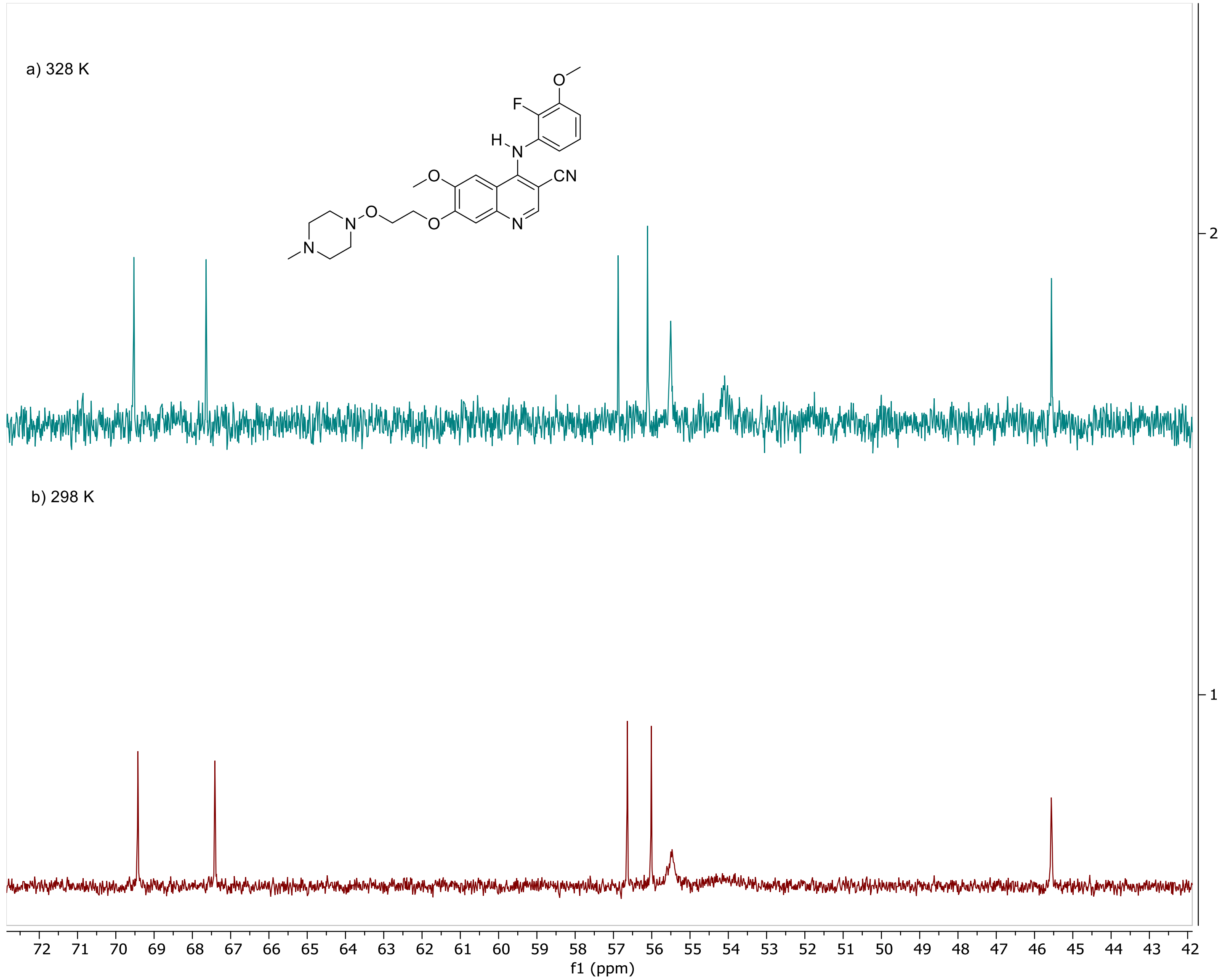

^{1}H NMR (500 MHz, $CDCl_3$) spectrum of 4-((4-chloro-3-methoxyphenyl)amino)-6-methoxy-7-(2-((4-methylpiperazin-1-yl)ethoxy)quinoline-3-carbonitrile (**14a**).

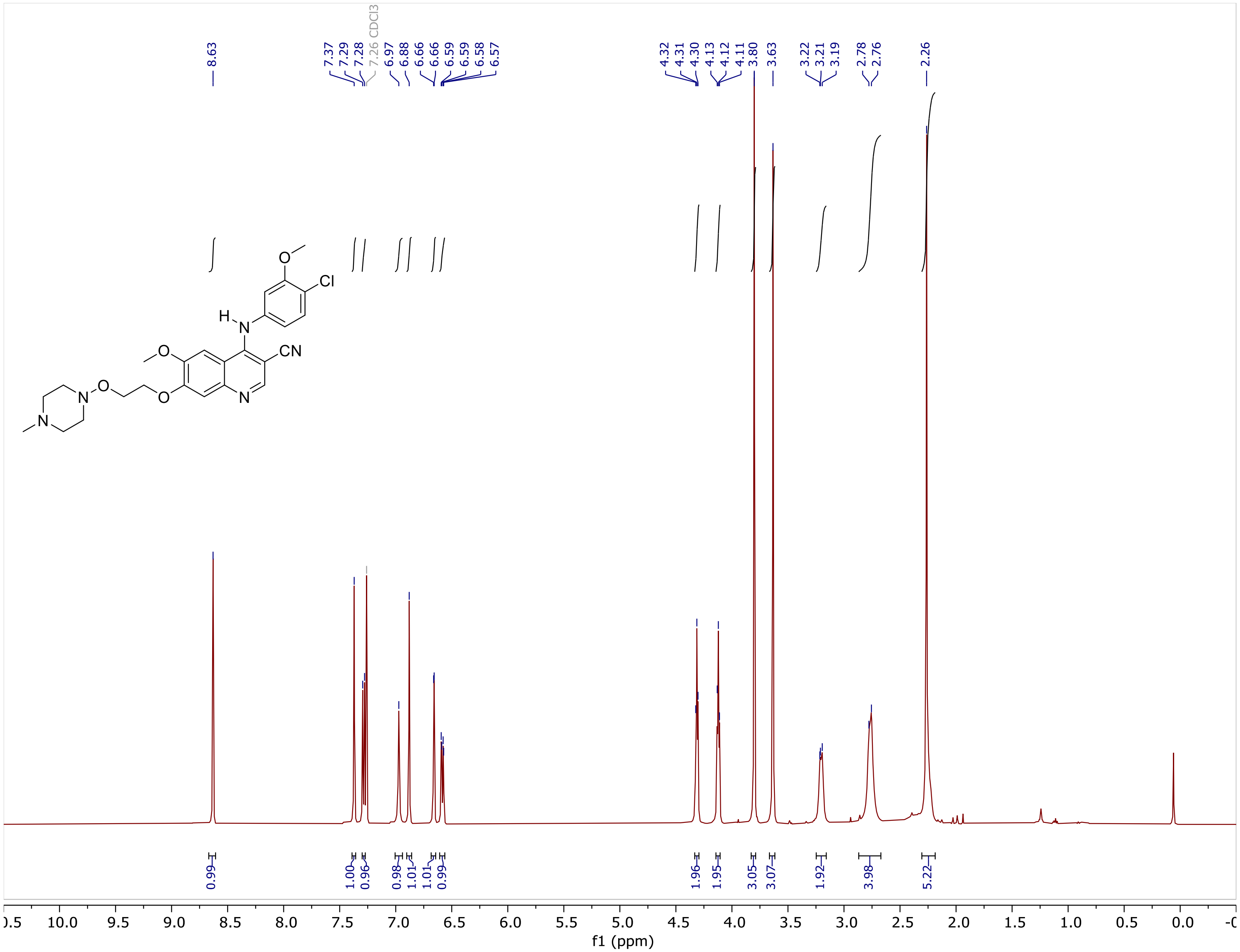

^{13}C NMR (126 MHz, $CDCl_3$) spectrum of 4-((4-chloro-3-methoxyphenyl)amino)-6-methoxy-7-(2-((4-methylpiperazin-1-yl)ethoxy)quinoline-3-carbonitrile (**14a**).

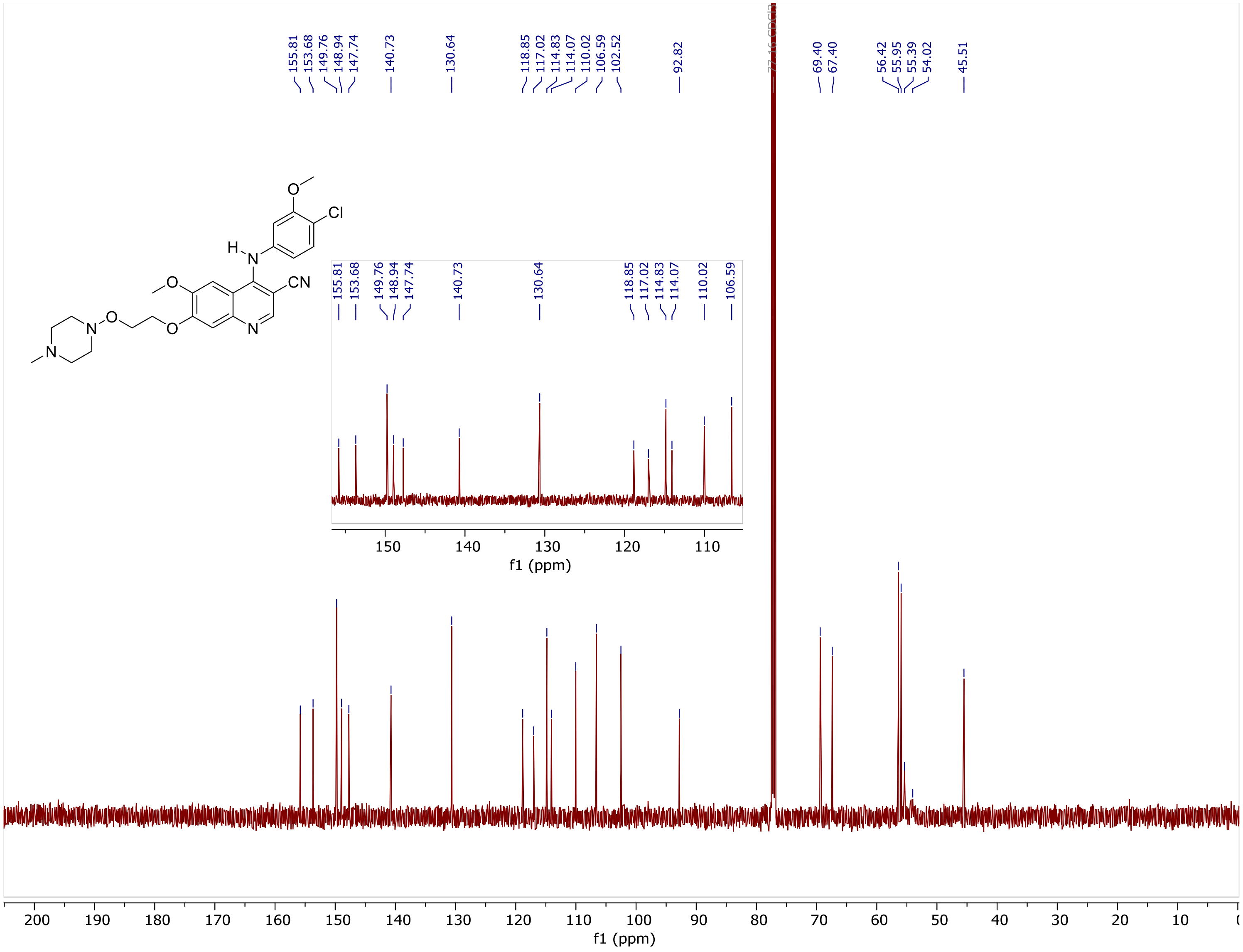

DQF-COSY NMR (500 MHz, $CDCl_3$) spectrum of 4-((4-chloro-3-methoxyphenyl)amino)-6-methoxy-7-(2-((4-methylpiperazin-1-yl)ethoxy)quinoline-3-carbonitrile (**14a**).

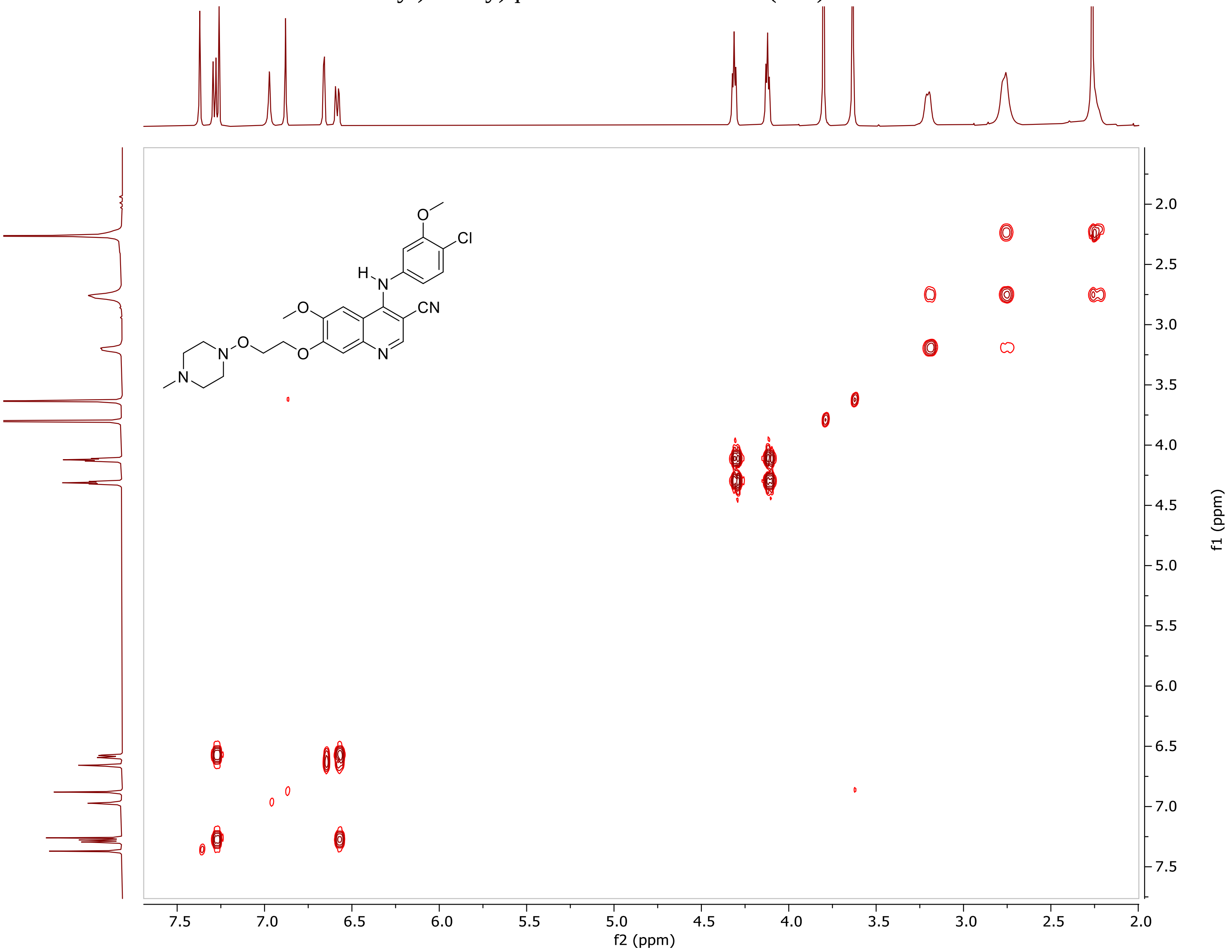

HSQC NMR (500 MHz, $CDCl_3$) spectrum of 4-((4-chloro-3-methoxyphenyl)amino)-6-methoxy-7-(2-((4-methylpiperazin-1-yl)ethoxy)quinoline-3-carbonitrile (**14a**).

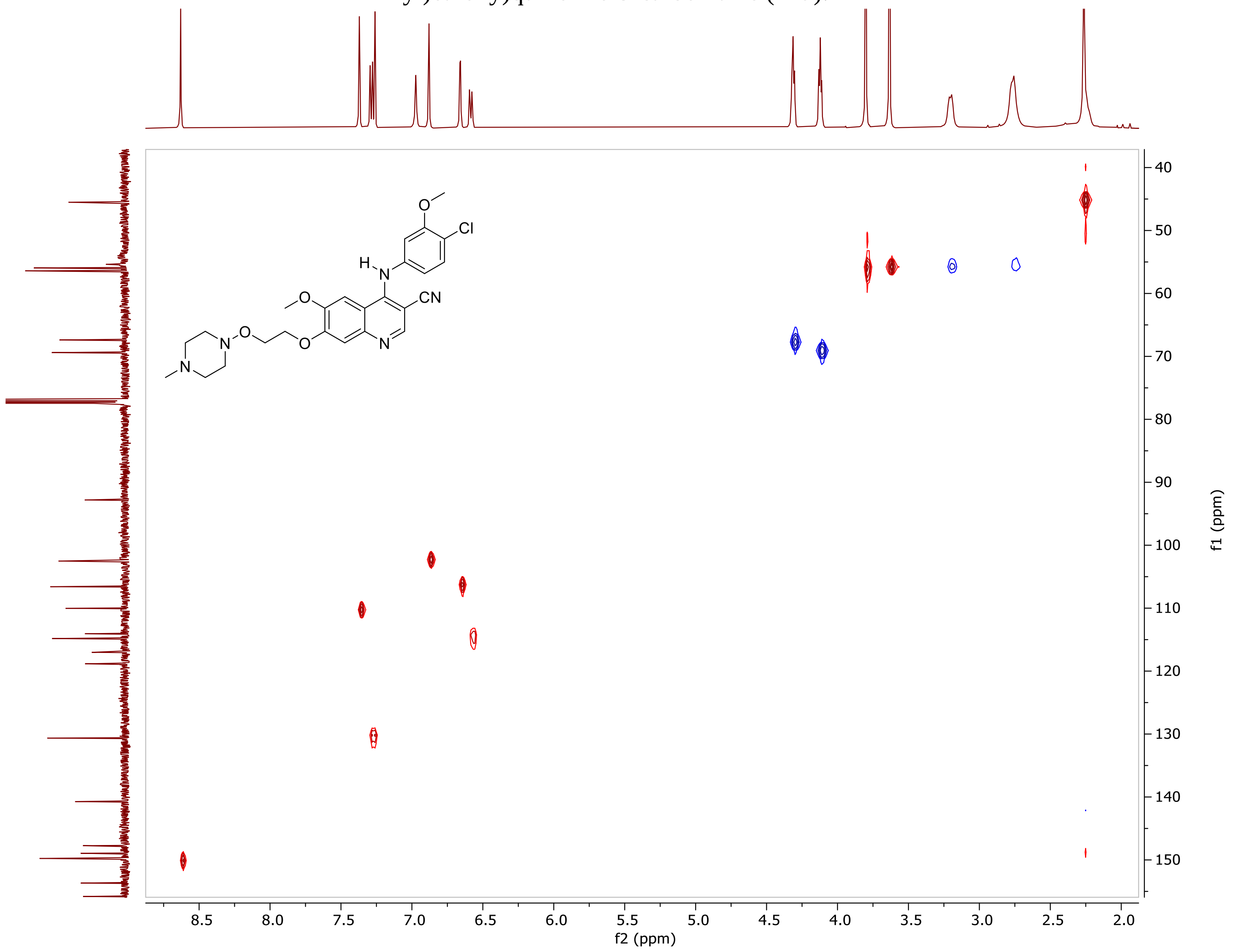

Expanded region of stacked **^{13}C NMR** (126 MHz, $CDCl_3$) spectrum of 4-((4-chloro-3-methoxyphenyl)amino)-6-methoxy-7-(2-((4-methylpiperazin-1-yl)ethoxy)quinoline-3-carbonitrile (**14a**) at a) 328K and b) 298 K.

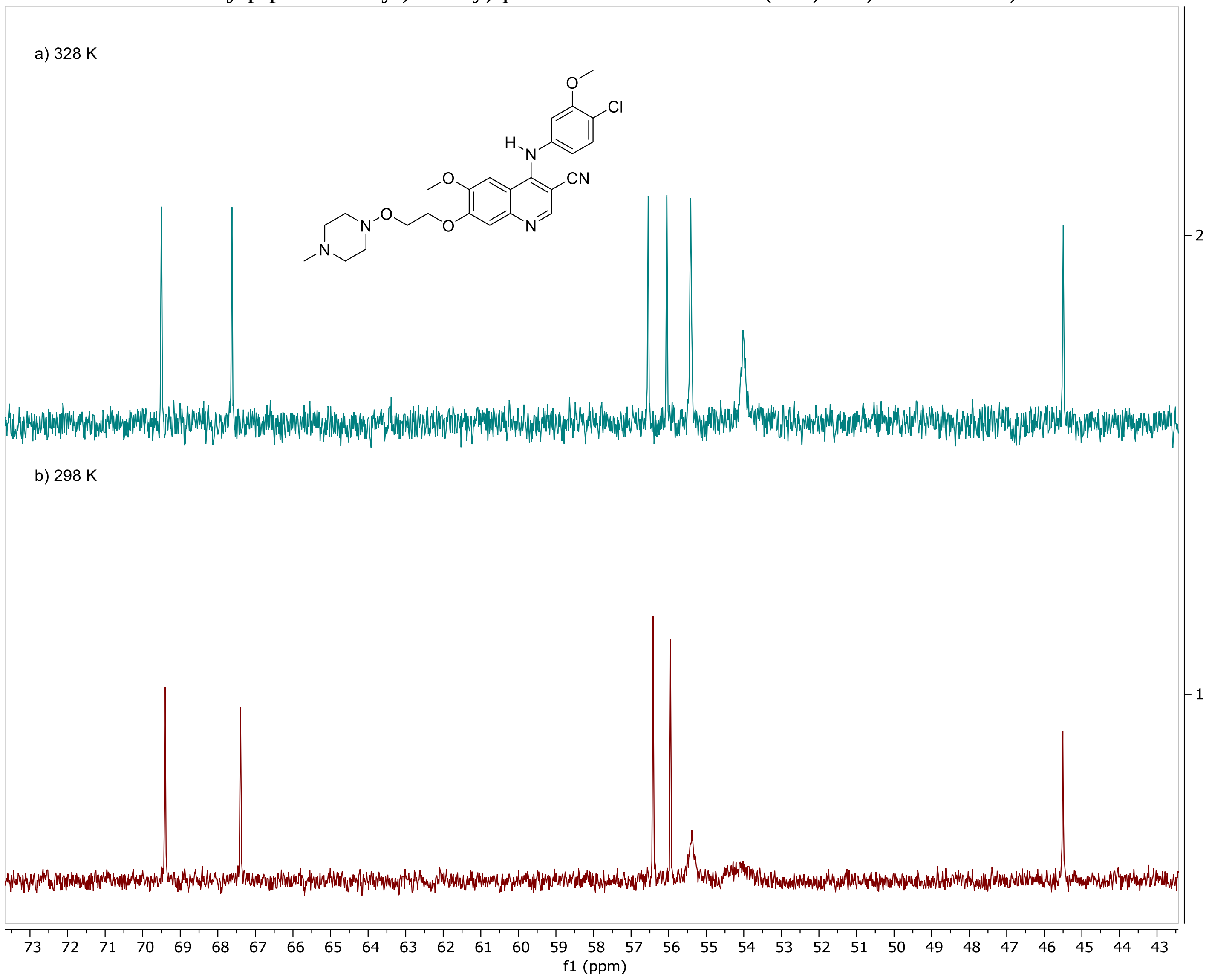

¹H NMR (500 MHz, $CDCl_3$) spectrum of 4-((4-chloro-2-methoxyphenyl)amino)-6-methoxy-7-(2-((4-methylpiperazin-1-yl)oxy)ethoxy)quinoline-3-carbonitrile (**15a**).

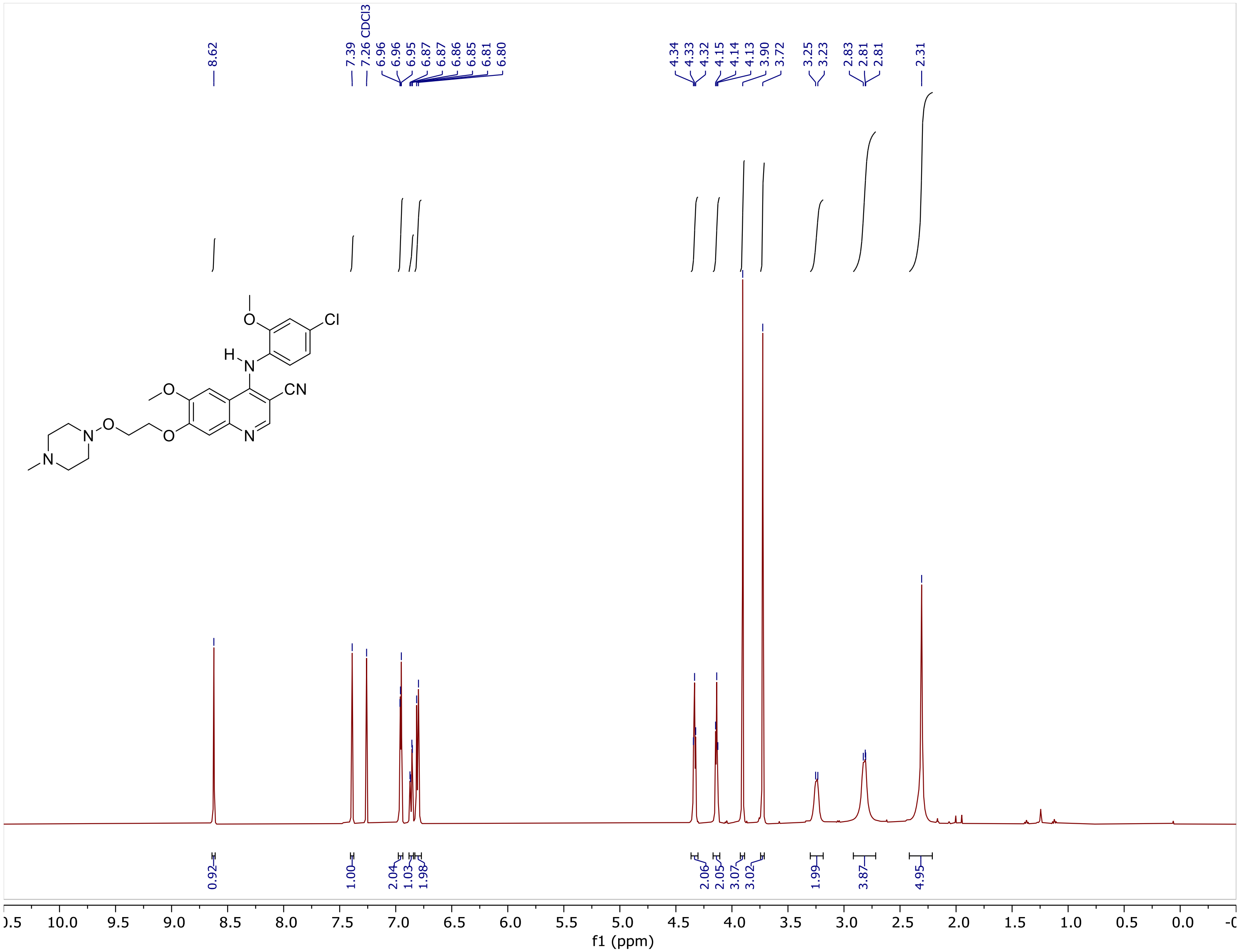

^{13}**C NMR** (126 MHz, $CDCl_3$) spectrum of 4-((4-chloro-2-methoxyphenyl)amino)-6-methoxy-7-(2-((4-methylpiperazin-1-yl)oxy)ethoxy)quinoline-3-carbonitrile (**15a**).

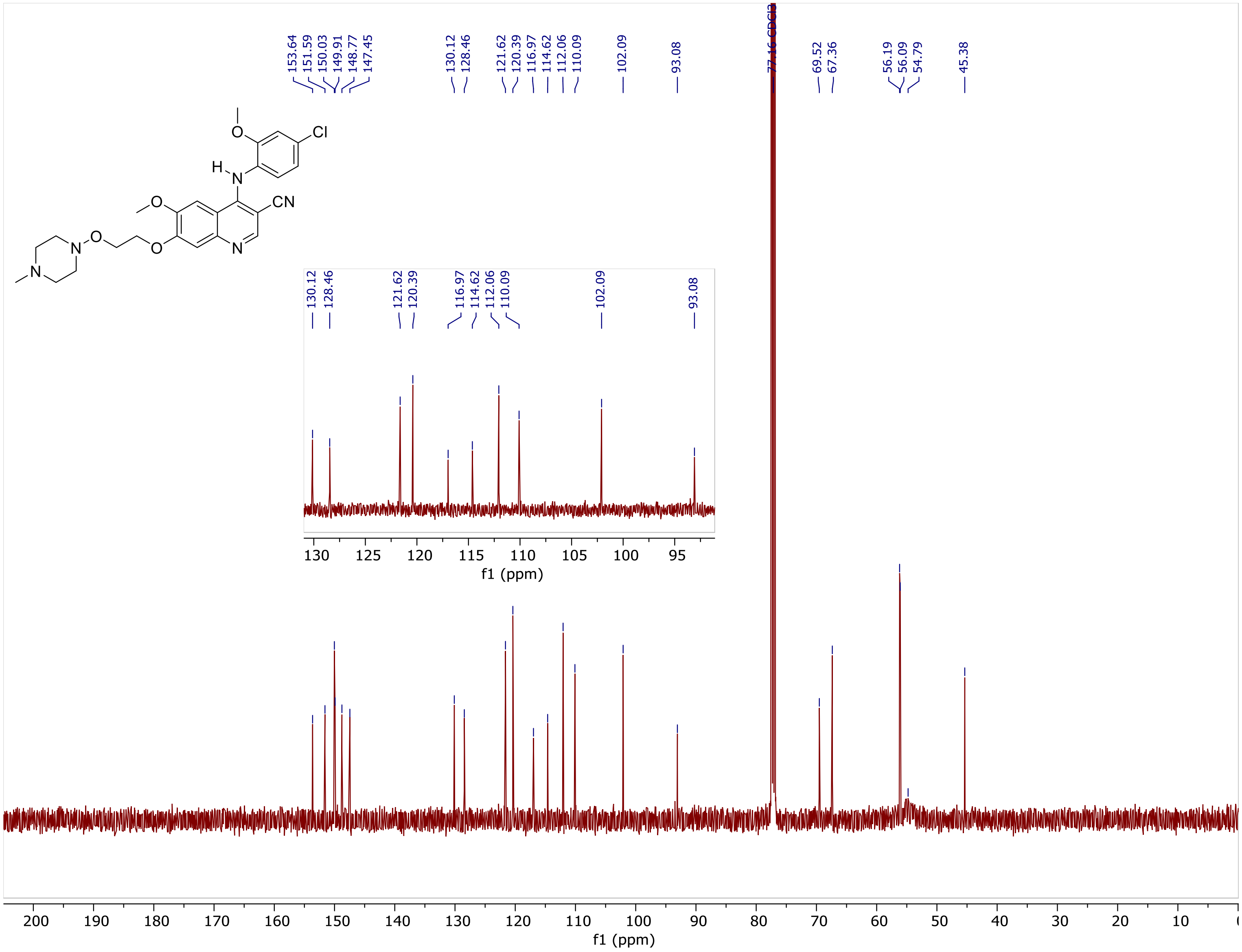

HSQC NMR (500 MHz, $CDCl_3$) spectrum of 4-((4-chloro-2-methoxyphenyl)amino)-6-methoxy-7-(2-((4-methylpiperazin-1-yl)oxy)ethoxy)quinoline-3-carbonitrile (**15a**).

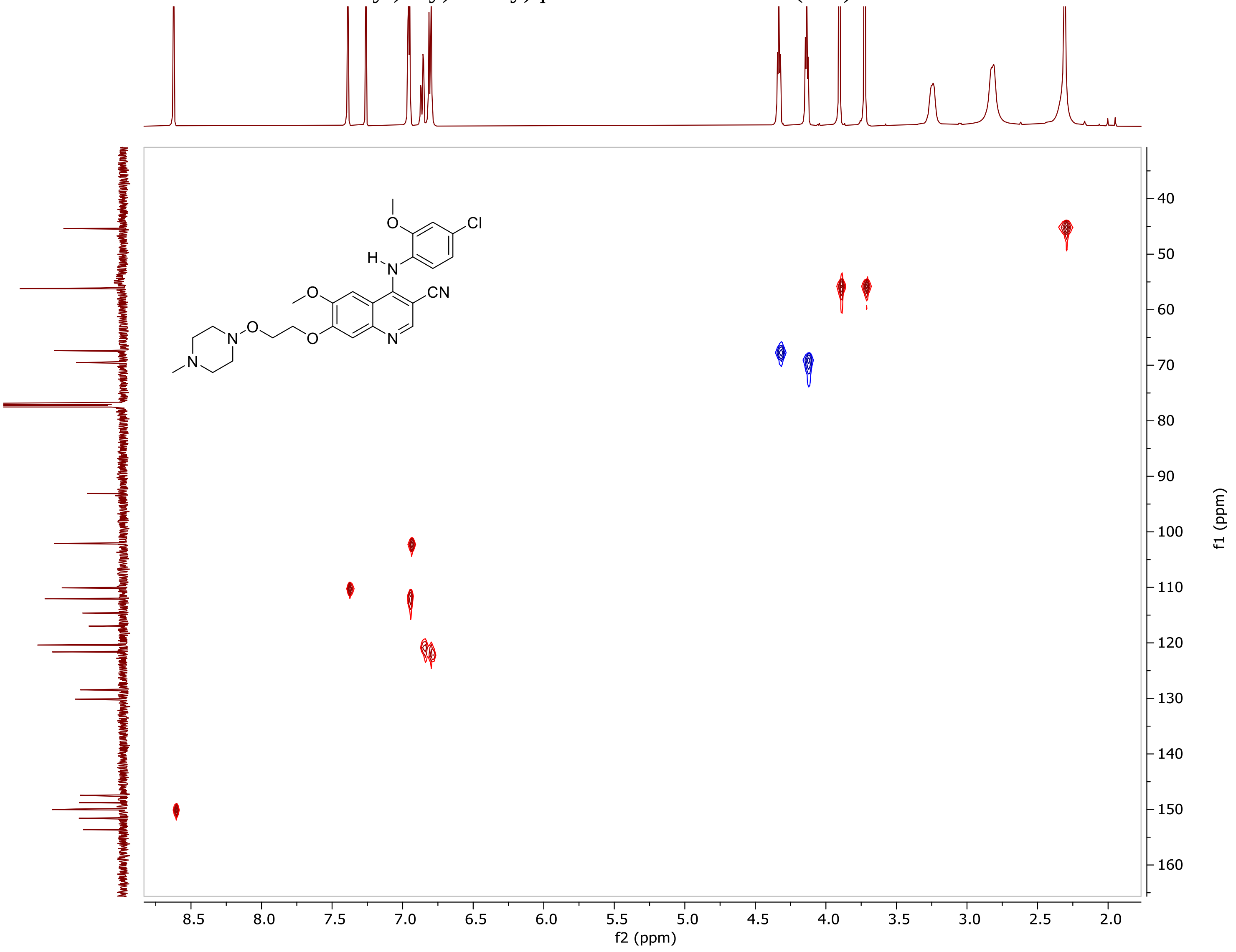

DQF-COSY NMR (500 MHz, $CDCl_3$) spectrum of 4-((4-chloro-2-methoxyphenyl)amino)-6-methoxy-7-(2-((4-methylpiperazin-1-yl)oxy)ethoxy)quinoline-3-carbonitrile (**15a**).

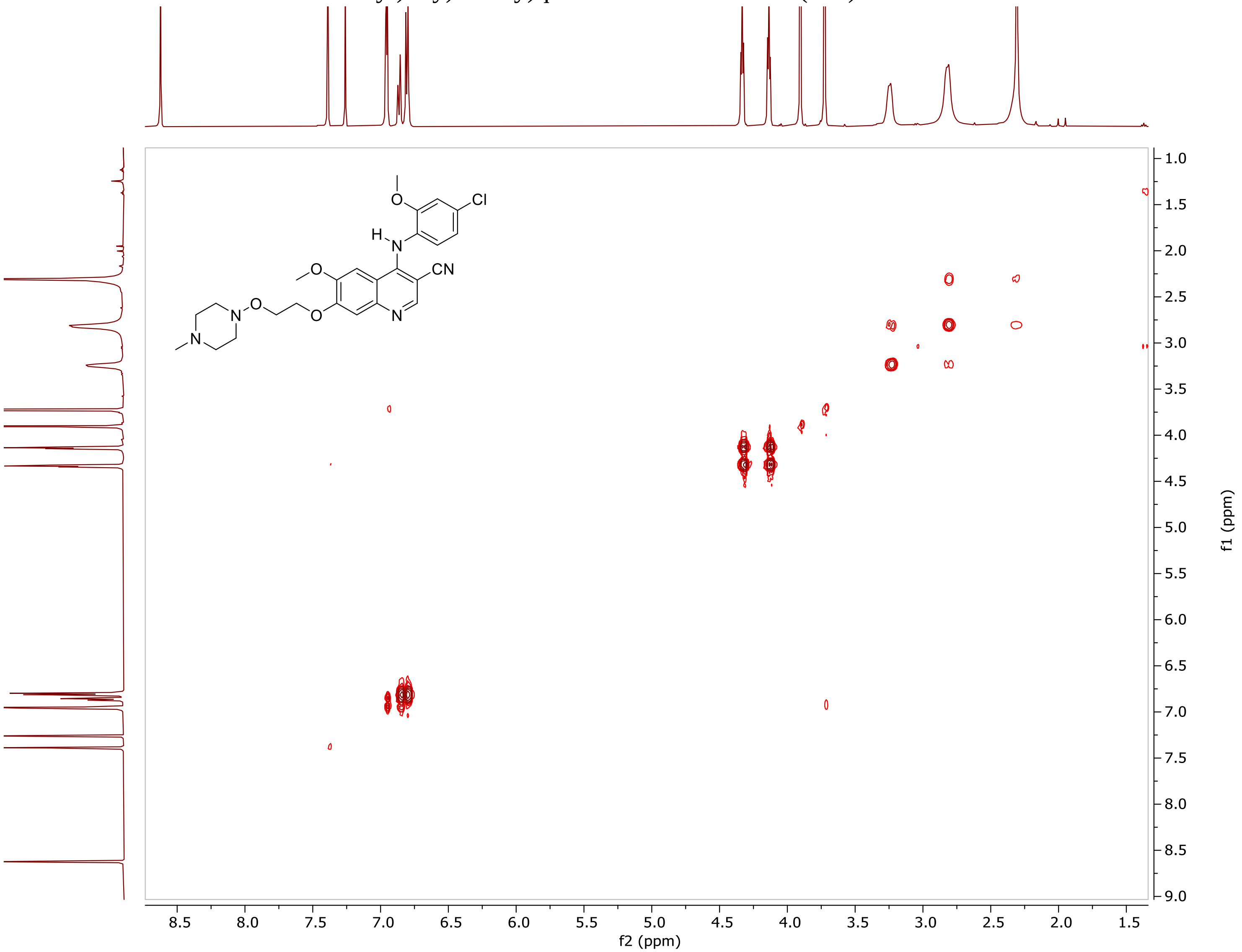

Expanded region of stacked ^{13}**C NMR** (126 MHz, $CDCl_3$) spectrum of 4-((4-chloro-2-methoxyphenyl)amino)-6-methoxy-7-(2-((4-methylpiperazin-1-yl)oxy)ethoxy)quinoline-3-carbonitrile (**15a**) at a) 328K and b) 298 K.

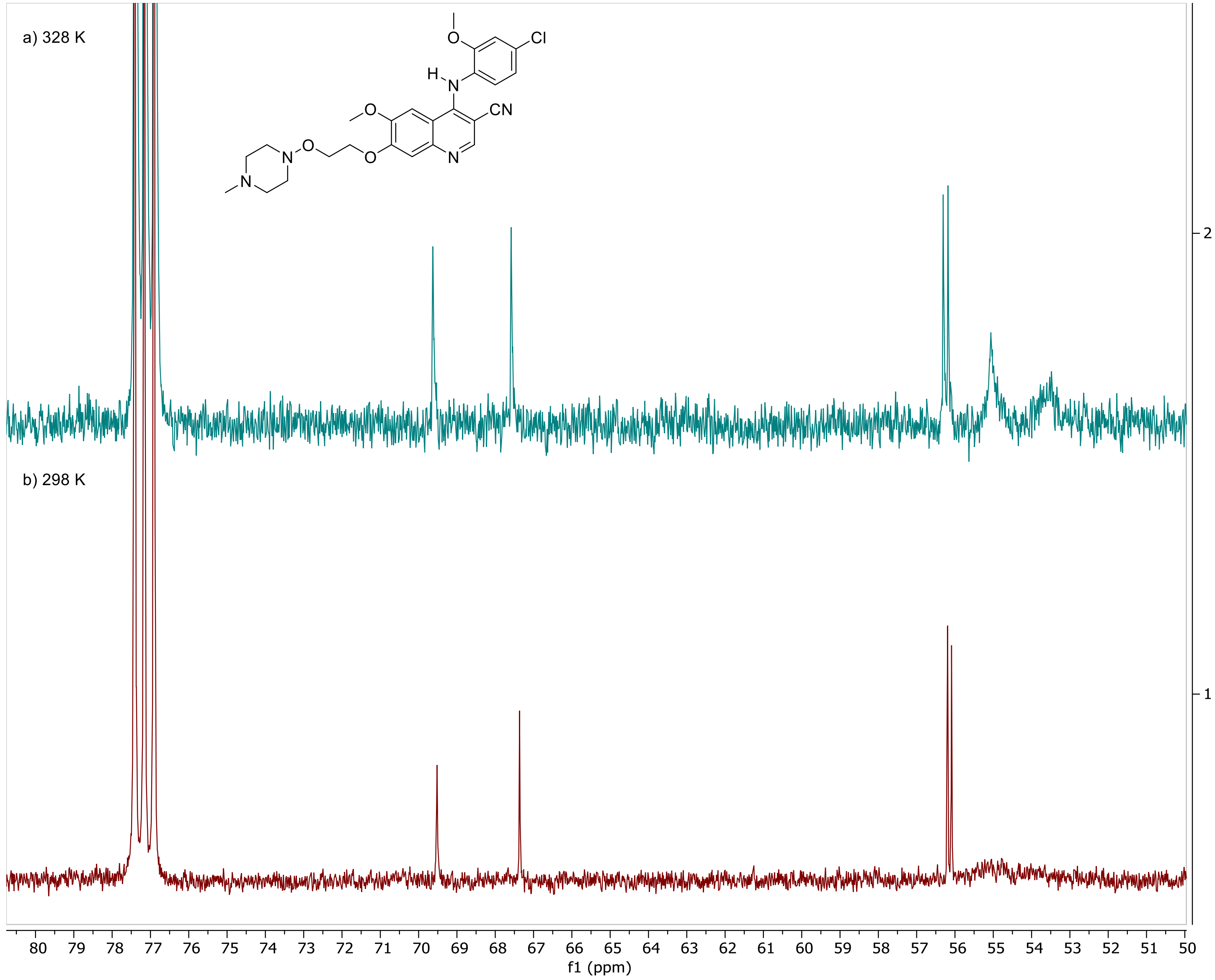

^{1}H NMR (500 MHz, $CDCl_3$) spectrum of 4-((5-chloro-6-methoxypyridin-2-yl)amino)-6-methoxy-7-(2-((4-methylpiperazin-1-yl)oxy)ethoxy)quinoline-3-carbonitrile (**16a**).

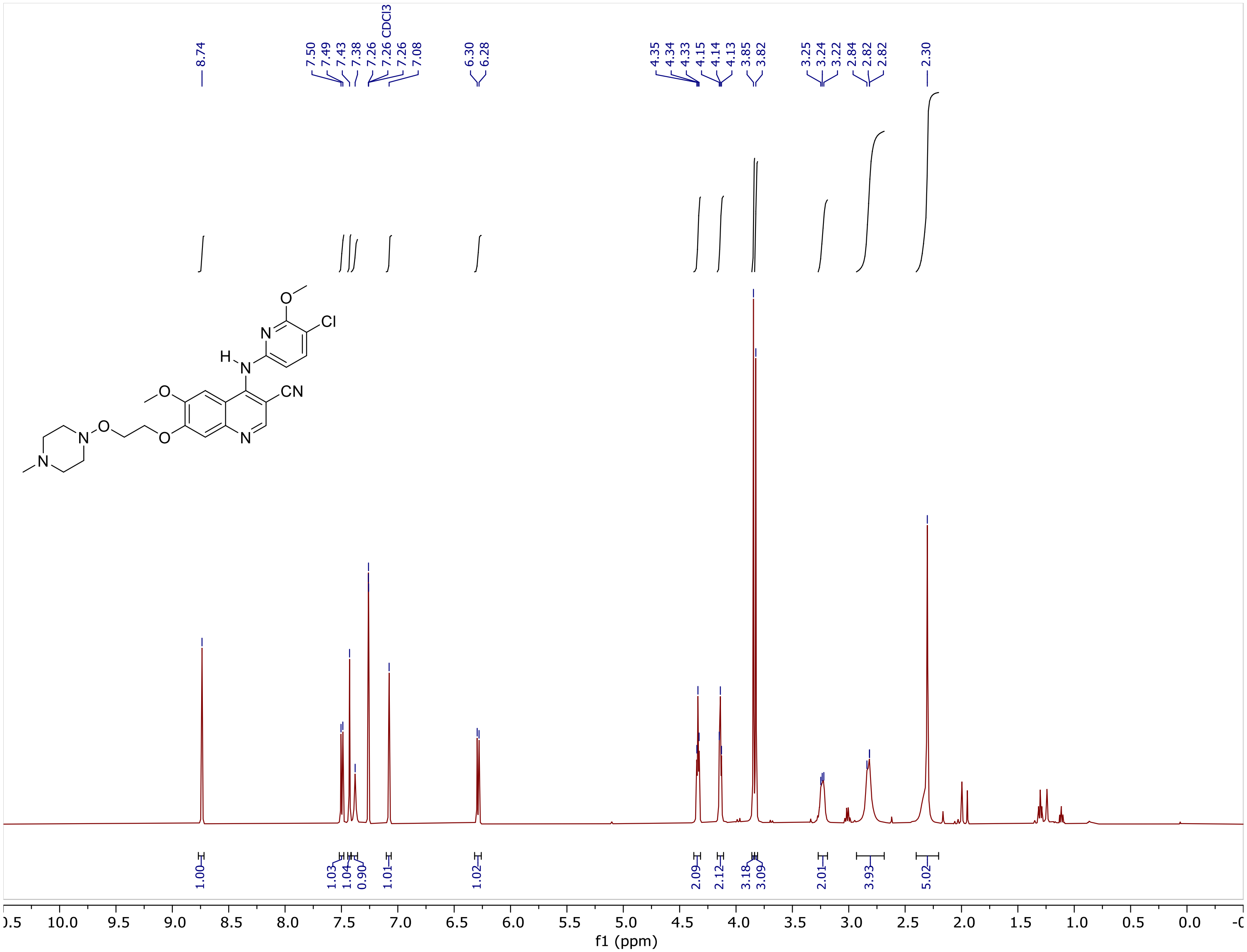

^{13}C NMR (126 MHz, $CDCl_3$) spectrum of 4-((5-chloro-6-methoxypyridin-2-yl)amino)-6-methoxy-7-(2-((4-methylpiperazin-1-yl)oxy)ethoxy)quinoline-3-carbonitrile (**16a**).

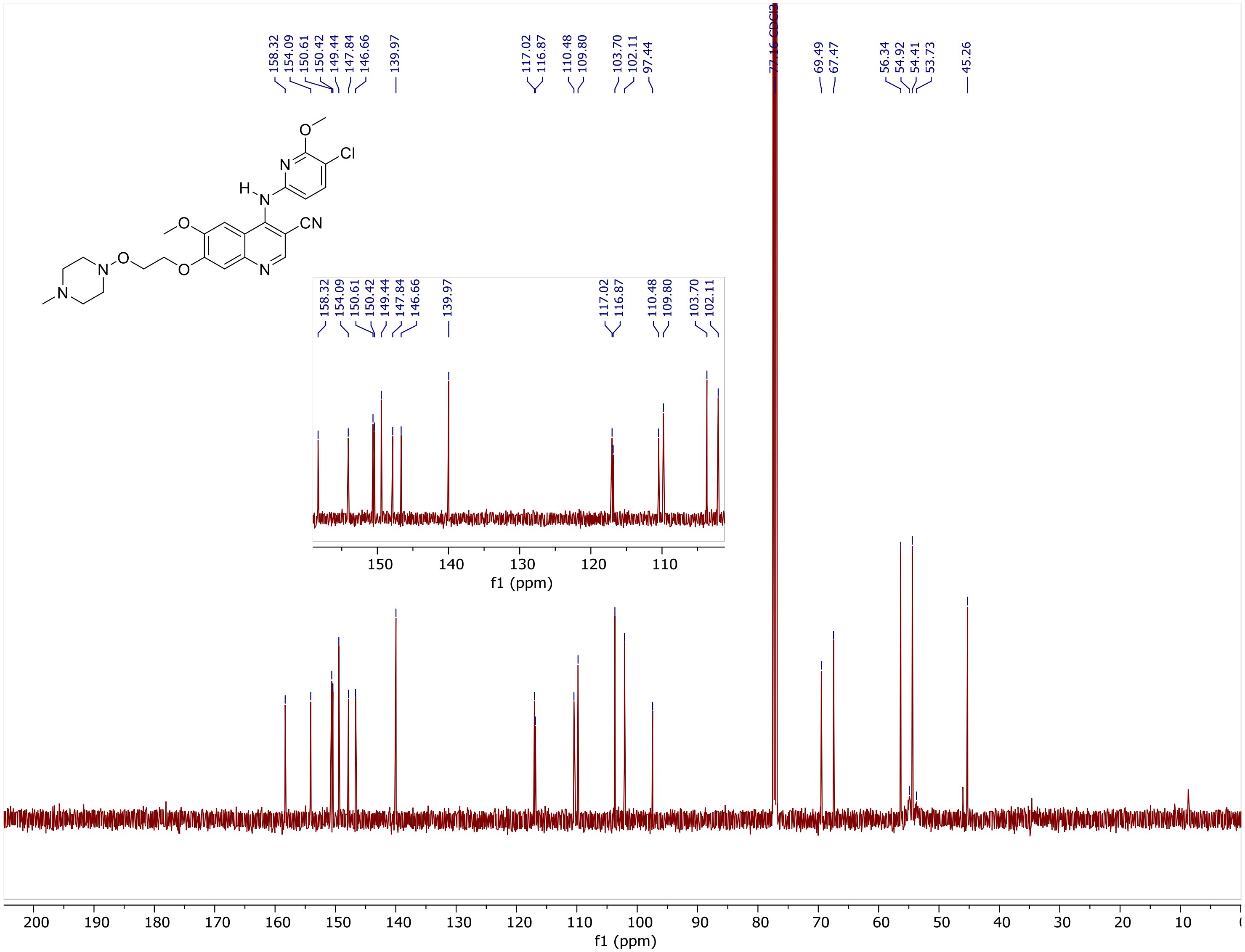

HSQC NMR (500 MHz, $CDCl_3$) spectrum of 4-((5-chloro-6-methoxypyridin-2-yl)amino)-6-methoxy-7-(2-((4-methylpiperazin-1-yl)oxy)ethoxy)quinoline-3-carbonitrile (**16a**).

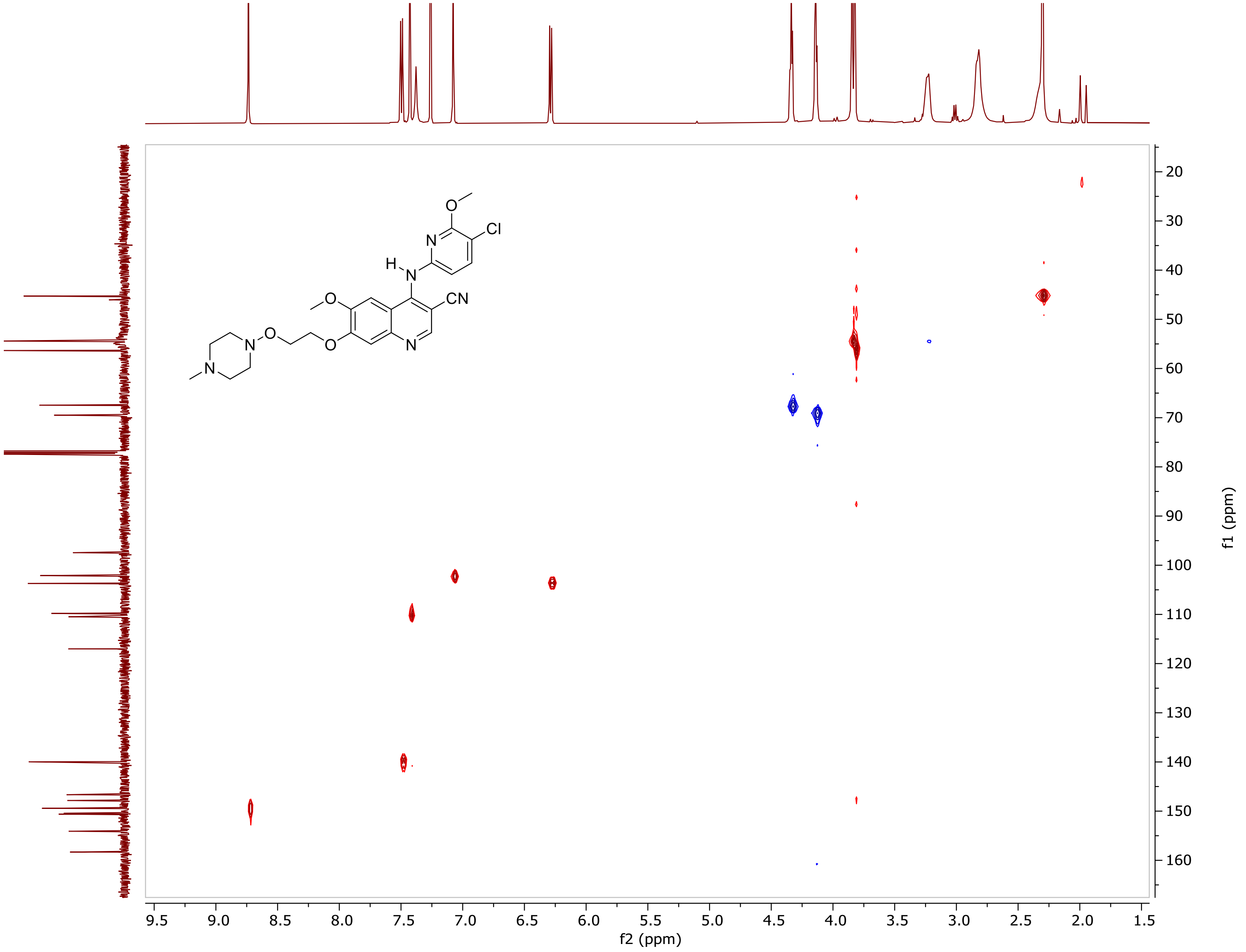

DQF-COSY NMR (500 MHz, $CDCl_3$) spectrum of 4-((5-chloro-6-methoxypyridin-2-yl)amino)-6-methoxy-7-(2-((4-methylpiperazin-1-yl)oxy)ethoxy)quinoline-3-carbonitrile (**16a**)

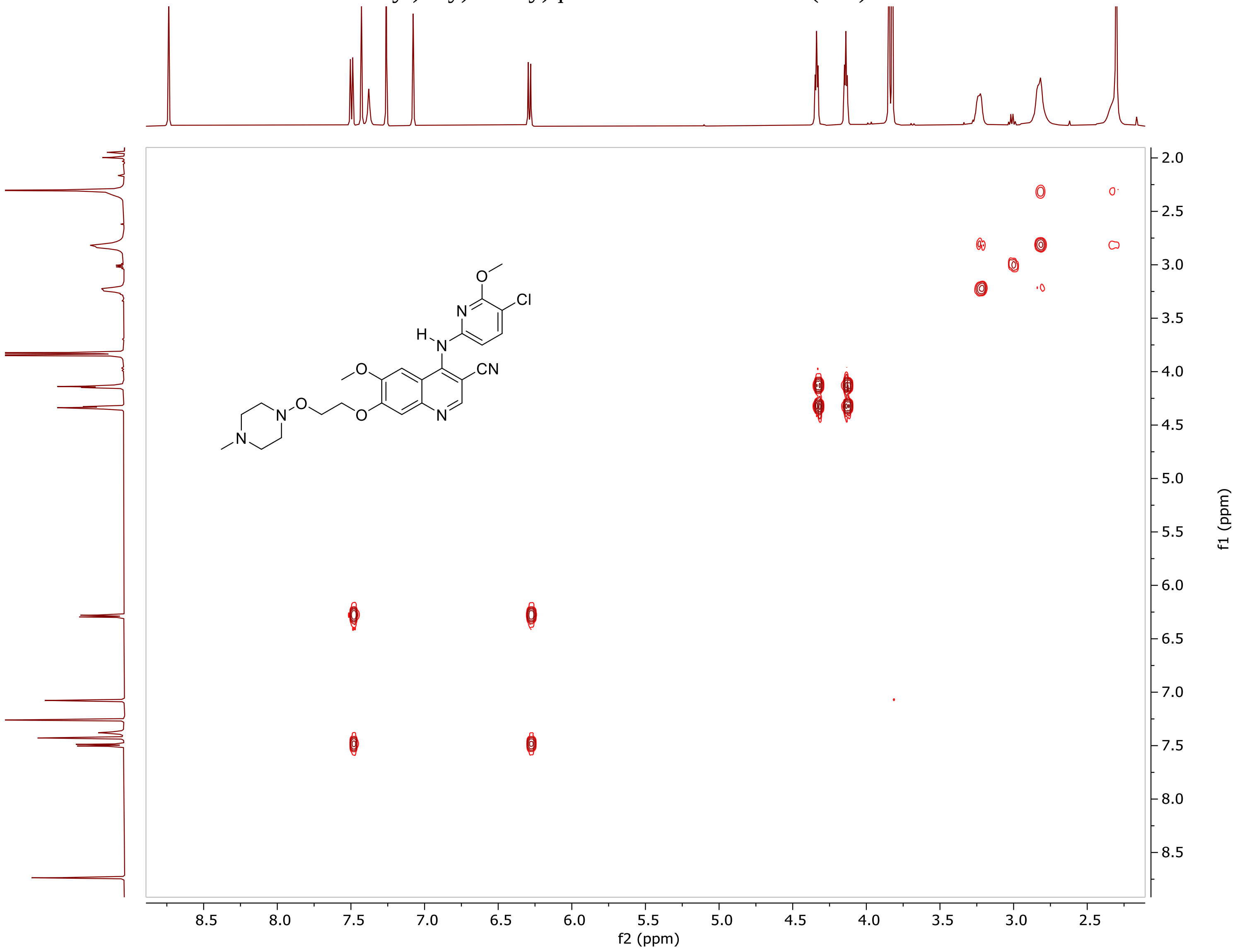

Expanded region of stacked ^{13}**C NMR** (126 MHz, $CDCl_3$) spectrum of 4-((5-chloro-6-methoxypyridin-2-yl)amino)-6-methoxy-7-(2-((4-methylpiperazin-1-yl)oxy)ethoxy)quinoline-3-carbonitrile (**16a**) at a) 328 K and b) 298 K.

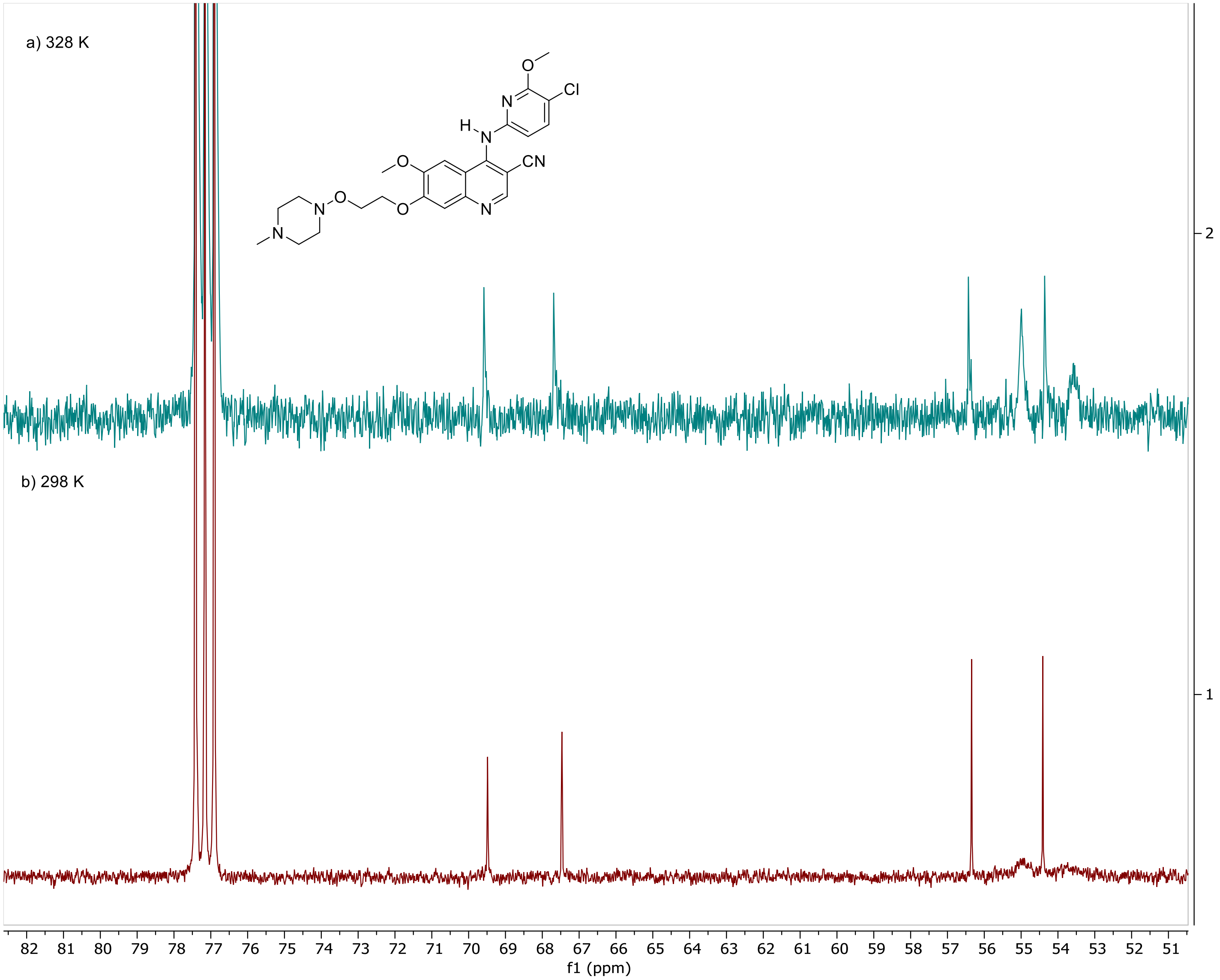

RT :0.00-10.00

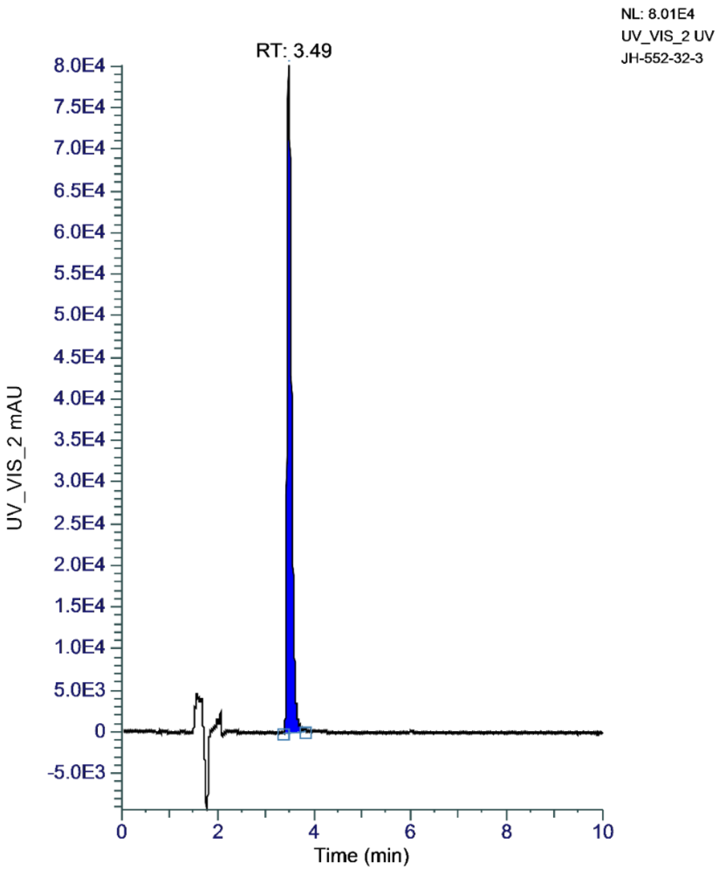

Figure S23. UHPLC trace of 4-((4-chloro-2-fluoro-5-methoxyphenyl)amino)-6-methoxy-7-(2-((4-methylpiperazin-1-yl)oxy)ethoxy)quinoline-3-carbonitrile (**8a**). UHPLC/UV area percent purity of compound **8a** at UV = 254 nm was determined to be 100% using automated Avalon peak area algorithm (peak list below).

Table S33. Peak List from Avalon peak area algorithm of compound **8a** at UV = 254 nm.

Retention Time (rt) (min)	Start rt (min)	End rt (min)	Peak Area	Area (%)
3.49	3.35	3.82	5.091E+5	100

Table S34. HPLC method. Mobile Phase A: CH_3CN. Mobile phase B: 0.1 % (v/v) formic acid in ultrapure H_2O. Flow rate: 1.000 [$mL \cdot min^{-1}$], injection volume: 1.50 µL.

Time (min)	Mobile Phase A (%)	Mobile Phase B (%)
0	30	70
10	30	70

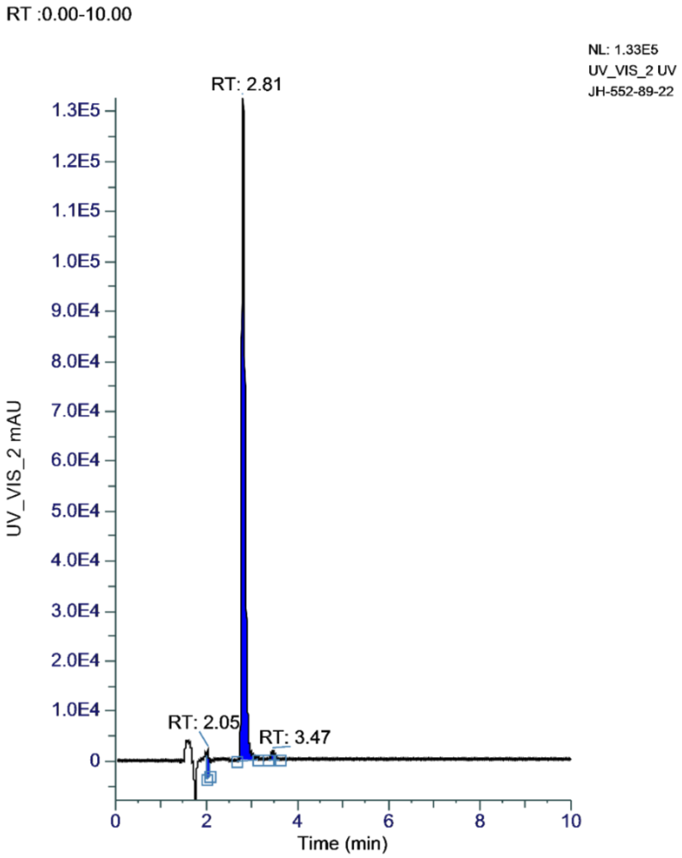

Figure S24. UHPLC trace of 4-((3-chloro-2-fluorophenyl)amino)-6-methoxy-7-(2-((4-methylpiperazin-1-yl)oxy)ethoxy)quinoline-3-carbonitrile (**11a**). UHPLC/UV area percent purity of compound **11a** at UV = 254 nm was determined to be 96.04% using automated Avalon peak area algorithm (peak list below).

Table S35. Peak List from Avalon peak area algorithm of compound **11a** at UV = 254 nm.

Retention Time (rt) (min)	Start rt (min)	End rt (min)	Peak Area	Area (%)
2.05	2.01	2.08	1.839E+4	2.64
2.71	2.68	3.14	6.697E+5	96.04
3.47	3.36	3.62	9.203E+3	1.32

Table S36. HPLC method. Mobile Phase A: CH_3CN. Mobile phase B: 0.1 % (v/v) formic acid in ultrapure H_2O. Flow rate: 1.000 [$mL\cdot min^{-1}$], injection volume: 1.20 µL.

Time (min)	Mobile Phase A (%)	Mobile Phase B (%)
0	30	70
10	30	70

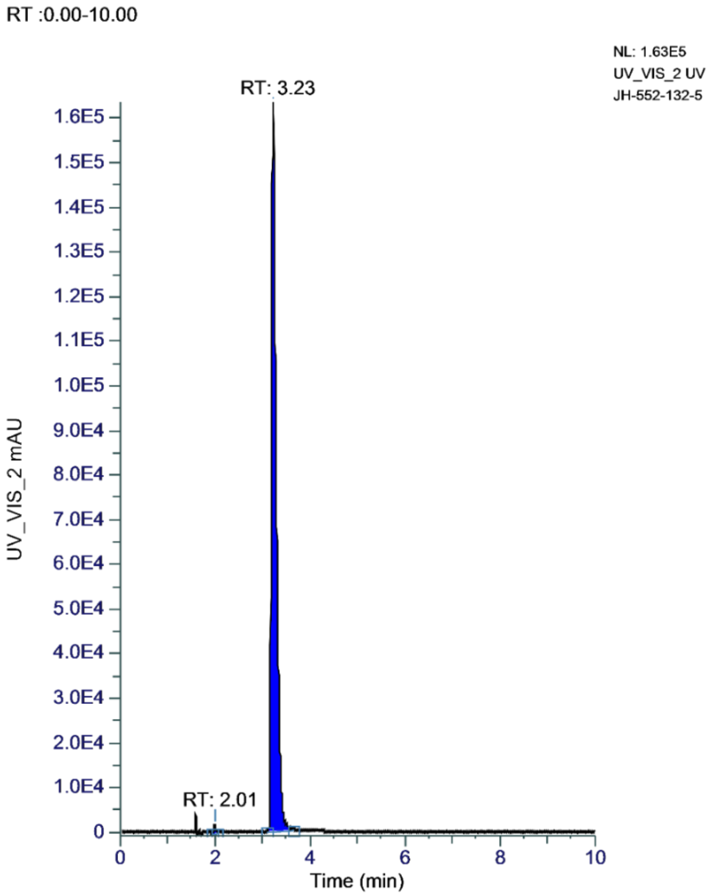

Figure S25. UHPLC trace of 4-((2-chloro-3-methoxyphenyl)amino)-6-methoxy-7-(2-((4-methylpiperazin-1-yl)oxy)ethoxy)quinoline-3-carbonitrile (**12a**). UHPLC/UV area percent purity of compound **12a** at UV = 254 nm was determined to be 99.63% using automated Avalon peak area algorithm (peak list below).

Table S37. Peak List from Avalon peak area algorithm of compound **12a** at UV = 254 nm.

Retention Time (rt) (min)	Start rt (min)	End rt (min)	Peak Area	Area (%)
2.01	1.95	2.05	1.732E+3	0.37
3.23	3.08	3.66	1.633E+5	99.63

Table S38. HPLC method. Mobile Phase A: CH_3CN. Mobile phase B: 0.1 % (v/v) formic acid in ultrapure H_2O. Flow rate: 1.000 [$mL \cdot min^{-1}$], injection volume: 0.75 µL.

Time (min)	Mobile Phase A (%)	Mobile Phase B (%)
0	25	75
10	25	75

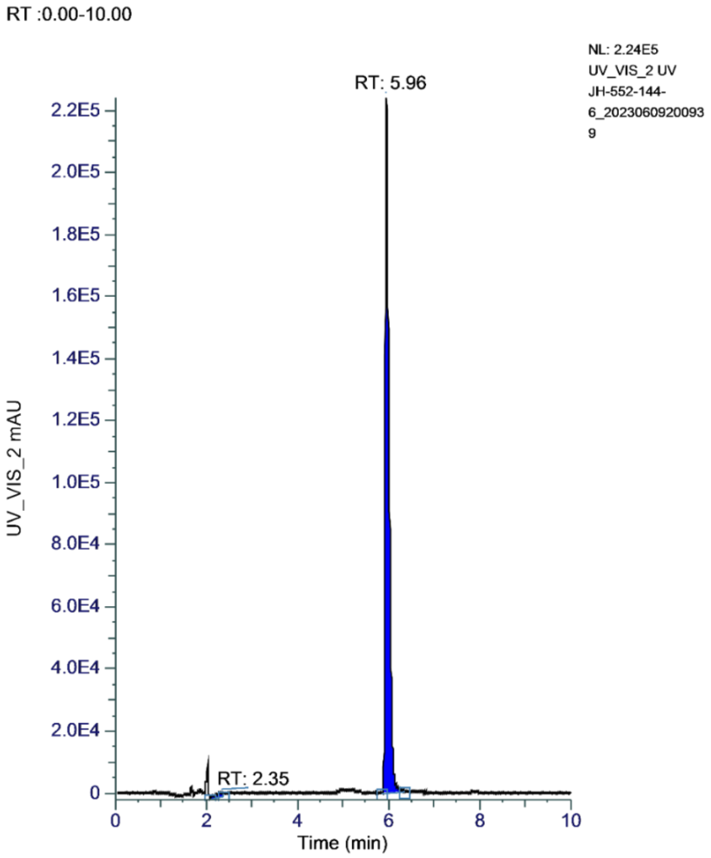

Figure S26. UHPLC trace of 4-((2-fluoro-3-methoxyphenyl)amino)-6-methoxy-7-(2-((4-methylpiperazin-1-yl)oxy)ethoxy)quinoline-3-carbonitrile (**13a**). UHPLC/UV area percent purity of compound **13a** at UV = 254 nm was determined to be 98.81% using automated Avalon peak area algorithm (peak list below).

Table S39. Peak List from Avalon peak area algorithm of compound **13a** at UV = 254 nm.

Retention Time (rt) (min)	Start rt (min)	End rt (min)	Peak Area	Area (%)
2.35	2.08	2.36	1.619E+4	1.19
5.95	5.85	6.34	2.24E+5	98.81

Table S40. HPLC method. Mobile Phase A: CH_3CN. Mobile phase B: 0.1 % (v/v) formic acid in ultrapure H_2O. Flow rate: 1.000 [mL·min^{-1}], injection volume: 1.00 µL.

Time (min)	Mobile Phase A (%)	Mobile Phase B (%)
0	15	85
3	25	75
10	25	75

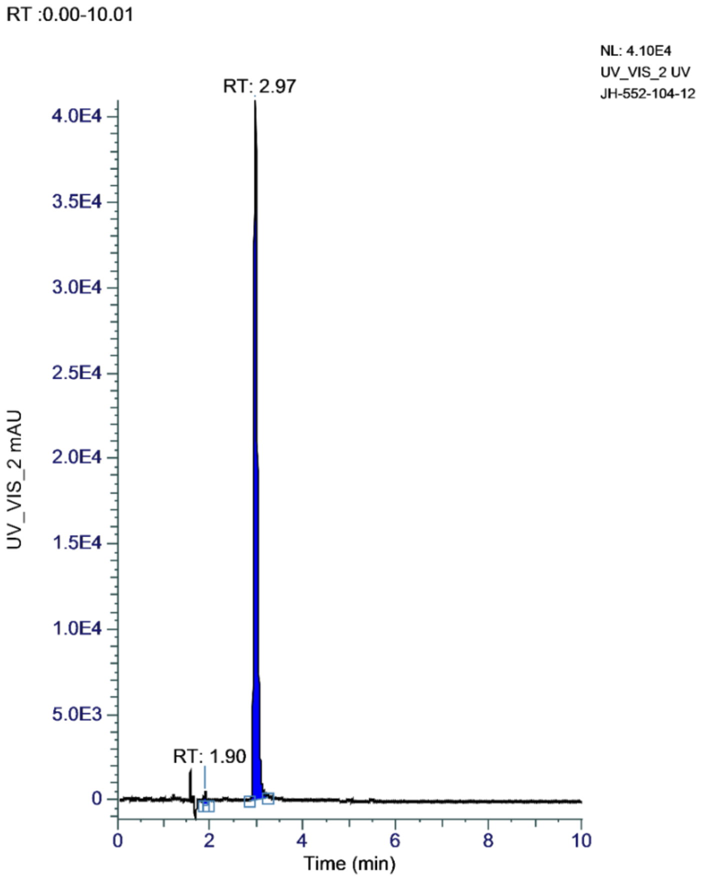

Figure S27. UHPLC trace of 4-((4-chloro-3-methoxyphenyl)amino)-6-methoxy-7-(2-((4-methylpiperazin-1-yl)ethoxy)quinoline-3-carbonitrile (**14a**). UHPLC/UV area percent purity of compound **14a** at UV = 254 nm was determined to be 98.64% using automated Avalon peak area algorithm (peak list below).

Table S41. Peak List from Avalon peak area algorithm of compound **14a** at UV = 254 nm.

Retention Time (rt) (min)	Start rt (min)	End rt (min)	Peak Area	Area (%)
1.90	1.84	1.96	2.888E+3	1.36
2.97	2.85	3.23	2.102E+5	98.64

Table S42. HPLC method. Mobile Phase A: CH_3CN. Mobile phase B: 0.1 % (v/v) formic acid in ultrapure H_2O. Flow rate: 1.000 [$mL\cdot min^{-1}$], injection volume: 0.30 µL.

Time (min)	Mobile Phase A (%)	Mobile Phase B (%)
0	30	70
10	30	70

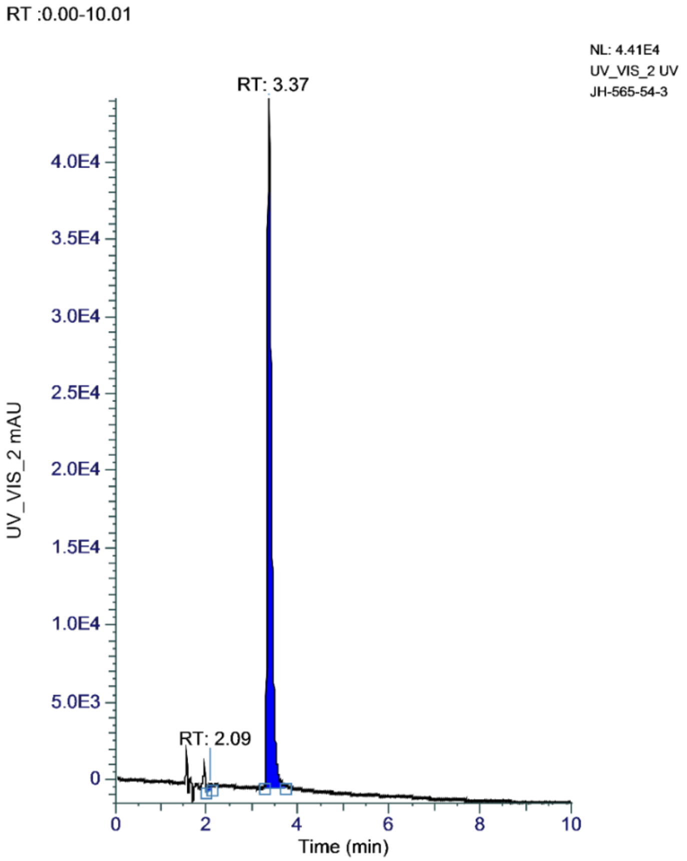

Figure S28. UHPLC trace of 4-((4-chloro-2-methoxyphenyl)amino)-6-methoxy-7-(2-((4-methylpiperazin-1-yl)oxy)ethoxy)quinoline-3-carbonitrile (**15a**). UHPLC/UV area percent purity of compound **15a** at UV = 254 nm was determined to be 99.20% using automated Avalon peak area algorithm (peak list below).

Table S43. Peak List from Avalon peak area algorithm of compound **15a** at UV = 254 nm.

Retention Time (rt) (min)	Start rt (min)	End rt (min)	Peak Area	Area (%)
2.09	1.99	2.11	2.217E+3	0.80
3.37	3.26	3.73	2.766E+5	99.20

Table S44. HPLC method. Mobile Phase A: CH_3CN. Mobile phase B: 0.1 % (v/v) formic acid in ultrapure H_2O. Flow rate: 1.000 [mL·min^{-1}], injection volume: 0.80 µL.

Time (min)	Mobile Phase A (%)	Mobile Phase B (%)
0	30	70
10	30	70

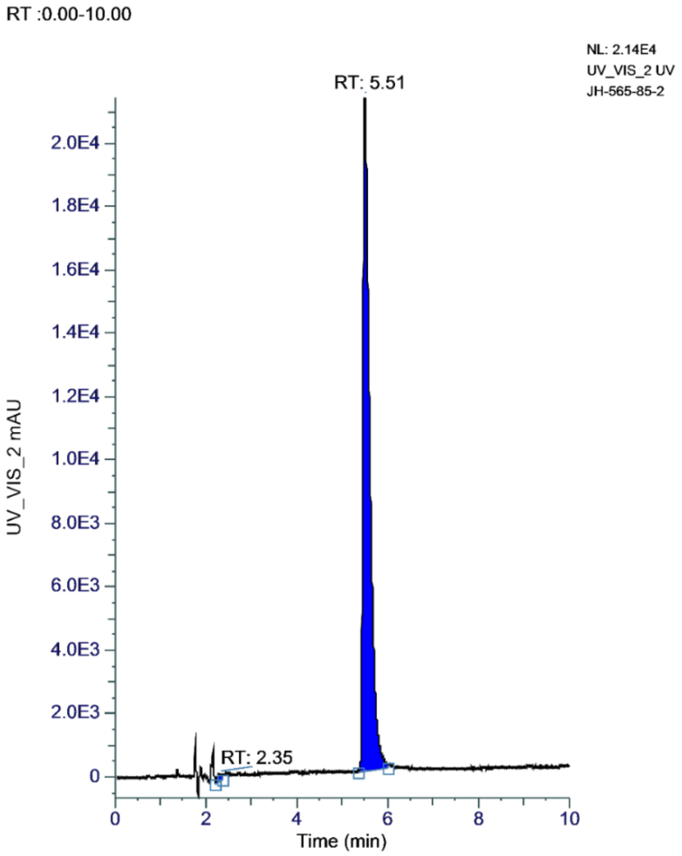

Figure S29. UHPLC trace of 4-((5-chloro-6-methoxypyridin-2-yl)amino)-6-methoxy-7-(2-((4-methylpiperazin-1-yl)oxy)ethoxy)quinoline-3-carbonitrile (**16a**). UHPLC/UV area percent purity of compound **16a** at UV = 254 nm was determined to be 99.42% using automated Avalon peak area algorithm (peak list below).

Table S45. Peak List from Avalon peak area algorithm of compound **16a** at UV = 254 nm.

Retention Time (rt) (min)	Start rt (min)	End rt (min)	Peak Area	Area (%)
2.35	2.21	2.36	1.317E+3	0.58
5.51	5.35	6.03	2.124E+5	99.42

Table S46. HPLC method. Mobile Phase A: CH_3CN. Mobile phase B: 0.1 % (v/v) formic acid in ultrapure H_2O. Flow rate: 1.000 [$mL\cdot min^{-1}$], injection volume: 0.50 µL.

Time (min)	Mobile Phase A (%)	Mobile Phase B (%)
0	30	70
10	30	70

5. X-Ray Crystal Structure of **28**

Crystallographic data has been deposited with the CCDC as entry 2334377.

Table S47. Crystal data and structure refinement for compound **28**

CCDC Identification code	2334377
Empirical formula	C13 H11 Cl N2 O2
Formula weight	262.69
Temperature	299 (2) K
Wavelength	0.71073
Crystal system	Tetragonal, I4(1)/a
Space group	P -1
Unit Cell Dimensions	a = 15.6473 (6) Å α = 90 °
	b = 15.6473 (6) Å β = 90 °
	c = 20.0849 (10) Å γ = 90 °
Volume	4917.5 (4) $Å^3$
Z	16
Density (calculated)	1.419 Mg/m^3
Absorption coefficient	0.305 mm^{-1}
F(000)	2176
Crystal Size	0.360 x 0.220 x 0.160 mm
Theta range for data collection	2.603 to 37.779 °
Index ranges	-26<=h<=26, -26<=k<=26, -34<=1<=34
Reflections collected	127498
Independent reflections	6597 [R(int) = 0.0722]
Completeness to theta = 25.242°	99.9%
Absorption correction	Semi-empirical from equivalents
Max. and min. transmission	0.7476 and 0.6508
Refinement method	Full-matrix least-squares on F^2
Data / restraints / parameters	6597 / 1/ 168
Goodness-of-fit on F2	1.044
Final R indices [I>2sigma(I)]	R1 = 0.0662, wR2 = 0.1478
R indices (all data)	R1 = 0.1294, wR2 = 0.1869
Largest diff. peak and hole	0.276 and -0.255 e.$Å^{-3}$

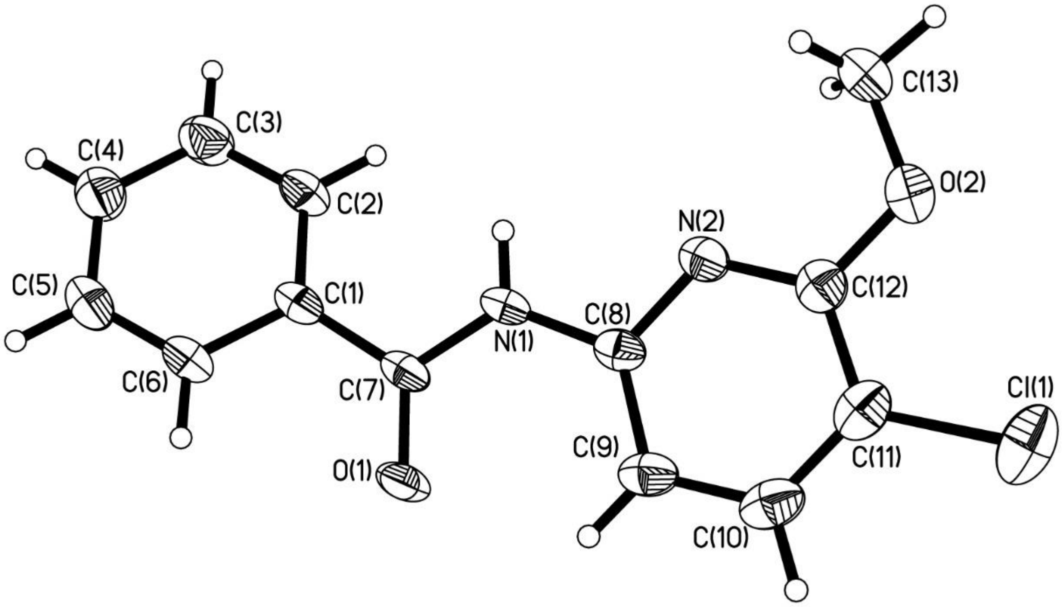

Figure S30. ORTEP drawing of compound **28** with 30% probability for thermal ellipsoids.

6. References

1) Hill, J.; Jones, R. M.; Crich, D. Atypical *N*-Alkyl to *N*-Noralkoxy Switch in a Dual cSRC/BCR-ABL1 Kinase Inhibitor Improves Drug efflux and hERG Affinity. *ACS Med. Chem. Lett.* **2023**, *14*, 1869-1875.

2) Davis, M. I.; Hunt, J. P.; Herrgard, S.; Ciceri, P.; Wodicka, L. M.; Pallares, G.; Hocker, M.; Treiber, D. K.; Zarrinkar, P. P. Comprehensive Analysis of Kinase Inhibitor Selectivity. *Nat. Biotechnol.* **2011**, *29*, 1046-1051.

3) Hill, J.; Jones, R. M.; Crich, D. Discovery of a Hydroxylamine-Based Brain-Penetrant EGFR Inhibitor for Metastatic Non-Small-Cell Lung Cancer. *J. Med. Chem.* **2023**, *66*, 15477-15492.

4) Bochevarov, A. D.; Harder, E.; Hughes, T. F.; Greenwood, J. R.; Braden, D. A.; Phillip, D. M.; Rinaldo, D.; Halls, M. D.; Zhang, J.; Friesner, R. A. Jaguar: a high-performance quantum chemistry software program with strengths in life and materials sciences. *Int. J. Quantum Chem.* **2013**, *113*, 2110-2142.

5) Abraham, M. H.; Abraham, R. J.; Acree, W. E. Jr., Aliev, A. E.; Leo, A. J.; Whaley, W. L. An NMR Method for the Quantitative Assessment of Intramolecular Hydrogen Bonding; Application to Physicochemical, Environmental, and Biochemical Properties. *J. Org. Chem.* **2014**, *79*, 11075-11083.
